# Revisited: Therapeutic and toxic blood concentrations of more than 1100 drugs and other xenobiotics

**DOI:** 10.1186/s13054-020-02915-5

**Published:** 2020-05-06

**Authors:** Martin Schulz, Achim Schmoldt, Hilke Andresen-Streichert, Stefanie Iwersen-Bergmann

**Affiliations:** 1 Drug Commission of German Pharmacists (AMK), Heidestraße 7, 10557 Berlin, Germany; 2 Institute of Pharmacy, Freie Universität Berlin, Kelchstraße 31, 12169 Berlin, Germany; 3 Institute of Legal Medicine, University Medical Centre Hamburg-Eppendorf, Butenfeld 34, 22529 Hamburg, Germany; 4 Department of Forensic Toxicology, Institute of Legal Medicine, Faculty of Medicine, University of Cologne, Melatengürtel 60-62, 50823 Cologne, Germany

**Keywords:** Critical care, Drug monitoring, Drug-related side effects and adverse reactions, Humans, Overdose, Intoxication, Xenobiotics/blood, Xenobiotics/toxicity

## Abstract

In order to assess the significance of drug/substance levels measured in intensive care medicine and clinical and forensic toxicology as well as for therapeutic drug monitoring, it is essential that a comprehensive collection of data is readily available. We revisited and expanded our 2012 compilation of therapeutic and toxic plasma concentration ranges as well as half-lives of now more than 1100 drugs and other xenobiotics.

Data have been abstracted from original papers, text books, and previous compilations and have been completed with data collected in our own forensic and clinical toxicology laboratories. We compiled the data presented in the table and the corresponding annotations over the past 30+ years. A previous compilation was completely double-checked, revised, and updated, if necessary. In addition, more than 200 substances, especially drugs who have been introduced since 2012 to the market as well as illegal drugs and other xenobiotics which became known to cause intoxications were added. We carefully referenced all data. Moreover, the annotations providing details were updated and revised, when necessary.

For more than 1100 drugs and other xenobiotics, therapeutic (“normal”) and, if data was available, toxic, and comatose-fatal plasma/blood concentrations as well as elimination half-lives were compiled in a table.

In case of intoxications, the blood concentration of the substance and/or metabolite better predicts the clinical severity of the case when compared to the assumed amount and time of ingestion. Comparing and contrasting the clinical case against the data provided, including the half-life, may support the decision for or against further intensive care. In addition, the data provided are useful for the therapeutic monitoring of pharmacotherapies, to facilitate the diagnostic assessment and monitoring of acute and chronic intoxications as well as to support forensic and clinical expert opinions.

## Introduction

Drug overdose has become the leading cause of death from injury in the USA [1]. In 2017, more than 2.6 million closed encounters were logged by the American Association of Poison Control Centers’ National Poison Data System and 2.12 million were related to human exposures. Although the total encounters showed a 3.8% decline from 2016, human exposures with more serious outcomes increased 3.1%. Consistent with previous years, the top five substance classes most frequently involved in all human exposures included three drug classes: analgesics (11.1%), household cleaning substances (7.4%), cosmetics/personal care products (6.8%), sedatives/hypnotics/antipsychotics (5.7%), and antidepressants (5.0%) [2]. According to the UK’s National Poisons Information Service Annual Report 2017/2018, around 160,000 hospital presentations occur annually as a result of poisoning, most frequently in the context of deliberate self-harm [3].

In case of intoxication or poisoning, the concentration of the ingested substance and/or metabolite in plasma/serum better predicts the clinical severity of the case and the potential outcome when compared to the assumed amount and time of ingestion. In addition, it is recommended that plasma concentrations of drugs having a narrow therapeutic range or with a highly variable response (such as in psychiatry) have to be measured and monitored. Apart from acute and chronic intoxications, it is indicated to draw blood samples for the following reasons: if doses are high and borderline, if signs of over-dosage occur although the dose is within normal range (e.g. genetic polymorphism), if there is no efficacy although the dose is correct, or if medication non-adherence can be expected [4].

In general, blood concentrations of drugs at steady state are retrievable from the dosage and pharmacokinetic data. However, sufficient pharmacokinetic data are often not available. Moreover, searching, retrieving, reading, analysing, and interpreting the relevant pharmacological, toxicological, and critical care literature in case of acute intoxications in daily intensive care practice are time-consuming and may delay or even mislead optimal clinical decisions. Therefore, it makes sense to offer a carefully referenced compilation of therapeutic and toxic plasma concentration ranges, as well as half-lives, of a large number of drugs and other xenobiotics for quick and comprehensive information [4].

## Materials and methods

The data presented in the table and the corresponding annotations (Additional file 1) have been developed over the past 30+ years. A previous compilation [4] has been completely revised and updated where necessary. In addition, more than 200 substances, especially drugs that have been introduced since then were added. Furthermore, we included new psychoactive substances (NPS), marketed as ‘legal highs’, as well as other illegal drugs which became known to cause intoxications. All data were carefully referenced. Moreover, the annotations providing details were updated and revised, if necessary.

Reviews, text books, compilations of other authors (mainly [5–25]), and most importantly, original publications concerning individual drugs, pharmacokinetic studies, and case reports have been used to set up and keep the database updated. Experience gained over more than 30 years from working in the clinical and forensic toxicological field contributed to the data presented.

The substances were selected by clinical and toxicological aspects, by frequency of prescribing or (mis-)use and other matters in the area of intensive care medicine as well as clinical and forensic toxicology.

The following clinical categories were used for grouping analytical data:

- Therapeutic: blood (plasma or serum) concentrations observed following therapeutically effective doses; no or only minimal side effects (drugs); “normal”: concentrations associated with no or only minimal toxic effects (other xenobiotics or environmental exposure) or achieved by a typical dosing regimen (illegal drugs).
- Toxic: blood (plasma/serum) concentrations producing toxicity or clinically relevant adverse effects.
- Comatose-fatal: blood (plasma/serum for comatose) concentrations and whole blood (for fatal) concentrations reported to have caused coma and death, respectively. Whether published data for deaths refer to levels measured ante-mortem or post-mortem (femoral or heart blood) is, however, often unknown.

## Results and discussion

For more than 1100 drugs and other xenobiotics, therapeutic (“normal”) and, if data was available, toxic, and comatose-fatal plasma/blood concentrations and elimination half-lives were compiled in one table (Additional file 1).

**Fig. 1:**
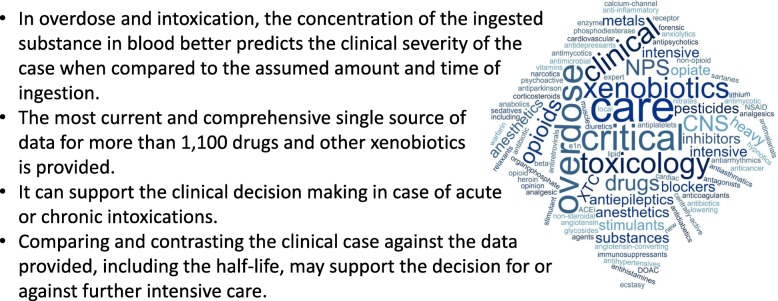
Summary findings

The compilation includes data for centrally active substances (e.g. anaesthetics, antidepressants, antiepileptics, antiparkinson drugs, antipsychotics, anxiolytics, hypnotics, lithium, opioids, sedatives, stimulants), cardiovascular drugs (e.g. angiotensin-converting enzyme inhibitors, angiotensin receptor antagonists [sartanes], antiarrhythmics, anticoagulants, antihypertensives, antiplatelets, beta blockers, calcium-channel blockers, cardiac glycosides, diuretics, lipid lowering drugs, nitrates), antimicrobial agents (e.g. antibiotics, antimalarials, antimycotics, antiretrovirals), and anabolics, non-opioid analgesics including non-steroidal anti-inflammatory drugs, antiasthmatics, anticancer drugs, antidiabetics, antihistamines, corticosteroids, immunosuppressants, local anaesthetics, muscle relaxants, phosphodiesterase inhibitors, and vitamins, among others (Fig. 1).

In addition and if data were available, other relevant xenobiotics such as controlled substances, illegal and recreational drugs, including new psychoactive substances, heavy metals, and pesticides, among others were listed.

To the best of our knowledge, this compilation is the most current and comprehensive single source of data, necessary to support the clinical decision-making and assessing blood concentrations in case of acute or chronic intoxications. Inter-individual deviation is, however, high. Therefore, any data listed can only be taken as an orientation.

Often, it is not possible to find the threshold between the therapeutic and toxic concentration for the specific patient. This is for instance the case if tolerance (especially true for all opioids), drug/drug-interactions, or additional diseases are involved. In order to keep the overall context clear, we preferred not to go into further details.

Many data about “comatose” or even “fatal” plasma/blood concentrations are consciously oriented on life threatening or lethal intoxications where low plasma/blood concentrations were detected. Considering these data may prevent underestimation of actual or potential dangers in clinical cases. Many intoxicated patients survived even with significantly higher plasma concentrations.

It is also difficult to relate the concentrations to the clinical picture because the interval between intake of the drug and drawing a blood sample is frequently unknown. In any case, it is more reliable to have the correct concentration measured rather than assuming how much drug/substance has been taken.

For a variety of data representing lethal cases, it is not known whether ante- or post-mortem, that is, (venous) femoral or heart blood levels were measured. If this information is unknown, we refrained from mentioning this detail.

Elimination half-lives are statistically more reliable than data gathered in case of intoxications. Yet even with this data, substantial deviation occurs. In addition, most pharmacokinetic parameters are retrieved from healthy subjects after application of relatively low doses. The data indicated generally deals with the terminal elimination half-life.

## Conclusions

In case of acute or chronic intoxications, the concentration of the ingested substance and/or metabolite in blood much better predicts the clinical severity of the case when compared to the assumed amount and time of ingestion. Comparing and contrasting the clinical case against the data provided, including the half-life, may support the decision for or against further intensive care. In addition, the data provided are useful for the therapeutic monitoring of pharmacotherapies, to facilitate the diagnostic assessment and monitoring of acute and chronic intoxications as well as to support forensic and clinical expert opinions.

## Supplementary information

**Additional file 1.** Therapeutic (“normal”), toxic, and comatose-fatal blood-plasma concentrations (mg/L, if not stated otherwise) in man. A table containing data for more than 1100 drugs and other xenobiotics in man including annotations and references.

## Data Availability

Not applicable.
