# Supplementary material for: Revisited: Therapeutic and toxic blood concentrations of more than 1100 drugs and other xenobiotics
Source: Crit Care. 2020 May 6;24:195. doi: 10.1186/s13054-020-02915-5 (PMC7201985; doi:10.1186/s13054-020-02915-5)
Supplement: Supplementary file 1 — Additional file 1. Therapeutic (“normal”), toxic, and comatose-fatal blood-plasma concentrations (mg/L, if not stated otherwise) in man. A table containing data for more than 1100 drugs and other xenobiotics in man including annotations and references. [file 13054_2020_2915_MOESM1_ESM.pdf]

Additional file 1 to

Revisited: Therapeutic and toxic blood concentrations of more than 1,100 drugs and other xenobiotics

Martin Schulz, Achim Schmoldt, Hilke Andresen-Streichert and Stefanie Iwersen-Bergmann; Critical Care 2020

Therapeutic ("normal"), toxic, and comatose-fatal blood-plasma concentrations (mg/L) in man

Substance	Blood-plasma concentration (mg/L)			$t_{½}$ (h)	References
	therapeutic ("normal")	toxic (from)	comatose-fatal (from)		
Abacavir (ABC)	0.9-3.9[308]			appr. 1.5	[1, 2]
Abiraterone	appr. 0.02-0.2			12	[780]
5-ABP	see 5-(2-Aminopropyl) benzofuran				
Acamprosate	0.25-0.7[231]	1[311]		(3-) 13-20 (-33)[232]	[3-5]
Acebutolol[1]	0.2-2 (0.5-1.26)[1]		15-20	3-11	[6-8]
Acecainide	see (N-Acetyl-)Procainamide				
Acecarbromal(um)	10-20 (sum)	25-30			[8, 9], [13]
Acemetacin	see Indometacin				
Acenocoumarol	0.03-0.1[197]	0.1-0.15		3-11	[3], [9-11]
Acetaldehyde	0-30	100-125			[10, 11]
Acetaminophen	see Paracetamol				

Note on liability: The data and information have been extracted from scientific sources (primary and secondary literature) and have been carefully reviewed. Nevertheless, transcription errors cannot be excluded. No responsibility is taken for the correctness of the information or data included. As a matter of principle, this compilation should be used with expert knowledge.

Substance	Blood-plasma concentration (mg/L) therapeutic ("normal")	toxic (from)	comatose-fatal (from)	t½ (h)	References
Acetazolamide	(4-) 10-20[267]	25-30		2-6 (-13)	[3], [11-14]
Acetohexamide	20-70	500		1.3	[15], [779]
Acetone	(2-) 5-20	100-400; 2000[8]	550	(6-) 8-31	[11], [16, 17]
Acetonitrile			0.77	32	[11]
Acetyldigoxin[3]	see Digoxin				
Acetylfentanyl			0.23-0.27		[945-947]
Acetylmethadol	see Levacetylmethadol				
Acetylsalicylic acid (aspirin, ASS, ASA)	20-200[2]	300-350[2]	(400-) 500[2]	3-20[2, 37]	[28-34]
Acitretin	0.01-0.29 (-0.5)[112]			2-4 days	[35, 36], [948, 949]
ACNU	see Nimustine				
Acrivastine	-0.07			1-2	[8]
Acrylfentanyl			0.01-5 ng/g; 0.5-2.1 ng/mL		[950, 951], [1071]
Acyclovir	0.4-1.5[203]			2-5[83]	[3], [10], [37-39]
Adalimumab	≥ 4.9 (5-9)			appr. 14 days	[40], [787]
N-(adamantan-1-yl)-1-(5-fluoropentyl)-1H-indole-3-carboxamide	see STS-135				

Note on liability: The data and information have been extracted from scientific sources (primary and secondary literature) and have been carefully reviewed. Nevertheless, transcription errors cannot be excluded. No responsibility is taken for the correctness of the information or data included. As a matter of principle, this compilation should be used with expert knowledge.

Substance	Blood-plasma concentration (mg/L)			t½ (h)	References
	therapeutic (“normal”)	toxic (from)	comatose-fatal (from)		
Adipiodone(-meglumine)	850-1200			0.5	[41]
Adrenaline	see Epinephrine				
Adriamycin	See Doxorubicin				
Agomelatine	0.007-0.3[310]	0.6[311]		1-2	[4]
AH-7921			350 (30-9100) ng/mL[464]		[1011], [1041], [1071]
Ajmaline	(0.1-) 0.53-2.21 (?)		5.5[8]	1.3-1.6, 5-6	[3], [42], [952]
Albendazole	0.5-1.5[92]			8-9[92]	[43-46]
Albuterol	see Salbutamol				
Alcuronium	0.3-3[353]			3.3 ± 1.3	[47]
Aldrin	-0.0015	0.0035		50-167 days (as dieldrin)	[11], [48]
Alendronate (Alendronic acid)	< 0.005[322]			1-2; terminal: days[594]	[49-51], [999]
Alfentanil	0.03-0.6[6]		0.1-0.2	0.6-2.3[96]	[52-55], [1071]
Alfuzosine	0.003-0.06	0.12		3-9	[8], [780]
Alimemazine (Trimeprazine)	0.05-0.4	0.5	1-3.2	8	[56, 57]
Aliskiren	0.07-0.45[426, 620]			30-70	[47]
Alizapride	0.1-2			2-3	[15]

Note on liability: The data and information have been extracted from scientific sources (primary and secondary literature) and have been carefully reviewed. Nevertheless, transcription errors cannot be excluded. No responsibility is taken for the correctness of the information or data included. As a matter of principle, this compilation should be used with expert knowledge.

Substance	Blood-plasma concentration (mg/L)			t½ (h)	References
	therapeutic ("normal")	toxic (from)	comatose-fatal (from)		
Allobarbital	2-5	10	20	40-48	[8], [11], [13], [58], [779]
Allopurinol[354]	2-19			0.5-3	[11], [47]
Almotriptan	0.05-0.07			3-4	[47]
Alogliptin	0.06-0.87			21	[780]
Alphaprodine	0.87-1			1.6-2.6	[11]
Alprazolam	0.005-0.05 (-0.08)[65]	0.1-0.4	0.3[252]	(6-) 12-15 (-20)	[4], [56], [59-63], [1231]
Alprenolol[48, 427]	0.025-0.14	1-2	40-48	2-7	[7, 8]
Aluminium	0.001 (< 0.005[234])	0.02-0.15	4.4[8]	160 days	[11], [47], [64-66], [953]
Amantadine	(0.06-) 0.2-0.6 (-1)	1.2[311]; 2.4[8]	21-48	9-15	[4], [47, 48]
5F-AMB (5F-AMB-PINACA)			0.3 ng/mL		[1037]
Amfebutamone	see Bupropion				
Amikacin	10-25[76]	30		(0.5-) 2-3	[67], [954]
Amiloride	0.017-0.026[620]			(6-) 17-24	[47]
Aminobenzoic acid	300-600	600			[66]
Aminoglutethimide	(0.05-) 7.5-25			10-15	[8], [11]
Aminophenazone	10-20			appr. 2-4	[15]

Note on liability: The data and information have been extracted from scientific sources (primary and secondary literature) and have been carefully reviewed. Nevertheless, transcription errors cannot be excluded.
No responsibility is taken for the correctness of the information or data included. As a matter of principle, this compilation should be used with expert knowledge.

Substance	Blood-plasma concentration (mg/L)			t½ (h)	References
	therapeutic ("normal")	toxic (from)	comatose-fatal (from)		
5-(2-Aminopropyl)benzofuran (5-APB)	appr. 0.1		0.1-4.2		[781], [955]
5-(2-Aminopropyl)indole (5-API, 5-IT)		0.015-0.59	0.7-18.6 mg/kg		[956, 957]
4-Aminopyridine (Fampridine)	0.025-0.075	0.14[8]; 0.2		3-3.5	[10, 11], [68]
5-Aminosalicylic acid (5-AS, 5-ASA)	see Mesalazine				
Amiodarone[261]	(0.5-) 1-2 (-2.5)	2.5-3		30-120 days	[3], [64], [69-71]
Amisulpride	0.1-0.4	0.64[311]	9.3[8]; 41.7[8]	12-20	[4], [8], [72], [958]
Amitriptyline[7, 48]<br>Amitriptyline + Nortriptyline	0.05-0.3<br>0.08-0.2	0.5-0.6<br>0.3[311]	1.5-2	(10-) 30-50	[4], [56], [73-83], [1084], [1231]
Amlodipine	0.003-0.015	0.088[8, 165]	0.1-0.2[8, 166]	34-50	[84-86], [780], [1064], [1151]
Ammonia	0.5-1.7				[11]
Amobarbital	1-5	(5-6) 10-30	13-96	15-30	[48], [87]
Amodiaquine	-0.05[270]			-[270]	[88]
Amoxapine	0.18-0.6[151]	3	5	8	[9]
Amoxicillin	0.5-1 (5-15)			1-2	[15]
Amphetamine	0.02-0.1	0.2	0.5-1	4-8 (7-34)[344]	[66], [89]
Amphotericin B	(0.1-) 0.2-3	(3-) 5-10	5.3[8]	24-48[110]	[47], [90-93]
Ampicillin	0.02-2 (2-20)			1	[67]

Note on liability: The data and information have been extracted from scientific sources (primary and secondary literature) and have been carefully reviewed. Nevertheless, transcription errors cannot be excluded.
No responsibility is taken for the correctness of the information or data included. As a matter of principle, this compilation should be used with expert knowledge.

Substance	Blood-plasma concentration (mg/L) therapeutic ("normal")	toxic (from)	comatose-fatal (from)	t½ (h)	References
Amrinone	1-2 (-4)			3-12	[3], [94, 95]
Amsacrine	(0.1-) 1-5.5			5-7	[96]
Amygdalin (Laetrile)	see Cyanide				[130], [250-256], [788-793]
Anileridine	< 0.5		0.9[8]		[11], [48]
Aniline	-0.02[328] (urine)	0.13[8, 355]	6	(2-) 3-4 (-7)	[11], [47, 48], [97-99]
Antimony	-0.01[329]	0.05-0.2			[64], [100], [959]
Antipyrine	see Phenazone				
5-ABP	see 5-(2-Aminopropyl) benzofuran				
5-API	see 5-(2-Aminopropyl)indole				
Apixaban	appr. 0.03-0.3		0.39[8]	9-14	[794-797], [960]
Apomorphine	0.002-0.02[204]			appr. 0.75	[3], [10]
Aprindine[48]	1-2	2-3		13-50	[15], [58]
Aprobarbital	4-20	30-40	50	14-34	[11], [47], [58]
Aripiprazole[43]<br>Aripiprazole + Dehydroaripiprazole	0.1-0.35<br>0.15-0.5	1[311, 345]	1.9[8]	60-80	[4], [101, 102], [798]
Armodafinil[607]	0.001-0.01[608]			10-20	[47], [780]

Note on liability: The data and information have been extracted from scientific sources (primary and secondary literature) and have been carefully reviewed. Nevertheless, transcription errors cannot be excluded.
No responsibility is taken for the correctness of the information or data included. As a matter of principle, this compilation should be used with expert knowledge.

Substance	Blood-plasma concentration (mg/L) therapeutic ("normal")	toxic (from)	comatose-fatal (from)	t½ (h)	References
Arsenic	0.002-0.012 (-0.07)[283]	0.05-0.25	0.6-9.3 (-15)	10-14[633]	[11], [47], [64], [87], [100], [103], [799], [1230]
Articaine	< 1.5-2 (?)			0.3 (-1)	[104]
Ascorbic acid (Vitamin C)	4-15			days	[3], [105-108], [800]
Asenapine	0.001-0.005	0.01[311]		13-39	[4]
Aspirin	see Acetylsalicylic acid				
Astemizole	0.002-0.05[43]	14[8]		2-4 days[42]	[3], [109], [961]
Atazanavir (ATV)	> 0.15[293]			(4-) 6.5-8.6 (-11)	[2], [47], [110, 111]
Atenolol	0.1-1 (-2)[77]	2-3	27[8]	4-14[9]	[7], [42], [64], [112]
Atomoxetine	0.2-1[317]	2[311]	5.4[8]	appr. 4[356]	[4], [962]
Atorvastatin[542]	0.007-0.01 (-0.25)	0.05		11-24	[47], [780], [1063]
Atovaquone	13.9 ± 6.9 (> 15)			2-3 days	[113]
Atracurium(besylate)	0.1-0.5 (-5)			appr. 0.5	[47], [58]
Atropine	0.002-0.025[155]	0.03-0.1	0.2	2-6.5, 13-38	[11], [66], [114]
Avanafil	-[428]			6-20	[47]
Avobenzone	0.001-0.009[419]			(29-) 74-112 (-480)	[801], [1122]
Azapropazone (Apazone)	40-90			8-24	[8], [13]

Note on liability: The data and information have been extracted from scientific sources (primary and secondary literature) and have been carefully reviewed. Nevertheless, transcription errors cannot be excluded. No responsibility is taken for the correctness of the information or data included. As a matter of principle, this compilation should be used with expert knowledge.

Substance	Blood-plasma concentration (mg/L)			t½ (h)	References
	therapeutic ("normal")	toxic (from)	comatose-fatal (from)		
Azathioprine[10]	0.05-2			1-2 (-4)[11]	[13], [15], [123]
Azelastine	0.002-0.003 (-0.01)			22-25	[15]
Azelnidipine	< 0.03 ?			(9-) 14-20	[802, 803]
Azilsartan medoxomil[429]	appr. 4-5[620]			12-20	[47]
Azithromycin	appr. 0.04-1			50-60 (2-4 days)	[115-119]
Aztreonam	1-10 (50-250)			1.5-2	[11]
Baclofen	0.08-0.4 (-0.6)	1.1-3.8	6-9.6	6.8 ± 0.7 (2-8)	[9], [11], [47], [804]
Bambuterol	see Terbutaline				
Barbexaclone	active metabolite = Phenobarbital (see there)				[12]
Barbital	2-20	20-50	50	57-120	[13], [15]
Barium	-0.001		0.37; 16-23[8]	10-18	[47]
BDF	See Bromobenzo-difuranylisopropylamine				
Benazepril	0.003-0.007			10.5	[780]
Bendrofluazide	0.05-0.1			appr. 3	[42]
Bendroflumethiazide	0.02-0.09[430, 620]			2.4-3.8	[47], [1152]

Note on liability: The data and information have been extracted from scientific sources (primary and secondary literature) and have been carefully reviewed. Nevertheless, transcription errors cannot be excluded. No responsibility is taken for the correctness of the information or data included. As a matter of principle, this compilation should be used with expert knowledge.

Substance	Blood-plasma concentration (mg/L)			t½ (h)	References
	therapeutic ("normal")	toxic (from)	comatose-fatal (from)		
Benoxaprofen	-50			19-39	[3]
Benperidol	0.001-0.01	0.02[311]		4-8	[4], [8]
Benzbromarone	2-10			2-4	[927, 928]
Benzene	-0.0002[271]		0.95[8]	9-24	[47], [58]
Benzhexol	see Trihexyphenidyl				
Benzonatate		2.5	0.8[8]; 4-35[8]	appr. 1-3	[8], [47], [963]
Benzoylecgonine[357]	-0.1		1	4-5	[8]
Benzphetamine	0.025-0.5	0.5	14[8]		[8], [11], [47]
Benztropine	0.01-0.18	0.05	0.2-0.7	12-24	[8], [11], [47]
Benzydamine	-[563]			6-23 (oral); 14-35 (topical)	[47]
Benzyl alcohol	-[431]	18[8, 194]		-[195]	[3], [47], [120, 121]
Benzylpenicillin	1.2-12			1	[41], [67]
N-Benzylpiperazine	0.01-1.2	0.7-6.3	8.3-20	4-6	[47]
Bepridil	0.6-2.5			33-42 (30-130)	[8], [122]
Beryllium	-0.0003				[58]
Betacarotene	4-6[196]				[3], [124]

Note on liability: The data and information have been extracted from scientific sources (primary and secondary literature) and have been carefully reviewed. Nevertheless, transcription errors cannot be excluded.
No responsibility is taken for the correctness of the information or data included. As a matter of principle, this compilation should be used with expert knowledge.

Substance	Blood-plasma concentration (mg/L)			t½ (h)	References
	therapeutic ("normal")	toxic (from)	comatose-fatal (from)		
Betaxolol	0.005-0.05		36[8]	14-22	[7], [47], [125]
Bethanidine	0.02-0.5			9-10	[3], [58]
Bevantolol	0.2-2			2	[8]
Bezafibrate	-15			2	[3]
Bicalutamide	1.5-17.5 (-25)[163]			(3-) 7-10 days	[126]
Biperiden	0.001-0.0065[406]	0.013[311]	0.25[8]	18-24	[4], [56]
Bismut (Bismuth)	< 0.05 (-0.1)	0.05-0.1		days	[47], [64]
Bisoprolol	0.01-0.1	0.2	0.17[8]	10-12	[7], [780], [964], [1064]
Bopindolol	0.001-0.015[54]			4-8[54]	[7], [965]
Borate	0-7	20	200	12-27	[47], [66]
Boron	0.8-6	20-50	50-150		[58]
Bornaprine	0.0007-0.0072[313]	0.014[311]		appr. 30	[4]
Bosentan	0.7-1.6[620]			3.5-5.1	[47]
Brallobarbital (Brallobarbitone)	4-8	8-10	15	20-40	[8], [13]
Bretylium	0.8-2.4			6-11	[3], [58]
Brexiprazole	0.04-0.14	0.28[311]		91	[4]

Note on liability: The data and information have been extracted from scientific sources (primary and secondary literature) and have been carefully reviewed. Nevertheless, transcription errors cannot be excluded.
No responsibility is taken for the correctness of the information or data included. As a matter of principle, this compilation should be used with expert knowledge.

Substance	Blood-plasma concentration (mg/L) therapeutic ("normal")	toxic (from)	comatose-fatal (from)	t½ (h)	References
Brivaracetam	0.5-0.9	1.8[311]		7-11	[4]
Brodifacoum		0.02	0.03-0.17; acute: 3.9[8]	20-60 days	[58], [127]
Bromadiolon		0.02		3-6 days (early), 10-24 days (late phase)	[11], [47], [58]
Bromazepam	(0.05-) 0.08-0.2	0.3-0.4	(1-) 2	8-22 (-35)	[4], [59]
Bromide	75-100 (-300)	500-1500; 3000[8, 242]	2000	9-15 days	[47], [58], [128-130]
Bromisoval	10-20	30-40		appr. 4[28, 105]	[13], [58]
Bromobenzodifuranylisopropylamine (Bromo-DragonFLY, BDF)		0.5-2 ng/mL	4.7 ng/mL[8]		[967, 968]
Bromocriptine	0.1-0.3 (-4) ng/mL[314]	8 ng/mL[311]		appr. 38	[4]
25B-NBOMe (2-(4-bromo-2,5-dimethoxyphenyl)-N-[(2-methoxyphenyl)methyl]ethanamine)		0.038	0.06-0.66		[966]
Bromoxynil		20	79[8, 433]; 137[8, 432]		[58], [969]
Bromperidol	0.012-0.02	0.03[311]		20-36	[4]
Brompheniramine	0.005-0.015	0.2[8]		2-10 (-20)	[15], [47]
Brotizolam	0.001-0.01 (-0.02)	0.02[311]	0.01-0.03[8]; 0.21	4-10	[4], [47], [131], [970]
Budesonide	appr. 0.009-0.045			2-3	[780]

Note on liability: The data and information have been extracted from scientific sources (primary and secondary literature) and have been carefully reviewed. Nevertheless, transcription errors cannot be excluded.
No responsibility is taken for the correctness of the information or data included. As a matter of principle, this compilation should be used with expert knowledge.

Substance	Blood-plasma concentration (mg/L) therapeutic ("normal")	toxic (from)	comatose-fatal (from)	t½ (h)	References
Budipine	appr. 0.1-0.3			30	[15]
Buflomedil	appr. 0.2-0.5 (-1.0)	15-25	25-50; 275[8]	2-4	[11], [42], [58]
Bumetanide	0.001-0.005			0.8-1.5	[780], [1205]
Bunitrolol	0.001-0.015			2-6	[7]
Bupivacaine	(0.25-) 0.5-1.5 (-2)	2-4	3.8[8]	0.5-3	[3], [132-134], [971]
Bupranolol[44]	-[434]			2-4	[7], [972]
Buprenorphine[340]	0.0005-0.005 (-0.014)[285]	0.01[311]; 0.03-0.1[339]	0.008-0.029	2-5 (i.v.); 18-49 (sublingual); appr. 19 (buccal)	[3, 4], [47], [135-141], [1006]
Bupropion (Amfebutamone)	0.01-0.02; 0.05-0.1[152]; 0.55-1.5[435]	1.2-2[246]; 2[311]	4[8]; 4.2[8]; 7.3[8]	(4-) 10-20; 17-47[436]	[4], [47], [80], [142-148], [973]
Buspirone[312]	0.001-0.004 (-0.01)[437]	0.03[311]	0.2 (0.05-1.1)[465]	1-5 (4-7)[438]	[4], [1012], [1194]
Busulfan	> 0.9[291]			2-4	[149-154]
Butabarbital	see Secbutabarbital				
Butalbital	1-5	10-15	15-30	30-40	[13], [15], [58]
1,4-Butanediol[466]	see 4-Hydroxybutyrate				[1013]
Butanone	-10	500			[58]

Note on liability: The data and information have been extracted from scientific sources (primary and secondary literature) and have been carefully reviewed. Nevertheless, transcription errors cannot be excluded.
No responsibility is taken for the correctness of the information or data included. As a matter of principle, this compilation should be used with expert knowledge.

Substance	Blood-plasma concentration (mg/L) therapeutic ("normal")	toxic (from)	comatose-fatal (from)	t½ (h)	References
Butaperazine	0.02-0.3 (-0.7)			12	[15], [779]
Butorphanol	0.0006-0.002			4-9	[8], [779]
Butriptyline	0.07-0.15	0.4-0.5	15	18-22	[15], [47], [779]
Butylone		0.2	0.13-129		[955], [1014, 1015]
Butylscopolamine	-0.7			4-5	[8]
Buturylfentanyl			99 ng/mL		[974], [1071]
Cabergoline	0.058-0.144 ng/mL[315]	0.39 ng/mL[311]		63-68	[4]
Cadmium	< 0.0003-0.0065	0.015-0.05		appr. 16 years	[47], [58], [100]
Caffeine (Coffein)	(2-) 4-10	15-20	80-180	2-10	[47], [64], [155-157]
Calcifediol (Calcidiol; see also Vitamin D)	0.01-0.05				[66]
Camazepam	0.1-0.6	2		20-24	[59]
Camostat[631]	87.1 ± 29.5 ng/mL[632]			100 ± 40 min[632]	[1227, 1228]
Camphor		0.3-0.4	1.7	2-8	[8], [47], [158]
Canagliflozin	0.17-1.3			(8-) 10-12 (-15)	[47], [780]
Candesartan	0.08-0.18 (-0.4)	0.54		5-7, 8-13	[15], [47], [780], [1064]
Canrenone	see Spironolactone				

Note on liability: The data and information have been extracted from scientific sources (primary and secondary literature) and have been carefully reviewed. Nevertheless, transcription errors cannot be excluded. No responsibility is taken for the correctness of the information or data included. As a matter of principle, this compilation should be used with expert knowledge.

Substance	Blood-plasma concentration (mg/L) therapeutic ("normal")	toxic (from)	comatose-fatal (from)	t½ (h)	References
Captopril	0.05-0.5 (-1)[620]	5-6	60	1-2 (-6)	[42], [47], [84], [159]
Carazolol[23]	-0.015			9	[7]
Carbachol	appr. 0.01 ?		3.6[8, 287]		[3], [160]
Carbamazepine[12]	2-8 (4-12)	10	20	12-60 (7-35)[140]	[12], [56], [161-166], [1231]
Carbaryl		5	6-27		[47], [167]
Carbenoxolone	appr. 5-30			8-20	[66]
Carbidopa	0.02-0.2[316]	0.4[311, 316]		2	[4]
Carbimazole	0.5-3.4[95]			3-6[95]	[8], [13]
Carbinoxamine	appr. 0.02-0.04			appr. 10-15	[168]
Carbocromene (Carbochromene)	0.8-2.4 (-3)			0.2-1.5	[8]
Carbon monoxide	-[200]	17-30%	50-60%		[10], [47], [58], [975-978]
Carbon tetrachloride	-0.07	0.12[8]; 7.1[8, 269]; 11[8]; 10-50	100-200	appr. 24; 42.6[8]	[58], [169-173]
Carboplatin	peak 10-25			2.5-6[106]	[47]
Carbromal(um)[13]	2-10	15-20	40	7-15	[13], [58]
Carfentanil			0.12[8]; 0.145[8]; 0.221[8]; 1.3[8] ng/mL		[979, 980], [1071]

Note on liability: The data and information have been extracted from scientific sources (primary and secondary literature) and have been carefully reviewed. Nevertheless, transcription errors cannot be excluded.
No responsibility is taken for the correctness of the information or data included. As a matter of principle, this compilation should be used with expert knowledge.

Substance	Blood-plasma concentration (mg/L) therapeutic (“normal”)	toxic (from)	comatose-fatal (from)	t½ (h)	References
Cariprazine[439]	0.01-0.02	0.04[311]		48-120	[4], [47]
Carisoprodol[440]	2.5-10	40; 30-50[104]	8-65[8, 441]; 110[104]	0.9-2.4	[47], [56], [58], [982]
ß-Carotine	see Betacarotene				
Carteolol	0.01-0.1			3-7	[7]
Carvedilol	appr. 0.02-0.15 (-0.3)	0.47[8]		6-10	[3], [8], [95], [983]
Cathine	0.054-0.087[370]; 0.11-0.2[442]; 0.016-0.31[553]			1.8-8.6	[984, 985]
Cathinone	0.047-0.087[370]; 0.009-0.17[554]			0.7-2.3	[984, 985]
Cefaclor	13-35 (i.v. -900)			0.5-1 (-2)	[3], [8], [11]
Cefadroxil	-30			1-2	[8]
Cefalexin	-65			1-1.5	[41]
Cefaloridine	20-80			1.5	[41]
Cefalotin	see Cephalotin				
Cefamandole	1-5 (10-40-150)			0.5-1.2	[41], [58]
Cefazolin	-150			1.5-2	[8], [41], [67]
Cefdinir	-4			1.1-2.3	[8], [47]
Cefepime	-160			2	[8]

Note on liability: The data and information have been extracted from scientific sources (primary and secondary literature) and have been carefully reviewed. Nevertheless, transcription errors cannot be excluded.
No responsibility is taken for the correctness of the information or data included. As a matter of principle, this compilation should be used with expert knowledge.

Substance	Blood-plasma concentration (mg/L) therapeutic (“normal”)	toxic (from)	comatose-fatal (from)	t½ (h)	References
Cefetamet	-7			2-3	[8]
Cefixime	-7			3-4	[8]
Cefmenoxime	-200			1-2	[8]
Cefodizime	-400			2-4	[8]
Cefoperazone	-250			1-2 (-5)	[8], [174]
Cefotaxime	0.5-2 (10-50; i.v. -225)			1-1.5	[67]
Cefotetan	65-90			3.5	[41]
Cefotiam	-150[71]			0.7-1.5 (-2)	[41], [175]
Cefoxitin	-150			0.7-1	[41]
Cefpodoxime	-7			2-3	[8]
Cefsulodin	20-100			1.6-1.9	[3], [8], [11]
Ceftazidime	20-40 (50-200)			1-4	[3], [8], [11]
Ceftibuten	appr. 3-20			2-4	[8]
Ceftizoxime	40-160			6-9	[41]
Ceftriaxone	15-75			4.5-8.5	[67], [1186]
Cefuroxime	0.5-1 (10-60; i.v. -180)[243]			1.1-1.3	[3], [10], [58], [176, 177]

Note on liability: The data and information have been extracted from scientific sources (primary and secondary literature) and have been carefully reviewed. Nevertheless, transcription errors cannot be excluded.
No responsibility is taken for the correctness of the information or data included. As a matter of principle, this compilation should be used with expert knowledge.

Substance	Blood-plasma concentration (mg/L) therapeutic ("normal")	toxic (from)	comatose-fatal (from)	t½ (h)	References
Celecoxib[48]	0.36-0.8[371]			7-16	[47]
Celiprolol	0.05-0.5 (-1)			3-6	[58]
Cephalothin (Cefalotin)	-30			0.5-0.6	[3], [41]
Cerivastatin	0.002-0.04			1.5-3	[8]
Cetirizine	appr. 0.1-0.6	2-5		7-10	[15], [47]
Chinidine	see Quinidine				
Chinine	see Quinine				
Chloralhydrate[14]	1.5-15	40-50	60-100	6-10 (-30)	[47], [58]
Chlorambucil	0.15-0.3			1.5-3	[15]
Chloramphenicol	5-10 (-15)[59]	25	28-108; 180[8, 552]	2-6	[11], [47], [58], [178], [1069]
Chlordane	-0.001	0.0025	1-7	88 days	[11], [58]
Chlordecone		0.6-32		63-148 days	[47]
Chlordiazepoxide[15]	0.4-3	3.5-10 (-15); 20.5[8]	20[8]; 26[8]	6-27	[11], [47], [56], [58], [179, 180]
Chlorhexidine				appr. 12 (dermally in rats)	[47]
Chlormethiazole	see Clomethiazole				

Note on liability: The data and information have been extracted from scientific sources (primary and secondary literature) and have been carefully reviewed. Nevertheless, transcription errors cannot be excluded.
No responsibility is taken for the correctness of the information or data included. As a matter of principle, this compilation should be used with expert knowledge.

Substance	Blood-plasma concentration (mg/L) therapeutic (“normal”)	toxic (from)	comatose-fatal (from)	t½ (h)	References
Chlormezanone	(3-) 5-9 (-14)	appr. 20	18[8]; 53[8]	20-30 (-60)	[43], [47], [56], [181]
Chlorobutanol		75			[66]
2-(4-chloro-2,5-dimethoxyphenyl)-N-[(2-methoxyphenyl)methyl]ethanamine	see 25C-NBOMe				
Chloroform	20-50 (-100)[443]	appr. 70	33[8]; 64[8]; 69[8]; 91[8]	1.5	[11], [47], [58], [182]
Chlorophacinone			3.4-28; 26[8]	6-23 days	[47]
Chloroquine	0.02-0.5 (-0.7)	0.8-1	3; 8[8]; 15[8]; 18[8]	dose-dependent (3-14 (-30-60)) days	[30], [47], [56], [183], [806], [1232]
Chlorothiazide	2-18			0.5-2	[3], [11], [58], [780]
Chlorphenamine (Chlorpheniramine)	(0.003-) 0.01-0.02		1.1[8]	(12-) 15-25 (-43)[358]	[3], [11], [47], [58]
Chlorpromazine[66]	0.03-0.1 (-0.5)	0.6[311]; 1-2	3-4	10-30	[4], [56], [184, 185]
Chlorpropamide	30-250	200-750		25-60	[58], [66]
Chlorprothixene	0.02-0.3	0.4[311]	0.4-0.9	8-12	[4], [47], [798]
Chlorpyrifos		0.2	0.5 (0.14-1.2)[276]	56-74	[47], [186]
Chlortalidone	0.15-0.3 (-1.4)	appr. 2		44-48 (24-89)	[47], [58]
Chlortetracycline	1-5 (-10)	30		5-6	[9], [13, 14], [58]
Cholecalciferol	see Vitamin D				

Note on liability: The data and information have been extracted from scientific sources (primary and secondary literature) and have been carefully reviewed. Nevertheless, transcription errors cannot be excluded.
No responsibility is taken for the correctness of the information or data included. As a matter of principle, this compilation should be used with expert knowledge.

Substance	Blood-plasma concentration (mg/L)			t½ (h)	References
	therapeutic ("normal")	toxic (from)	comatose-fatal (from)		
Chromium	< 0.0005[388]	0.001-0.091 (-0.3) (inhalative exposition); 0.2-4.6 (after oral intake)	1[555]; 32[8]; 68[8]	3-4 years	[47], [805], [987]
Cibenzoline	0.2-0.4 (-0.9)	(0.5-) 1	3.4-23	(6)- 7-8 (-15)[83]	[3], [47], [187]
Cicletanine	appr. 1-2			5-23	[3], [8]
Ciclosporine A (Cyclosporine, CsA)	< 0.1-0.25	0.3-0.4[16]		10-27[169]	[188-193]
Cidofovir	appr. 7-43			2.5	[3], [8]
Cilazapril (Cilazaprilat)	0.003-0.09			30-50	[8]
Cilostazol	0.18-0.54			12	[780]
Cimetidine	0.25-3 (0.75-4)	19-50	110[8]	1.5-4	[47], [58], [194, 195]
Cinnarizine	0.04-0.33	7.4[8]	1.1[8]	12-34	[47]
Cinoxacin	appr. 15			1.5-4	[8]
Ciprofloxacin	2.5-4	11.5[8]		3-6 (-8)	[47], [196-199], [203]
Cisaprid	0.04-0.08			6-12	[8]
Citalopram	0.05-0.11	0.22[311]; 2.45[8]	3.4-10.5 (5-6[160])	25-48[170]	[4], [8], [47], [56], [200-202], [204-207], [807, 808], [1084]
Cladribine	appr. 0.006			0.1-0.2 (6.4-19.7)	[208-210]

Note on liability: The data and information have been extracted from scientific sources (primary and secondary literature) and have been carefully reviewed. Nevertheless, transcription errors cannot be excluded. No responsibility is taken for the correctness of the information or data included. As a matter of principle, this compilation should be used with expert knowledge.

Substance	Blood-plasma concentration (mg/L) therapeutic ("normal")	toxic (from)	comatose-fatal (from)	t½ (h)	References
Clarithromycin	appr. 0.2-2			3-7[217]	[8], [115], [118], [211-213]
Clemastine	appr. 0.001-0.002			10-32	[8], [47], [214]
Clenbuterol	0.0003-0.0006	0.003[8]	0.001[8, 424]	30-35	[47], [940-942]
Clindamycin	appr. 0.5-2[556]			2-3	[8], [47]
Clobazam[17]	0.03-0.3	0.5[311]	0.7[8, 444]; 3.9[8]	18-42	[4], [47], [59], [215]
Clobutinol	appr. 0.05-0.2			23-34	[3], [8]
Clodronate (Clodronic acid)	0.7-1[460]			(2.5-) 7-8;<br>terminal: days[594]	[49], [996, 997]
Clofibrate	50-250			10-18	[88]
Clomethiazole (Chlormethiazole)	0.1-5[446]	(2.8-) 4-15	10-214	(2-) 3-5 (-7)	[4], [9], [13], [47], [216, 217]
Clomipramine[48, 85]<br>Clomipramin + N-Desmethylclomipramin	(0.02-) 0.09-0.25 (-0.4)[226]<br>0.23-0.45	0.4-0.6<br>0.45[311]	1-2	16-60	[4], [76], [80], [218-223], [1084]
Clonazepam	(0.004-) 0.02-0.07[150]	0.08[311]; 0.1		20-60	[4], [60], [161], [224]
Clonazolam	-[421]	-[421]			[835]
Clonidine	0.001-0.002 (-0.004)	0.009[8];<br>0.025-0.05	0.023[8]; 0.23[8]; 5.2[8]	5-20	[47], [225-227]
Clopamide	appr. 0.06-0.18[620]			6-14	[47]
Clopenthixol (Clopentixol)	0.002-0.015	0.1-0.3		15-25	[8], [11]

Note on liability: The data and information have been extracted from scientific sources (primary and secondary literature) and have been carefully reviewed. Nevertheless, transcription errors cannot be excluded. No responsibility is taken for the correctness of the information or data included. As a matter of principle, this compilation should be used with expert knowledge.

Substance	Blood-plasma concentration (mg/L) therapeutic (“normal”)	toxic (from)	comatose-fatal (from)	t½ (h)	References
Clopidogrel[445]	0.001-0.006			1.5-4 (-7)	[47]
Clorazepate[15]	see Nordazepam			1-2	[164]
Clotiapin	0.007-0.15	0.12-0.38	0.3[8]; 1.3[8]	4-10	[47]
Clotiazepam	0.1-0.7			3-15	[8]
Cloxacillin	5-30 (-85)			0.5-1 (0.3-2)	[8], [11], [58]
Clozapine[136]	(0.1-) 0.35-0.6	0.6-1 (9.5[8])	1.2[8]; 2[8]; 5.2[8]	(6-) 12-16	[4], [72], [101], [228-240], [809]
25C-NBOMe (2-(4-chloro-2,5-dimethoxyphenyl)-N-[(2-methoxyphenyl)methyl]ethanamine)		0.16 ng/mL[8, 447]	0.8 ng/g[8, 448]		[47], [988, 989], [995]
Cobalamin	see Vitamin B_{12}				
Cobalt	0.0001-0.001	0.4-0.5	0.42[8]	2 (early); 38 (late phase)	[11], [47], [58]
Cocaine	0.05-0.3	(0.25-) 0.5-1 (-5.2)	0.9-21	0.5-1[18]	[13, 14], [47], [58], [241, 242], [990], [1083]
Codeine[48]	0.03-0.25	0.5-1[339]	0.45-2 (-48)	3-4	[3], [47], [243, 244]
Coffein (Coffeine)	see Caffeine				
Colchicine	0.0003-0.0025	0.005 (0.019[8])	0.009[8]; 0.024[8]; 0.066[8]	11-32 (-60)[143]	[10], [47], [245-248]
Colecalciferol	see Vitamin D				

Note on liability: The data and information have been extracted from scientific sources (primary and secondary literature) and have been carefully reviewed. Nevertheless, transcription errors cannot be excluded. No responsibility is taken for the correctness of the information or data included. As a matter of principle, this compilation should be used with expert knowledge.

Substance	Blood-plasma concentration (mg/L) therapeutic ("normal")	toxic (from)	comatose-fatal (from)	t½ (h)	References
Colistin	1-5[86]			1.5-2 (-5)	[47]
Copper	0.6-1.5	0.8-2	2.5-5 (-66)	26 days	[66]
Cotrimoxazole	see Sulfamethoxazole and Trimethoprim				
Coumatetralyl		0.009[8]; 0.12[8]		12-13[452]	[47], [249]
Cresol (Methylphenols)	-[449]	appr. 50	120		[58], [991]
Cromolyn (Cromoglycate)	appr. -0.01			1-1.5	[3]
5F-Cumyl-PEGACLONE			0.09-0.45 ng/mL[8, 565]		[1081, 1082]
Cyamenazine	0.0009-0.016		1.8[8]; 3.6[8]; 9.8[8]	10-12	[47]
Cyanide	-[177]	(0.2-) 0.5; 0.514[8]	1-3	0.7-2.1 (whole blood); appr. 19[184]	[47], [130], [250-256], [788-793]
Cyclizine	0.1-0.25	0.75-1	15; 16[8]	7-24	[11], [47], [58], [810]
Cyclobarbital	2-6	10	20; 68[8]; 70[8]	8-17	[811, 812]
Cyclobenzaprine	appr. 0.003-0.04	0.4	0.8; 1[8]	18 (9-40)[253]	[11], [58], [257], [992]
Cyclohexane	-0.4[450]			1-3	[47]
1-Cyclohexyl-4-(1,2-diphenylethyl)piperazine (MT-45)	-		0.52[8, 559]; 2.9[8,451]		[993], [1070]
Cyclophosphamide	10-25			(1.3-) 4-8 (-16)	[3], [8]

Note on liability: The data and information have been extracted from scientific sources (primary and secondary literature) and have been carefully reviewed. Nevertheless, transcription errors cannot be excluded. No responsibility is taken for the correctness of the information or data included. As a matter of principle, this compilation should be used with expert knowledge.

Substance	Blood-plasma concentration (mg/L) therapeutic (“normal”)	toxic (from)	comatose-fatal (from)	t½ (h)	References
Cyclopropane	80-180				[11], [58]
Cyclopropylfentanyl[411]	-		1.4-43[412]; 51[8]; 76[8] ng/mL		[813-816], [1015], [1071]
Cyclosporine	see Ciclosporine				
Cyproheptadine	appr. -0.05	0.13[8]	0.47[8]	8-9; 20-40	[3], [47]
Cyproterone acetate	0.01-0.2			30-40 (-120)	[47], [126]
Cysteamine	appr. > 1.55[282]			appr. 1	[3], [258]
Cytarabine	0.05-0.5			0.1-0.2 (1.9-2.5)	[9], [209]
2,4-D	see 2,4-Dichloro-phenoxyacetic acid				
Dabigatran	(0.03-) 0.04-0.2 (-0.3)	0.2?; 2.7[8]	5.6[8]	9-14[83]	[794], [796, 797], [817-820], [960]
Dalfampridine	0.015-0.055			5.8	[780]
Danazol	appr. -0.2			4.5	[3]
Dantrolene	(0.1-) 0.4-1.5 (-3)			4-12	[3], [9], [259]
Dapagliflozin	0.006-0.285			12.9	[780]
Dapsone[48]	0.5-2	10	18[8]	25-31	[260, 261]
Daptomycin	> 3.2 (trough)	24.3 (trough)		(4-) 7-9 (-12)	[821-825], [1186]

Note on liability: The data and information have been extracted from scientific sources (primary and secondary literature) and have been carefully reviewed. Nevertheless, transcription errors cannot be excluded.
No responsibility is taken for the correctness of the information or data included. As a matter of principle, this compilation should be used with expert knowledge.

Substance	Blood-plasma concentration (mg/L)			t½ (h)	References
	therapeutic ("normal")	toxic (from)	comatose-fatal (from)		
Darifenacin	0.0015-0.02			16	[780]
Darunavir (DRV)	> 3.3 (1.3-7.4)[301]			appr. 15	[2], [110], [262]
DEET	see N,N-Diethyl-3-methylbenzamide				
Deferoxamine (Desferrioxamine)	3-15			4-6	[8], [47]
Delorazepam[557]	0.01-0.07			80-115	[47]
Demoxepam	0.5-0.74	1	2.7		[66], [1194]
Desipramine[48,69]	0.01-0.5 (0.1-0.3)	0.3[311]; 0.5-1	3	15-25[70]	[4], [74], [78-80], [263, 264]
Desloratadine[372]	0.002-0.006[373]	0.012[311]		17-27	[4]
Desmethyldiazepam (N-Desmethyldiazepam)	see Nordazepam (= Nordiazepam)				[59]
Des(methyl)venlafaxine	0.1-0.4	0.8[311]		10-17	[4]
Detajmium	0.01-0.7		1.8[8]	13-14	[8]
Dexamethasone	appr. 0.05-0.27[247]	0.8		2.5-9.5	[3], [265]
Dexfenfluramine[351]	appr. 0.03-0.06	0.15-0.25		appr. 18	[11], [58], [66]
Dexketoprofen[374]	appr. 3.7			0.5-2	[47]
Dexmedetomidine	-appr. 3.5 ng/mL[469]			3-10	[47], [1017]

Note on liability: The data and information have been extracted from scientific sources (primary and secondary literature) and have been carefully reviewed. Nevertheless, transcription errors cannot be excluded. No responsibility is taken for the correctness of the information or data included. As a matter of principle, this compilation should be used with expert knowledge.

Substance	Blood-plasma concentration (mg/L) therapeutic (“normal”)	toxic (from)	comatose-fatal (from)	t½ (h)	References
Dexmethylphenidate[342]	0.013-0.023[318]	0.044[311, 318]		appr. 2	[4]
Dextromethorphan[48]	0.001-0.04 (0.23[458])	0.1-2.8	1.1-3 (-20)	2-4 (23-42[458])	[47], [266-270]
Dextromoramide[350]	0.05-0.15	0.2[339]	0.1-1.5	1.5-4-7	[8], [47], [58], [271, 272]
Dextropropoxyphene[305]	0.05-0.3 (-0.5)	0.6-1	1-2	10-30	[30], [48]
Diacetylmorphine or Diamorphine (DAM)	see Heroin (and Morphine)			2-5 minutes	[273-281]
3,4-Diaminopyridin (DAP)	< 0.04[213]	0.1 (?)		0.3-2[214]	[282]
Diazepam[19]	0.1-2 (-2.5)[459]	3[311]-5[459]		24-48	[4], [13, 14], [59], [224], [241], [283-286]
Diazinon	-	0.05-0.1 (-0.5)	0.7-277		[47], [58]
Diazoxide	10-20 (-50)	50 (-100)		20-36 (-48)	[3], [10], [42], [58], [84]
Dibenzepine	0.025-0.15 (0.1-0.5)	3[359]	18[359]	3.5-5	[11], [58]
Dichloromethane	-		50-280 (-2200)	0.6 (early); 4-8 (late phase)	[47]
2,4-Dichlorophenoxyacetic acid (2,4-D)	-	appr. 100	200; 392[8]; 720[8]	4-140[182]	[3], [11], [47], [58]
Dichlorprop	-	52[8]	250[8]; 450[8]	70-90	[47]
Dichlorvos (2,2-Dichlorvinyl-dimethyl-phosphate, DDVP)	-		29	0.16	[47]
Diciclomine	see Dicyclomine				[8]

Note on liability: The data and information have been extracted from scientific sources (primary and secondary literature) and have been carefully reviewed. Nevertheless, transcription errors cannot be excluded.
No responsibility is taken for the correctness of the information or data included. As a matter of principle, this compilation should be used with expert knowledge.

Substance	Blood-plasma concentration (mg/L) therapeutic ("normal")	toxic (from)	comatose-fatal (from)	t½ (h)	References
Diclazepam[421]	-[468]				[1016]
Diclofenac	0.5-3	50; 60[8]		1-3	[47], [287-289]
Dicoumarol	8-30	22-192		1-4 days	[8], [11]
Dicyclomine (Dicycloverin)	-0.1	appr. 0.2	0.2[8]; 0.5[8]	1.8-2 (-5)	[8], [47]
Didanosine (DDI)	appr. 1-30 µmol/L			appr. 1.4	[2, 3], [38], [290, 291]
Dieldrin	-0.0015	0.15-0.3	0.5	2-12 months	[11], [47], [58]
Diethylcarbamazine	> 0.8-1.0			4-15	[292]
Diethylene glycol	-	0.04[8, 453]; 17[8, 454]; 200-500	0.2[558]-360[455]	3-4	[47]
N,N-Diethyl-3-methylbenzamide (N,N-Diethyl-m-toluamide; DEET)	-	1.6	112[8]; 240[8]	appr. 2.5	[47]
Diethylpentenamide (Valdetamide)	2-10	20	45	6-7	[8], [13]
Diethylpropion	0.003-0.007 (-0.2)	2	5.4[8]	4-8[23]	[3], [8], [58]
Difenacoum	-	0.5		11-42 days	[47]
Diflunisal	40-100 (-200)	300	260[8]; 370[8]; 520[8]	5-12[83]	[3], [9], [47], [58], [293]
Digitoxin	0.008-0.018	0.03	0.04	140-200 (7-9 days)	[294, 295], [783]
Digoxin	0.0005-0.0009	(0.0012-) 0.002[383]	0.004-0.005	40-70	[18-27], [782-786]

Note on liability: The data and information have been extracted from scientific sources (primary and secondary literature) and have been carefully reviewed. Nevertheless, transcription errors cannot be excluded.
No responsibility is taken for the correctness of the information or data included. As a matter of principle, this compilation should be used with expert knowledge.

Substance	Blood-plasma concentration (mg/L)			t½ (h)	References
	therapeutic ("normal")	toxic (from)	comatose-fatal (from)		
Dihydralazine	see Hydralazine				
Dihydrocodeine	0.03-0.25	0.5-1 (-12)[339]	0.4-166	3-4	[14], [47], [58]
Dihydroergotamine	0.001-0.01			4-15	[3], [8], [47]
Diltiazem	0.03-0.13 (-0.25)[157]	0.8-1	2-6; 7[8]; 8[8]	2-6 (4-9)	[3], [47], [58], [84]
Dimenhydrinate	see Diphenhydramine				
Dimethadione[360]	(350-) 700-1000	1000		5-10 days	[3], [8], [11, 12]
Dimethindene	0.01-0.05			appr. 6	[3], [8]
Dimethoate	-		355.5 (160.0-674.0) μmol/L[277]	appr. 5	[47], [186]
2,5-Dimethoxy-4-bromoamphetamine (DOB)			0.01[8]; 0.02[8]; 0.9[8]	17-22	[47]
2,5-Dimethoxy-4-bromophenethylamine (2C-B)		0.34[8]		1.1	[47], [994]
3,4-Dimethylmethcathinone (3,4-DMMC)				27[8]	[1018]
N,N-Dimethyltryptamine	0.001-0.1			0.5-1.5	[47], [58]
4,6-Dinitro-2-methylphenol (Dinitro-O (ortho)-cresol [DNOC])	1-5	30-60	34[8]; 75[8]	5-6 days	[47], [58]
Dionin	see Ethylmorphine				

Note on liability: The data and information have been extracted from scientific sources (primary and secondary literature) and have been carefully reviewed. Nevertheless, transcription errors cannot be excluded. No responsibility is taken for the correctness of the information or data included. As a matter of principle, this compilation should be used with expert knowledge.

Substance	Blood-plasma concentration (mg/L) therapeutic (“normal”)	toxic (from)	comatose-fatal (from)	t½ (h)	References
Diphenhydramine	0.05-0.1 (-1)	(0.1-) 1-2 (-4)	1.1 (in infants); 5-10	3-14, 20-60	[47], [58], [114], [296, 297]
Diphenidine			0.012-1.38[470]		[1015]
Diphenoxylate	appr. 0.01-0.08		0.34[8]	2-3	[47]
Dipipanone	appr. -0.05	0.43	< 0.1 (oral); 0.5-6.2 (i.v.)	3.2-3.8	[47]
Diprophylline	see Dyphylline				
Dipyridamole	0.1-1.5 (-2)	4	9.2[8]; 187[8]	(6-) 11-13 (-28)	[9], [13, 14], [47]
Dipyrone	see Metamizole				
Diquat	-	0.1-0.4	0.4-4.5; 65[8]		[11], [47]
Disopyramide	2-7[78]	8	27	(3-) 5-8 (-11)	[11], [47], [58], [64], [70], [986]
Disulfiram	0.05-0.4	0.5[311]; 5	8[456]; 120[456]	5-9	[4], [47], [58]
Divalproex	see Valproic acid			(9-) 12.5 (-16)	[780]
Dixyrazine	appr. 0.3[249]		5.5[8]; 9.4[8]		[4], [298]
DOB	see 2,5-Dimethoxy-4-bromoamphetamine				
Dofetilide	0.002-0.0055			(5-) 9-10 (-13.5)	[780]
Domperidone	appr. 0.01-0.1	0.2[311]		12-16	[3], [299-302]

Note on liability: The data and information have been extracted from scientific sources (primary and secondary literature) and have been carefully reviewed. Nevertheless, transcription errors cannot be excluded.
No responsibility is taken for the correctness of the information or data included. As a matter of principle, this compilation should be used with expert knowledge.

Substance	Blood-plasma concentration (mg/L) therapeutic ("normal")	toxic (from)	comatose-fatal (from)	t½ (h)	References
Donepezil[207]	appr. 0.03-0.075	0.1[311]		70-100	[4], [303, 304]
Doripenem	appr. -12			(1-) 2.5-8	[826], [1186]
Dothiepin (Dosulepin)[20]	0.045-0.1	0.2[311]; (0.3-) 0.8	0.3-5.8	(8-) 11-20 (-40)	[4], [47], [305, 306]
Doxacurium	0.01-0.3			1-2[83]	[8]
Doxapram	(1.5-) 2-5	9[268]		2.4-9.9	[3], [11], [58], [307]
Doxazosin	0.01-0.15	0.3[311]		10-22	[47], [95]
Doxepin[21]<br>Doxepin plus N-desmethyldoxepin	0.01-0.2 (0.03-0.1)<br>0.05-0.15	0.5-1<br>0.3[311]	2-4	(8-) 15-20 (-25)	[4], [73], [77], [80], [264], [308-310]
Doxorubicin (Adriamycin)	0.006-0.02			20-48	[3], [8], [58]
Doxycycline	1-5 (-10)	30		15-28	[8], [58]
Doxylamine[289]	0.05-0.2[457]	0.32[311]; 1-2	5	(7-) 9-11 (-13)	[4], [47], [87], [311], [827]
Dronabinol (Delta-9-tetrahydrocannabinol, THC)	0.005-0.01 (-0.05)[137]			50-100	[241], [312, 313]
Dronedarone	0.084-0.167[398]			24-30	[780], [828]
Droperidol	appr. -0.05			1.5-2.5[237]	[3], [314]
Drotrecogin alfa	mean 0.072			1.6	[315]
Duloxetine	0.03-0.12	0.24[311]		9-19	[4], [223]

Note on liability: The data and information have been extracted from scientific sources (primary and secondary literature) and have been carefully reviewed. Nevertheless, transcription errors cannot be excluded.
No responsibility is taken for the correctness of the information or data included. As a matter of principle, this compilation should be used with expert knowledge.

Substance	Blood-plasma concentration (mg/L) therapeutic (“normal”)	toxic (from)	comatose-fatal (from)	t½ (h)	References
Dutasteride	0.025-0.055			4-5 weeks	[780]
Dyphylline	6.5-14 (-20)	40		2	[11], [58]
Ecamsule (Mexoryl SX)	0.0005-0.012[419]				[801]
Ecdysterone	appr. -0.008				[829], [1066]
Edoxaban	0.02-0.1	0.15 (-0.2)[402]		(6-) 10-14	[47], [794], [797], [830], [960]
Edrophonium	0.15-0.2	appr. 0.15		1.3-2.4	[3], [58], [316]
Efavirenz (EFV)	> 1.0 appr. -3[297]	6[311]		40-55	[2], [110, 111], [780]
Eletriptan	0.06-0.23			3-7	[47]
Embutramide			2-90	9.6	[47]
Emetine	(0-)[472] 0.005-0.075	0.008-0.5	2.4[8]	10-18; 24-48	[8], [47]
Empagliflozin	0.03-0.36			9-10	[780]
Emtricitabine				appr. 10	[780]
Enalapril[52]	0.01-0.05 (-0.1)	0.3	2.8[8]	(6-) 8-11 (-55)	[47], [84], [159]
Encainide[48]	-[175]			1.5-3.5[176]	[3]
Endrin	-0.003	0.01-0.03	0.62[8]		[47], [58]
Enflurane	44-144[473]		130[8]; 710[8, 474]	36[560]	[47]

Note on liability: The data and information have been extracted from scientific sources (primary and secondary literature) and have been carefully reviewed. Nevertheless, transcription errors cannot be excluded. No responsibility is taken for the correctness of the information or data included. As a matter of principle, this compilation should be used with expert knowledge.

Substance	Blood-plasma concentration (mg/L) therapeutic ("normal")	toxic (from)	comatose-fatal (from)	t½ (h)	References
Enfuvirtide	2.6-3.4			3-4	[47], [1001]
Enoxacin	1-4[475]			3-6	[47], [197], [1019]
Enoximone	≥ 0.2 (3-4 ?)			4-7	[3], [8], [95]
Enprofylline	1-5	10		appr. 2	[8]
Entacapone	0.4-1.0 (-7.0)	2[311]		(0.5-) 1.5-3.5	[3, 4], [8]
Enzalutamide[568]	appr. 19-25 (-30)[391]			appr. 6 (3-10) days	[831, 832], [1086]
Eperisone	appr. 0.0007-0.008[476]	0.16-4.1	15.3[8]	1-4	[47]
Ephedrine	0.02-0.2	0.15[8]; 1; 15[8]; 22[8]; 23[8]	2.7[8]; 5[8]; 3.5-21[471]	3-11	[8], [47], [57, 58], [87], [269]
Epinephrine (Adrenaline)	appr. 6.8 ng/L[478]		60 ng/L[8, 561]	0.7[477]	[47], [1073]
Epirubicin	appr. (0.05-) 0.2-3			10-45	[47], [1000]
Eplerenone	appr. 1-1.7[479, 620]			3-6	[47]
Eprosartan	0.4-1.0 (-1.85)			5-9	[3], [8]
Eptastigmine	0.0002-0.006			appr. 1	[8]
Eptinezumab				26-28 days	[1102]
Erenumab	appr. 10-25 ?			21-28 days	[1102], [1105, 1106]
Ergotamine	0.36-0.42 ng/mL[375]	0.82 ng/mL[376]		1.5-2.5	[47]

Note on liability: The data and information have been extracted from scientific sources (primary and secondary literature) and have been carefully reviewed. Nevertheless, transcription errors cannot be excluded.
No responsibility is taken for the correctness of the information or data included. As a matter of principle, this compilation should be used with expert knowledge.

Substance	Blood-plasma concentration (mg/L)			t½ (h)	References
	therapeutic ("normal")	toxic (from)	comatose-fatal (from)		
Ertapenem	appr. -100 (total[410])			3.5-7[409]	[825], [833, 834], [1186]
Erythromycin	0.5-6 (peak 4-12)	12-15		1-3	[11], [58]
Escitalopram[343]	0.015-0.08	0.16[311]		26.3 ± 10.8 (27-32)	[4], [223]
Eslicarbazepine acetate[480]	10-35	70[311]		20-40	[4]
Esmolol	0.15-2			4-16 minutes	[7]
Esomeprazol	appr. 0.6-6.6			0.6-1.4	[47]
Estazolam	0.055-0.2		0.48[8]; 1.25[8]	10-24	[3], [47], [58]
Eszopiclone[341]	appr. 0.01-0.09	0.15		4-9	[47], [780], [935]
Etacrynic acid (Ethacrynic acid)	0.05-0.1			1-4	[3], [8, 9]
Etamsylate (Ethamsylate)	15-20			2.5-4	[13, 14], [58]
Ethadione	500-1000	1000			[8]
Ethambutol	(0.5-) 3-5 (-6.5)	6-10	84[8]	2.5-3.5; 6-15	[47], [58], [317]
Ethanol		1000-2000	3500-4000	-[139]	[13]
Ethchlorvynol	0.5-8	20	50	10-25 (-35)	[3], [58]
Ethinamate	1.5-10	50-100	200[8]	appr. 2	[8], [13]
Ethosuximide	30-100 (40-60)	120[311]; 150-200	250	30-60	[4], [12], [64], [161], [164]

Note on liability: The data and information have been extracted from scientific sources (primary and secondary literature) and have been carefully reviewed. Nevertheless, transcription errors cannot be excluded.
No responsibility is taken for the correctness of the information or data included. As a matter of principle, this compilation should be used with expert knowledge.

Substance	Blood-plasma concentration (mg/L)			t½ (h)	References
	therapeutic ("normal")	toxic (from)	comatose-fatal (from)		
Ethyl chloride	-		36[8]; 200/650[481]		[47]
Ethylene glycol		200-500	2000 (500-7750)	3-4 (rats); 2-3[482]; 14.2-19.7[483] (31[8, 278])	[47], [66], [318-326], [1020, 1021]
Ethylmorphine[48] (Dionin)	0.3-0.6[339, 377]		0.05[8]; 0.3-2.9[339]	2-3	[47]
Ethylone (3,4-Methylenedioxy-N-ethylcathinone, = MDED, bk-MDEA)				1.7[8]; 0.038-2.57[484]	[1022]
Ethylphenidate				0.026-2.18[485]	[1023]
Etidocaine	0.5-1.5	1.6-2		2-3	[327]
Etilefrine	appr. 0.06			2-3.5	[1002], [1053-1054]
Etizolam[48]	0.008-0.018[421, 486]	0.03[8]	0.26[8]	2-3	[47], [1024-1028]
Etodolac	10-20 (> 14[229])	40		6-8	[3], [8], [328]
Etomidate	0.1-0.5 (-1)			3.9 ± 1.1 (2-11)	[3], [9]
Etoposide	2-6 (peak 8-14)			4-11	[3], [11]
Etoricoxib	appr. 1.3-3.6			20-36	[47]
Etravirine (ETR)	0.275 (0.081-2.98)[302]			appr. 41	[2], [47], [110]
Everolimus	0.003-0.008 (-0.014)[275, 487]			28 ± 7	[329-332], [1029]
Ezetimibe	0.005-0.045	0.09[311]		appr. 30	[333, 334], [780]

Note on liability: The data and information have been extracted from scientific sources (primary and secondary literature) and have been carefully reviewed. Nevertheless, transcription errors cannot be excluded.
No responsibility is taken for the correctness of the information or data included. As a matter of principle, this compilation should be used with expert knowledge.

Substance	Blood-plasma concentration (mg/L) therapeutic ("normal")	toxic (from)	comatose-fatal (from)	t½ (h)	References
Famciclovir	see Penciclovir				[47]
Famotidine	0.02-0.2	0.42[8]		2-4.5	[3], [335, 336]
Fampridine	see 4-Aminopyridine				
Favipiravir				4.6 ± 1.2	[1229]
Febuxostat	0.035-1.45			6-7	[780]
Felbamate	(30-) 50-110[164]	100[311]; 150-200		15-23	[4], [12], [337-339]
Felbinac	appr. 0.4-1[326]			10-17	[3], [340]
Felodipine	0.001-0.012	0.01		22-27[88]	[8], [84], [341]
Fenbufen	appr. -60			10-12	[3], [58]
Fendiline	0.02-0.15			appr. 20	[58]
Fenetylline (Fenethylline)[490]				appr. 1.3	[47]
Fenfluramine	0.04-0.3	0.5 – 0.7	6	18-25	[8], [58]
Fenitrothion			1.1-17[491]	33-64	[47]
Fenofibrate[489]	5-30[241]	60[311]		20-22	[3], [342], [780]
Fenoldopam	0.003-0.06			0.1	[8], [58]
Fenoprofen	(25-) 30-60	120[311]		2-3	[343], [780]

Note on liability: The data and information have been extracted from scientific sources (primary and secondary literature) and have been carefully reviewed. Nevertheless, transcription errors cannot be excluded. No responsibility is taken for the correctness of the information or data included. As a matter of principle, this compilation should be used with expert knowledge.

Substance	Blood-plasma concentration (mg/L) therapeutic ("normal")	toxic (from)	comatose-fatal (from)	t½ (h)	References
Fenoterol	(0.001-) 0.01-0.04			appr. 7	[929, 930]
Fentanyl	0.0003[4]-0.3[339]	-[339]	0.003-0.02[8,103]	1-3.5 (transdermal patch: appr. 17)	[47], [52], [54], [335], [344-351], [1071], [1078-1080]
Fenthion			1.4 (0.2-4.6)[279]	12	[47], [186]
Fesoterodine[492]	0.005[493]			5-9[494]	[47], [1030]
Fexofenadine	appr. 0.1-0.9[191]	2.7		14-18	[8], [352], [780]
Finasteride	0.008-0.01			5-7	[353]
Fingolimod	appr. 0.001-0.018[495]			6-9 days	[47]
Flecainide[48]	(0.2-) 0.4-0.8	1[311]; 1-2	2.6[8]; 13[8]; 15[8]	10-20	[70], [354], [780]
Flephedrone (4-Fluoromethcathinone, 4-FMC)			0.6[8]; 4.4[8]		[955]
Fleroxacin	appr. 5			(8-) 10-12 (-13)	[1031]
Flibanserin	appr. 0.4[496]			6-14	[1032]
Flocoumafen			0.005-0.273[497]	6-7 days[498]	[47]
Flubromazepam	-[421]	0.05-0.08; 0.412[8]	0.83[8, 401]	appr. 100	[835], [837-839]
Flubromazolam	appr. 0.008[421, 499]		0.059[8, 500]	10-20[501]	[836], [1031], [1033]
Flucloxacillin	3-30			1-2	[11], [58]

Note on liability: The data and information have been extracted from scientific sources (primary and secondary literature) and have been carefully reviewed. Nevertheless, transcription errors cannot be excluded. No responsibility is taken for the correctness of the information or data included. As a matter of principle, this compilation should be used with expert knowledge.

Substance	Blood-plasma concentration (mg/L) therapeutic (“normal”)	toxic (from)	comatose-fatal (from)	t½ (h)	References
Fluconazole	appr. 2-6 (-15)	20; 95[8]		22-34[83]	[11], [91], [355-358], [1186]
Flucytosine	(20-) 25-50 (-70)	100		3-5	[8], [58]
Flumazenil[22]	(0.01-) 0.02-0.1	0.5		1-2	[931-934]
Flunarizine	0.025-0.2	0.3		days	[42]
Flunitrazepam[23]	0.005-0.015	0.05	0.3 (0.11-0.74)[502]	10-20 (-30)	[56], [59], [359], [1012], [1034]
Fluoride	0.095-0.190 (-0.285)[39]	0.5-2	3	2-9	[10], [47], [58], [130], [360-362]
5-Fluoro ADB (Methyl 2-[1-(5-fluoropentyl)-1H-indazole-3-carboxamide]-3,3-dimethylbutanoate = 5-Fluoro MDMB-PINACA)	-		0.11-1.92 ng/mL		[1035, 1036]
5F-AMB (5F-AMB-PINACA)	see AMB				
4-Fluoroamphetamine	-	0.06-0.43[503]	0.32[8, 504]		[47]
Fluorofentanyl	-		2.4 ng/mL[8]; 30 ng/mL		[1038], [1071, 1072]
2-Fluoromethamphetamine (2-FMA)	-		6.9 ng/mL[8, 506]		[1041]
4-Fluoromethlyphenidate	-	0.032[8, 417]			[840]
5-Fluorouracil	0.05-0.3	0.4-0.6		< 0.5	[3], [11], [58]
Fluoxetine	0.12-0.5[130]	1[311]	2.2; 3.8[507]; 6[8]	2-6 days[130]	[4], [47], [75], [202], [263, 264], [363, 364, [1084]]

Note on liability: The data and information have been extracted from scientific sources (primary and secondary literature) and have been carefully reviewed. Nevertheless, transcription errors cannot be excluded. No responsibility is taken for the correctness of the information or data included. As a matter of principle, this compilation should be used with expert knowledge.

Substance	Blood-plasma concentration (mg/L)			t½ (h)	References
	therapeutic (“normal”)	toxic (from)	comatose-fatal (from)		
Flupentixol (Flupenthixol)	0.0005-0.005	0.015[311]		20-40	[4]
Fluphenazine	0.001-0.01	0.015[311]		10-18[45]	[4]
Flupirtine	0.5-1.5	appr. 3-4	11[8]	7-11	[365], [1042]
Flurazepam[24]	0.075-0.165[508]	0.33[311]; 0.2-0.5	0.5-2.8[509]; 24[8]	appr. 2-3[24]	[4], [47]
Flurbiprofen	5-15			3-4	[366]
Fluspirilen	0.0001-0.0022	0.0044[311]		7-14 days	[4]
Flutamide[60]	0.4-1.5[60]			7-20[60]	[43], [367]
Fluvastatin	0.05-0.44		2.4[8]	1-3	[47], [1063]
Fluvoxamine	0.06-0.23	0.5[311]-0.65; 1.97[8]	2.8[8]; 5.4[8]	(8-) 15-22 (-28)	[4], [47], [202], [368, 369], [1084]
Folic acid	appr. 0.004-0.03[389]			1.5-2	[800], [944]
Fomepizole	8-35[510]			10-40 (dose-dependent)	[47], [1055-1058]
Formoterol	appr. 20-80 ng/L			5-13	[47]
Fosamprenavir (FPV)[513]	> 0.4[292]			4-11; 7.7[319]	[2], [47], [110, 111]
Fosinopril	appr. 0.13-0.25[511]; appr. 0.71-1.2[514]			6-17[512]	[47]
Fosphenytoin[336]	see also Phenytoin	30		0.13-0.25	[12]

Note on liability: The data and information have been extracted from scientific sources (primary and secondary literature) and have been carefully reviewed. Nevertheless, transcription errors cannot be excluded. No responsibility is taken for the correctness of the information or data included. As a matter of principle, this compilation should be used with expert knowledge.

Substance	Blood-plasma concentration (mg/L) therapeutic ("normal")	toxic (from)	comatose-fatal (from)	t½ (h)	References
5F-Cumyl-PEGACLONE	see Cumyl				
5F-PB-22	-		0.37-1.5 ng/mL[505]		[1039, 1040], [1225]
Fospropofol[515]				0.7-0.9	[47]
Fremanezumab				30-32 days	[1102], [1104]
Frovatriptan	appr. 0.004-0.008			20-30	[47]
Furanyl fentanyl	-		2.7 (0.4-42.9) ng/mL[516]		[1011], [1071]
Furosemide (Frusemide)	2-5 (-10)	25-30		0.7-1.5 (-3)	[3], [47], [58]
Fusidic acid	30-200			4-6 (9-13)	[8], [1003]
Gabapentin	appr. 0.5-6 (-20-30)[185]	25[311]; 45[8]; 85[8]; 105[8]; 127[8]	37[8]	5-7 (-9)	[4], [12], [47, 48], [370-382], [804], [841, 842]
Galantamine (Galanthamine)	(0.01-) 0.03-0.06	0.09[311]		6-10	[4], [383, 384]
Galcanezumab	appr. 10-30 ?			27 days	[1102, 1103]
Gallopamil	0.02-0.1		8[8]	3-8	[84], [385]
Gamma-butyrolactone[517]	see 4-Hydroxybutyrate				[1043]
Gamma-hydroxybutyric acid (gamma-hydroxybutyrate, GHB, liquid ecstasy)	see 4-Hydroxybutyrate				[386, 387], [843-847], [1043]
Ganciclovir	(0.29-0.51) 0.5-5[107]	3-5		2-4[83]	[38, 39], [388]

Note on liability: The data and information have been extracted from scientific sources (primary and secondary literature) and have been carefully reviewed. Nevertheless, transcription errors cannot be excluded. No responsibility is taken for the correctness of the information or data included. As a matter of principle, this compilation should be used with expert knowledge.

Substance	Blood-plasma concentration (mg/L)			t½ (h)	References
	therapeutic ("normal")	toxic (from)	comatose-fatal (from)		
Gemcitabine	3.9-5.3[146]	-[192]		0.05 (0.18-0.43)	[209]
Gemfibrozil	appr. -25			1.5	[3]
Gentamicin	(2-) 4-10[233]	12		1.5-6	[20], [67], [389-394]
GHB (gamma-hydroxybutyric acid)	see 4-Hydroxybutyrate				
Glibenclamide (Glyburide)	0.05-0.2	0.6		10	[47], [780]
Gliclazide	1-3.7			6-14	[47]
Glimepiride	0.09-0.5	1[311]		4-15	[47], [780]
Glipizide	0.1-1 (-1.5)	2		3-7	[66], [780]
Glutethimide	0.2-5	(5-) 10-30	15-50 (10-97[518])	5-20	[30], [47]
Glyburide	see Glibenclamide				
Glycerol	appr. 8 (2.9-17)[522]		3643[8]	0.6-1.1 (low concentrations); 2.5-10[523]	[47], [1044], [1074]
Glyceryl trinitrate (GTN)	see Nitroglycerin				
Glyphosate			3.7-1980[524]; 118-7480[525]	2-4	[47], [1059, 1060]
Gold	3-8[519]	(5-) 10-15	0.4[8]	21-31 days (oral)	[47, 48], [58], [1045]
Granisetron	appr. 0.005-0.017			3-21[520]	[3], [8], [47]

Note on liability: The data and information have been extracted from scientific sources (primary and secondary literature) and have been carefully reviewed. Nevertheless, transcription errors cannot be excluded.
No responsibility is taken for the correctness of the information or data included. As a matter of principle, this compilation should be used with expert knowledge.

Substance	Blood-plasma concentration (mg/L) therapeutic ("normal")	toxic (from)	comatose-fatal (from)	t½ (h)	References
Griseofulvin	0.3-1.7			22 (9-33)	[8], [47]
Guaifenesin	0.3-1.4		25[8]	appr. 1	[3], [8], [47]
Guanethidine	0.01			5-10 days	[3], [58]
Guanfacine	appr. 0.001-0.01			13.5 (10-30)	[780]
Halazepam[15]	see Nordazepam			30-40	[13, 14]
Haloperidol	(0.001-) 0.005-0.017	0.006/0.023[521]; 0.015[311]; 0.05	0.18[8, 74]; 0.5	10-35[153]	[4], [43], [47], [185], [193], [240], [395-398]
Halothan	22-260		33-720[526]; 3.4/8.3[8, 527]	43	[48], [87]
Hematin	50-100				[13, 14]
Hemin	see Hematin				
Heptabarb (Heptabarbital)	0.5-4	8-15	20	6-11	[13, 14]
Heptaminol	appr. 0.2-1 (-1.5)			2-3	[13, 14]
Heroin (Diacetylmorphine, Diamorphine)[337]	-[338, 528]	-[339]		2-5 minutes	[273-281], [1046]
Hexachlorobenzene (HCB)	-0.0001[332]			appr. 2 years	[100]
ß-Hexachlorocyclohexane (ß-HCH, ß-Lindane)	-0.0001[332]			appr. 7 years	[100]
Hexachlorophene	0.003-0.65 (-1)		35	6-44	[11], [58]

Note on liability: The data and information have been extracted from scientific sources (primary and secondary literature) and have been carefully reviewed. Nevertheless, transcription errors cannot be excluded. No responsibility is taken for the correctness of the information or data included. As a matter of principle, this compilation should be used with expert knowledge.

Substance	Blood-plasma concentration (mg/L)			t½ (h)	References
	therapeutic ("normal")	toxic (from)	comatose-fatal (from)		
n-Hexane	< 0.09 ng/mL[463]			1.5-2	[47]
Hexapropymate	2-5	10-20			[8], [11]
Hexobarbital	1-5	10-20	50	4-6	[13, 14], [58]
Hirudin-rec	-[171]	-[171]		(1-) 2.5-3	[3], [43], [399]
Homosalate	0.004-0.023[583]			(18-) 47-78 (-162)	[1122]
Hydralazine[5]	0.05-0.5 (-1.5)			2-6	[84]
Hydrochlorothiazide	appr. 0.04-0.45 (-2)	4[311]		(6-) 9-12 (-15)	[3], [42], [779, 780], [1150, 1151]
Hydrocodone	0.01-0.04 (0.05)	0.1[311, 339]	0.13-7	appr. 4	[8], [47], [244], [780]
Hydromorphone	appr. 0.005-0.015 (-0.03)	0.1[311, 339]	0.03[8]; 0.06[8]; 0.2	2-3	[8, 9], [270], [400], [780], [1047], [1075]
4-Hydroxybutyrate (gamma-hydroxybutyric acid; GHB)[48]	appr. 50-120[393]	80 (abuse); 200[311]	30-9200 (mean 640; median 280; intoxication)	(0.3-) 0.5-1	[3, 4], [8], [47], [244], [401, 402], [843-847], [1043]
Hydroxychloroquine[384]	0.1-0.5[385]	0.5-0.8	4; 48[8]; 104[8]	dose-dependent days[386]	[3], [13, 14], [47], [403], [780], [848-852], [1226]
4-Hydroxy-3-methoxymethamphetamine (HMMA)	-[333]			11.5-13.5	[404-406]
Hydroxytryptophan (5-HTP)	appr. 0.5-1.5 (-9.4[529])			2-7[530]	[47]

Note on liability: The data and information have been extracted from scientific sources (primary and secondary literature) and have been carefully reviewed. Nevertheless, transcription errors cannot be excluded. No responsibility is taken for the correctness of the information or data included. As a matter of principle, this compilation should be used with expert knowledge.

Substance	Blood-plasma concentration (mg/L) therapeutic ("normal")	toxic (from)	comatose-fatal (from)	t½ (h)	References
Hydroxyzine	0.05-0.1	0.1	0.7[8]; 2.5[8]; 3[8]; 39[8]	7-20	[11], [47], [56], [58]
Hyoscyamine	0.0004-0.006			7.5	[780]
Ibandronat (Ibandronic acid)	< 0.1[321]			10-60; terminal: days	[47], [49], [1004, 1005]
Ibuprofen	15-30 (-50)	200	185[8]; 260[422]; 352[346]; 1233[531]	0.9-2.5 (-3)	[47], [293], [343], [407, 408], [926]
Idebenone	0.05-0.2[532]			(8-) 16-22 (-26)	[3], [47], [409], [1048]
Iloperidone	0.005-0.01	0.02[311]; 0.07[533]		18-33	[4], [1049]
Iloprost	appr. 0.0001			appr. 0.5	[936, 937]
Imatinib	0.72[258]			appr. 18	[410, 411]
Imipenem	0.5-5 (20-75)			1-1.5	[8], [412], [825], [833], [1186]
Imipramine[48, 125]<br>plus Desipramine	0.05-0.35<br>0.175-0.3	0.5-1<br>0.3[311]	1.5-2	(6-) 11-25[26, 125]	[4], [74], [76-80], [308, 309], [413], [1084]
Inamrinone	-[534]		76[8]	4-6	[47]
Indapamide	0.13-0.25			14-15	[780]
Indinavir (IDV)	> 0.1[260]	appr. 0.5		1.5-2	[2, 3], [110, 111], [211], [414]
Indometacin (Indomethacin)	0.3-1 (-3)	4-5		5-10 (adults); 10-33 (neonates)	[47], [415], [780]
Indoramin	appr. 0.025-0.1			12 (3.5-15)	[95]

Note on liability: The data and information have been extracted from scientific sources (primary and secondary literature) and have been carefully reviewed. Nevertheless, transcription errors cannot be excluded. No responsibility is taken for the correctness of the information or data included. As a matter of principle, this compilation should be used with expert knowledge.

Substance	Blood-plasma concentration (mg/L) therapeutic ("normal")	toxic (from)	comatose-fatal (from)	t½ (h)	References
Infliximab	3-7[564]			6-12 days	[47], [1077]
25I-NBOMe	see NBOMe				
INH (isonicotinic acid hydrazide)	see Isoniazid				
Iproniazid	appr. -5 ?				[58]
Irbesartan	appr. 1.9-3.3[371]			11-15	[47]
Iridium	-0.02				
Iron	0.5-2	6	17	3-8	[42], [47], [58], [64]
Isavuconazole	appr. 2.5[535]			50-110	[47]
Isoflurane	appr. 20-150		1.8-48[537]	7 min to 58 h[536]	[47]
Isoniazid (INH)[5]	5-10	20	(30-) 100	(0.6-) 1-3 (-6.7)[5]	[3], [8], [317]
Isopropanol[361]		200-400	330[8]; 730[8]; 1000	2.5-3	[47], [58]
Isosorbide-dinitrate (ISDN)[538]	appr. 0.02-0.2[539]			0.4-0.8	[47]
Isosorbide-5-mononitrate (IS-5-MN)	0.1-1	3.1		2-5 (-8)	[47], [95]
Isotretinoin	appr. 0.001-0.002 (topical)[224]			10-20	[3], [416, 417]
Isoxicam	5-15			20-50	[8], [853]
Isradipine	0.0005-0.002 (-0.01)	0.01	0.26[8, 259]	5-10	[3], [84], [418-420]

Note on liability: The data and information have been extracted from scientific sources (primary and secondary literature) and have been carefully reviewed. Nevertheless, transcription errors cannot be excluded. No responsibility is taken for the correctness of the information or data included. As a matter of principle, this compilation should be used with expert knowledge.

Substance	Blood-plasma concentration (mg/L) therapeutic ("normal")	toxic (from)	comatose-fatal (from)	t½ (h)	References
5-IT	see 5-(2-Aminopropyl)indole				
Itraconazole	appr. 0.4-2[111]			24-36	[421-424]
Ivabradine	appr. 0.01-0.06[620]	0.11[8]; 0.37[8]; 0.52[8]		1.3-2.8	[47]
Ivermectin	appr. 0.05[51]		0.09[8]	16-28	[3], [47], [425]
Kanamycin	trough: 1-4; peak: 10-25	25-30		(0.5-)2-3 (-5)	[8]
Kavain	appr. 0.05	0.18-5	1.4[8]	2.8-6.7	[47]
Ketamine	(0.1-) 1-6	0.02[540]; 0.42[562]; 7 (abuse)	3.8[8]; 6.9[8]	1-3 (-4)	[47], [56], [58], [426-428]
Ketanserin	0.05-0.5		2.8[8]	10-22	[8], [47]
Ketazolam[15]	0.001-0.02			1-3	[59]
Ketobemidone	0.01-0.05		0.2-3.2	1.8-4.2	[3], [47], [426], [428]
Ketoconazole	1-3 (-6)			6-10	[8]
Ketoprofen	1-6 (-20)	12[311]	1100[8]	1,1-2 (-4.2)	[47], [429-433], [780]
Ketorolac	0.5-3	5[311]		4-10	[3], [8], [780]
Ketotifen[541]	0.0004-0.004	0.02	1.2[8]?	(4-) 7-21 (-27)	[57], [1061, 1062], [1076]
Kratom	see Mitragynine				

Note on liability: The data and information have been extracted from scientific sources (primary and secondary literature) and have been carefully reviewed. Nevertheless, transcription errors cannot be excluded.
No responsibility is taken for the correctness of the information or data included. As a matter of principle, this compilation should be used with expert knowledge.

Substance	Blood-plasma concentration (mg/L)			t½ (h)	References
	therapeutic (“normal”)	toxic (from)	comatose-fatal (from)		
Labetalol	0.03-0.2 (-0.65)	1[8]; 2.9[8]	1.7[8]	3-10	[3], [7], [47], [95]
Lacidipine	0.001-0.006			12-19	[8], [1087]
Lacosamide	1-10	20[311]		10-15	[4]
Laetrile (Amygdalin)	see Cyanide				
Lamivudine	-[230]			5-7	[2, 3], [434]
Lamotrigine	1-15	20	36[8, 347]; 50[8]	(15-) 23-37[109]	[4], [8], [47], [435-437]
Lansoprazole	0.06-0.4			1-2	[780], [1088]
Lead	-0.09[324]	0.4-0.6	3	-[180]	[47], [64], [100], [438, 439]
Leflunomide[255]	6-100[256]			4-28 days	[3], [440-442]
Lenalidomide	0.06-1	2613 ng/h/mL[392]		3-4	[854-856], [1989, 1090]
Lercanidipine	appr. 0.1-10 ng/mL[620]	0.28[8]		6-10	[47], [1064]
Levacetylmethadol[50] (= Acetylmethadol)	appr. 0.02-0.06			32-116[50]	[8], [47]
Levamisole	appr. 0.1-0.7 (0.7-2.5)[569]			2-8	[8], [47], [1091, 1092]
Levetiracetam	(3-) 10-40	50[311]; 400[8, 264]		4-10	[4], [8], [443]
Levocabastine	< 0.001-0.01[147]			30-40	[444], [1093]
Levocetirizine[378]	0.3-0.5			6-10	[47]

Note on liability: The data and information have been extracted from scientific sources (primary and secondary literature) and have been carefully reviewed. Nevertheless, transcription errors cannot be excluded.
No responsibility is taken for the correctness of the information or data included. As a matter of principle, this compilation should be used with expert knowledge.

Substance	Blood-plasma concentration (mg/L) therapeutic ("normal")	toxic (from)	comatose-fatal (from)	t½ (h)	References
Levodopa (L-Dopa)	0.3-2[28]	5[311]; 20[8]	650[8]	1-3[215]	[4], [445-449]
Levomepromazine[27]	0.005-0.025 (-0.2)	0.32[311]; 0.4	0.5	16-78	[4], [270], [1094]
Levomethadone[352]	0.04-0.4[339]	0.4[311]; 0.5[339]	0.04[8]; 0.1-0.2[362]	24-48 (-55)	[3, 4], [270], [450], [1095]
Levomilnacipran[570]	0.08-0.12[405]	0.2[311]		6-9 (-12)	[4], [780]
Levorphanol	0.007-0.02	0.1	0.9[8]; 2.7[8]	11-30	[8], [47], [1096]
Levothyroxine	0.045-0.14[47]			6-8 (-10) days	[451], [1097]
Lidocaine (Lignocaine)[571]	0.5-5[113]	6-7	10, 12[8]	1-4[113]	[47], [57], [70], [327], [452-454]
Linagliptin	0.002-0.004			50-90 (single dose); 110-150 (multiple dose)	[47], [780]
Lindane (ß-HCH, HCCH)	see Hexachlorocyclohexane				
Linezolid	0.5-4	appr. 7.5		4-6	[8], [823], [1098], [1186]
Lisinopril	(0.005-) 0.02-0.07	0.5[8]		4-12	[47], [84]
Lithium	4-8[79]	8[311]; 13	16	8-50[28]	[4], [47], [80], [264], [455-457]
Lofepramine[572]	0.003-0.01			0.5-5[572]	[47], [57], [458]
Loperamide	0.00024-0.0031[84]	0.12[8]	0.077[8]; 0.18[8]	7-15	[459], [857-860]
Lopinavir (LPV)	> 1.0[294]			5-6	[2], [110, 111]

Note on liability: The data and information have been extracted from scientific sources (primary and secondary literature) and have been carefully reviewed. Nevertheless, transcription errors cannot be excluded. No responsibility is taken for the correctness of the information or data included. As a matter of principle, this compilation should be used with expert knowledge.

Substance	Blood-plasma concentration (mg/L)			t½ (h)	References
	therapeutic (“normal”)	toxic (from)	comatose-fatal (from)		
Loprazolam	0.003-0.01			11-20	[59]
Loratadine[138]	0.001-0.02			2-15[138]	[460, 461]
Lorazepam	(0.02-) 0.03-0.25	0.3-0.5		10-16 (-40)	[4], [59], [224], [462, 463]
Lorcainide[573]	0.1-0.4 (-0.9)			5-10	[8], [464-466]
Lormetazepam[574]	0.002-0.01 (-0.025)	0.1[311]		8-15	[4], [59]
Lornoxicam	0.1-0.8			2-6	[8]
Losartan	< 0.2 (-0.65)[227]	1.8		1.5-2	[3], [8], [780]
Lovastatin[543]	0.003-0.018			2-3	[47], [1063]
Loxapine[23, 320]	0.005-0.03 (-0.1)	0.02[311]; 1	7.7	6-8[320]	[4], [8], [11], [58]
Lurasidone	0.015-0.04	0.12[311]		20-40	[4], [780]
Lysergide (lysergic acid diethyl amide, LSD)	0.0005-0.005	0.001	0.002-0.005	appr. 2-5	[8], [11], [58], [467]
Macitentan	0.2-0.4[620]			10-18	[47]
Magnesium	55-75[121]	120-140	150-180	2.1-2.9	[3], [47], [58], [468]
Malathione	-	0.35[8]; 0.5	1.8[8]; 175[8;] 517[8]	3-6	[47], [58]
Manganese	0.0005-0.0015		6.7[8, 575]	12-36 days	[47], [58]
Mannitol				0.25-1.2[83]	[3]

Note on liability: The data and information have been extracted from scientific sources (primary and secondary literature) and have been carefully reviewed. Nevertheless, transcription errors cannot be excluded. No responsibility is taken for the correctness of the information or data included. As a matter of principle, this compilation should be used with expert knowledge.

Substance	Blood-plasma concentration (mg/L) therapeutic ("normal")	toxic (from)	comatose-fatal (from)	t½ (h)	References
Maprotiline	0.075-0.13	0.22[311]; 0.5-1	1-5	20-60	[4], [8], [1084]
Maraviroc (MVC)	> 0.05[300]				[110]
MCPA	see 2-Methyl-4-chlorophenoxyacetic acid				
MCPP	see 2-Methyl-4-chlorophenoxypropionic acid				
MDA	see Methylenedioxy-amphetamine				
MDEA	see Methylenedioxyethyl-amphetamine				
MDMA	see Methylenedioxymethyl-amphetamine				
MDMB-PINACA	see 5-Fluoro ADB				
Mebendazole	≥ 0.1[67]			3-9	[47], [1099]
Meclizine (Meclozine)	0.001-0.045 (-0.15)			appr. 5-7	[47], [780], [861]
Meclofenamic acid (Meclofenamate)	appr. (1-) 2-7			0.8-5.3	[3], [780], [1214]
Mecoprop	see 2-Methyl-4-chlorophenoxy-propionic acid				
Medazepam[30]	0.1-0.5 (-1)	0.6		2-5[30]	[8], [47], [59], [61]

Note on liability: The data and information have been extracted from scientific sources (primary and secondary literature) and have been carefully reviewed. Nevertheless, transcription errors cannot be excluded. No responsibility is taken for the correctness of the information or data included. As a matter of principle, this compilation should be used with expert knowledge.

Substance	Blood-plasma concentration (mg/L)			t½ (h)	References
	therapeutic (“normal”)	toxic (from)	comatose-fatal (from)		
Mefenamic acid	2-10 (-20)	25		2-4	[9], [47]
Mefloquine	0.4-1[108]	1.5-2[8]		10-30 days	[47], [469, 470]
Melatonin	0.0005-0.1[379]			0.5-1	[47]
Melitracen	0.01-0.1			12-24	[8]
Meloxicam	0.4-2	4[311]		17-22	[8], [471]
Melperone	0.03-0.1	0.2[219, 311]	17[8]	4-8	[4], [8], [472, 473], [780]
Melphalan	-1.5			1.5-2	[8]
Memantine	0.09-0.15	0.3[311]	12[8]	60-100	[4], [47]
Meperidine	see Pethidine				
Mephenesin	3-10		16[8]	1-4	[8], [47]
Mepindolol	0.007-0.07			3-6	[7], [1100]
Mepivacaine	appr. 0.4-4[576]	5	50	1-3	[15], [58]
Meprobamate	5-10	10	30	6-17	[8], [56]
Meptazinol	0.025-0.25		16[8]	1-4	[8], [47], [1101]
Mercaptopurine[364]	0.03-0.1	1-2		0.5-1.5	[8], [47]
Mercury	appr. 0.0015-0.002 (< 0.005)[178]	0.05	0.5	> 18-24 days	[47], [64], [66], [100], [474-478]

Note on liability: The data and information have been extracted from scientific sources (primary and secondary literature) and have been carefully reviewed. Nevertheless, transcription errors cannot be excluded. No responsibility is taken for the correctness of the information or data included. As a matter of principle, this compilation should be used with expert knowledge.

Substance	Blood-plasma concentration (mg/L)			t½ (h)	References
	therapeutic ("normal")	toxic (from)	comatose-fatal (from)		
Meropenem	> 2-8 (-12)[387]	25		1 (-7)	[862]
Mescaline	1.5-3.8			6	[47]
Mesalazine (Mesalamine)	appr. -1[119]			0.5-2.4[120]	[479]
Mesoridazine	0.1-1	3-5	3[8]; 4[8]; 16[8]	2-9 (-20)	[15], [47], [58]
Mesuximide	see Methsuximide				
Metaclazepam	0.05-0.2			7-23	[8], [59]
Metamizole (Dipyrone)[5]	10[32]	20[32]		5-8	[8]
Metandienone (Methandienone)			0.008[8, 424]		[940]
Metaxalone	0.6-3	11[8]	37[8]	2-14	[47], [780]
Metformin	0.1-2	5-10[397]	91[8]; 119[8]; 166[8]	2-9	[8], [58], [480, 481], [863-865], [938, 939]
Methadone	(0.05-) 0.1-0.6[135]	0.3[339]; 0.6[311]	0.4 (0.05-1)[362]	24-48 (-55)	[4], [482-489], [1095]
Methamphetamine (Methylamphetamine)	-0.1	0.2-1	1-18[8]; 40[8]	6-15[344]	[8], [47]
Methanol	appr. -3[577]	200	900	10-12 (-24)[325]	[8], [47], [58], [325], [490]
Methapyrilene	appr. 0.1	4	4.4[8, 408]; 12[8]; 380[8]		[11], [771], [867]
Methaqualone	1-3	3-5	5-10	10-40	[8]
Methemoglobin (Met-Hb)	-[199]	25-30%	50-70%		[3], [10, 11], [467]

Note on liability: The data and information have been extracted from scientific sources (primary and secondary literature) and have been carefully reviewed. Nevertheless, transcription errors cannot be excluded. No responsibility is taken for the correctness of the information or data included. As a matter of principle, this compilation should be used with expert knowledge.

Substance	Blood-plasma concentration (mg/L) therapeutic (“normal”)	toxic (from)	comatose-fatal (from)	t½ (h)	References
Methimazole	0.5-2.5			2-28	[8], [467]
Methocarbamol	25-40 (-50)	250		1-2	[3], [11], [58]
Methohexital	0.5-5 (-11)[55]	2-20		1-6	[8, 9], [1107]
Methomyl	-	0.61[8]; 1.6[8]	1.6[8]; 26 (8-57)		[47]
Methotrexate (MTX)	0.01 – appr. 0.5[578]	0.75-1[579]	0.12[8]	(3-) 6-8 (low dose); 8-17 (high dose)	[3], [47], [1108]
Methotrimeprazine	see Levomepromazine				
Methoxsalen (8-Methoxypsoralene)	0.025-0.1 (-0.2)	0.6[8]; 1		0.5-1.5	[3], [10], [47], [58]
Methsuximide (Mesuximide)	10-40[223]	45[311]; 40-50		20-40 (-45)	[3, 4], [8], [11], [58], [491]
4’-Methyl-alpha-pyrrolidinohexanophenone (MPHP)	-		0.1[8]		[868]
2-Methyl-4-chlorophenoxyacetic acid (MCPA)	-	appr. 100	180[8]; 456[8]	12-72[187]	[3], [11], [47], [492]
2-Methyl-4-chlorophenoxypropionic acid (MCPP, Mecoprop)	-	appr. 100	669[8]; 715[8,181]	14-39[183]	[3], [11], [47]
Methyldopa	1-5	7[8]	9[8]	1.5-3 (4-14)	[9], [13, 14], [47]
3,4-Methylenedioxyamphetamine (MDA)[580]	-0.4	1.5	1.6-26[8]	(6-) 10.5-12.5 (-30)	[8], [47], [58], [404-406], [467], [493, 494]

Note on liability: The data and information have been extracted from scientific sources (primary and secondary literature) and have been carefully reviewed. Nevertheless, transcription errors cannot be excluded.
No responsibility is taken for the correctness of the information or data included. As a matter of principle, this compilation should be used with expert knowledge.

Substance	Blood-plasma concentration (mg/L)			t½ (h)	References
	therapeutic ("normal")	toxic (from)	comatose-fatal (from)		
3,4-Methylenedioxyethylamphetamine (MDEA, MDE)	-0.2		1-4.2[8]; 12[8]	4-8[334]	[47], [493], [495-497]
3,4-Methylenedioxymethylamphetamine (MDMA, Ecstasy, XTC)	0.1-0.35[236]	0.35-0.5	0.4-0.8; 2.9[8]	(4-) 7-8 (-10)	[8], [58], [404-406], [467], [493, 494], [498, 499]
3-Methyl-fentanyl	-		0.3-1.9 ng /mL		[1071, 1072]
Methylphenidate	0.01-0.06	0.05[311]; 0.1-0.5; 0.107[8, 407]	2	2-7	[4], [8], [270], [866]
Methylphenobarbital (Mephobarbital)	see Phenobarbital				
4-Methylthioamphetamine (4-MTA, p-MTA)		1[8]	2[8]; 4.6[8]; 7.4[8]	7	[47], [500-504]
Methyprylon(e)[581]	1-20	12-25	50; 66[8]	3-11	[8, 9], [1109, 1110]
Metiamide	0.01-0.06				[58], [1111]
Metildigoxin (Methyldigoxin)[3]	see Digoxin				
Metipranolol[33]	0.02-0.08 (-0.17)			2-3.5	[15], [869]
Metoclopramide	(0.01-) 0.05-0.15	0.2	4.4[8]	3-6	[15], [66]
Metocurine	appr. 0.4-1			5-7	[1112]
Metolazone	0.005-0.05			6-11	[47], [780], [1113]
Metoprolol[48]	(0.02-) 0.035-0.5 (-0.6)	0.65[8]; 7.8; 12[8]	4.7[8]; 18[8]; 25[8]; 63[8]; 142[8]	2.5-7.5	[3], [7], [47], [870]

Note on liability: The data and information have been extracted from scientific sources (primary and secondary literature) and have been carefully reviewed. Nevertheless, transcription errors cannot be excluded. No responsibility is taken for the correctness of the information or data included. As a matter of principle, this compilation should be used with expert knowledge.

**Substance**	**Blood-plasma concentration (mg/L)** therapeutic ("normal")	toxic (from)	comatose-fatal (from)	**t½ (h)**	**References**
Metrifonate[582]	appr. 1.4-3.6			2-5	[8], [47]
Metronidazole	3-10 (-30)			6-14	[8], [1186]
Mexiletine	0.5-2	1.5-2	21-45[8]	6-17 (-26)	[47], [70], [505]
Mexoryl SX	see Ecamsule				
Mianserin	0.015-0.07	0.14[311]; 0.25-0.5	1.6[8]; 8.6[8]	14-33	[4], [8], [56], [223], [1084]
Mibefradil	appr. 0.2-0.3			17-25	[8]
Miconazole	< 1			24	[8]
Midazolam	0.04-0.1 (-0.25)[134]	1-1.5		1-3[46]	[8], [61], [224], [506-508]
Mifepristone (RU-486)	0.1-4[216]			(20-) 24-48 (-54)	[47], [509], [1114]
Milnacipran	(0.05-) 0.1-0.15	0.3[311]	22[8]	5-8	[4], [1115]
Milrinone	0.15-0.25	0.3		1-3	[3], [8], [42], [95]
Minaprine	appr. 0.1-0.4 (-1)?			appr. 25-34	[1119, 1120]
Minoxidil	appr. 0.02-0.2[149]	3.1[8]		1-4	[3], [84], [1116]
Mirabegron	0.005-0.03 (-0.1)			30-50	[780], [1117]
Mirtazapine	0.03-0.08 (-0.3)	0.16[311]; 1-2	2-3	20-40[188]	[3, 4], [510], [1084]
Misoprostol	-[286]			0.5-1	[3], [511, 512], [1118]

Note on liability: The data and information have been extracted from scientific sources (primary and secondary literature) and have been carefully reviewed. Nevertheless, transcription errors cannot be excluded. No responsibility is taken for the correctness of the information or data included. As a matter of principle, this compilation should be used with expert knowledge.

Substance	Blood-plasma concentration (mg/L) therapeutic ("normal")	toxic (from)	comatose-fatal (from)	t½ (h)	References
Mitotane	14-20	20		18-159 days	[513]
Mitragynine (Kratom)[467]	0.02-0.1[103, 585]	[339]	[413]; 0.23[8]; 0.6[8]; 1.06[8]	23 ± 16	[241], [871-877]
Mizolastine	appr. 0.2-0.8			5-17	[3], [8], [47]
Moclobemide[141]	0.3-1.0 (-3)	2[311]; (2.8; 18; 60.9)[586]	11[162]; 30	1-7	[514-519], [1084, 1085], [1127]
Modafinil	1-1.7 (-4)[257]	3.4[311]	35[8]	10-15	[4], [89], [520], [1126]
Moexiprilat[587]	0.005-0.04			2-10	[8]
Molindone	0.005-0.5	0.15[8]	6[8]; 9.3[8]	1.2-2.8	[47]
Molsidomine	0.002-0.03			1-2.5	[95]
Molybdenum	-0.005[588]	0.0077[8]			[47], [1129]
Montelukast (MK-0476)	0.02-0.3	0.6[311]		3-6	[3], [521], [1130]
Moricizine[23]	0.12-1.27			(3-) 6-13	[8], [467], [1131, 1132]
Morphine[288]	0.01-0.1	0.1[339]	0.1[339]	1-4	[87], [522-525]
Moxonidine	0.001-0.002 (-0.004)			2-4	[8], [95]
MT45	see 1-Cyclohexyl-4-(1,2-diphenylethyl)piperazine				
Muromonab-CD3 (OKT 3)	0.1-1.5			appr. 18	[1133, 1134]

Note on liability: The data and information have been extracted from scientific sources (primary and secondary literature) and have been carefully reviewed. Nevertheless, transcription errors cannot be excluded. No responsibility is taken for the correctness of the information or data included. As a matter of principle, this compilation should be used with expert knowledge.

Substance	Blood-plasma concentration (mg/L)			t½ (h)	References
	therapeutic (“normal”)	toxic (from)	comatose-fatal (from)		
Mycophenolate mofetil[212]	0.3-3.5[211]	44[8]		16-18[212]	[526-529], [1135]
Nabumetone	-[206]			-[206]	[47], [530], [780]
Nadolol	0.01-0.25		1.3[8]	(14-) 20-24	[7, 8], [47]
Naftidrofuryl (Nafronyl)	< 0.5		7.5[8]	1-2	[47], [1136]
Nalbuphine	0.02-0.2			2.5-7	[8]
Nalidixic acid	10-30	40-50		1-2 (-7)	[3], [8, 9]
Nalmefene	0.01-0.05	0.2[311]		5-11	[3, 4]
Naloxone	0.01-0.03			1-2	[8]
Naltrexone	0.003-0.05[99]	0.2[311, 589]		2-5 (-13)	[4]
Naphyrone (Naphthylpyrovalerone)		0.03[335]		appr. 34	[531]
Naproxen	(20-) 50-100	200; 414[8]	840[8]; 1040[8]; 1320[8, 599]	9-22	[8], [47], [293], [343], [532, 533], [1155]
Naratriptan	appr. 0.01-0.05			5-6	[3], [8]
Nateglinide	0.09-1.2 (-6)			1-2	[47], [780], [1136]
25I-NBOMe 2-(4-iodo-2, 5-dimethoxyphenyl)-N-(2-methoxybenzyl)ethanamine		0.3-2.8 ng/mL	0.24[8]; 0.76[8]; 4.7[8]; 16[8]		[47], [1050-1052]
Nebivolol[48]	0.001-0.02 (-0.06)[620]	0.12[311]; 0.48[8]		8-14 (-27)	[3], [8], [47], [534-536], [780]

Note on liability: The data and information have been extracted from scientific sources (primary and secondary literature) and have been carefully reviewed. Nevertheless, transcription errors cannot be excluded. No responsibility is taken for the correctness of the information or data included. As a matter of principle, this compilation should be used with expert knowledge.

Substance	Blood-plasma concentration (mg/L)			t½ (h)	References
	therapeutic ("normal")	toxic (from)	comatose-fatal (from)		
Nedocromil	< 0.02			1.5-3.3	[3], [47]
Nefazodone	0.03-2.5[220]	5.5[8, 221]		2-6[222]	[3], [8], [537-542]
Nefopam	0.01-0.1	0.6[8]; 4[8]	12[8]	3-8	[8], [47]
Nelfinavir (NFV)	> 0.8[295]			3.5-5	[2], [87], [110, 111]
Neostigmine	appr. 0.001-0.01[127]			0.4-1.3	[316], [543]
Netilmicin	0.5-2; 6-10[590]	appr. 15-20		0.5-3[80]	[8], [878], [1137]
Nevirapine (NVP)	> 3.0[298]			25-30	[2], [110, 111]
Nicardipine	0.07-0.1		5[8]	7-12	[84], [1138]
Nickel	0.0015-0-005[330]	appr. 3[8]	7.5[8]; 19[8]		[47], [58], [100]
Nicotine	0.005-0.03[123]	0.4[8]	1-2[461, 462]	1-4[124]	[3], [8], [270], [544, 545], [1007-1010]
Nicotinic acid	4-18			0.3-1	[8]
Nifedipine	0.01-0.2		0.15[8]; 1.2[8]; 5.4[8]	2-5	[47], [84]
Niflumic acid	2-35			2-3	[8]
Nilvadipine	< 0.01			9-13	[84], [1139]
Nimesulide[235]	3-6.5			2-7 (11-20)	[3], [8], [546]
Nimodipine	0.01-0.05			1-2	[8], [84]

Note on liability: The data and information have been extracted from scientific sources (primary and secondary literature) and have been carefully reviewed. Nevertheless, transcription errors cannot be excluded.
No responsibility is taken for the correctness of the information or data included. As a matter of principle, this compilation should be used with expert knowledge.

Substance	Blood-plasma concentration (mg/L)			t½ (h)	References
	therapeutic ("normal")	toxic (from)	comatose-fatal (from)		
Nimustine (ACNU)	0.2-0.5 ng/mL				[58]
Nisoldipine	0.001-0.003	1.5[8]		7-12 (-15)	[3], [47], [84]
Nitrazepam	0.03-0.1	0.2[311]	5	18-30	[4], [8], [59]
Nitrendipine	(0.005-) 0.01-0.05			8-12	[8], [84]
p-Nitroaniline (4-Nitroaniline)	-		4.2[8]		[547]
Nitrofurantoin	0.5-5			0.7-1.5	[8], [1140]
Nitroglycerin (Glyceryl trinitrate, GTN)	appr. -0.015			20-30 minutes	[548]
Nitroprusside	see Thiocyanate	see also Cyanide			
Nizatidine	0.05-1			0.7-2.1	[8], [47]
Nomifensin(e)	0.01-0.1	8	17[8]	2-5	[8], [87]
Norclozapine (N-Desmethylclozapine)	see Clozapine			appr.8	[4]
Nordazepam (Desmethlydiazepam)[23]	(0.02[273]-) 0.12 -0.8	1.5[311]		30-90	[4], [8], [284, 285], [467]
Nordiazepam	see Nordazepam				
Norephedrine	see Phenylpropanolamine				
Norfenefrine	< 0.002 (-0.4)			(2-) 3-7	[8], [1141]
Norfloxacin	0.5-5			3-4 (-6)	[3], [47], [58]

Note on liability: The data and information have been extracted from scientific sources (primary and secondary literature) and have been carefully reviewed. Nevertheless, transcription errors cannot be excluded. No responsibility is taken for the correctness of the information or data included. As a matter of principle, this compilation should be used with expert knowledge.

Substance	Blood-plasma concentration (mg/L)			t½ (h)	References
	therapeutic ("normal")	toxic (from)	comatose-fatal (from)		
Norfluoxetine (N-Desmethylfluoxetine)	see Fluoxetine			7-9 (4-16) days	[4], [1084]
Normesuximide (N-Desmethylmesuximide)[31]	10-40	45[311]		36-45	[4]
Norsertraline (Desmethylsertraline, DMS)	see Sertraline			appr. 70	[4], [879]
Nortriptyline[48] (see also Amitriptyline)	0.07-0.17 (0.05-0.15)	0.3[311]; 0.5	1-3	18-44 (-56)[68]	[4], [76], [80-82], [223], [1084]
Noscapine	0.02-0.4			1.5-4	[47]
Obidoxime	1-10 (appr. 10-20 µmol/L)			1-12	[58], [549], [1142]
Ocfentanil[395]	-		3.6[8]; 9.1[8, 396;] 15.3[8] ng/mL		[880-883], [1071]
Octisalate	0.001-0.006 (-0.017)[583]			27-77	[1122]
Octinoxate	0.001-0.008 (-0.03)[583]			50-157	[1122]
Octocrylene	0.001-0.02[419]			50-80	[801], [1122]
Ofloxacin	2-3 (-5.5)	39[8]		5-8	[3], [550], [1143]
OKT 3	see Muromonab-CD3				
Olanzapine[548]	(0.001-) 0.02-0.08[548]	0.1[311]; 0.12[8]	0.25[8]; 1[8]; 2.5[8]; 4.9[8]	(20-) 30-60	[4], [72], [101], [551-555]
Olmesartan	0.1-1			6-15	[47], [780]
Omecamtiv mecarbil	0.03-0.5	appr. > 1.2		18-21	[884-888]

Note on liability: The data and information have been extracted from scientific sources (primary and secondary literature) and have been carefully reviewed. Nevertheless, transcription errors cannot be excluded.
No responsibility is taken for the correctness of the information or data included. As a matter of principle, this compilation should be used with expert knowledge.

Substance	Blood-plasma concentration (mg/L)			t½ (h)	References
	therapeutic ("normal")	toxic (from)	comatose-fatal (from)		
Omeprazole[48]	0.05-4[98]	8[311]		0.5-1 (-1.5)	[780]
Ondansetron	0.03-0.3			3-5.5	[8], [66]
Opipramol	0.05-0.5	1[311]	3[8]-10	6-12[262]	[4], [8], [43], [556]
Orphenadrine	(0.05-) 0.1-0.2 (-0.8)	1.7	3.6[8]; 5	13-20	[9], [47], [56], [296], [557]
Oxaprozin	60-200			30-70	[47], [780]
Oxatomide	0.02-0.1	0.25	0.9[8]	14-30	[3], [47]
Oxazepam	0.2-1.5	2[311]	3.5; 4.4[8]; 5.3[8]	4-20	[3, 4], [56], [59], [283], [1231]
Oxazolam	see Nordazepam				[1145]
Oxcarbazepine	10-35[172]	35[8]; 40[311]-45		1-5[172]	[3, 4], [12], [47], [381], [558-560]
Oxpentifylline	see Pentoxyfylline				
Oxprenolol	0.05-0.3 (-1.0)	2-3	10	1-4	[7, 8]
Oxybenzone	0.083-0.532[419]			appr. 79[583]	[801], [1122]
Oxybutinin (Oxybutynin)[590]	0.001-0.02			2-5 (-12)	[8], [47], [780]
Oxycodone[48, 591]	0.005-0.1[339]	0.2[339]	0.6-0.7; 5[8]	2-5[53]	[47], [58], [244], [270], [1231]
Oxyfedrine	appr. 0.06			4.2	[95]
Oxymorphone[592]	0.5-5 ng/mL[339]	-[339]		(4-) 7-9 (-12)	[47], [780], [1146]

Note on liability: The data and information have been extracted from scientific sources (primary and secondary literature) and have been carefully reviewed. Nevertheless, transcription errors cannot be excluded. No responsibility is taken for the correctness of the information or data included. As a matter of principle, this compilation should be used with expert knowledge.

Substance	Blood-plasma concentration (mg/L) therapeutic ("normal")	toxic (from)	comatose-fatal (from)	t½ (h)	References
Oxyphenbutazone	25-100	200		48-72	[8], [47]
Oxypurinol[61]	5-15	20		18-30	[8], [11], [58]
Oxytocin	appr. -0.2 ng/mL			3-5 minutes	[3]
Paclitaxel	0.25-7[122]	-[593]		(4-) 8-20	[8], [47], [1147]
Paliperidone (9-Hydroxyrisperidone)[550]	0.02-0.06	0.12[311]		17-24 (-30)	[4], [47], [1194]
Pamidronate (Pamidronic acid)	< 0.02[323]			days[594]	[49], [561]
Pancuronium	0.025-0.1 (-0.6)[606]	0.4[8, 198]	0.7[8]; 1.6[8]	1.5-2.5	[8], [47], [562]
Pantoprazole	1.5-14[98]	28[311]		1-2	[3], [8], [47]
Papaverine	0.2-2			1-2 (6-7)	[47], [57], [780]
Paracetamol	(5-) 10-25	100-150[595]	200-300	2-4	[243], [563-571], [1148, 1149]
Paraldehyde	10-100	200	500	(3-) 4-10	[48], [87]
Paraoxon[596]	-	0.005			[11]
Paraquat	-	0.05	0.12[8, 348]; 1-2[201]	8-12[349]	[3], [11], [572-580]
Parathion[596]	-	0.01-0.05	0.05-0.08		[3], [10], [58]
Paroxetine[48]	0.002-0.065	0.12[311]; 0.4	1.2[8]; 3.7[8]; 4[8, 306]	16-24[93]	[4], [202], [263], [368], [581-583], [1084, 1085]
Pefloxacin	(0.1-) 1-10	25		8-15	[8, 9], [58]

Note on liability: The data and information have been extracted from scientific sources (primary and secondary literature) and have been carefully reviewed. Nevertheless, transcription errors cannot be excluded.
No responsibility is taken for the correctness of the information or data included. As a matter of principle, this compilation should be used with expert knowledge.

Substance	Blood-plasma concentration (mg/L) therapeutic ("normal")	toxic (from)	comatose-fatal (from)	t½ (h)	References
Pemoline	1-7			7-13	[8]
Penbutolol	0.01-0.3 (-1.0)			17-26	[7-9]
Penfluridol	0.004-0.025			70	[3], [58]
(D-)Penicillamine	1.7-5.6 (-11)			1-3	[8]
Penicillin G	0.1-2.5 (-10)			0.5-1	[9], [780]
Pentachlorophenol (PCP)	-0.2	30	38[8]; 45	13-19 days	[11], [47]
Pentamidine	0.3-0.5	appr. 0.8		6-9	[3], [8]
Pentazocine	0.01-0.2	1-2[339]	3	2-5	[8]
Pentobarbital[598]	1-5	5	10	20-48	[8, 9], [47], [87]
Pentoxifylline[72]	appr. 0.5-2	4[311]	33[8]	0.5-3	[8], [47], [780]
Pentoxyverine	-0.18			2-3 (-6)	[8], [47]
Perampanel	0.18-0.98	1[311]		48-105	[4]
Perazine	(0.01-) 0.1-0.23	0.46[311]; 6.1[8]	9.6[8]	8-16 (-35)	[4], [8], [584], [1156]
Perhexiline	0.11-0.6[309]	0.6-1.2		(7-) 12-18 (-23)	[3], [585, 586]
Periciazine (Pericyazine)[597]	0.005-0.03	0.1		7-9	[9], [1154]
Perindopril[600]	0.08-0.15			0.8-3[265]	[3], [8]

Note on liability: The data and information have been extracted from scientific sources (primary and secondary literature) and have been carefully reviewed. Nevertheless, transcription errors cannot be excluded. No responsibility is taken for the correctness of the information or data included. As a matter of principle, this compilation should be used with expert knowledge.

Substance	Blood-plasma concentration (mg/L)			t½ (h)	References
	therapeutic ("normal")	toxic (from)	comatose-fatal (from)		
Perphenazine	0.6-2.4 ng/mL[161]	5 ng/mL[311]		8-12 (-21)	[4], [587]
Pethidine (Meperidine)[115]	0.1-0.8[115]	1-2[339]	1 (-2)	3-6 (-10)	[588-592]
Phenacetin[62]	5-10 (-20)	50		appr. 1	[9], [13], [779]
Phenazepam	0.02-0.04[601]		1[8]; 1.6[8]	60	[47], [1157]
Phenazone (Antipyrine)	1-25	50		10-12 (-16)	[8, 9]
Phencyclidine (PCP)	0.01-0.2	0.007-0.24 (-0.8)	(0.3-) 1-5	1-12 (-50)	[8], [58], [467], [779]
Phendimetrazine	0.02-0.1		0.3[8]; 0.7[8]	2-4	[8], [47], [1158]
Phenelzine	0.001-0.002 (-0.2)	0.5	1.5[8]	6-8	[3], [58]
Pheneturide		5-20		30-90	[12]
Phenformin	0.03-0.1	0.6	3	4-13	[3], [8]
Pheniramine	0.01-0.27		appr. 2	16-19	[3], [8]
Phenmetrazine	0.02-0.25	0.5	4	appr. 8	[8]
Phenobarbital	10-40	40; 50[311]	50-60	60-130	[4], [8], [12], [161], [164], [415]
Phenol	< 10 (in urine)	50; 21.6[8]	50[8]; 90[8]	0.5-1 (-4.5); 14[8]	[11], [47], [889]
Phenprocoumon	1-3 (-5)[602]	5		100-160[35]	[11], [58]
Phensuximide	4-10 (-20)	80		4-12	[8], [58], [779]

Note on liability: The data and information have been extracted from scientific sources (primary and secondary literature) and have been carefully reviewed. Nevertheless, transcription errors cannot be excluded. No responsibility is taken for the correctness of the information or data included. As a matter of principle, this compilation should be used with expert knowledge.

Substance	Blood-plasma concentration (mg/L) therapeutic ("normal")	toxic (from)	comatose-fatal (from)	t½ (h)	References
Phentermine	0.03-0.1	0.9	1; 7.6[8]	appr. 20	[8], [47], [58]
Phenylbutazone[36]	50-100	120-200	400	30-175[37]	[9], [13]
Phenylephrine	0.4-3.4 ng/mL			0.5-4	[47], [168], [890-892]
Phenylpropanolamine (Norephedrine)	0.1-0.5	2	4.6[8]; 48[8]	3-7	[9], [47]
Phenytoin	(5-15) 10-20[81]	20-25[311]	38[8]; 43[8]	10-60[37]	[4], [161], [164], [593-600]
Pholcodine	0.02-0.08	0.1	1; 2.5[8]	35-75	[47], [1159]
Physostigmine	0.04-0.06			0.3-1.5	[303], [316], [601]
Pimozide	(0.003-) 0.015-0.02	0.02[311]		24-55 (-214)	[4], [47], [1160]
Pinazepam[15]	0.01-0.05			16	[59], [1161]
Pindolol	0.02-0.15	0.7-1.5		2-5	[7]
Pioglitazone	0.4-2			3-11 (-20)	[47], [780], [893], [1194]
Pipamperone	0.1-0.4	0.5[311]; 3.2[8]		17-22	[4], [47]
Piperacillin	1-5 (20-70)			1-2 (-4)	[8], [58], [1186]
Piperazine	0.02-0.1	0.5			[42], [58]
Pipotiazine	0.001-0.06	0.1		8-11	[3], [8, 9], [58]
Piracetam	appr. 20-50			4.5-7	[8]

Note on liability: The data and information have been extracted from scientific sources (primary and secondary literature) and have been carefully reviewed. Nevertheless, transcription errors cannot be excluded.
No responsibility is taken for the correctness of the information or data included. As a matter of principle, this compilation should be used with expert knowledge.

Substance	Blood-plasma concentration (mg/L) therapeutic ("normal")	toxic (from)	comatose-fatal (from)	t½ (h)	References
Pirenzepine	0.03-0.45			8-20	[8]
Piretanide	appr. 0.1-1 (-4)[620]			0.6-1.7[83]	[47], [1203, 1204]
Piritramide	0.0035-0.014[128]	-[128, 339]	0.11[8]	4-10	[8], [47], [602]
Pirmenol	1-4			6-18	[3], [8], [11]
Piroxicam	2-6	14[8]		30-70	[8]
Pitavastatin	0.031-0.081			5-13	[47], [1063]
Pizotifen (Pizotyline)	0.007-0.009			26	[58]
Posaconazole	> 0.5-0.7[603]			20-66	[47], [603-605], [894, 895], [1162]
Practolol	1.5-5			5-13	[7-9]
Prajmalium (Prajmaline)[48]	0.06-0.44		3.9[8]	5-7	[8], [47]
Pramipexole	appr. 0.0002-0.007	0.015[311]		8-14	[4], [8], [467]
Pranlukast	appr. 0.2-1.2			appr. 2-9	[3], [8]
Pravastatin	0.0025-0.0063			appr. 3	[780]
Prazepam[15, 418]	see Nordazepam			1-3	[8], [47], [59], [896]
Praziquantel	appr. 0.2			1-2.5	[8], [1163]
Prazosin	0.001-0.02	0.9		2-3	[42], [47], [95]

Note on liability: The data and information have been extracted from scientific sources (primary and secondary literature) and have been carefully reviewed. Nevertheless, transcription errors cannot be excluded.
No responsibility is taken for the correctness of the information or data included. As a matter of principle, this compilation should be used with expert knowledge.

Substance	Blood-plasma concentration (mg/L)			t½ (h)	References
	therapeutic ("normal")	toxic (from)	comatose-fatal (from)		
Prednisolone	0.5-1	2[311]		2-6	[8], [780]
Prednisone	see Prednisolone				
Pregabalin	2-8 (-17)	10[311]; 13[8]; 60[8]; 67[8]	76[8]; 110[8]	appr. 6	[4], [373], [606, 607], [842], [897-899]
Prilocaine	0.5-1.5 (-2)[126]	5-6	appr. 20	1-2	[8], [327], [608]
Primaquine	appr. 0.1-0.2			4-7	[8]
Primidone[63]	5-10 (-15)	25[311]	65[8]	4-12 (-22)	[4], [8], [47], [161], [164]
Probenecid	20-200			3-17[37]	[8], [47]
Procaine	0.2-2.5 (-15)	20		-0.5	[15], [58]
Procainamide[5]	2.5-14	8-15	20	2-5 (-8)	[8], [70], [609]
Prochlorperazine	(0.001-) 0.01-0.05	0.2-0.3	5[8]	7-9 (-18)	[3], [9], [47], [58]
Procyclidine	0.08-1	1-2	7.8[8]	7-16	[8], [11]
Proguanil[48]	appr. 0.04-0.15[114]			13-24[114]	[58], [610]
Promazine	0.01-0.05 (-0.4)	1	5	5-41 (8 ± 7)	[8]
Promethazine	0.01-0.05 (-0.2)	0.1[311]; 1-2	2.4[8]; 1.8-5.4[250]	5-20	[4], [8], [56], [611, 612]
Propafenone[48]	(0.04-) 0.3-2	1.1[8]; 2	1.4[8]; 7.7[8]; 9[8]	2-10[48], 10-32[48]	[42], [47], [70], [613]
Propallylonal	0.3-10	appr. > 10		appr. 3	[8]

Note on liability: The data and information have been extracted from scientific sources (primary and secondary literature) and have been carefully reviewed. Nevertheless, transcription errors cannot be excluded.
No responsibility is taken for the correctness of the information or data included. As a matter of principle, this compilation should be used with expert knowledge.

Substance	Blood-plasma concentration (mg/L) therapeutic ("normal")	toxic (from)	comatose-fatal (from)	t½ (h)	References
2-Propanol	see Isopropanol				
Propantheline	0.002-0.05			1-4	[3], [47]
Propiomazine	0.05-0.2		0.1-5.4[8]	2-15	[47], [56], [614]
Propofol	1-8		2.4[8]; 2.5[8]; 4.3[8]; 5.3[8]	3-8[91]	[615, 616], [900-903]
Propoxyphene	see Dextropropoxyphene				
Propoxur	-		0.3[8]		[87]
Propranolol	0.02-0.3[403]	1	4-10	2-6	[7], [9], [42], [112], [1153]
Propylene glycol	0.05-0.5[403]	1000; 4700[8]		2-5	[47], [58], [617]
Propylhexedrine	0.01	0.5	1.7[8, 414]; 2-3	1 (nasal inhalation); 4 ± 1.5 (oral)	[58], [871], [904], [1164]
Propyphenazone	1.5-12.5			1-3	[8, 9], [1165, 1166]
Prothipendyl	0.03-0.08	0.05[311]; 0.5 (-1)	60[8]	2-3	[4], [8], [1167]
Protriptyline	0.05-0.3	0.5	1; 20.7[8]	50-200	[8], [58], [87]
Pseudoephedrine	(0.05-) 0.5-0.8	1.6[311]	19-20[280]	9-16	[11], [58], [266, 267], [269], [618, 619]
Psilocin[380]	appr. 0.008	0.018[8]		1.8-4.5	[47], [1168]
Psilocybin	see Psilocin				

Note on liability: The data and information have been extracted from scientific sources (primary and secondary literature) and have been carefully reviewed. Nevertheless, transcription errors cannot be excluded. No responsibility is taken for the correctness of the information or data included. As a matter of principle, this compilation should be used with expert knowledge.

Substance	Blood-plasma concentration (mg/L) therapeutic ("normal")	toxic (from)	comatose-fatal (from)	t½ (h)	References
Pyrazinamide	30-75			(5-) 9-10 (-25)	[3], [9], [620]
Pyridostigmine	< 0.05-0.2			0.5-1.5 (-2.5)	[316], [621, 622]
Pyridoxine (Vitamin B_6)	0.003-0.018			3-6	[66], [800]
Pyrilamine			11[8]; 5-27[8]		[47], [87]
Pyrimethamine	0.1-2		1-10[8]	70-96	[3], [47], [1169]
Pyrithyldione	1-10			11-20	[9], [14]
Quazepam	0.01-0.05 (-0.15)[131]			39 (25-41)	[8], [11], [58, 59]
Quetiapine[549]	0.1-0.5[239, 600]	1[311]; 1.8	0.95[8]; 1.4[8]; 1.9[8]; 9[8]; 12.7[8]	appr. 5-7[240]	[4], [47], [87], [623-628], [807], [905], [1171], [1187]
Quinapril[617]	0.15-0.5[617, 620]			2-3[618]	[47]
Quinidine	2-5 (-8)	8-10	10-28[8]	4-12	[23], [70], [1170]
Quinine	1-7	10		4-15	[8], [58], [629, 630]
Rabeprazole	0.2-1.8	3.6[311]		1-2	[8], [780]
Raloxifene	0.67-1.3 ng/mL			15-45	[47], [780]
Raltegravir (RAL)	0.072 (0.029-0.118)[303]			7-12	[47], [110]
Ramipril	appr. 0.001-0.04[228]	0.08[311]		1-5	[3], [8], [780]
Ranitidine	0.05-1	3[311]		2-4	[8], [631], [780]

Note on liability: The data and information have been extracted from scientific sources (primary and secondary literature) and have been carefully reviewed. Nevertheless, transcription errors cannot be excluded. No responsibility is taken for the correctness of the information or data included. As a matter of principle, this compilation should be used with expert knowledge.

Substance	Blood-plasma concentration (mg/L)			t½ (h)	References
	therapeutic (“normal”)	toxic (from)	comatose-fatal (from)		
Ranolazine	0.37-1.1			3-15	[47], [780], [1172]
Rapamycin	see Sirolimus				
Reboxetine	0.06-0.35 [281]	0.7[311]		(8-) 12-14 (-30)	[3, 4], [632, 633]
Recainam	1.3-5.7			5-7	[634], [1173]
Remacemide	appr. 0.1-1			4	[8]
Remifentanil	-0.02			3-10 minutes	[8], [1071], [1174]
Remoxipride	2.15 ± 0.59[132]		41-150	5-10	[56], [635, 636]
Repaglinide	0.004-0.06 (-0.2)			0.5-2.5	[47], [780], [1175]
Reserpine	-0.0015-0.003			75 (2-12 days)	[47], [780], [1176]
Retigabine	0.45-0.9	1.8[311]		8-10	[4]
Retinol (Vitamin A)	0.2-0.8 (0.7-2.8 µmol/L)		1.2[8]		[66], [637], [800]
Ricin	0.003-0.01[601]	1.5[8]			[47], [638], [1177]
Rifabutin	0.05-0.15 (-1.1)			24-58	[3], [47], [1178]
Rifampicin (Rifampin)	0.1-10[101]	204[8]	55[8]	1-6[37]	[47], [317], [620], [639]
Rifapentine	4-24			10-15	[47], [620], [1179]
Rilmenidine	0.003-0.006[620]			6-10	[47]

Note on liability: The data and information have been extracted from scientific sources (primary and secondary literature) and have been carefully reviewed. Nevertheless, transcription errors cannot be excluded.
No responsibility is taken for the correctness of the information or data included. As a matter of principle, this compilation should be used with expert knowledge.

Substance	Blood-plasma concentration (mg/L) therapeutic ("normal")	toxic (from)	comatose-fatal (from)	t½ (h)	References
Riluzole	0.05-0.5 (-1.5)			(5-) 9-15	[8], [47], [467]
Risedronat (Risedronic acid)	appr. 0.4-5 ng/mL			4-12 days	[47], [49], [998], [1180]
Risperidone[48, 550]	0.02-0.06[272]	0.12[311]	1.8[8]	2-4[159]	[4], [47], [457], [640, 641], [906, 907]
Ritonavir	appr. 5-11 (-20)			3-5	[2, 3], [642]
Rivaroxaban	(0.001-) 0.01-0.36	0.005-0.35		5-9; elderly 11-13	[794], [796, 797], [819], [908], [960], [1181]
Rivastigmine	0.008-0.02	0.04[311]		1-2 (oral); appr. 3 (transdermal patch)	[4], [8], [303], [643, 644]
Rizatriptan	0.015-0.1			2-3	[3], [8], [47]
Rocuronium	5-10 (-17)[606]		1.5[8]; 4.9[8]	appr. 1.5	[8], [47], [1182]
Roflumilast[158]	0.0013-0.0024			10-30	[47], [780], [1183]
Ropinirole	0.4-6 ng/mL	12 ng/mL[311]		2-10	[4], [8], [645]
Ropivacaine	0.4-1.8	(1-) 2[173]		2[168]	[47], [646-648]
Rosiglitazone[116]	0.1-0.3 (-0.65)			3-4	[8], [1184]
Rosuvastatin	0.006-0.02			12-32	[47], [780], [1063]
Rotigotine	0.1-0.7 ng/mL	2 ng/mL[311]		5-7	[4]
Roxatidine	0.1-0.8			5-6	[8]

Note on liability: The data and information have been extracted from scientific sources (primary and secondary literature) and have been carefully reviewed. Nevertheless, transcription errors cannot be excluded.
No responsibility is taken for the correctness of the information or data included. As a matter of principle, this compilation should be used with expert knowledge.

Substance	Blood-plasma concentration (mg/L) therapeutic ("normal")	toxic (from)	comatose-fatal (from)	t½ (h)	References
Roxithromycin	4-12			6-15	[3], [47], [115]
RU-486	see Mifepristone				
Rufinamide	(2-) 5-30	40[311]		6-10	[4], [47]
Sacubitril[415]	appr. -3<br>(appr. -16 for Sacubitrilat)			3.9 ± 3.6[416]	[909]
Salbutamol (Albuterol)	0.004-0.014 (-0.02)	0.018-0.45	0.16	3-6	[58], [649], [1185]
Salicylamide	5-40			appr. 1	[8]
Salicylic acid[609]	20-200	300-400	(400-) 500	3-20	[3], [28], [30-33]
Salvinorin A	0.01-0.04[610]			40-80 minutes	[29], [47]
Saquinavir (SQV)	> 0.1-0.25[296]			(1-) 3-7 (-12)	[2], [47], [110, 111]
Saxagliptin[420]	0.008-0.025			2.5	[780], [910-912]
Scopolamine	0.1-0.3 (-1) ng/mL		1.2 ng/mL[8]	2-6	[8], [11], [47]
Secbutabarbital	5-10 (-15)	20	30	34-42	[8]
Secobarbital	1.5-5	7-10	10-15	15-30	[8], [87]
Selegiline	see Amphetamine and Methamphetamine				
Selenium	0.045-0.13 (-0.19)	0.4-1	2; (2.6[8]; 2.8[8]; 18.4[8]; 38[8])	69-77 days	[3], [47], [87], [650-652]

Note on liability: The data and information have been extracted from scientific sources (primary and secondary literature) and have been carefully reviewed. Nevertheless, transcription errors cannot be excluded.
No responsibility is taken for the correctness of the information or data included. As a matter of principle, this compilation should be used with expert knowledge.

Substance	Blood-plasma concentration (mg/L) therapeutic ("normal")	toxic (from)	comatose-fatal (from)	$t_{½}$ (h)	References
Seratrodast	3-25				[8], [1188]
Sertindole	0.05-0.1	0.2[311]		55-90	[4], [101]
Sertraline[423]	0.01-0.15 (-0.5)	0.29[8]; 0.3[311]	1.1-1.5; 1.6[8]; 3[8]	(22-) 24-28 (36)	[4], [58], [223], [653], [879], [1084, 1085]
Sevoflurane	40-80 (appr. -134)[381]		8[8]; 26[8]	1.8-3.8	[47]
Sibutramine[366]	appr. 0.001-0.01	0.11[8]		4-8	[8], [47]
Sildenafil	appr. 0.05-0.5	1[311]	6.2[8]	3-5	[8], [780], [1189]
Silver	-0.005[367] (-0.02[611])			48-52 days	[47], [58]
Simvastatin[544]	0.003-0.006	0.01[311]		2-4[290]	[48], [780], [1063]
Sirolimus (Rapamycin)	0.005-0.015[244]	0.015 (-0.06)		57-63	[654-657]
Sisomicin	0.5-10			appr. 1	[8], [1191]
Sitagliptin	0.05-0.38	0.72[311]; 3.8[8, 612]		8-14	[47], [780], [911], [1190]
Sodium aurothiomalate	see Gold				
Sodium nitroprusside	see Thiocyanate			0.1	[47]
Sodium oxybate (GHB)	see 4-Hydroxybutyrate				
Sodium valproate	see Valproic acid				
Sotalol[167]	0.5-3 (-4)	7.5-16[8]	36[8]; 40[8]; 43[8]	5-13 (-18)	[7, 8], [70], [658, 659]

Note on liability: The data and information have been extracted from scientific sources (primary and secondary literature) and have been carefully reviewed. Nevertheless, transcription errors cannot be excluded.
No responsibility is taken for the correctness of the information or data included. As a matter of principle, this compilation should be used with expert knowledge.

Substance	Blood-plasma concentration (mg/L) therapeutic ("normal")	toxic (from)	comatose-fatal (from)	t½ (h)	References
Sparteine[48]	0.5-1			2.5	[8]
Spiramycin	0.4-3			5-8	[3], [660]
Spiraprilate	0.006-0.045			33-41	[8]
Spironolactone[545]	0.05-0.25 (-0.5)[73]			13-24[73]	[8], [47], [58], [1064]
Stanozolol			0.056[8, 424]; 0.257[8, 425]		[940], [943]
Stiripentol	1-10	15[311]		4-13	[3, 4], [8]
Streptomycin	1-5 (15-40)	40		2-4	[8], [11], [58], [467]
Strontium	-0.075			30-60	[47]
Strychnine		0.075-0.1	(0.2-) 1.6[8]-4.7[8]	10-15	[8], [11], [661-667]
STS-135				3 min (in vitro)	[1224, 1225]
Sufentanil	(0.02-) 0.5-10 ng/mL[6]	-[339]	1-7 ng/mL[8]; 27 ng/mL[8]	1.6-6.3 (7-49)	[47], [54], [344], [346], [668-671], [1071]
Sulbactam	8-80			1-2[70]	[3], [47], [174]
Sulfamethoxazole	30-60[56]	400		9-12	[8], [672]
Sulfasalazine[34]	5-30	50	130[8]	4-10	[8]
Sulfisoxazole	90-100 (-210)			5-8	[48], [1192]
Sulfinpyrazone	6-17			3-5	[8]

Note on liability: The data and information have been extracted from scientific sources (primary and secondary literature) and have been carefully reviewed. Nevertheless, transcription errors cannot be excluded.
No responsibility is taken for the correctness of the information or data included. As a matter of principle, this compilation should be used with expert knowledge.

Substance	Blood-plasma concentration (mg/L)			t½ (h)	References
	therapeutic ("normal")	toxic (from)	comatose-fatal (from)		
Sulindac	1-5[102]	10[311]	130[8]	appr. 7	[87], [673], [780]
Sulpiride	0.05-0.4 (-1)[225]	1[311]; 5[8]	3.8-39[8]	4-14	[4], [43], [47], [58]
Sultiam (Sulthiame)	0.5-12.5 (2-8)	12-15	20-25	3-30	[8], [491], [677]
Sumatriptan	0.018-0.06	0.12[311]		1-4	[8], [47], [780]
Suramin	> 100[117]	300[118]		44-54	[8], [58], [674]
2,4,5-T	see 2,4,5-Trichloro-phenoxyacetic acid				
Tacrine	appr. 0.01	0.02		2-4	[8], [303]
Tacrolimus	(0.0005-) 0.005-0.015 (-0.02)	(0.015-) 0.02-0.025; 0.123[8]		9-16	[11], [191], [675-684]
Tadalafil	0.09-0.48	0.96[311]		16-19	[47], [780]
Talinolol	0.04-0.15		5[8, 129]; 20[8]	10-14 (-20)	[7, 8], [47], [685, 686]
Talipexole	appr. 0.0001-0.001			5-9	[8]
Tamoxifen	0.05-0.5			5-7 days	[3], [47]
Tamsulosin	0.003-0.022			5-16	[47], [780]
Tapentadol	0.01-0.13 (-0.3)[339]	-[339]	0.3[8, 613]; 1.1[8, 614]; 2[8]; 6.6[8]	3-7	[47], [680], [684]
Taxol	see Paclitaxel				

Note on liability: The data and information have been extracted from scientific sources (primary and secondary literature) and have been carefully reviewed. Nevertheless, transcription errors cannot be excluded.
No responsibility is taken for the correctness of the information or data included. As a matter of principle, this compilation should be used with expert knowledge.

Substance	Blood-plasma concentration (mg/L) therapeutic ("normal")	toxic (from)	comatose-fatal (from)	t½ (h)	References
Teicoplanin	(10-) 15-20 (-40)	200		10-15; 83-168[83]	[8], [58], [688], [913]
Telmisartan	0.006-0.225 (-1.2)			(18-) 21-25 (-30)	[47], [780], [1151]
Temazepam	0.02-0.15 (-0.9)	1	3.8[8]; 8.2[8]; 9[8]	6-25	[8], [58, 59], [689], [702]
Tenoxicam[174]	1.5-4 (-10)			(50-) 70-90	[8], [47], [690, 691], [1193]
Terazosin	0.02-0.08	0.16[311]		8-12	[8], [780]
Terbinafine	0.01-0.03[205]			20-36	[3], [10], [47], [58]
Terbutaline	0.001-0.006 (-0.03)		0.04	16-20[89]	[8], [58], [1195]
Terfenadine[368]	< 0.01	0.04-0.06[148]	0.4[8]	15-22[64]	[3], [47], [692]
Tetrachloroethylene	-		4-5; 22[8]; 44[8]; 66[8]	33-72	[47], [58], [87]
Tetracycline	1-5 (5-10)	30		6-10	[3], [8], [11]
Tetrahydrocannabinol (THC)	see Dronabinol				
Tetrazepam[40]	0.05-0.6 (-1)			(10-) 16-44	[8], [47], [58, 59]
Thalidomide	0.5-1.5 (-8)			5-9	[3], [8]
Thallium	-0.002 (-0.0006[331])	(0.003-) 0.1-0.5[179]; 5.6[8]	0.5-11	2-4 days	[3], [11], [47], [100], [693, 694]
Theobromine	(4-) 10-15	20		6-10	[11], [58]
Theophylline	(5-) 8-15 (-20)[82]	20	50	6-9[41]	[415], [695-701], [703-705]

Note on liability: The data and information have been extracted from scientific sources (primary and secondary literature) and have been carefully reviewed. Nevertheless, transcription errors cannot be excluded.
No responsibility is taken for the correctness of the information or data included. As a matter of principle, this compilation should be used with expert knowledge.

Substance	Blood-plasma concentration (mg/L) therapeutic ("normal")	toxic (from)	comatose-fatal (from)	t½ (h)	References
Thiamphenicol	0.5-3-10 (-15)	20		2-7	[8]
Thiamylal	appr. 5		29[8, 304]	0.6-0.8 (initial); 12-34 (terminal)	[47], [87]
Thiazinamium	0.05-0.15	0.3			[8], [11], [467], [1196]
Thiocyanate, from Nitroprusside	5-30[144]	35-100	200	3-4 days[83]	[8], [11], [255], [706], [1197, 1198]
Thiopental[57]	1-5	7	10-15[58]	3-8	[8], [48], [707], [1199]
Thioproperazine	appr. 0.001-0.02	0.1			[8, 9]
Thioridazine	0.1-0.2 (-2)[133]	0.4[311]	2.4[8]; 3-10	7-13 (-36)	[4], [8], [56]
Thiothixene	see Tiotixene				
Thyroxine	see Levothyroxine				
Tiagabine	0.02-0.2	0.3[311]; 0.5-0.6; 3.1[8, 245]		7-9	[3, 4], [8], [12], [381], [708-712]
Tianeptine	0.03-0.08	0.16[311]	5.1[8]	2.5-3	[4], [1200]
Tiapride	1-2	4		2-6	[8, 9], [47]
Tiaprofenic acid	appr. 15-40[193]			1.5-3 (-6)	[3], [713, 714]
Ticlopidine	< 1-2			70-130[100]	[47], [58]
Tiletamine			0.85[8]	1-4 (in animals)	[47], [87]

Note on liability: The data and information have been extracted from scientific sources (primary and secondary literature) and have been carefully reviewed. Nevertheless, transcription errors cannot be excluded. No responsibility is taken for the correctness of the information or data included. As a matter of principle, this compilation should be used with expert knowledge.

Substance	Blood-plasma concentration (mg/L) therapeutic ("normal")	toxic (from)	comatose-fatal (from)	t½ (h)	References
Tilidine[25]	0.05-0.3[339]	-[339]	1.7[8]	1-7	[9], [47], [715, 716]
Tiludronate (Tiludronic acid)	0.2-1.5 (-8)			65-78 (-150)	[49], [58], [717]
Timolol	0.005-0.05 (-0.1)			2-6	[8]
Tin	0.03-0.14				[58]
Tinidazol	10-50 (-60)			11-15 (-20)	[3], [47], [58]
Tiopronin	2-5			12-37	[58], [718], [1201]
Tiotixene (Thiothixene)	0.001-0.02 (0.01-0.1)	0.1		12-36	[47, 48], [58], [185]
Tiotropium	16 ng/L[307]			5-6 days	[719]
Tipranavir (TPV)	> 20.5[299]			5.5-6	[2], [110, 111]
Tizanidine	0.005 -0.045	0.09[311]		2-4	[3], [47], [780]
Tobramycin	< 2- (5-10)[154]	12-15		0.5-3	[3], [67], [198], [878]
Tocainide	4-12 (6-10)	13-15; 20[8]	74[8]; 78[8]; 140[8]	8-25	[3], [8], [47], [58], [720, 721]
Tofenacin(e)	0.025-0.1	0.5-1			[9], [58]
Tolbutamide	45-100	(120-) 400-500	640[8]	4-12	[8], [58], [722]
Tolcapone	3-6[615]	12[311]		2	[4]
Tolmetin	10-80	160[311]		2-4	[58], [780]

Note on liability: The data and information have been extracted from scientific sources (primary and secondary literature) and have been carefully reviewed. Nevertheless, transcription errors cannot be excluded.
No responsibility is taken for the correctness of the information or data included. As a matter of principle, this compilation should be used with expert knowledge.

Substance	Blood-plasma concentration (mg/L) therapeutic ("normal")	toxic (from)	comatose-fatal (from)	t½ (h)	References
Tolperisone	0.09-0.3	0.7[8, 382]	7-14	1.8-2.9	[47]
Tolterodine[48, 492]	0.0008-0.002 (-0.005[493])			2-3	[47], [780], [804], [1030]
Toluene			10 (-48[8, 254])	13-68[254]	[47], [58]
Topiramate	2-10[218]	16[311]	49[8]	18-30	[4], [8], [381], [712], [1202]
Topotecan	appr. 0.001-0.01[190]			2-3	[3], [723, 724]
Torasemide (Torsemide)	0.064-0.52 (-2)			2-6	[47], [780], [1206-1208]
Tramadol[48, 584]	0.1-1 (> 0.3)[87, 339]	1[339]	2[8, 49]; 5.2[8]; 5.3[8, 404]; 9.4[8, 551]; 13[8]; 38.3[8, 252]	5-10	[11], [63], [244], [725, 726], [902], [1067, 1068], [1123-1125], [1231]
Trandolapril[619]	0.43-3 ng/mL[619, 620]		58 ng/mL[8]	0.6-0.8[619]	[47]
Tranexamic acid	10-50			1-3 (-10)	[8], [47]
Tranylcypromine	< 0.05	0.1[311]; 0.5[8, 202]	0.7[8]; 5[8]	1-3.5	[4], [727]
Trapidil	(4-) 6-10			2-6	[8], [95], [728]
Trazodone[145]	0.7-1 (-2)	1.2[311]; 3-4	9; 12-15[8]	4-11 (-13)	[4], [43], [58], [64], [263], [729]
Trenbolone			0.163[8, 425]		[943]
Triamterene	0.01-0.1 (-0.2)	0.2[311]		1.5-4 (-6)	[8], [42], [780]
Triazolam	0.002-0.02	0.04[311]	0.03[8]	(1-) 2-5	[4], [58], [423], [730, 731]

Note on liability: The data and information have been extracted from scientific sources (primary and secondary literature) and have been carefully reviewed. Nevertheless, transcription errors cannot be excluded.
No responsibility is taken for the correctness of the information or data included. As a matter of principle, this compilation should be used with expert knowledge.

Substance	Blood-plasma concentration (mg/L) therapeutic ("normal")	toxic (from)	comatose-fatal (from)	t½ (h)	References
2,2,2-Tribromoethanol		50	90		[58]
1,1,1-Trichloroethane			(15[8]); 100-1000	20-26[622]	[47], [58]
2,2,2-Trichloroethanol[369]	5-15	40-70	60-100	6-10[327]	[47], [58]
Trichloroethylene			9.7[8]; 16[8]; 21[8]	30-38[623]	[47], [87]
2,4,5-Trichlorophenoxyacetic acid (2,4,5-T)	-	appr. 100	200	11-23 (-33)	[3], [11], [47]
Trifluoperazine	0.001-0.01 (-0.05)	0.1-0.2	0.4[8]	7-18	[8], [87]
Triflupromazine	0.03-0.1	0.3-0.5		appr. 6	[8]
Trihexyphenidyl (Trihex)	0.05-0.2[75]	0.5	0.12[8]	(3-5) 24-41	[9], [47], [114], [1215]
Trimeprazine	see Alimemazine				
Trimethadione[274]	20-40			16	[58]
Trimethobenzamide	1-2		184[8]	7-9	[11], [47], [58]
Trimethoprim	1.5-2.5[56]	20		8-11	[8], [672]
Trimipramine[566]	(0.01-) 0.15-0.3	0.6[311]	1.7-8.2[251]	10-20 (-40)	[4], [56], [223], [1084, 1085]
Tripelenamine	0.02-0.06		10[8]	5-8	[8], [11], [57], [87]
Triprolidine	0.004-0.045			1-5	[47], [57]
Tropisetron[48]	(0.003-) 0.02-0.05 (-0.08)			7-9 (-30)	[8], [47], [66]

Note on liability: The data and information have been extracted from scientific sources (primary and secondary literature) and have been carefully reviewed. Nevertheless, transcription errors cannot be excluded.
No responsibility is taken for the correctness of the information or data included. As a matter of principle, this compilation should be used with expert knowledge.

Substance	Blood-plasma concentration (mg/L) therapeutic ("normal")	toxic (from)	comatose-fatal (from)	t½ (h)	References
Tubocurarine	(0.04-) 0.4-3 (-6)			2-4	[8], [48], [58], [1216]
Tungsten	-0.035		5[8]	2-4	[47], [58]
U-47700[394]	-	0.24[8, 399]; 0.394[8]	0.017-0.49; 0.32[8]; 0.37[8, 400]; 0.53[8]; 0.82[8]; 1.1[8]; 1.52[8]; 3.04[8]	appr. 6	[837], [914-920], [1071]
Uranium	0.04 ng/mL[331]		0.77[8, 624]	450 days	[47], [100]
Urapidil	appr. 0.1-0.2			2-3 (-7)	[3], [58], [95]
Valdetamide	see Diethylpentenamide				
Valnoctamide	5-6 (-25)	40		7-14	[58], [1217]
Valproic acid	(40-) 50-100 (-150)	120[311]; 150-200	556[8]; 720[8]	7-17 (-20)	[4], [47], [64], [161], [164]
Valsartan	appr. 0.8-6	12[311]		5-12 (-14)	[8], [780], [909]
Vanadium	-0.05	0.0058[8]	6.2[8]	4-12 days	[47]
Vancomycin	10-20[142]	30 (-40)		3.6-11[83]	[47], [67], [390], [732-736], [823], [878]
Vardenafil	0.002-0.004 (-0.04)			2-5	[47], [780]
Varenicline	0.004-0.005	0.01[311]	0.26[8]	23-39	[4], [1218]
Vecuronium[624]	appr. 0.2-0.37 (-0.5)[624]		1.2[8]	1-1.5	[8, 9], [47], [1219]
Vedolizumab	> 14-16			appr. 25-26 days	[921]

Note on liability: The data and information have been extracted from scientific sources (primary and secondary literature) and have been carefully reviewed. Nevertheless, transcription errors cannot be excluded.
No responsibility is taken for the correctness of the information or data included. As a matter of principle, this compilation should be used with expert knowledge.

Substance	Blood-plasma concentration (mg/L) therapeutic ("normal")	toxic (from)	comatose-fatal (from)	t½ (h)	References
Venlafaxine[567]	(0.06-) 0.1-0.4[189]	0.8[311]; 1-1.5[266]	3.2-24[8]	3-7 (-14)[625]	[4], [11], [101], [223], [737], [807], [1084, 1085]
Verapamil[90]	(0.01-) 0.02-0.25 (-0.4)	1	2.5; 0.9[8]; 3.9[8]; 85[8]	6-14[42]	[8], [42], [56], [64], [84], [721], [738, 739], [870], [922]
Vigabatrin	2-10 (-15)[94]	20[311]		5-8	[4], [47], [381]
Vilazodone	0.03-0.07	0.14[311]		18-32	[4]
Vildagliptin	0.18-0.3 (-1.6)			2-3	[911], [923, 924]
Viloxazine	1-4 (-8?)	47[8]; 60[8]	40[8]; 45[8]	2-5	[8], [43], [47]
Vincamine	< 0.25			1-2	[8], [47]
Vinylbital	1-3	5	8	18-33	[8]
Viquidil	0.15-0.25			6-12	[11]
Vitamin A	see Retinol				
Vitamin B_6	see Pyridoxine				
Vitamin B_{12}	(200-) 300-500 (-750) pg/mL[390]			21-29	[800], [944]
Vitamin C	see Ascorbic acid				
Vitamin D	(0.02-) 0.03-0.09[263]	0.2; 0.46[8]		appr. 30 days	[740-743], [800], [925]

Note on liability: The data and information have been extracted from scientific sources (primary and secondary literature) and have been carefully reviewed. Nevertheless, transcription errors cannot be excluded.
No responsibility is taken for the correctness of the information or data included. As a matter of principle, this compilation should be used with expert knowledge.

Substance	Blood-plasma concentration (mg/L)			t½ (h)	References
	therapeutic ("normal")	toxic (from)	comatose-fatal (from)		
Voriconazole	1-6	3.5 (-6.0)[284]		4-10[37]	[47], [603], [744, 745], [895], [1186]
Vortioxetine	0.006-0.04 (-0.07)	0.08[311]		57-66	[4], [780], [1194]
Warfarin	1-3 (-7)[602]	10-12	100	37-50[97]	[3], [14], [42], [746]
Wismut	see Bismut				
Xamoterol	appr. 0.02-0.05 (-0.2)			13-30	[58], [1210-1213], [1220]
Xipamide	3-11 (-20)			5-8	[47], [1209]
Xylene	< 0.002[616]		3-40	20-30	[47], [58]
Yohimbine[546]	appr. 0.05-0.3		5.2[8]; 5.4[8]; 7.4[8]; 8[8, 547]	1-3	[8], [47], [1065], [1221]
Zafirlukast	0.005-0.03 (0.7)			5-20	[8], [47]
Zalcitabine	appr. 0.1 (0.5 µmol/L)			1-3[83]	[38], [290]
Zaleplon(e)	(0.001-) 0.02-0.04 (-0.1)	0.2[311]		1-2	[4], [8], [935]
Zanoterone	0.1-0.5				[747]
Zidovudine	0.1-0.3 (-1)[51]	2-3; 24[8]		0.5-3	[2], [38], [47], [290], [748-750]
Zinc	0.6-1.3[626]	2	42[8]	5-16 months[627]	[47], [66], [1222]
Zipeprol	0.1-0.7		2.3-31[8]	1.2	[47], [57]
Ziprasidone	(0.02-) 0.05-0.2	0.4[311]		2-8	[4], [8]

Note on liability: The data and information have been extracted from scientific sources (primary and secondary literature) and have been carefully reviewed. Nevertheless, transcription errors cannot be excluded. No responsibility is taken for the correctness of the information or data included. As a matter of principle, this compilation should be used with expert knowledge.

Substance	Blood-plasma concentration (mg/L) therapeutic ("normal")	toxic (from)	comatose-fatal (from)	t½ (h)	References
Zoledronat (Zoledronic acid)	0.22-2.2[628]			1-189 days	[47], [49], [751-753]
Zolmitriptan	0.003-0.01			1.6-3.8	[8], [47]
Zolpidem	0.08-0.16[629] (-0.2)	0.32[311]; 0.5	1.5-4	1-5	[4], [8], [11], [310], [754-756], [935], [1231]
Zomepirac	0.1-4		152[8]	4-10	[8], [87]
Zonisamide	(10-) 20-30 (-40)	40-70	100[208]	50-70[209]	[8], [47], [381], [712], [757, 758]
Zopiclone	(0.01-) 0.055-0.085[630] (-0.12)	0.15; 0.3[311]	0.6-1.8	2-8	[4], [11], [15], [56], [935]
Zotepine	0.01-0.15	0.3[311]		13-16	[4]
Zuclopenthixol[48, 365]	0.004-0.05 (-0.1)	0.1[311]		15-25	[4], [15], [1223]

Drug concentrations are mentioned as mg/L (= µg/mL), if not otherwise stated.

To convert drug concentrations to or from SI units, use the following formulas (MW = molecular weight):
To determine the conversion factor: $\mathrm{CF} = \frac{1000}{\mathrm{MW}}$

To convert *to* SI units: mg/L (= µg/mL) x CF = µmol/L
To convert *from* SI units: µmol/L ÷ CF = mg/L (= µg/mL)

Note on liability: The data and information have been extracted from scientific sources (primary and secondary literature) and have been carefully reviewed. Nevertheless, transcription errors cannot be excluded. No responsibility is taken for the correctness of the information or data included. As a matter of principle, this compilation should be used with expert knowledge.

Example carbamazepine (MW: 236.27): CF = $\frac{1000}{236.27}$ = 4.23

Conversion:
mg/L (µg/mL) → µmol/L: (8 mg/L) x 4.23 = 33.8 µmol/L

µmol/L → mg/L (µg/mL) : (33.8 µmol/L) ÷ 4.23 = 8 mg/L (= 8 µg/mL)

Clinical categories used for grouping analytical data

Therapeutic: blood-plasma/serum concentrations (in general, trough at steady state) observed following therapeutically effective doses; no or only minimal side effects (drugs); “normal”: concentrations associated with no or only minimal toxic effects (other xenobiotics, including ‘recreational’ use).

Toxic: blood-plasma/serum concentrations which produce toxicity/clinically relevant side effects/symptoms.

Comatose-fatal: blood-plasma/serum (comatose) concentrations and whole blood (fatal) concentrations reported to have caused coma and death, respectively. Whether published data for deaths refer to levels measured ante-mortem or post-mortem (femoral or heart blood) is often unknown.

In addition to specific references provided in the table, data were compared and contrasted against database sources, published review articles and textbooks [3, 4, 7-15, 41-43, 47, 48, 56-59, 64, 66, 74, 87, 88, 259, 270, 294, 467, 759-780, 878, among others] but not specifically indicated for every drug/substance, as well as supplemented with our experiences in clinical and forensic toxicology.

Note on liability: The data and information have been extracted from scientific sources (primary and secondary literature) and have been carefully reviewed. Nevertheless, transcription errors cannot be excluded.
No responsibility is taken for the correctness of the information or data included. As a matter of principle, this compilation should be used with expert knowledge.

Meaning of brackets e.g., therapeutic plasma concentration = (0.02-) 0.08-0.25 or $t_{½}$ = (4-) 8-22 (-35): These brackets may have slightly different meanings and depend on the substance. For example for antibiotics such as amoxicillin or ampicillin, they refer to trough and peak concentrations, respectively. In most cases, however, the concentrations in brackets refer either to (published) data mentioning a wider boundary of therapeutic (or toxic) concentration ranges but the majority of sources do not include this range or a concentration resulting from (very) low or (unusual) high doses.

This may also include different doses used in different indications (e.g., diuretics, betablockers, benzodiazepines, oral anticoagulants), acute vs. chronic application, very old or very young patients, patients with very low body weight/mass, or even slow and fast /extensive metabolizers. Sometimes (e.g., opioids), this reflect tolerability developed – that is, chronic opioid users may tolerate very high doses compared to opioid-naïve persons. Hence for opioid-naïve persons, a small dose (leading to a low blood/CNS concentration) might be deleterious.

As an example, for lorazepam [(0.02-) 0.08-0.25], the most cited and used therapeutic range is 0.08-0.25 mg/L. However, there is evidence that even lower concentrations may be measured after therapeutic low doses.

So, if not specifically mentioned or annotated, the data in brackets widen the range but do not cover most of the cases.

Note on liability: The data and information have been extracted from scientific sources (primary and secondary literature) and have been carefully reviewed. Nevertheless, transcription errors cannot be excluded.
No responsibility is taken for the correctness of the information or data included. As a matter of principle, this compilation should be used with expert knowledge.

Annotations

1 active metabolites of acebutolol: N-acetylacebutolol ($t_{1/2}$: 9–14 h): therapeutic concentration 1–2.5 mg/L, comatose-fatal from appr. 90 mg/L, and diacetolol ($t_{1/2}$: 8–13 h): therapeutic 0.65–4.5 mg/L, comatose-fatal from appr. 100 mg/L

2 as salicylic acid (for analgesic and antipyretic effects)

3 as digoxin

4 typical therapeutic serum concentrations of fentanyl are 0.0003–0.0012 mg/L after application of a 25 mg/h transdermal patch; high concentrations during surgery and mechanical ventilation

5 slow (poor) and rapid (extensive) acetylators (metabolizers)

6 during mechanical ventilation

7 active metabolites nortriptyline (see Table) and amitriptyline oxide ($t_{1/2}$: 1.5–3 h)

8 case report

9 in patients with impaired renal function in some cases up to 100 h

10 active metabolite 6-mercaptopurine ($t_{1/2}$: 1–1.5 h)

11 appr. 0.2 h for azathioprine

12 active metabolite carbamazepine-10,11-epoxide ($t_{1/2}$: 5–16 h; usual plasma concentration range 0.2–2 mg/L) should be considered in case of intoxication

13 each sum carbromal(um) + carbromide ($t_{1/2}$: 12–15 days)

14 each as trichloroethanol

15 active metabolite N-desmethyldiazepam = nordazepam (see Table)

16 nephrotoxic

17 active metabolite N-desmethylclobazam (therapeutic reference range: 0.3–3 mg/L; laboratory alert level[311] 5 mg/L)

18 duration of pharmacological effects: 0.3–0.4 h; major metabolite: benzoylecgonine ($t_{1/2}$: 5–6 h)

19 active metabolites nordazepam and oxazepam (see Table)

20 active metabolite nordothiepin ($t_{1/2}$: 20–60 h)

21 active metabolite desmethyldoxepin (= nordoxepin, $t_{1/2}$: 33–80 h) should be considered in case of intoxication

22 benzodiazepine antagonist

23 active metabolites

24 active metabolite desalkylflurazepam ($t_{1/2}$: 74 ± 24 h)

25 active metabolite nortilidine ($t_{1/2}$: 6 h), comatose-fatal plasma concentration: 4.4 mg/L[8]

26 in some cases up to 80 h

27 active metabolite levomepromazine sulfoxide ($t_{1/2}$: 5–10 h)

28 therapeutic concentration range 3-O-methyldopa 0.7–10.9 mg/L

29 active metabolite desipramine (see Table)

30 active metabolites diazepam, nordazepam, and temazepam (see Table) with longer $t_{1/2}$

31 active metabolite of mesuximide; the metabolite is the active compound in vivo

32 sum of active metabolites

33 each as desacetylmetipranolol

34 active metabolite 5-aminosalicylic acid (mesalazine, see Table); rapid/slow acetylators of the primary metabolite sulfapyridine

35 in some cases longer

36 active metabolite oxyphenbutazone ($t_{1/2}$: 27–64 h)

37 dose dependent and depending on the duration of therapy; the drug stimulates its own metabolism

38 active metabolite of procainamide

39 for the management of osteoporosis

40 active metabolites diazepam (see Table), nordiazepam (see Table), and nortetrazepam ($t_{1/2}$: 25–51 h)

41 smokers: $t_{1/2}$: 3–6 h

42 for astemizole plus desmethylastemizole during steady state appr. 20 days

43 active metabolite dehydroaripiprazole

44 blood drug concentrations following therapeutically effective doses below detection limit

45 as decanoate ($t_{1/2}$: 5–12 days)

46 in intensive care patients in some cases $t_{1/2}$ 8–22 h

47 physiologic, total serum (TT4; protein bound and non-protein bound); free T4 (fT4): 8–21 ng/L

48 rapid (extensive) and slow (poor) metabolizers (genetic polymorphism)

49 6 month-old-child, appr. 15 h after 100 mg tramadol rectally

50 active metabolites noracetylmethadol and dinoracetylmethadol

51 C_{max} 0.038 ± 0.006 mg/L after a single oral dose of 150 µg/kg in nine patients with onchocerciasis ($t_{1/2}$: 56 ± 7 h)

52 as enalaprilat

53 duration of clinical effect: 3–5 h

54 product after hydrolysis

55 narcotic; analyzed during the distribution phase

56 for pneumocystis carinii pneumonia (PcP) treatment: sulfamethoxazole 100–200 mg/L, trimethoprim 5–10 mg/L

57 metabolite: pentobarbital (see Table)

58 “narcotic”

59 higher with meningism (-25 mg/L); decreased protein binding in neonates results in increased unbound drug

60 each as 2-hydroxyflutamide (active and major metabolite)

61 active metabolite of allopurinol

62 active metabolite paracetamol (synonym acetaminophen, see Table)

63 active metabolites phenobarbital (see Table) and phenylethylmalonide (7-10 mg/L; $t_{1/2}$: 16–50 h)

64 as active carboxylic acid metabolite = fexofenadine

65 1 mg oral alprazolam/day equals appr. a plasma concentration of 0.01 mg alprazolam/L during steady state; usually, higher doses/plasma concentrations are recommended for the treatment of phobias when compared to panic disorders/attacks

66 highly inter- and intraindividual variable kinetics; for children (therapeutically): 0.04–0.1 mg/L; active metabolite desmethylchlorpromazine

67 ≥ 0.25 μmol/L desirable for echinococcosis

68 mean: 27 h; for geriatric patients (> 65 years) in some cases increased to more than 90 h

69 active metabolite 2-hydroxydesipramine ($t_{1/2}$: mean 18 h; in patients with impaired renal function several-fold increased)

70 in patients with impaired renal function several-fold increased

71 in colon tissue 0.8–1.8 h after 1 x 2 g i.v.: 94.0–7.4 μg/g

72 active metabolites 1-(5-hydroxyhexyl)-3,7-dimethylxanthine and 1-(3-carboxypropyl)-3,7-dimethylxanthine ($t_{1/2}$: 1–1.6 h), among others, with 5 and 8 times, respectively, higher plasma levels than pentoxifylline

73 as canrenone, one of the active metabolites of spironolactone ($t_{1/2}$ spironolactone: 1–4 h)

74 appr. 8 h after ingestion of probably 210 mg haloperidol and 1400 mg orphenadrine-HCl with life-threatening arrhythmias

75 data on effective plasma concentrations for Parkinson's disease not proven

76 once or twice daily regimen: peak: 15–40 mg/L, trough: 1–5 (-7) mg/L; individually very variable

77 for hypertension: 0.2–0.45 mg/L; for coronary heart disease or arrhythmias: 0.3–0.8 mg/L

78 therapeutic concentration of the unbound fraction: 0.5–2 mg/L; therapeutic concentration of the metabolite desalkyldisopyramide < 5 mg/L (ratio of metabolite to the parent compound disopyramide guide to duration of therapy and possibly to the likelihood of toxicity)

79 in mmol/L (mEq/L, mval/L): 0.4–1.2 (0.6–1.4) mmol/L, toxic from 1.5 mmol/L; conversion factor: mmol/L x 6.93 = mg/L (mmol/L x 0.693 = mg/dL)

80 terminal elimination $t_{1/2}$: 37 ± 6 h, increased in case of renal dysfunction

81 therapeutic concentration of the unbound fraction: 1–2.2 mg/L

82 for (sleep) apnea: 5–10 mg/L

83 increased in patients with impaired renal function

84 C_{max} 3–5 h after 4 mg loperamide hydrochloride orally: 1–3 ng/mL

85 active metabolite N-desmethylclomipramine ($t_{1/2}$: (21-) 37–43 (-65) h, mean: 40 h)

86 after a first dose in a group of patients given 1.5–2.5 mg/kg colistin methate, peak and trough serum colistin levels averaged 21 and 2.8 mg/L, respectively

87 post-operative (on-demand; i.v.): 0.02–1–2 mg/L (median: 0.29–0.92 mg/L) as minimal (analgesic) effective concentration; O-desmethyltramadol: 0.03–0.04 mg/L (median: 0.036 mg/L)

88 10–36 h

89 11–26 h

90 stereoselective metabolism; therapeutic concentration after oral application higher than after intravenous administration

91 $t_{1/2}$ for ß-phase of the elimination: 0.5–1 h

92 as albendazole sulfoxide (active metabolite)

93 $t_{1/2}$ in slow (poor) metabolizers appr. 40 h

94 trough plasma concentration at steady state during 2 g twice daily orally appr. 9 mg/L; C_{max} (0.8 h after 1 g p.o.): appr. 45 mg/L

95 as active metabolite methimazole (thiamazole)

96 mean 80 min

97 15–85 h

98 plasma concentrations do not correspond with pharmacological effects

99 naltrexone plus 6-ß-naltrexone: 0.025–0.1 mg/L; plasma concentrations of the less potent major metabolite 6-ß-naltrexol ($t_{1/2}$: 11–13 h) are usually 1.5–10 times higher

100 at steady state; 4–15 h after a single dose

101 sum rifampicin plus metabolites

102 sum sulindac plus metabolites (sulindac sulfide, $t_{1/2}$: 15–18 h; $t_{1/2}$ sulindac sulfone: 17–20 h)

103 abuse

104 sum carisoprodol plus meprobamate

105 12–15 days for the metabolites

106 $t_{1/2}$ for total platinum plasma concentrations: 20–40 h (up to 6–7 days)

107 2–20 µmol/L

108 (non-active) carboxylic acid metabolite ($t_{1/2}$: appr. 20 days): 1.5–5.5 mg/L

109 during concomitant therapy with carbamazepine or phenytoin mean 13.5–15 (range 8–33) h, during concomitant therapy with valproic acid 48–59 (range 31–89) h

110 in infants and after intoxications in some cases dramatically increased to appr. 6–14 days

111 during steady state 3–4 h after oral doses of 100–400 mg; prophylaxis of candidiasis: > 0.2 mg/L and of aspergillosis: > 1.0 mg/L in patients with acute myeloid leukemia

112 plasma concentrations of the major metabolite 13-cis-acitretin are usually higher

113 higher and increased, respectively, in patients with impaired hepatic function; for tinnitus aurium therapeutic plasma concentration appr. 1–2 mg/L

114 biologically active/major metabolite cycloguanil ($t_{1/2}$: 8–17 h): plasma concentration after daily oral doses of 100–200 mg proguanil appr. 0.02–0.06 mg/L

115 active metabolite norpethidine ($t_{1/2}$: 14–24 (-48) h) with higher toxicity than pethidine; toxic from appr. 0.5 mg/L

116 main metabolite N-desmethylrosiglitazone with appr. 20-fold lower potency

117 as cytostatic drug: > 200 mg/L

118 neurotoxic

119 in terminal renal insufficiency appr. 0.5–2 mg/L, cumulation of the inactive metabolite N-acetyl-5-aminosalicylic acid (Ac-5-ASA) up to 20 mg/L without adverse effects

120 $t_{1/2}$ of the inactive major metabolite N-acetyl-5-aminosalicylic acid (Ac-5-ASA) appr. 6–9 h

121 tocolytic (4.5–6.25 mval(mEq)/L = 2.25–3.125 mmol/L). Approximate normal range: 18–25 µg Mg^{2+}/mL (1.8–2.5 mg/dL, 0.74–1.03 mmol/L); conversion factor: mg/dL x 0.4113 = mmol/L

122 C_{max} appr. 2–8 µmol/L (i.e. 1.7–6.8 mg/L, after 135–390 mg/m^2 intravenously for 3 h); much lower after intraperitoneal injection

123 as transdermal system (patch); plasma concentrations of the major metabolite cotinine ($t_{1/2}$: mean 16–20 h) appr. 10 times higher

124 mean 2 h; after application of the transdermal system possibly longer

125 active metabolites desipramine (see Table), 2-hydroxyimipramine ($t_{1/2}$: 6–18 h), and 2-hydroxydesipramine[69]

126 3–7 min after retrobulbar blockade: 0.5–1.1 mg/L

127 for myasthenia gravis

128 half maximal effective concentration (EC_{50}) for analgesia: 0.0088 ± 0.0053 mg/L; EC_{50} for respiratory depression: 0.035 ± 0.022 mg/L

129 appr. 14 h after oral ingestion of 1.5 g and hemoperfusion

130 fluoxetine plus norfluoxetine; $t_{1/2}$ of the active metabolite N-desmethylfluoxetine (= norfluoxetine): 4–16, mean 7–9 days

131 active metabolites 2-oxoquazepam ($t_{1/2}$: 39 (28–43) h) and N-desalkyl-2-oxoquazepam (N-desalkylflurazepam, $t_{1/2}$: 74 ± 24 h)

132 peak plasma concentration during steady state

133 range of plasma concentrations after therapeutically effective doses of thioridazine for the active metabolites mesoridazine (thioridazine-2 sulfoxide): 0.2–1.6 mg/L ($t_{1/2}$: 10–14 h) and sulforidazine (thioridazine-2 sulfone): up to 0.6 mg/L ($t_{1/2}$: 10–16 h) and for the inactive metabolite thioridazine-5(ring) sulfoxide: 0.06–4 mg/L; probably, the best correlation exists between the plasma concentration of mesoridazine and the clinical response

134 usually sleep occurred with ≥ 0.1 mg/L; in infants and children (< 13 years): in some cases during mechanical ventilation up to 3 mg/L; α-

hydroxymidazolam-glucuronide likely contributes in case of impaired renal function to prolonged sedation

135 plasma concentration range of the primary metabolite 1,5-dimethyl-3,3-diphenyl-2-ethylidene-pyrrolidine (EDDP) during steady state: 0.005–0.055 mg/L (daily oral methadone dose: 10–225 mg, mean 60 mg)

136 ratio clozapine/active metabolite N-desmethylclozapine (= norclozapine, $t_{1/2}$: 19.2 ± 10.2 h) usually 1.0–2.5

137 maximum antiemetic effect at > 0.01 mg/L

138 active metabolite descarboethoxyloratadine (desloratadine, $t_{1/2}$: 17–24 h): appr. 0.005–0.02 mg/L

139 0.15 ± 0.05 ‰ per h

140 during chronic administration appr. 10–20 h (induction of own metabolism)

141 caution is warranted in case of concomitant use or intoxication with serotonin reuptake inhibitors (SSRI) such as citalopram, clomipramine, fluoxetine, or paroxetine: possible serotonin syndrome

142 trough concentrations of 15–20 mg/L for complicated infections/sepsis as a surrogate (when the MIC is 1 mg/L or less) for the target 24-hour area under the concentration-time curve (AUC_{24}) to a minimum inhibitory concentration (MIC) ratio of ≥ 400; peak concentration: < 40 mg/L

143 distribution half-life: 0.3–0.5 (-1) h

144 non-smoker: 1–4 mg/L (17–69 µmol/L); smoker: 3–12 mg/L (52–206 µmol/L)

145 major active metabolite 1-m-chlorophenylpiperazine (mCPP); plasma concentration appr. 1/10 compared to trazodone

146 plasma concentration (15–20 µmol/L) for maximal cellular accumulation of the active form gemcitabine-5'-triphosphate

147 after nasal or oral application

148 *Torsade de Pointes*, usually due to cytochrome P450 3A4 inhibition (e.g., ketoconazole, erythromycin) and/or impaired hepatic function

149 after oral administration; after topical application: plasma concentration < 0.03 mg/L and $t_{1/2}$ appr. 22 h

150 for each added 1 mg/day dose of clonazepam, there is appr. an increase of 12 ng/mL in the plasma (patients with panic disorder)

151 sum of amoxapine and his major metabolite 8-hydroxyamoxapine ($t_{1/2}$: appr. 30 h; $t_{1/2}$ 7-hydroxyamoxapine: 4–6.5 h)

152 sum of bupropion (amfebutamone) and morpholinole metabolite ($t_{1/2}$: 19–22 h)

153 after i.m.-application as decanoate appr. 3 weeks

154 C_{min} < 1–2 mg/L at best (especially in patients with renal dysfunction)

155 appr. 0.02 mg/L in organophosphorous ester poisoning depending on clinical symptoms

156 in case of organophosphorous ester (e.g. parathione) intoxication; 250 mg intravenously as bolus followed by an infusion of 750 mg/24 h

157 if used as an antiarrhythmic appr. 0.1–0.4 mg/L

158 active metabolite roflumilast N-oxide

159 extensive metabolizers; $t_{1/2}$ for poor metabolizers: 15-20 h; $t_{1/2}$ for risperidone plus 9-hydroxyrisperidone: 22–24 h

160 6 case reports: post-mortem 5.2–49 µg citalopram/g blood and 0.3–1.4 µg desmethylcitalopram/g blood

161 concentration/dose-values for extensive metabolizers: 0.025–0.688 (median 0.098) nmol/L per mg oral perphenazine, and 0.096–0.750 (median 0.195) nmol/L per mg oral perphenazine (mol wt 506.07) for poor metabolizers, respectively

162 two cases after ingestion of appr. 4 g moclobemide in combination with clomipramine (plasma concentration: 0.3–0.5 mg/L, i.e. toxic)

163 as R-enantiomer, mean: 9 mg/L

164 dosage: 50–55 mg/kg per day

165 appr. 2.5 h after ingestion of 50–100 mg amlodipine besylate with alcohol (263 mmol ethanol/L)

166 0.101 mg/L 4 h after ingestion of 70 mg and 0.185 mg/L at 10.5 h, complicated by oxazepam ingestion

167 data for d,l-sotalol

168 after i.v.-application; $t_{1/2}$: 4–7 h following epidural administration (appr. 4–5 h following intercostal block and appr. 6–8 h following brachial plexus blockade, respectively)

169 mean 19 h; $t_{1/2}$ of oral ciclosporine microemulsion is appr. 8 h

170 a longer $t_{1/2}$, up to 3.8 days, has been reported in elderly patients

171 target range of activated partial thromboplastin time (aPTT) is prolongation of 50–70 sec; aPTT prolongation of more than 100 seconds has been associated with an increased risk of hemorrhagic events

172 as 10-hydroxycarbazepine for seizures (0.4–2 mg/L for oxcarbazepine); in patients with trigeminal neuralgia, therapeutic target range of the active metabolite 10-hydroxycarbazepine ($t_{1/2}$: 7–14 (-20) h): 50–110 µmol/L (appr. 13–28 mg/L)

173 mild CNS symptoms (limited data)

174 pharmacologically inactive metabolites 5'- and 6'-hydroxytenoxicam

175 effective plasma concentrations for the 2 active metabolites: O-desmethylencainide (0.05–0.3 mg/L; toxic from 0.3 mg/L, $t_{1/2}$: 11 h) and 3-methoxy-o-desmethylencainide (0.06–0.28 mg/L; $t_{1/2}$: > 24 h) during long-term therapy

176 in poor metabolizers 9–11 h

177 "normal": 0.001–0.006; smoker: 0.005–0.012 (-0.15) mg/L; µmol/L x 0.026 = mg/L

178 reference value; 0.001 µg/g creatinine or 0.0014 mg/L urine; < 30 µg/24 h urine ("normal"); "toxic" from appr. 0.05–0.3 mg/L urine. Reference value for children in Germany: 0.0008 mg/L blood and 0.0004 mg/L urine

179 > 0.04 mg/L urine

180 up to (appr. 5–25) years in chronically exposed workers

181 combination with 2,4-D and chlorpyrifos

182 urine pH-dependent; in case of intoxication/overdose: 70–90 h

183 overdose

184 one case of toxicokinetic estimation in acute potassium cyanide (KCN) poisoning

185 dependent on indication; > 2.0 mg/L for partiell seizures; tentative target range according to Neels HM et al. 2004 [12]: 12–20 mg/L; peak concentration at steady state appr. 4.6 mg/L (300 mg three times daily (tid)) and appr. 8.4 mg/L (600 mg tid)

186 prolonged in case of impaired renal function to 16–43 h; > 100 h in dialysis dependent patients

187 dependent on urine pH, if alkaline appr. 8–10 h

188 females showed significantly longer elimination half-lives (35.4 ± 13.7 h) than males (21 ± 5 h); the $t_{1/2}$ of the R(-)-enantiomer is twice that of the S(+)-enantiomer

189 venlafaxine plus O-desmethylvenlafaxine. After doses of 25, 75, and 150 mg every 8 h for three days, mean peak serum levels were 0.053, 0.167, and 0.393 mg/L; corresponding levels of the major active metabolite O-desmethylvenlafaxine ($t_{1/2}$: 10–11 h) were 0.148, 0.397, and 0.686 mg/L

190 at least 10 nmol of the lactone (mol wt 421.46)/L; decreases in absolute neutrophil counts of 50–90% were observed with steady state plasma concentrations of total topotecan (lactone + hydroxy acid) of 20–60 nmol/L, respectively

191 a mean steady state peak plasma concentration of 0.286 mg/L was observed in healthy volunteers after 60 mg (oral solution) every 12 h for 10 doses

192 the metabolite 2',2'-difluorodeoxyuridine (dFdU) has minimal antitumor activity but may contribute to the toxicity of gemcitabine

193 C_{max} after 200 mg three times daily

194 serum concentration of benzoic acid following high dose diazepam i.v.-infusion and severe metabolic acidosis (5-year-old girl; urine concentration: 1,200 mg/L)

195 1.5 h in dogs after i.v.-administration

196 for erythropoietic protoporphyria (EPP)

197 trough; peak: 0.1–0.5 mg/L

198 + 0.4 mg of its metabolite 3-deacetylpancuronium/L

199 "normal": ≤ 2–3% of total hemoglobin (Hb); from 15–20%: cyanosis, headache, dizziness

200 carboxyhemoglobin (COHb) averages 1–2% in urban non-smokers and 5–6% in smokers

201 2 h after ingestion; concentrations above 2 mg/L at 4 h, 1.6 mg/L at 12 h, 0.6 mg/L at 16 h, and 0.16 mg/L at 24 h are lethal

202 3 h after ingestion of 400 mg with no severe symptoms

203 mean steady state trough concentration; peak concentration: 5–15 mg/L

204 for Parkinson's disease (appr. 15–50 pmol/mL)

205 peak: 0.5–3 mg/L

206 plasma concentrations below detection limit; plasma concentrations of the active metabolite 6-methoxy-2-naphthylacetic acid (assumed therapeutic range 8.1–21 mg/L; $t_{1/2}$: appr. 23–24 h), which appears to be responsible for the effects, were 10–37 mg/L 3–6 h after single oral doses of 250, 500, and 1000 mg, respectively

207 active metabolite 6-O-desmethyldonepezil

208 coma in a patient overdosing zonisamide, carbamazepine, and clonazepam

209 25–30 h in patients co-medicated with enzyme-inducing anticonvulsants (e.g., phenobarbital)

210 2–4 h in patients co-medicated with enzyme-inducing anticonvulsants (e.g., phenobarbital)

211 renal-transplant patients treated long-term (2–3 years) with mycophenolate mofetil had significantly lower trough plasma concentrations of the active metabolite mycophenolic acid (1.94 ± 0.24 mg/L) when compared to patients taking mycophenolate mofetil (1 g twice daily) short-term (2–10 months; 3.53 ± 0.45 mg/L); proposed mycophenolic acid pre-dose target concentration: 1–3.5 mg/L

212 as mycophenolic acid (active metabolite)

213 ten men with multiple sclerosis, 10–20 mg p.o. every 6 h and analyzed 30 min before the next dose; peak levels < 0.1 mg/L 30 min after a dose

214 nine patients, maximum tolerated oral dose 50–100 mg

215 $t_{1/2}$ of the metabolite 3-O-methyldopa: 15 h

216 appr. 2.5 µmol/l (1 mg/L) 24 h after single doses of 100–800 mg and during daily treatment with 200 mg

217 active metabolite 14-hydroxyclarithomycin ($t_{1/2}$: 5–7 h)

218 C_{max} following oral administration of 200, 400, 800, and 1200 mg, respectively: 3.7, 8, 18, and 29 mg/L; tentative target range according to Neels et al., 2004 [12]: 5–25 mg/L

219 at a daily dosage of 60, 120, and 240 mg the mean ± SD concentration in patients with symptomatic ventricular tachyarrhythmias (n = 9–18) was 75 ± 46, 144 ± 105, and 324 ± 180 nmol/L, respectively

220 nonlinear kinetics

221 appr. 5 h after ingestion of 3 g, not associated with severe toxicity to a 27-year-old woman

222 slightly increased (8–12 h) in patients with impaired hepatic function; active metabolites hydroxynefazodone ($t_{½}$: 2-5 h), m-chlorophenyl-piperazine ($t_{½}$: 4–10 h), and triazoledione ($t_{½}$: 10–12 h)

223 each as the active metabolite N-desmethylmesuximide; methsuximide ($t_{½}$: 1–2 h) steady state concentration: appr. 0.04–0.08 mg/L

224 mean steady state trough concentration in 15 young adults receiving a daily dose of 0.47–1.71 mg isotretinoin/kg: 0.05–0.34 mg/L ($t_{½}$: 29 ± 40 h), and for the 4-oxo metabolite ($t_{½}$: 22 ± 10 h): 0.16–0.68 mg/L

225 for depression; higher in case of schizophrenia (0.2–1 mg/L)

226 suggested threshold for the sum of clomipramine (0.05–0.06 mg/L) and N-desmethylclomipramine (0.16–0.18 mg/L): 0.2–0.24 mg/L

227 for the active metabolite E-3174 ($t_{½}$: 4–9 h); plasma concentration of losartan producing 50% of maximal blood pressure response to exogenous angiotensin-II: 0.032 mg/L

228 as ramiprilat ($t_{½}$: 13–17 (50–110) h)

229 half maximal inhibitory concentration (IC_{50}) for analgesic effect after oral surgery

230 the inhibitory concentration to reduce the level of extracellular hepatitis B DNA by 50% varied from 2.3 µg/L to 1.3 mg/L; C_{max} after 150–300 mg p.o.: 1.2–2.0 mg/L

231 C_{max} at steady state (666 mg three times daily p.o.)

232 after oral administration of the enteric-coated tablet

233 trough < 2 plus peak 6–10 (5–12) mg/L

234 reference value; < 0.015 mg/L urine

235 active metabolite 4'-hydroxynimesulide ($t_{½}$: 3–9 h)

236 mean C_{max} 126.5 and 226.3 ng/mL 2 h after 75 and 125 mg p.o. and 162.9 and 291.8 ng/mL after oral administration of 1.0 and 1.6 mg MDMA/kg body weight, respectively, to young adults; mean C_{max} for the metabolites 4-hydroxy-3-methoxymethamphetamine (HMMA) 171.9 and 173.5 ng/mL, 3,4-methylenedioxymethamphetamine (MDA) 8.4 and 13.8 ng/mL, and 4-hydroxy-3-methoxyamphetamine (HMA) 3.5 and 3.9 ng/mL, respectively

237 in patients > 60 years prolonged up to 10 h

238 adjuvant in methadone maintenance therapy

239 means of the ‘average’ steady state plasma concentration for the relatively high dose of 250 mg every 8 h appr. 0.8–1 mg/L

240 combination of distribution and elimination processes; $t_{½}$ up to 22 h in cases of overdose

241 as active metabolite fenofibric acid

242 appr. 37.5 mmol/L (= mval/L, mEq/L)

243 steady state concentration 21.6 ± 14.2 mg/L (mean ± SD) during continuous infusion of 3 g (1.1–2.2 mg/kg h) every 24 h in 44 patients undergoing coronary artery bypass graft surgery

244 target – whole blood – trough concentration if ciclosporine is being used at trough concentrations of 0.075–0.15 mg/L; without ciclosporine: appr. 0.03 mg/L (LC/UV assay)

245 4 h after ingestion of 30–40 tiagabine hydrochloride 8 mg tablets (coma)

246 bupropion plus 10-hydroxybupropion ($t_{½}$: 17–47 h)

247 calculated steady state concentration in children (4 months to 16 years) receiving 0.3 mg/kg body weight i.v.

248 femoral blood concentration of the metabolite desmethylalimemazine after fatal intoxication: 0.2–1.3 µg/g

249 40–50 min after 0.15 mg/kg i.v.

250 femoral blood concentration of the metabolite desmethylpromethazine after fatal intoxication (n=3): 0.3–1.8 µg/g

251 femoral blood concentration of the metabolite desmethyltrimipramine after fatal intoxication (n=10): 0.3–2.5 µg/g

252 µg/g femoral blood; in multiple-substance intoxication median 0.2 (P_{10}/P_{90}: 0.11/1.6) µg/g e.g., fatal overdose with tramadol, alprazolam (0.21 mg/L), and ethanol (1.29 g/kg) in a 30-year-old woman

253 enterohepatic circulation; prolonged in elderly subjects to 33.4 hours (range: 20.0–53.4 h)

254 whole blood

255 all data refer to the active metabolite teriflunomide (A771726)

256 steady state concentrations of 8.8 ± 2.9 mg/L at 5 mg/day, 18 ± 9.6 mg/L at 10 mg/day, and 63 ± 36 mg/L at 25 mg/day, respectively

257 steady state trough concentrations after 400 mg/d orally; two major metabolites modafinil acid (appr. 0.5–0.8 mg/L, $t_{½}$: 7.3 ± 1.1 h) and modafinil sulfone (appr. 4.5–5.3 mg/L), but neither appears to contribute to the wake-promoting properties of modafinil

258 mean plasma trough concentration at steady state obtained from 400 mg imatinib/day in 83 adult patients with chronic phase chronic myeloid leukemia; peak: 2.3 mg/L

259 in a 5-year-old girl

260 suggested minimum target trough concentration in patients with HIV-1 susceptible to the antiretroviral drug (dose of 800 mg two times daily)

261 active metabolite N-desethylamiodarone ($t_{½}$: 57–64 days), which achieves plasma concentrations similar to the parent compound

262 inactive metabolites deshydroxyethylopipramol ($t_{½}$: 97 ± 24 h) and opipramol N-oxide ($t_{½}$: 10.7 ± 3.2 h)

263 as 25-hydroxyvitamin D [25(OH)D, calcidiol]; vitamin D deficient: < 0.01 mg/L (< 10 ng/mL = 25 nmol/L); vitamin D insufficient: < 0.02 (–0.03) mg/L (50 (–75) nmol/L); > 0.02 mg/L (> 50 nmol/L) considered optimal vitamin D status; conversion factor: mg/L x 2,500 = nmol/L (ng/mL x 2.5 = nmol/L)

264 6 h after reportedly ingestion of 30 g in a 38-year-old woman

265 metabolite perindoprilat, 3–10 hours, with a prolonged terminal half-life between 25 and 120 h

266 sum of venlafaxine and O-desmethylvenlafaxine

267 for glaucoma 4–5 mg/L

268 doxapram plus keto-doxapram

269 24 h after ingestion of appr. 20 mL

270 active metabolite desethylamodiaquine ($t_{½}$: 1–10 days)

271 smokers: –0.6 ng/mL

272 risperidone plus 9-hydroxyrisperidone; 0.002-0.02 for risperidone, 0.01–0.06 mg/L for 9-hydroxyrisperidone

273 as active metabolite after administration of therapeutic doses of diazepam

274 active metabolite dimethadione (see Table)

275 HPLC-MS/MS (or FPIA) blood, in combination with ciclosporine microemulsion

276 median (interquartile range, IQR) in n=439 patients at hospital admission (1.3 [0.4–3.5] µmol/L; in 35 deaths: median (IQR) 4.7 [3.6–5.9] µmol/L

277 median (interquartile range, IQR) in n=264 patients at hospital admission; in 61 deaths: median (IQR) 846 (657–1183) µmol/L

278 in the presence of ethanol or during ethanol treatment; $t_{1/2}$ longer in patients with a serum creatinine concentration ≥ 130 µmol/L

279 median (interquartile range, IQR) in n=99 patients at hospital admission (4.9 [0.6–16.6] µmol/L; in 16 deaths: median (IQR) 12.3 [0.94–30.3] µmol/L)

280 3 dead infants aged ≤ 6 months with post-mortem blood levels of pseudoephedrine ranging from 4.7–7.1 mg/L

281 daily dose 2–8 mg p.o.

282 treatment goal: cystine levels < 1 nmol cystine/mg protein

283 according to other sources: < 0.005 mg/L; urine: < 0.012 mg/L; hair: < 0.5 µg/g; reference value for children in Germany: 0.015 mg/L urine. Case report: at day one 0.13–0.16 mg/L blood (urine: 67.5 mg/L) in a 43-year-old-male after ingestion of appr. 54 g arsenic trioxide

284 potentially increased risk for visual adverse effects (> 3.5 mg/L) and abnormal liver function, respectively

285 trough plasma buprenorphine and norbuprenorphine concentrations in excess of 0.0007 mg/L (0.7 ng/mL) were associated with minimal withdrawal symptoms in 11 heroin-dependent subjects

286 serum peak concentration of misoprostol acid (MPA) 574.8 ± 250.7, 287.6 ± 144.3, and 125.2 ± 53.8 pg/mL after sublingual, oral, and vaginal application, respectively, of 0.4 mg misoprostol to 40 women undergoing termination of pregnancy

287 on the first day of hospital admission after unintentional ingestion of appr. 400–500 mg carbachol (corresponding urine concentration: 374 mg/L).

288 main (probably inactive) metabolite: morphine-3-glucuronide (M3G); active metabolite: morphine-6-glucuronide (M6G)

289 metabolite: nordoxylamine

290 active ß-hydroxy-metabolite

291 targeted range between AUC of 9–12 mg/L/h

292 as amprenavir; suggested minimum target trough concentration in patients with HIV-1 susceptible to the antiretroviral drug (dose of 700 mg twice daily)

293 suggested minimum target trough concentration in patients with HIV-1 susceptible to the antiretroviral drug (dose of 300 mg once daily)

294 suggested minimum target trough concentration in patients with HIV-1 susceptible to the antiretroviral drug (dose of 400 mg twice daily)

295 suggested minimum target trough concentration in patients with HIV-1 susceptible to the antiretroviral drug (dose of 1,250 mg twice daily)

296 suggested minimum target trough concentration in patients with HIV-1 susceptible to the antiretroviral drug (dose of 1,000 mg twice daily)

297 suggested minimum target trough concentration in patients with HIV-1 susceptible to the antiretroviral drug (dose of 600 mg once daily)

298 suggested minimum target trough concentration in patients with HIV-1 susceptible to the antiretroviral drug (dose of 200 mg twice daily)

299 suggested minimum target trough concentration for antiretroviral therapy-experienced patients who have resistant HIV-1 strains (dose of 500 mg twice daily)

300 suggested minimum target trough concentration for antiretroviral therapy-experienced patients who have resistant HIV-1 strains

301 median (range) trough concentration from clinical trials (dose 600 mg twice daily); suggested threshold: > 0.55 mg/L

302 median (range) trough concentration from clinical trials

303 median (range) trough concentration from clinical trials

304 post-mortem heart blood level (death by hanging?)

305 active enantiomer of propoxyphene; active metabolite norpropoxyphene

306 heart blood

307 C_{max} at steady state achieved after 2–3 weeks of once-daily inhalation of 18 µg tiotropium; t_{max} after inhalation of 18 µg: 5 min

308 steady state peak concentration following a 300 mg twice daily or a 600 mg once-daily regimen

309 active metabolite cis-monohydroxyperhexiline ($t_{1/2}$: 10–29 h)

310 1–2 h after 50 mg

311 “laboratory alert level”; mostly according to AGNP Consensus Guidelines for therapeutic drug monitoring in psychiatry: update 2017 [4] i.e., drug concentrations above the recommended reference range, based on reports on intolerance or intoxications. In most cases, however, arbitrarily defined as plasma concentration that is 2-fold higher than the upper limit of the therapeutic reference range

312 active metabolite 6-hydroxybuspirone

313 C_{max} 1–2 h after 4 mg

314 at low dose therapy (2.5 mg); at maximum dose (25 mg): 0.001–0.004 mg/L

315 C_{max} 0.5–4 h after drug intake for 4 weeks

316 C_{max} after 2 h

317 60–90 min after intake of 1.2 mg/kg per day

318 four h after 20 mg

319 $t_{½}$ with ritonavir 15–23 h

320 active metabolite 8-hydroxyloxapine ($t_{½}$: 20–60 h)

321 C_{max} after 2h-infusion of 6 mg: 0.328 mg/L, after infusion of 2 mg: 0.246 mg/L

322 mean C_{max} after a single oral standard dose of 70 mg in healthy volunteers 33–41 ng/mL; mean C_{max} after a 2h-infusion of 10 mg: 265 ng/mL

323 mean C_{max} after a 4h-infusion of 15 mg: appr. 0.25 mg/L

324 male; female: –0.07 mg/L, children: –0.06 mg/L [reference value for children in Germany: 0.035 mg/L (whole) blood]

325 shorter in case of hemodialysis or continuous venovenous hemodiafiltration; in a, fatal, case with 4,400 mg methanol/L blood and in the presence of adequate ethanol level (1,000 mg/L or 1 ‰) appr. 3.5 h

326 after topical (dermal) application

327 prolonged in newborns (27.8 ± 21.3 h)

328 reference value for Germany: 0.014 mg/L urine

329 reference value for children in Germany: 0.0003 mg/L urine

330 reference value for children in Germany: 0.0045 mg/L urine

331 reference value in urine for children in Germany

332 reference value in whole blood in Germany

333 metabolite of 3,4 methylenedioxymeth(yl)amphetamine (MDMA)

334 $t_{½}$ (R)-MDE: 7.9 (6–11) h; $t_{½}$ (S)-MDE: 4.0 (3–6) h

335 40 h after oral ingestion of appr. 100 mg (0.03 mg/L 60 h after drug intake)

336 active metabolite phenytoin

337 prodrug; main active metabolites are morphine and morphine-6-glucuronide (M6G); main (probably) inactive metabolite = morphine-3-glucuronide (M3G)

338 in maintenance therapy e.g., for heavily dependent opioid addicts. 30 min after i.v- application of 150–300 mg diacetylmorphine (heroin): 0.1–0.24 mg/L morphine ($t_{½}$: 1–4 h), 2.6–5.9 mg/L morphine-3-glucuronide (M3G; $t_{½}$: (2–) 3–5 h), 0.5–1.0 mg/L morphine-6-glucuronide (M6G; $t_{½}$: (1–) 2–3 h), and 0.08–0.29 mg/L 6-monoacetylmorphine (6-MAM; $t_{½}$: appr. 2–5 min; n=4); in another study 30 min after i.v- application of 260–300 mg diacetylmorphine: 0.39–0.75 mg/L morphine, 3.2–5.2 mg/L M3G, 0.5–0.7 mg/L M6G, and 0.08–0.19 mg/L 6-MAM (n=4)

339 depending on tolerance and state/severity of pain

340 metabolites: norbuprenophine (active; plasma concentration after therapeutic buprenorphine doses appr. 0.5–2 (-20) ng/mL; $t_{½}$: 35.6 (1.1–66.8) h after i.v., 73.6 (13.4–143) h after buccal, and 83 (10–243) h after sublingual application), buprenorphine-glucuronide, and norbuprenorphine-glucuronide

341 active enantiomer of zopiclone

342 active enantiomer of methylphenidate

343 active enantiomer of citalopram

344 strongly dependent on pH of urine

345 symptomatic poisoning in adults is more likely with doses above 90 mg

346 on hospital day #2

347 19 h post-ingestion of appr. 4 g

348 five h post-ingestion; all patients with a plasma paraquat level above 3.44 mg/L died

349 prolonged in (paraquat-induced) renal failure to appr. 80–120 (-150) h

350 active enantiomer of moramide

351 active enantiomer of fenfluramine

352 active enantiomer of methadone (see Table); in many cases, analytical discrimination between both enantiomers is not possible

353 after a bolus dose of 0.25 mg/kg body weight (n=10): 2.3 mg/L at 3 min, 0.84 mg/L at 30 min, 0.61 mg/L at 1 h, and 0.44 mg/L at 2 h

354 active metabolite oxypurinol

355 eleven h post-ingestion

356 $t_{½}$ in poor metabolizers of cytochrome P450 2D6 is appr. 21 h

357 metabolite of cocaine

358 dependent on pH of urine

359 sum of dibenzepine and desmethyldibenzepine

360 active metabolite of trimethadione

361 metabolite: acetone

362 in non-users of opioids

363 active metabolite norlorcainide ($t_{½}$: 28–32 h), therapeutic plasma concentration: 0.1–1.5 mg/L

364 metabolite of azathioprine

365 (cis-)isomer of clopenthixol

366 active metabolites norsibutramine ($t_{½}$: 12–22 h) and dinorsibutramine ($t_{½}$: 14–23 h)

367 with silver sulphadiazine ointment for burns: 0.06–0.6 mg/L (non-toxic)

368 active metabolite fexofenadine

369 as metabolite of chloralhydrate

370 peak plasma concentrations (C_{max}) in four subjects after 1 h chewing khat leaves that supplied 28.1–45.1 mg cathine and 34.3–64.1 mg cathinone

371 higher for poor metabolizers of cytochrome P450 2C9

372 enantiomer of loratadine

373 0.001–0.0035 mg/L for the active metabolite 3-hydroxydesloratadine ($t_{½}$: 17–27 h)

374 enantiomer of ketoprofen

375 after a single oral dose of 2 mg

376 average of 12 victims

377 serum morphine (active metabolite) levels were appr. 0.013 in extensive metabolizers and 0.003 in poor metabolizers

378 enantiomer of cetirizine

379 endogenous: –0.01–0.09 ng/mL

380 active metabolite of psilocybin

381 during anaesthesia

382 measured 14 h post-ingestion

383 serum concentrations from 1.2–2.0 ng/mL (0.0012–0.002 mg/L) were associated with a higher risk of death

384 main metabolite desethylchloroquine

385 plasma concentrations of hydroxychloroquine less than 0.2 mg/L are indicative of poor medication adherence in patients with systemic lupus erythematodes. Mean serum concentration in n=20 patients treated with oral doses of three times 200 mg hydroxychloroquine sulfate daily for COVID-19 was 0.46 ± 0.2 mg/L [1226]

386 at least 5 days; terminal $t_{½}$ appr. 43 days

387 for actual minimal inhibitory concentration (MIC) distributions see e.g., EUCAST database at: www.eucast.org

388 urine: < 0.0006 mg/L, < 0.0007 mg total Cr/24 h

389 appr. 9.1–68.1 nmol/L

390 to convert pg/mL to pmol/L, multiply by 0.7378. In a recent general population study in the Netherlands, plasma concentrations > 455 pg/mL were associated with increased risk of all-cause mortality

391 sum enzalutamide and active metabolite N-desmethylenzalutamide; at week 13 in metastatic castration-resistant prostate cancer (mCRPC) patients taking 160 mg enzalutamide/d (n=680), the trough concentration for enzalutamide was 11.4 ± 2.95 mg/L, 13.0 ± 3.8 mg/L for N-desmethylenzalutamide, and 8.4 ± 6.8 mg/L for the carboxylic acid metabolite

392 cutoff AUC_{0-24}; can be a good predictor of grade 3-4 adverse events in elderly (Japanese) patients with newly diagnosed multiple myeloma

393 suggested cut-off for endogenous concentrations: blood 4 mg/L, urine 10 mg/L, and hair 3 ng/mg

394 N-desmethyl-U-47700 is probably a better blood and (mainly) urine biomarker of U-47700 intake and intoxication. N,N-didesmethyl-U-47700 is another biomarker

395 O-desmethylocfentanil is probably a relevant metabolite

396 post-mortem in peripheral blood; in heart blood: 0.005, 0.023, and 0.027 mg/L

397 to minimize the development of lactic acidosis plasma concentrations above 2.5 mg/L should be avoided

398 C_{max} during steady state; $t_{½}$ of N-debutyl-dronedarone appr. 20–25 h

399 + 1.4 mg/L phenazepam

400 + 0.83 mg/L flubromazepam ($t_{½}$ appr. 100 h)

401 + 0.37 mg/L U-47700

402 trough edoxaban concentration related to frequency of major gastrointestinal bleeding (MGIB); probability of MGIB significantly increased at appr. 0.06 (-0.1) mg/L

403 after dermal or inhalative occupational exposure

404 + 0.2 mg/L propofol; self-administration

405 steady state concentrations expected under a therapeutic dose of 100 mg/d

406 0.2–2 h after 4 mg

407 in a 14-year-old girl 2.5 h after ingestion of 21 long-acting methylphenidate 54 mg tablets (total of 1,134 mg methylphenidate)

408 + 900 mg/L ethanol

409 $t_{½}$ in 3–12 months-old children appr. 2.5 h

410 high level of protein binding

411 active (?) metabolite cyclopropylnorfentanyl

412 in 32 cases, post-mortem serum concentrations of 0.0153 ± 0.0119 mg/L (median 0.0123 mg/L) cyclopropylfentanyl were reported

413 post-mortem blood concentrations ranging from 0.02 to 0.18 µg/g in nine cases of accidental intoxications where mitragynine and O-desmethyltramadol were detected

414 fatal case, together with a blood concentration of 0.39 mg/L mitragynine; the cause of death was ruled propylhexedrine toxicity

415 on the market as sodium salt complex of anionic forms of sacubitril (an inactive prodrug of the active neprilysin inhibitor, sacubitrilat [$t_{1/2}$: 18.4 ± 6.8 h[416]]) and valsartan

416 in patients with heart failure with reduced ejection fraction; $t_{1/2}$ is higher when compared to healthy subjects, $t_{1/2}$: sacubitril 1–2 h, sacubitrilat 9–13 h

417 as (±)-threo 4-fluroromethylphenidate (4F-MPH); urine concentration 0.83 mg/L

418 as (the active metabolite) desmethyldiazepam = nordazepam

419 maximum plasma concentration after applying 2 mg of sunscreen (spray, lotion or cream) per 1 cm^2 to 75% of body surface area (BSA) 4 times per day for 4 days (each n=6 healthy volunteers)

420 active metabolite 5-hydroxysaxagliptin ($t_{1/2}$: 3–7 h)

421 according to Høiseth et al. [835], the median (range) concentrations in 'drugged drivers' were:
0.012 mg/L (0.00048-0.10) for flubromazolam (n=25),
0.055 mg/L (0.0047-1.2) for flubromazepam (n=24),
0.013 mg/L (0.0021-0.057) for diclazepam (n=15),
0.050 mg/L (0.019-0.17) for etizolam (n=14),
0.0053 mg/L (0.0019-0.011) for clonazolam (n=7) and
0.074 mg/L for pyrazolam (n=1).

422 + 560 mg/L valproate

423 active metabolite norsertraline (desmethylsertraline, DMS)

424 post-mortem femoral blood concentrations of appr. 0.001 mg/L clenbuterol, appr. 0.056 mg/L stanozolol, and appr. 0.008 mg/L metandienone in a 34-year-old male bodybuilder with previous unknown heart disease

425 post-mortem blood concentrations of 0.163 mg/L trenbolone and 0.257 mg/L stanozolol in a 36-year-old male bodybuilder

426 one hour after the last oral 300 mg dose

427 active metabolite 4-hydroxyalprenolol, therapeutic: 0.04–0.06 mg/L

428 0.6–0.7 hours after oral ingestion of 50, 100, or 200 mg average plasma concentrations of 0.68, 0.88, or 2.15 mg/L were achieved

429 prodrug of azilsartan

430 patients receiving a single daily oral 5 mg-dose for two weeks developed steady state peak plasma concentrations averaging 0.05 mg/L at 2.3 h post-administration

431 a single dermal scalp application of 5% benzyl alcohol lotion resulted in plasma benzyl alcohol concentrations of 1.6–3.0 mg/L at 0.5–1 hours in four of 19 children ages 6 months to 11 years

432 two h post-ingestion

433 same case, 20 h post-ingestion (prior to death), in a mixed intoxication with MCPA

434 a single oral 300 mg-dose in 4 healthy adults led to an average peak plasma carboxybuprenolol concentration of 4.19 mg/L

435 buproprion + hydroxybupropion

436 $t_{½}$ buproprion: 1–15 h, $t_{½}$ hydroxybuproprion: 17–47 h

437 buspirone + 8-hydroxybuspirone 0.001–0.004 mg/L

438 $t_{½}$ for metabolites

439 active metabolites are N-desmethylcariprazine and N,N-didesmethylcariprazine

440 active metabolite meprobamate, therapeutic plasma concentration: 10–30 mg/L, $t_{½}$: appr. 8 h

441 carisoprodol concentration (without meprobamate); corresponding meprobamate levels were 6.8–62 mg/L

442 car drivers with no signs of impairment

443 during anaesthesia

444 plus 86 mg/L desmethylclobazam

445 the active metabolite of clopidogrel is a thiol derivative which is mainly formed via cytochromes P450 (CYP2C19, among others) in the liver

446 at 4–8 h

447 ‘Holland film’; 15 hours post-ingestion

448 ante-mortem; post-mortem femoral vein blood 0.6 ng/g, urine 2.93 ng/g

449 current German biologic tolerance value (BAT) for exposure to cresol isomers in the workplace is 200 mg/L in an end-of-shift urine specimen

450 in exposed workers at air concentrations of 5–710 ppm at the end of the day

451 mono intoxications

452 after oral administration in mice

453 several days after exposure

454 17-month-old child

455 post-mortem blood concentrations

456 simultaneous use of alcohol and disulfiram

457 0.2–0.35 mg/L at 2 h, 0.05–0.16 mg/L at 12 h

458 in poor metabolizers

459 diazepam plus nordazepam (see Table)

460 peak plasma concentration of an oral standard dose of 800 mg clodronate

461 a lethal blood concentration of nicotine is about 2 mg/L, corresponding to appr. 4 mg/L plasma

462 cotinine concentration in urine in a 15-month-old female 12 h after admission to ICU was 1.7 mg/L; she died at hospital day 44

463 median blood hexane level of 1,200 unexposed United States citizens

464 data from 13 fatalities, post-mortem femoral blood

465 mg/kg; 11 persons with certified deaths by intoxication with more than one drug and/or with drugs in combination with a significant concentration of ethanol

466 prodrug of 4-hydroxybutyrate (GHB)

467 active metabolite 7-hydroxymitragynine with appr. 30 times higher potency at μ-opioid receptors

468 a peak plasma concentration of 0.0034 mg/L diclazepam was achieved after oral intake of 1 mg diclazepam

469 peak plasma concentration after 1 μg/kg intranasal dexmedetomidine 0.00025–0.00028 mg/L (0.25–0.28 ng/mL). Peak plasma concentration of 0.00034 mg/L (0.34 ng/mL) 38 min after an intranasal dose of 84 μg to 6

healthy men; the same dose given by 10 min intravenous infusion to the same subjects produced median end of infusion peak plasma level of 0.0035 mg/L (3.5 ng/mL)

470 three fatal cases with more than one drug and/or with drugs in combination with a significant concentration of ethanol

471 four cases

472 blood concentrations of emetine were measurable in only 6 of 10 emergency room adult patients who received 30 mL of ipecac syrup for treatment of drug or chemical overdose

473 arterial blood enflurane levels present in 4 adult surgical patients during enflurane anaesthesia

474 fatalities after enflurane abuse

475 appr. 3 mg/L 6 h after the last dose in 20 patients who received 400 mg twice a day over a period of 4 days

476 peak plasma levels 1–2 hours after ingestion

477 intramuscular; intravenous: 2 min

478 endogenous plasma epinephrine concentrations in 40 supine resting adults

479 peak plasma levels 1–2 h after ingestion of 50–100 mg

480 prodrug of the active compound eslicarbazepine

481 in one case 200 mg/L in serum during hospital treatment; 650 mg/L in post-mortem blood

482 under treatment with fomepizole or ethanol during high-efficiency hemodialysis

483 under treatment with fomepizole but without hemodialysis

484 concentration range in 7 fatal ethylone cases

485 seven fatal ethylphenidate cases

486 peak plasma concentrations after 0.5 and 1 mg etizolam, respectively

487 post-transplantation, trough levels of everolimus should be maintained at 3–8 ng/mL when used in combination with other immunosuppressive drugs (calcineurin inhibitor and glucocorticoid) and at 6–10 ng/mL when used without a calcineurin inhibitor. In the treatment of tuberous sclerosis complex, it is recommended that everolimus concentrations should be managed at 5–15 ng/mL

488 prodrug of penciclovir

489 active metabolite fenofibric acid, therapeutic plasma concentration 5–11 mg/L

490 active metabolite amphetamine

491 serum fenitrothion concentrations in n=27 adults who survived poisoning with this chemical

492 prodrug of the active metabolite 5-hydroxymethyl-tolterodine (5-HMT)

493 a single 8 mg oral extended-release dose in 14 healthy adults yielded an average peak plasma concentration of 0.005 mg/L at 5 h; peak plasma levels of 5-HMT in poor cytochrome P450 2D6 metabolizers were appr. twice those in extensive metabolizers

494 as 5-hydroxymethyltolterodine (5-HMT)

495 20 healthy younger adults receiving 1.25 or 5 mg oral doses once daily for 1 week attained average peak plasma levels of 0.001 or 0.0043 mg/L, respectively, at 12 h after the first dose and 0.005 or 0.018 mg/L, respectively, at 12 h after the last dose

496 a single oral 100 mg dose given to n=24 healthy fasting adults resulted in a median peak plasma flibanserin concentration of 0.413 mg/L

497 serum concentration in eight nonfatal poisonings

498 one patient

499 a single oral 0.5 mg dose given to one adult resulted in a peak serum concentration of 8 ng/mL (8 h post-ingestion)

500 19 h after ingestion of 3 mg flubromazolam

501 one patient

502 mg/kg; sum of flunitrazepam and 7-aminoflunitrazepam in 175 persons dying solely from flunitrazepam overdosage

503 n=12 adults arrested for impaired driving

504 ante-mortem; post-mortem 0.48 mg/L

505 in four fatal cases

506 intoxication with AH-7921 and 2-FMA, and other drugs

507 median of 6 case reports: post-mortem 1.3–6.8 mg fluoxetine/L blood and median 2.1 (0.9–5.0) mg norfluoxetine/L blood

508 at 10 h after drug intake under steady state conditions

509 blood flurazepam concentrations in 3 adult deaths due solely to flurazepam

510 concentration suggested by the manufacturer to cause adequate inhibition of alcohol dehydrogenase activity (Antizo® package insert, 1997)

511 2.4–3.2 h after a single 10 mg or 20 mg oral dose

512 as fosinoprilat

513 prodrug of amprenavir

514 2.6–3.1 hours after the last of 40 or 80 mg oral doses twice daily for 2 weeks

515 water soluble prodrug of propofol

516 data from n=13 fatalities

517 prodrug of 4-hydroxybutyrate (GHB)

518 fatalities

519 background blood gold levels in n=130 German citizens: 0.0001 (0.00001–0.0029) mg/L

520 immediate release dosage form; extended release: 10–50 h

521 two children, ages 4 and 5, accidentally given haloperidol doses of 2 and 5 mg p.o.

522 endogenous plasma concentrations in healthy adults

523 the elimination $t_{1/2}$ of glycerol depends upon the glycerol concentration ($t_{1/2}$ = [G]o/2k); the serum glycerol concentration decreases more rapidly in the late phase of the observation period

524 in poisoned patients

525 post-mortem blood glyphosate levels in n=16 adults who intentionally ingested overdoses; death was strongly associated with greater age, larger ingestions and high plasma glyphosate concentrations on admission (> 734 mg/L)

526 5 fatal cases after abuse by inhalation or ingestion with suicidal intent

527 analytical testing was performed 3 months after autopsy

528 for 6-acetylmorphine, plasma levels in 11 patients 15 min after the end of smoking of 150–400 mg diacetylmorphine (heroin) were 0.068 (0.015–0.156) mg/L, and 0.021 and 0.009 mg/L after 45 and 90 minutes, respectively.

529 nine neurology patients receiving daily oral therapy with 1150–2900 mg hydroxytryptophan plus 200 mg of carbidopa for 8–54 months had steady state peak plasma hydroxytryptophan levels averaging 9.4 (range 3.0–17) mg/L

530 co-administration of carbidopa resulted in a doubling of the elimination half-life of 5-HTP

531 4 h post-ingestion; survived with supportive measures

532 adults with unusually high oral doses of 5 to 75 mg/kg idebenone achieved average peak plasma levels of 1.64–9.53 mg/L

533 7 h post-ingestion

534 a single oral 100 mg dose given to 6 adult male congestive heart failure patients resulted in an average peak plasma concentration of 1.3 mg/L at 1.4 h

535 1 hour after a single oral 200 mg dose to six healthy, younger, fasting men

536 the elimination of isoflurane follows a 3 term exponential decay, with half-lives of 6.7 minutes, 1.3 h, and 58 h representing the vessel-rich, muscle, and fat compartments, respectively.

537 deaths after self-administration of isoflurane

538 the metabolites isosorbide-5-mononitrate (IS-5-MN) and isosorbide-2-mononitrate (IS-2-MN) show pharmacological activity

539 the sublingual administration of 5 mg ISDN to n=6 healthy young man resulted in an average peak plasma concentration of 0.016 mg/L ISMN at 30 minutes; peak plasma levels in n=7 patients receiving chronic high dose therapy (360–720 mg ISDN daily) ranged from 0.046–0.224 mg/L (ISDN), 0.179–0.512 mg/L IS-2-MN, and 0.91-1.97 mg/L IS-5-MN

540 the United Kingdom established a threshold of 0.02 mg/L for blood ketamine as being indicative of impaired driving ability (UK GOV 2014)

541 active metabolite norketotifen

542 active metabolites ortho- and para-hydroxyatorvastatin; reference plasma concentration 0.013–0.043 mg/L and 0.001–0.005 mg/L, respectively

543 active metabolite lovastatin hydroxy acid; reference plasma concentration 0.003–0.018 mg/L

544 active metabolite simvastatin hydroxy acid; reference plasma concentration 0.001–0.009 mg/L

545 (major) active metabolite canrenone

546 metabolites 11-hydroxyyohimbine (active) and 10-hydroxyyohimbine

547 in subclavian blood

548 (active?) metabolite norolanzapine, therapeutic range 0.006–0.024 mg/L

549 metabolites norquetiapine, 7-hydroxyquetiapine, and 7-hydroxy norquetiapine

550 paliperidone (9-hydroxyrisperidone) could be a prescribed antipsychotic (parent drug) and a metabolite of risperidone

551 a case with severe hypoglycemia: serum concentration 1 h after hospital admission; O-desmethyltramadol 1.3 mg/L, N-desmethyltramadol 3.3 mg/L, acetaminophen 109.5 mg/L

552 the ‘gray baby syndrome’ occurs in newborns who develop excessive serum concentrations of the drug

553 serum cathine concentrations in n=19 khat cases of suspected driving under the influence of drugs

554 serum cathinone concentrations in 16 of 19 khat cases of suspected driving under the influence of drugs

555 three days after ingestion of a liquid containing hexavalent chromium (Cr(VI)) and inorganic arsenic (iAs) the total Cr concentrations were 2.18 and 1.07 mg/L in whole blood and plasma, respectively, and 4.54 mg/L Cr(VI) in erythrocytes

556 appr. six h after oral ingestion of 300 mg clindamycine-HCl

557 metabolic precursor of lorazepam (see Table)

558 persons survived 1–3 days before they died

559 in combination with 0.035 mg/L etizolam (see Table) in femoral blood

560 elimination of enflurane follows a 3-term exponential decay, with half-lives of 18 min (17% of the absorbed dose, located in the central compartment), 3.2 h (41% from the muscle tissue compartment) and 36 h (42% from the fat compartment)

561 in femoral blood; in heart blood 14,585 ng/L

562 blood ketamine and norketamine concentrations in n=14 impaired drivers averaged 0.42 (range 0.17-0.85) mg/L and 0.61 norketamine (range 0.19-1.4) mg/L, respectively

563 six healthy women given a single oral 50 mg tablet attained peak plasma levels averaging 0.61 mg/L benzydamine

564 a target range of 3-7 mg/L for the trough serum infliximab concentration was suggested for inflammatory bowel disease patients

565 post-mortem femoral blood concentrations found in four cases where other (illegal) drugs were involved

566 active metabolite N-desmethyltrimipramine (= nortrimipramine)

567 active metabolites O-desmethylvenlafaxine (major) and N-desmethylvenlafaxine

568 active metabolite N-desmethylenzalutamide

569 high dose therapy in cancer patients

570 enantiomer of milnacipran (see Table)

571 active metabolites monoethylglycinexylidide (MEGX) and glycinexylidide (GX)

572 active metabolite desipramine, $t_{1/2}$: 15–25 h (see Table)

573 active metabolite norlorcainide, $t_{1/2}$: 28 h, therapeutic plasma concentration appr. –1.5 (-2) mg/L

574 (minor) active metabolite lorazepam

575 post-mortem (3 days post-admission)

576 depending on the route of administration

577 endogenous level

578 depending on the dose protocol and leucovorin application; minimal cytotoxic concentration appr. 0.01 µmol/L (= appr. 0.005 mg/L)

579 24 h, 48 h, and 72 h after high-dose i.v. infusion, target concentrations < 10 µmol/L (4.6 mg/L), < 1 µmol/L (0.46 mg/L), and < 0.1 µmol/L (0.046 mg/L), respectively

580 active metabolite of MDMA

581 major metabolite 5-methylpyrithyldione

582 nonenzymatically transformed to 2,2-dichlorvinyl-dimethyl-phosphate (dichlorvos, DDVP) (see Table)

583 maximum plasma concentration after applying 2 mg of sunscreen (spray or lotion) per 1 cm^2 to 75% of body surface area at 0 h on day 1 and 4 times on day 2 through day 4 at 2-h-intervals (each n=12 healthy volunteers)

584 major and active metabolite O-desmethyltramadol; further metabolites N-desmethyltramadol and N, O-didesmethyltramadol

585 steady state after Kratom tea for 7 days in nine chronic, regular, healthy users

586 in case one, plasma moclobemide was 2.8 mg/L with 1.8 mg/L clomipramine; in case two plasma moclobemide 18 mg/L; in case three 60.9 mg/L; none of the patients showed serious effects during 24 h of observation. Plasma moclobemide at 10 to 30 times therapeutic was not associated with major toxic effects; see eg. ref. [516].

587 actve metabolite of the prodrug moexipril

588 in unexposed adults

589 sum naltrexone and 6ß-naltrexol

590 measurement of serum netilmicin concentrations, with maintenance within the ranges 6 to 10 mg/L (peak) and 0.5 to 2 mg/L (trough), are desirable

591 active metabolite N-desethyloxybuti(y)nin; therapeutic plasma concentration 0.01-0.08 mg/L

592 active metabolite 6-oxymorphol

593 neutropenia is related to the duration that plasma concentrations were ≥ 0.05 µmol/L or > 0.1 µmol/L

594 elimination of bisphosphonates is extremely slow, and their terminal half-life can be as long as 10 years in humans

595 the use of a nomograph/nomogram relating plasma drug concentration, time since ingestion and hepatotoxicity is helpful in evaluating the need for antidotal treatment, see eg. ref. [1148, 1149]

596 paraoxon is the active metabolite of parathion

597 active metabolites 7-hydroxypericyazine and pericyazine sulphoxide

598 also an active metabolite of thiopental

599 58-year-old woman who ingested 26 g of naproxen in a suicidal attempt and developed cardiovascular shock, hypocoagulability and thrombopenia

600 active metabolite perindoprilat

601 whole blood concentration

602 monitoring and management of therapy by International Normalized Ratio (INR) and by the thromboplastin time (Quick test)

603 for invasive aspergillosis

604 active metabolite N-desalkylquetiapine (= norquetiapine, $t_{½}$: 10–13 h); therapeutic plasma concentration 0.1–0.25 mg/L

605 given to cancer patients in a phase I study

606 controlled anesthesia

607 active enantiomer of racemic modafinil

608 as modafinil

609 major active metabolite of acetylsalicylic acid

610 C_{max}; six healthy adults inhaled a single high dose of vaporized salvinorin A (n=4, 21 µg/kg; n=2, 18 µg/kg body weight)

611 asymptomatic silver workers

612 16 h post-ingestion of 1700 mg sitaglipin

613 mixed intoxication with several other drugs and ethanol

614 peripheral blood (urine: 88 mg/L), plus 0.58 mg oxycodone/L blood

615 C_{max} at 2 h

616 in non-exposed citizens

617 active metabolite quinalaprilat, therapeutic plasma concentration 0.6–1.8 mg/L

618 as quinalaprilat

619 active metabolite trandolaprilat, therapeutic plasma concentration 0.001–0.006 mg/L, $t_{½}$: 16–24 h

620 peak plasma concentration

621 active 3-hydroxymethyl metabolite, $t_{½}$: 13–15 h

622 $t_{½}$ 10–12 h for the metabolite trichloroethanol and 70–85 h for the metabolite trichloroacetic acid

623 $t_{½}$ 12–13 h for the metabolite trichloroethanol and 86–99 h for the metabolite trichloroacetic acid

624 the plasma concentration of the active metabolite 3-desacetylvecuronium ($t_{½}$: 2 h) may exceed that of vecuronium

625 $t_{½}$ of the metabolite O-desmethylvenlafaxine: 10–20 h

626 post-mortem baseline zinc concentrations: 4.0–8.7 mg/L

627 whole body

628 peak plasma concentrations at end-of-infusion after doses of 4–16 mg

629 at 1–3 h

630 at 1.5–2 h

631 active metabolite 4-(4-guanidinobenzoyloxy)phenylacetic acid

632 C_{max} and $t_{½}$, respectively, after a single oral 200 mg camostat mesilate dose in five healthy adults

633 as arsenious acid, after infusion of arsenic trioxide

Abbreviations: appr. approximately, *AUC* area under the (blood plasma concentration-time) curve, C_{max} maximum (peak) plasma/serum concentration, C_{min} minimum (trough) plasma/serum concentration (usually at steady state), *h* hour/hours, *ICU* intensive care unit, *min* minutes, *mol wt* molecular weight, *ref.* reference(s), *SD* standard deviation, $t_{½}$ terminal elimination half-life (if not stated otherwise), t_{max} time to peak concentration (C_{max})

References

1. Yuen GJ, Weller S, Pakes GE. A review of the pharmacokinetics of abacavir. Clin Pharmacokinet. 2008;47(6):351-71.

2. University of Liverpool and eMedFusion. University of Liverpool and eMedFusion. http://www.hiv-druginteractions.org/. Accessed 29 Feb 2020.

3. IBM Micromedex® DRUGDEX® (electronic version). IBM Watson Health, Greenwood Village, Colorado, USA. Available at https://www.micromedexsolutions.com/ (updated periodically).

4. Hiemke C, Bergemann N, Clement HW, Conca A, Deckert J, Domschke K, et al. Consensus guidelines for therapeutic drug monitoring in neuropsychopharmacology: update 2017. Pharmacopsychiatry. 2018;51(1/02):9-62.

5. Saivin S, Hulot T, Chabac S, Potgieter A, Durbin P, Houin G. Clinical pharmacokinetics of acamprosate. Clin Pharmacokinet. 1998;35(5):331-45.

6. Rooney M, Massey KL, Jamali F, Rosin M, Thomson D, Johnson DH. Acebutolol overdose treated with hemodialysis and extracorporeal membrane oxygenation. J Clin Pharmacol. 1996;36(8):760-3.

7. Schulz M, Meyer W, Schmitz W, Scholz J, Schmoldt A. Beta-Rezeptorenblocker. Grundlagen zur Arzneimittelauswahl für eine rationale Therapie [Beta receptor blockers. Principles for drug selection for rational therapy] [Article in German]. Med. Monatsschr. Pharm. 1989;12(8):237-44.

8. Regenthal R, Krueger M, Koeppel C, Preiss R. Drug levels: therapeutic and toxic serum/plasma concentrations of common drugs. J Clin Monit Comput. 1999;15(7-8):529-44.

9. Meyer FP. Indicative therapeutic and toxic drug concentrations in plasma: a tabulation. Int J Clin Pharmacol Ther. 1994;32(2):71-81.

10. Uges DRA. Referentiewaarden van xenobiotica in humaan material. Pharm Weekbl. 1995;130:180-204.

11. Uges DRA. Orientierende Angaben zu therapeutischen und toxischen Konzentrationen von Arzneimitteln und Giften in Blut, Serum oder Urin [in German]. Weinheim: VCH; 1990.

12. Neels HM, Sierens AC, Naelaerts K, Scharpe SL, Hatfield GM, Lambert WE. Therapeutic drug monitoring of old and newer anti-epileptic drugs. Clin Chem Lab Med. 2004;42:1228-55.

13. Schulz M, Schmoldt A. Zusammenstellung therapeutischer und toxischer Plasmakonzentrationsbereiche von Arzneistoffen [in German]. Anaesthesist. 1994;43(12):835-44.

14. Schulz M, Schmoldt A. Therapeutic and toxic blood concentrations of more than 500 drugs. Pharmazie. 1997;52(12):895-911.

15. Regenthal R, Krüger M, Köppel C, Preiß R. Zu Möglichkeiten und Grenzen von therapeutischen und klinisch-toxikologischen Referenzwerten für Plasma-/Serum-/Vollblutkonzentrationen von Arzneimitteln bei akuten Vergiftungen - eine Übersicht [in German]. Anästhesiol Intensivmed. 1999;40:129-44.

16. Wang G, Maranelli G, Perbellini L, Raineri E, Brugnone F. Blood acetone concentration in "normal people" and in exposed workers 16 h after the end of the workshift. Int Arch Occup Environ Health. 1994;65(5):285-9.

17. Zettinig G, Watzinger N, Eber B, Henning G, Klein W. Überlebte Vergiftung nach Einnahme der zehnfachen Letaldosis von Aceton [in German]. Dtsch Med Wochenschr. 1997;122(48):1489-92.

18. Adams KF, Jr., Patterson JH, Gattis WA, O'Connor CM, Lee CR, Schwartz TA, et al. Relationship of serum digoxin concentration to mortality and morbidity in women in the digitalis investigation group trial: a retrospective analysis. J Am Coll Cardiol. 2005;46(3):497-504.

19. Dobbs RJ, O'Neill CJ, Deshmukh AA, Nicholson PW, Dobbs SM. Serum concentration monitoring of cardiac glycosides. How helpful is it for adjusting dosage regimens? Clin Pharmacokinet. 1991;20(3):175-93.

20. El Desoky E, Meinshausen J, Buhl K, Engel G, Harings-Kaim A, Drewelow B, et al. Generation of pharmacokinetic data during routine therapeutic drug monitoring: Bayesian approach vs. pharmacokinetic studies. Ther Drug Monit. 1993;15(4):281-8.

21. Hoppe MM, Iafrate RP, Hendeles L, Neims A. A pediatric drug dosing and monitoring guide. Fl J Hosp Pharm. 1988;8:259-69.

22. Josune I, Victoria CM, Mar AM, Dominguez-Gil HA. Impact of written guidelines on the appropriateness of serum digoxin concentrations. Ann Pharmacother. 1993;27(6):791-2.

23. Mordel A, Halkin H, Zulty L, Almog S, Ezra D. Quinidine enhances digitalis toxicity at therapeutic serum digoxin levels. Clin Pharmacol Ther. 1993;53(4):457-62.

24. Rathore SS, Curtis JP, Wang Y, Bristow MR, Krumholz HM. Association of serum digoxin concentration and outcomes in patients with heart failure. JAMA. 2003;289(7):871-8.

25. Terra SG, Washam JB, Dunham GD, Gattis WA. Therapeutic range of digoxin's efficacy in heart failure: what is the evidence? Pharmacotherapy. 1999;19(10):1123-6.

26. Ujhelyi MR, Colucci RD, Cummings DM, Green PJ, Robert S, Vlasses PH, et al. Monitoring serum digoxin concentrations during digoxin immune Fab therapy. DICP. 1991;25(10):1047-9.

27. Wells TG, Young RA, Kearns GL. Age-related differences in digoxin toxicity and its treatment. Drug Saf. 1992;7(2):135-51.

28. Cham BE, Johns D, Bochner F, Imhoff DM, Rowland M. Simultaneous liquid-chromatographic quantitation of salicylic acid, salicyluric acid, and gentisic acid in plasma. Clin Chem. 1979;25:1420-5.

29. Johnson MW, MacLean KA, Caspers MJ, Prisinzano TE, Griffiths RR. Time course of pharmacokinetic and hormonal effects of inhaled high-dose salvinorin A in humans. J Psychopharmacol. 2016;30(4):323-9.

30. Irey NS, Froede RC. Evaluation of deaths from drug overdose. A clinicopathologic study. Am J Clin Pathol. 1974;61:778-84.

31. Mandelli M, Tognoni G. Monitoring plasma concentrations of salicylate. Clin Pharmacokinet. 1980;5:424-40.

32. Martens J, Meyer FP. Besondere Eignung eines photometrischen Verfahrens zur Bestimmung von Salicylsäure im Therapeutischen Drug Monitoring [in German]. Pharmazie. 1995;50(1):41-3.

33. Pond SM, Armstrong JG, Henderson A. Late diagnosis of chronic salicylate intoxication. Lancet. 1993;342:687.

34. Watson JE, Tagupa ET. Suicide attempt by means of aspirin enema. Ann Pharmacother. 1994;28:467-9.

35. Larsen FG, Jakobsen P, Knudsen J, Weismann K, Kragballe K, Nielsen-Kudsk F. Conversion of acitretin to etretinate in psoriatic patients is influenced by ethanol. J Invest Dermatol. 1993;100:623-7.

36. Sommerburg C, Bauer R, Orfanos CE, Petres J, Thiele B, Ulrich REH. Therapeutische Wirksamkeit und neue Daten zur Pharmakokinetik von Acitretin [in German]. Dt Dermatol. 1994;42:1316-27.

37. Friedman D, Weller S, Dix L. Acyclovir plasma concentrations and duration of herpes zoster pain: higher levels associated with the greater efficacy achieved with Valtrex (valacyclovir HCl). In: 34. Int. Conf. AAC; 1993:A72.

38. Morse GD, Shelton MJ, O'Donnell AM. Comparative pharmacokinetics of antiviral nucleoside analogues. Clin Pharmacokinet. 1993;24:101-23.

39. Shibata N, Kitamura A, Yoshikawa Y, Inoue T, Bamba T, Takada K. Simultaneous determination of aciclovir and ganciclovir in plasma by HPLC and pharmacokinetic interactions. Pharm Pharmacol Commun. 2000;6:501-6.

40. Committee for Medicinal Products for Human Use (CHMP). Humira (adalimumab) EPAR - Product information. [European Medicines Agency] https://www.ema.europa.eu/en/medicines/human/EPAR/humira. Accessed 17 Feb 2020.

41. Sonntag O. Arzneimittel-Interferenzen [in German]. Stuttgart - New York: Thieme; 1985.

42. Repetto MR, Repetto M. Therapeutic, toxic, and lethal concentrations in human fluids of 90 drugs affecting the cardiovascular and hematopoietic systems. J Toxicol Clin Toxicol. 1997;35(4):345-51.

43. Dinnendahl V, Fricke U. Arzneistoff-Profile. Basisinformation über arzneiliche Wirkstoffe [in German]. Eschborn: Govi; 2006.

44. Marriner SE, Morris DL, Dickson B, Bogan JA. Pharmacokinetics of albendazole in man. Eur J Clin Pharmacol. 1986;30:705-8.

45. Mirfazaelian A, Dadashzadeh S, Rouini MR. An HPLC method for determination of albendazole main metabolites. Pharm Pharmacol Commun. 2000;6:563-6.

46. Zeugin T, Zysset T, Cotting J. Therapeutic monitoring of albendazole: a high-performance liquid chromatography method for determination of its active metabolite albendazole sulfoxide. Ther Drug Monit. 1990;12:187-90.

47. Baselt RC. Disposition of toxic drugs and chemicals in man. 11th ed. Seal Beach: Biomedical Publications; 2017.

48. Winek CL, Wahba WW, Winek CL, Jr., Balzer TW. Drug and chemical blood-level data 2001. Forensic Sci Int. 2001;122:107-23.

49. Cremers SC, Pillai G, Papapoulos SE. Pharmacokinetics/pharmacodynamics of bisphosphonates: use for optimisation of intermittent therapy for osteoporosis. Clin Pharmacokinet. 2005;44:551-70.

50. Porras AG, Holland SD, Gertz BJ. Pharmacokinetics of alendronate. Clin Pharmacokinet. 1999;36(5):315-28.

51. Cocquyt V, Kline WF, Gertz BJ, Van Belle SJ, Holland SD, DeSmet M, et al. Pharmacokinetics of intravenous alendronate. J Clin Pharmacol. 1999;39(4):385-93.

52. Lemmens HJ. Pharmacokinetic-pharmacodynamic relationships for opioids in balanced anaesthesia. Clin Pharmacokinet. 1995;29:231-42.

53. Maitre PO, Vozeh S, Heykants J, Thomson DA, Stanski DR. Population pharmacokinetics of alfentanil: the average dose-plasma concentration relationship and interindividual variability in patients. Anesthesiology. 1987;66:3-12.

54. Scholz J, Steinfath M, Schulz M. Clinical pharmacokinetics of alfentanil, fentanyl and sufentanil. An update. Clin Pharmacokinet. 1996;31(4):275-92.

55. Wada DR, Mandema JW. Context sensitive pharmacokinetics in anesthesia: application to alfentanil [Abstract]. Pharm Res. 1994;11:S-424.

56. Druid H, Holmgren P. A compilation of fatal and control concentrations of drugs in postmortem femoral blood. J Forensic Sci. 1997;42:79-87.

57. Repetto MR, Repetto M. Therapeutic, toxic, and lethal concentrations of 73 drugs affecting respiratory system in human fluids. J Toxicol Clin Toxicol. 1998;36(4):287-93.

58. Uges DRA. TIAFT reference blood level list of therapeutic and toxic substances. [http://www.gtfch.org/cms/images/stories/Updated_TIAFT_list_202005.pdf]

59. Klotz U, Laux G. Tranquillantien [in German]. Stuttgart: WVG; 1996.

60. Labbate LA, Pollack MH, Otto MW, Tesar GM, Rosenbaum JF. The relationship of alprazolam and clonazepam dose to steady-state concentration in plasma. J Clin Psychopharmacol. 1994;14:274-6.

61. Laurijssens BE, Greenblatt DJ. Pharmacokinetic-pharmacodynamic relationships for benzodiazepines. Clin Pharmacokinet. 1996;30:52-76.

62. Lesser IM, Lydiard RB, Antal E, Rubin RT, Ballenger JC, DuPont R. Alprazolam plasma concentrations and treatment response in panic disorder and agoraphobia. Am J Psychiatry. 1992;149:1556-62.

63. Michaud K, Augsburger M, Romain N, Giroud C, Mangin P. Fatal overdose of tramadol and alprazolam. Forensic Sci Int. 1999;105(3):185-9.

64. Flanagan RJ. Guidelines for the interpretation of analytical toxicology results and unit of measurement conversion factors. Ann Clin Biochem. 1998;35:261-7.

65. N.N. Aluminium [in German]. Bundesgesundhbl. 1998;41(6):271.

66. Repetto MR, Repetto M. Concentrations in human fluids: 101 drugs affecting the digestive system and metabolism. J Toxicol Clin Toxicol. 1999;37(1):1-9.

67. Butler DR, Kuhn RJ, Chandler MH. Pharmacokinetics of anti-infective agents in paediatric patients. Clin Pharmacokinet. 1994;26:374-95.

68. Stork CM, Hoffman RS. Characterization of 4-aminopyridine in overdose. J Toxicol Clin Toxicol. 1994;32: 583-7.

69. Bouillon T, Bartmus D, Schiffmann H, Gundert-Remy U. Amiodaron zur Therapie ventrikulärer Arrhythmien bei einem Neugeborenen. Computergestützte pharmakokinetische Analyse zur Dosisfindung [in German]. Arzneimitteltherapie 1994;12:151-4.

70. Jürgens G, Graudal NA, Kampmann JP. Therapeutic drug monitoring of antiarrhythmic drugs. Clin Pharmacokinet. 2003;42:647-63.

71. Sauro SC, DeCarolis DD, Pierpont GL, Gornick CC. Comparison of plasma concentrations for two amiodarone products. Ann Pharmacother. 2002;36:1682-5.

72. Mauri MC, Paletta S, Di Pace C, Reggiori A, Cirnigliaro G, Valli I, Altamura AC. Clinical pharmacokinetics of atypical antipsychotics: an update. Clin Pharmacokinet. 2018;57(12):1493-528.

73. Boehnert MT, Lovejoy FH, Jr. Value of the QRS duration versus the serum drug level in predicting seizures and ventricular arrhythmias after an acute overdose of tricyclic antidepressants. N Engl J Med. 1985;313:474-9.

74. Breyer-Pfaff U, Gaertner HJ. Antidepressiva. Pharmakologie, therapeutischer Einsatz und Klinik der Depression [in German]. Stuttgart: WVG; 1987.

75. el-Yazigi A, Chaleby K, Gad A, Raines DA. Steady-state kinetics of fluoxetine and amitriptyline in patients treated with a combination of these drugs as compared with those treated with amitriptyline alone. J Clin Pharmacol. 1995;35:17-21.

76. Furlanut M, Benetello P, Spina E. Pharmacokinetic optimisation of tricyclic antidepressant therapy. Clin Pharmacokinet. 1993;24:301-18.

77. Hanzlick RL. Postmortem blood concentrations of parent tricyclic antidepressant (TCA) drugs in 11 cases of suicide. Am J Forensic Med Pathol. 1984;5:11-3.

78. Lieberman JA, Cooper TB, Suckow RF, Steinberg H, Borenstein M, Brenner R, et al. Tricyclic antidepressant and metabolite levels in chronic renal failure. Clin Pharmacol Ther. 1985;37:301-7.

79. Lieberman JA, Cooper TB, Suckow RF, Steinberg H, Borenstein M, Brenner R, et al. Tricyclic antidepressant drug and metabolite levels in chronic renal failure. Ann N Y Acad Sci. 1986;463:304-6.

80. Linder MW, Keck PE, Jr. Standards of laboratory practice: antidepressant drug monitoring. National Academy of Clinical Biochemistry. Clin Chem. 1998;44:1073-84.

81. Miljkovic B, Pokrajac M, Timotijevic I, Varagic V. Clinical response and plasma concentrations of amitriptyline and its metabolite-nortriptyline in depressive patients. Eur J Drug Metab Pharmacokinet. 1996;21:251-5.

82. Preskorn SH, Fast GA. Therapeutic drug monitoring for antidepressants: efficacy, safety, and cost effectiveness. J Clin Psychiatry. 1991;52(Suppl.):23-33.

83. Ulrich S, Läuter J. Comprehensive survey of the relationship between serum concentration and therapeutic effect of amitriptyline in depression. Clin Pharmacokinet. 2002;41:853-76.

84. Kirsten R, Nelson K, Kirsten D, Heintz B. Clinical pharmacokinetics of vasodilators. Part I. Clin Pharmacokinet. 1998;34:457-82.

85. Koch AR, Vogelaers DP, Decruyenaere JM, Callens B, Verstraete A, Buylaert WA. Fatal intoxication with amlodipine. J Toxicol Clin Toxicol. 1995;33:253-6.

86. Stanek EJ, Nelson CE, DeNofrio D. Amlodipine overdose. Ann Pharmacother. 1997;31:853-6.

87. Musshoff F, Padosch S, Steinborn S, Madea B. Fatal blood and tissue concentrations of more than 200 drugs. Forensic Sci Int. 2004;142:161-210.

88. Sweetman SC. Martindale. The complete drug reference. 33 ed. London: Pharmaceutical Press; 2002.

89. Hellriegel ET, Arora S, Nelson M, Robertson P, Jr. Steady-state pharmacokinetics and tolerability of modafinil administered alone or in combination with dextroamphetamine in healthy volunteers. J Clin Pharmacol. 2002;42:450-60.

90. Cleary JD, Hayman J, Sherwood J, Lasala GP, Piazza-Hepp T. Amphotericin B overdose in pediatric patients with associated cardiac arrest. Ann Pharmacother. 1993;27:715-9.

91. Hay RJ. Recent advances in the management of fungal infections. Q J Med. 1987;64:631-9.

92. Lipp H-P. Amphotericin B und seine Lipidcarrier. Eine kritische Übersicht [in German]. Krankenhauspharmazie. 1997;18:104-13.

93. Mohr JF, Hall AC, Ericsson CD, Ostrosky-Zeichner L. Fatal amphotericin B overdose due to administration of nonlipid formulation instead of lipid formulation. Pharmacotherapy. 2005;25:426-8.

94. Hellinger A, Wolter K, Marggraf G, Pentz R, Fritschka E. Elimination of amrinone during continuous veno-venous haemofiltration after cardiac surgery. Eur J Clin Pharmacol. 1995;48:57-9.

95. Kirsten R, Nelson K, Kirsten D, Heintz B. Clinical pharmacokinetics of vasodilators. Part II. Clin Pharmacokinet. 1998;35:9-36.

96. Paxton JW, Kim SN, Whitfield LR. Pharmacokinetic and toxicity scaling of the antitumor agents amsacrine and CI-921, a new analogue, in mice, rats, rabbits, dogs, and humans. Cancer Res. 1990;50:2692-7.

97. N.N. Stoffmonographie und Referenzwerte für monocyclische Aminoaromaten im Urin. Stellungnahme der Kommission Human-Biomonitoring des Umweltbundesamtes [in German]. Bundesgesundhbl. 2011;54:650-63.

98. Iwersen-Bergmann S, Schmoldt A. Acute intoxication with aniline: detection of acetaminophen as aniline metabolite. Int J Legal Med. 2000;113(3):171-4.

99. Kütting B, Goen T, Schwegler U, Fromme H, Uter W, Angerer J, et al. Monoarylamines in the general population--a cross-sectional population-based study including 1004 Bavarian subjects. Int J Hyg Environ Health. 2009;212:298-309.

100. Schulz C, Angerer J, Ewers U, Heudorf U, Wilhelm M. Revised and new reference values for environmental pollutants in urine or blood of children in Germany derived from the German environmental survey on children 2003-2006 (GerES IV). Int J Hyg Environ Health. 2009;212:637-47.

101. Choong E, Rudaz S, Kottelat A, Guillarme D, Veuthey JL, Eap CB. Therapeutic drug monitoring of seven psychotropic drugs and four metabolites in human plasma by HPLC-MS. J Pharm Biomed Anal. 2009;50:1000-8.

102. Young MC, Shah N, Cantrell FL, Clark RF. Risk assessment of isolated aripiprazole exposures and toxicities: a retrospective study. Clin Toxicol (Phila). 2009;47:580-3.

103. Duenas-Laita A, Perez-Miranda M, Gonzalez-Lopez MA, Martin-Escudero JC, Ruiz-Mambrilla M, Blanco-Varela J. Acute arsenic poisoning. Lancet. 2005;365:1982.

104. Oertel R, Rahn R, Kirch W. Clinical pharmacokinetics of articaine. Clin Pharmacokinet. 1997;33:417-25.

105. Biesalski HK. Antioxidative Vitamine in der Prävention [in German]. Dt Ärztebl. 1995;92:A-1316.

106. Jacob RA. Assessment of human vitamin C status. J Nutr. 1990;120(Suppl. 11):1480-5.

107. Lykkesfeldt J, Prieme H, Loft S, Poulsen HE. Effect of smoking cessation on plasma ascorbic acid concentration. BMJ. 1996;313:91.

108. Wang S, Schram IM, Sund RB. Determination of plasma ascorbic acid by HPLC: method and stability studies. Eur J Pharm Sci. 1995;3:231-9.

109. Snook J, Boothman-Burrell D, Watkins J, Colin-Jones D. Torsade de pointes ventricular tachycardia associated with astemizole overdose. Br J Clin Pract. 1988;42:257-9.

110. HHS Panel on Antiretroviral Guidelines for Adults and Adolescents [Guidelines for the use of antiretroviral agents in HIV-1-infected adults and adolescents]. [Department of Health & Human Services, USA]. https://aidsinfo.nih.gov/contentfiles/lvguidelines/AdultandAdolescentGL.pdf. Accessed 25 Feb 2020.

111. von Hentig N. Messung von Plasmakonzentrationen antiretroviraler Arzneimittel in der HIV-Therapie [in German]. Dtsch Med Wochenschr. 2008;133:191-5.

112. Stoschitzky K, Kahr S, Donnerer J, Schumacher M, Luha O, Maier R, et al. Stereoselective increase of plasma concentrations of the enantiomers of

propranolol and atenolol during exercise. Clin Pharmacol Ther. 1995;57:543-51.

113. Spencer CM, Goa KL. Atovaquone. A review of its pharmacological properties and therapeutic efficacy in opportunistic infections. Drugs. 1995;50:176-96.

114. Tune L, Coyle JT. Serum levels of anticholinergic drugs in treatment of acute extrapyramidal side effects. Arch Gen Psychiatry. 1980;37:293-7.

115. Bahal N, Nahata MC. The new macrolide antibiotics: azithromycin, clarithromycin, dirithromycin, and roxithromycin. Ann Pharmacother. 1992;26:46-55.

116. Lode H. The pharmacokinetics of azithromycin and their clinical significance. Eur J Clin Microbiol Infect Dis. 1991;10:807-12.

117. Peters DH, Friedel HA, McTavish D. Azithromycin. A review of its antimicrobial activity, pharmacokinetic properties and clinical efficacy. Drugs. 1992;44:750-99.

118. Rodvold KA, Gotfried MH, Danziger LH, Servi RJ. Intrapulmonary steady-state concentrations of clarithromycin and azithromycin in healthy adult volunteers. Antimicrob Agents Chemother. 1997;41:1399-402.

119. Schulz M, Peruche B. Azithromycin, ein neues Makrolid-Antibiotikum [in German]. Pharm Ztg. 1994;139(40):3346-52.

120. Hiller JL, Benda GI, Rahatzad M, Allen JR, Culver DH, Carlson CV, et al. Benzyl alcohol toxicity: impact on mortality and intraventricular hemorrhage among very low birth weight infants. Pediatrics.1986;77:500-6.

121. Lopez-Herce J, Bonet C, Meana A, Albajara L. Benzyl alcohol poisoning following diazepam intravenous infusion. Ann Pharmacother. 1995;29:632.

122. Hakamäki T, Apoil E, Arstila M, Timmer CJ, Lehtonen A. Bepridil in the elderly. A pharmacokinetic and clinical monitoring study. Curr Ther Res. 1988;44:752-8.

123. Gilissen LP, Wong DR, Engels LG, Bierau J, Bakker JA, Paulussen AD, et al. Therapeutic drug monitoring of thiopurine metabolites in adult thiopurine tolerant IBD patients on maintenance therapy. J Crohns Colitis. 2012;6(6):698-707.

124. Greenberg ER, Baron JA, Karagas MR, Stukel TA, Nierenberg DW, Stevens MM, et al. Mortality associated with low plasma concentration of beta carotene and the effect of oral supplementation. JAMA. 1996;275:699-703.

125. Berthault F, Kintz P, Tracqui A, Mangin P. A fatal case of betaxolol poisoning. J Anal Toxicol. 1997;21:228-31.

126. Mahler C, Verhelst J, Denis L. Clinical pharmacokinetics of the antiandrogens and their efficacy in prostate cancer. Clin Pharmacokinet. 1998;34:405-17.

127. Palmer RB, Alakija P, de Baca JE, Nolte KB. Fatal brodifacoum rodenticide poisoning: autopsy and toxicologic findings. J Forensic Sci. 1999;44:851-5.

128. Danel VC, Saviuc PF, Hardy GA, Lafond JL, Mallaret MP. Bromide intoxication and pseudohyperchloremia. Ann Pharmacother. 2001;35:386-7.

129. Hoizey G, Souchon PF, Trenque T, Frances C, Lamiable D, Nicolas A, et al. An unusual case of methyl bromide poisoning. J Toxicol Clin Toxicol. 2002;40:817-21.

130. Stein U, Steinecke H, Pragst F, Prügel M, Ulrich P, Gondro T. Ionenselektive Elektroden und Mikrodestillation [in German]. Toxichem Krimtech. 1999;66:129-41.

131. Saito T, Takeichi S, Nakajima Y, Yukawa N, Osawa M. A case of homicidal poisoning involving several drugs. J Anal Toxicol. 1997;21:584-6.

132. Hempel V, Lenz G. Lokalanästhetika - Wirkungsweise, Eigenschaften, Pharmakokinetik und Toxizität [in German]. Anästh Intesivmed. 1982;23:337-45.

133. Kastrissios H, Triggs EJ, Sinclair F, Moran P, Smithers M. Plasma concentrations of bupivacaine after wound infiltration of an 0.5% solution after inguinal herniorrhaphy: a preliminary study. Eur J Clin Pharmacol. 1993;44:555-7.

134. Lenderink AW, Langen MCJ, Schippers D. Bupivacain serum levels after intra-abdominal instillation [Abstract]. Pharm World Sci. 1994;16:D8.

135. Elkader A, Sproule B. Buprenorphine: clinical pharmacokinetics in the treatment of opioid dependence. Clin Pharmacokinet. 2005;44:661-80.

136. Kuhlman JJ, Jr., Levine B, Johnson RE, Fudala PJ, Cone EJ. Relationship of plasma buprenorphine and norbuprenorphine to withdrawal symptoms during dose induction, maintenance and withdrawal from sublingual buprenorphine. Addiction. 1998;93:549-59.

137. Tracqui A, Kintz P, Ludes B. Buprenorphine-related deaths among drug addicts in France: a report on 20 fatalities. J Anal Toxicol. 1998;22:430-4.

138. Walsh SL, Preston KL, Stitzer ML, Cone EJ, Bigelow GE. Clinical pharmacology of buprenorphine: ceiling effects at high doses. Clin Pharmacol Ther. 1994;55:569-80.

139. Kintz P. Deaths involving buprenorphine: a compendium of French cases. Forensic Sci Int. 2001;121:65-9.

140. Kuhlman JJ, Jr., Lalani S, Magluilo J, Jr., Levine B, Darwin WD. Human pharmacokinetics of intravenous, sublingual, and buccal buprenorphine. J Anal Toxicol. 1996;20:369-78.

141. Lai SH, Yao YJ, Lo DS. A survey of buprenorphine related deaths in Singapore. Forensic Sci Int. 2006;162:80-6.

142. Findlay JWA, Van Wyck FJ, Smith PG, Butz RF, Hinton ML, Blum MR, et al. Pharmacokinetics of bupropion, a novel antidepressant agent, following oral administration to healthy subjects. Eur J Clin Pharmacol. 1981;21:127-35.

143. Friel PN, Logan BK, Fligner CL. Three fatal drug overdoses involving bupropion. J Anal Toxicol. 1993:17:436-8.

144. Holm KJ, Spencer CM. Bupropion: a review of its use in the management of smoking cessation. Drugs. 2000;59:1007-24.

145. Hsyu PH, Singh A, Giargiari TD, Dunn JA, Ascher JA, Johnston JA. Pharmacokinetics of bupropion and its metabolites in cigarette smokers versus nonsmokers. J Clin Pharmacol. 1997,37:737-43.

146. Lai AA, Schroeder DH. Clinical pharmacokinetics of bupropion: a review. J Clin Psychiatry. 1983;44:82-4.

147. Posner J, Bye A, Dean K, Peck AW, Whiteman PD. The disposition of bupropion and its metabolites in healthy male volunteers after single and multiple doses. Eur J Clin Pharmacol. 1985;29:97-103.

148. Sweet RA, Pollock BG, Kirshner M, Wright B, Altieri LP, DeVane CL. Pharmacokinetics of single- and multiple-dose bupropion in elderly patients with depression. J Clin Pharmacol. 1995;35:876-84.

149. Cull G, O'Halloran S, Ilett KF. Therapeutic drug monitoring for busulfan in plasma during conditioning chemotherapy for autologous stem cell transplantation in relapsed primary cerebral lymphoma. Ther Drug Monit. 2010;32:333-7.

150. Juenke JM, Miller KA, McMillin GA, Johnson-Davis KL. An automated method for supporting busulfan therapeutic drug monitoring. Ther Drug Monit. 2011;33:315-20.

151. Malar R, Sjoo F, Rentsch K, Hassan M, Gungor T. Therapeutic drug monitoring is essential for intravenous busulfan therapy in pediatric hematopoietic stem cell recipients. Pediatr Transplant. 2011;15:580-8.

152. Radich JP, Gooley T, Bensinger W, Chauncey T, Clift R, Flowers M, et al. HLA-matched related hematopoietic cell transplantation for chronic-phase CML using a targeted busulfan and cyclophosphamide preparative regimen. Blood. 2003;102:31-5.

153. Slattery JT, Clift RA, Buckner CD, Radich J, Storer B, Bensinger WI, et al. Marrow transplantation for chronic myeloid leukemia: the influence of plasma busulfan levels on the outcome of transplantation. Blood. 1997;89:3055-60.

154. Yeh RF, Pawlikowski MA, Blough DK, McDonald GB, O'Donnell PV, Rezvani A, et al. Accurate Targeting of Daily Intravenous Busulfan with 8-Hour Blood

Sampling in Hospitalized Adult Hematopoietic Cell Transplant Recipients. Biol Blood Marrow Transplant. 2012;18(29):265-72.

155. Cook DG, Peacock JL, Feyerabend C, Carey IM, Jarvis MJ, Anderson HR, et al. Relation of caffeine intake and blood caffeine concentrations during pregnancy to fetal growth: prospective population based study. BMJ. 1996;313:1358-62.

156. Mizuno A, Uematsu T, Gotoh S, Katoh E, Nakashima M. The measurement of caffeine concentration in scalp hair as an indicator of liver function. J Pharm Pharmacol. 1996;48:660-4.

157. Risselmann B, Rosenbaum F, Roscher S, Schneider V. Fatal caffeine intoxication. Forensic Sci Int. 1999;103:S49-52.

158. Köppel C, Martens F, Schirop T, Ibe K. Hemoperfusion in acute camphor poisoning. Intensive Care Med. 1988;14:431-3.

159. Alderman CP. Adverse effects of the angiotensin-converting enzyme inhibitors. Ann Pharmacother. 1996;30:55-61.

160. Schulz M, Graefe T, Stuby K, Andresen H, Kupfermann N, Schmoldt A. Case report: acute unintentional carbachol intoxication. Crit Care. 2006;10:R84.

161. Brodie MJ, Dichter MA. Antiepileptic drugs. N Engl J Med. 1996;334:168-75.

162. Collins DM, Gidal BE, Pitterle ME. Potential interaction between carbamazepine and loxapine: case report and retrospective review. Ann Pharmacother. 1993;27:1180-7.

163. Elmquist WF, Riad LE, Leppik IE, Sawchuk RJ. The relationship between urine and plasma concentrations of carbamazepine: implications for therapeutic drug monitoring. Pharm Res. 1991;8:282-4.

164. French J. The long-term therapeutic management of epilepsy. Ann Intern Med. 1994;120:411-22.

165. Kale PB, Thomson PA, Provenzano R, Higgins MJ. Evaluation of plasmapheresis in the treatment of an acute overdose of carbamazepine. Ann Pharmacother. 1993;27:866-70.

166. Liu H, Delgado MR. Therapeutic drug concentration monitoring using saliva samples. Focus on anticonvulsants. Clin Pharmacokinet. 1999;36:453-70.

167. Duck BJ, Woolias M. Reversed-phase high performance liquid chromatographic determination of carbaryl in postmortem specimens. J Anal Toxicol. 1985;9:177-9.

168. Stockis A, Deroubaix X, Jeanbaptiste B, Lins R, Allemon AM, Laufen H. Relative bioavailability of carbinoxamine and phenylephrine from a retard capsule after single and repeated dose administration in healthy subjects. Arzneimittelforschung. 1995;45:1009-12.

169. Schmoldt A, Schulz M, Frese JH. Klinik und Therapie einer Intoxikation mit Tetrachlorkohlenstoff [Course and therapy of a carbon tetrachloride intoxication] [in German]. In: Gerichtsmedizin. Festschrift für Wilhelm Holczabek. Edited by Bauer G. Wien: Franz Deuticke; 1988. p. 529-31.

170. Goldermann L, Gellert J, Teschke R. Quantitative assessment of carbon tetrachloride levels in human blood by head-space gas chromatography: application in a case of suicidal carbon tetrachloride intoxication. Intensive Care Med. 1983;9:131-5.

171. Mathieson PW, Williams G, MacSweeney JE. Survival after massive ingestion of carbon tetrachloride treated by intravenous infusion of acetylcysteine. Hum Toxicol. 1985;4:627-31.

172. Ruprah M, Mant TG, Flanagan RJ. Acute carbon tetrachloride poisoning in 19 patients: implications for diagnosis and treatment. Lancet. 1985;1:1027-9.

173. Tombolini A, Cingolani M. Fatal accidental ingestion of carbon tetrachloride: a postmortem distribution study. J Forensic Sci. 1996;41:166-8.

174. Danziger LH, Piscitelli SC, Occhipinti DJ, Resnick DJ, Rodvold KA. Steady-state pharmacokinetics of cefoperazone and sulbactam in patients with acute appendicitis. Ann Pharmacother. 1994;28:703-7.

175. Schulz M, Schmoldt A. Konzentrationen von Cefotiam im Kolongewebe und Plasma nach Applikation zur perioperativen Antibiotikaprophylaxe [Concentrations of cefotiam in colon tissue and plasma after application for perioperative antibiotic prophylaxis] [in German]. ZAC Zeitschr antimikr antineopl Chemother. 1992;10:33-7.

176. Lorenz R, Lehn N, Born P, Herrmann M, Neuhaus H. Antibiotische Prophylaxe mit Cefuroxim bei endoskopischen Eingriffen an den Gallenwegen [in German]. Dtsch Med Wochenschr. 1996;121:223-30.

177. Pass SE, Miyagawa CI, Healy DP, Ivey TD. Serum concentrations of cefuroxime after continuous infusion in coronary bypass graft patients. Ann Pharmacother. 2001;35:409-13.

178. Koren G. Therapeutic drug monitoring principles in the neonate. National Academy of CLinical Biochemistry. Clin Chem. 1997;43:222-7.

179. Bailey DN. Blood concentrations and clinical findings following overdose of chlordiazepoxide alone and chlordiazepoxide plus ethanol. J Toxicol Clin Toxicol. 1984;22:433-46.

180. Maxa JL, Ogu CC, Adeeko MA, Swaner TG. Continuous-infusion flumazenil in the management of chlordiazepoxide toxicity. Pharmacotherapy. 2003;23:1513-6.

181. Köppel C, Kristinsson J, Wagemann A, Tenczer J, Martens F. Chlormezanone plasma and blood levels in patients after single and repeated oral doses and after suicidal drug overdose. Eur J Drug Metab Pharmacokinet. 1991;16:43-7.

182. Dell'Aglio DM, Sutter ME, Schwartz MD, Koch DD, Algren DA, Morgan BW. Acute chloroform ingestion successfully treated with intravenously administered N-acetylcysteine. J Med Toxicol. 2010;6:143-6.

183. Croes K, Augstijns P, Sabbe M, Desmet K, Verbeke N. Diazepam treatment in chloroquine intoxication: a case report [Abstract]. Pharm Weekbl Sci. 1992;14:D9.

184. Javaid JI. Clinical pharmacokinetics of antipsychotics. J Clin Pharmacol. 1994;34:286-95.

185. Milton GV, Jann MW. Emergency treatment of psychotic symptoms. Pharmacokinetic considerations for antipsychotic drugs. Clin Pharmacokinet. 1995;28:494-504.

186. Eddleston M, Eyer P, Worek F, Mohamed F, Senarathna L, von Meyer L, et al. Differences between organophosphorus insecticides in human self-poisoning: a prospective cohort study. Lancet. 2005;366:1452-9.

187. Vaughan Williams EM. Classifying antiarrhythmic actions: by facts or speculation. J Clin Pharmacol. 1992;32:964-77.

188. Dumont RJ, Ensom MH. Methods for clinical monitoring of cyclosporin in transplant patients. Clin Pharmacokinet. 2000;38:427-47.

189. Horton RC, Bonser RS. Interaction between cyclosporin and fluoxetine. BMJ. 1995;311:422.

190. Lindholm A. Cyclosporine A: clinical experience and therapeutic drug monitoring. Ther Drug Monit. 1995;17:631-7.

191. Lindholm A, Sawe J. Pharmacokinetics and therapeutic drug monitoring of immunosuppressants. Ther Drug Monit. 1995;17:570-3.

192. Oellerich M, Armstrong VW, Kahan B, Shaw L, Holt DW, Yatscoff R, et al. Lake Louise Consensus Conference on cyclosporin monitoring in organ transplantation: report of the consensus panel. Ther Drug Monit. 1995;17:642-54.

193. Tonkin AL, Bochner F. Therapeutic drug monitoring and patient outcome. A review of the issues. Clin Pharmacokinet. 1994;27:169-74.

194. Gugler R, Fuchs G, Dieckmann M, Somogyi AA. Cimetidine plasma concentration-response relationships. Clin Pharmacol Ther. 1981;29:744-8.

195. Shinn AF. Clinical relevance of cimetidine drug interactions. Drug Saf. 1992;7:245-67.

196. Cohen H, Francisco DH. Twelve-gram overdose of ciprofloxacin with mild symptomatology. Ann Pharmacother. 1994;28:805-6.

197. Davis JD, Aarons L, Houston JB. Relationship between enoxacin and ciprofloxacin plasma concentrations and theophylline disposition. Pharm Res. 1994;11:1424-8.

198. Schentag JJ, Nix DE, Adelman MH. Mathematical examination of dual individualization principles (I): Relationships between AUC above MIC and area under the inhibitory curve for cefmenoxime, ciprofloxacin, and tobramycin. DICP. 1991;25:1050-7.

199. Staß H, Peltola H, Kuhlmann J, Rahm V. Single dose and steady state pharmacokinetics of ciprofloxacin (CIP) in pediatric patients following administration of a new oral suspension (10 mg/kg tid) [Abstract]. Naunyn-Schmiedeberg's Arch Pharmacol. 1996;353:R153.

200. Friberg LE, Isbister GK, Hackett LP, Duffull SB. The population pharmacokinetics of citalopram after deliberate self-poisoning: a Bayesian approach. J Pharmacokinet Pharmacodyn. 2005;32:571-605.

201. Gutierrez M, Abramowitz W. Steady-state pharmacokinetics of citalopram in young and elderly subjects. Pharmacotherapy. 2000;20:1441-7.

202. van Harten J. Clinical pharmacokinetics of selective serotonin reuptake inhibitors. Clin Pharmacokinet. 1993;24:203-20.

203. Jaehde U, Sorgel F, Reiter A, Sigl G, Naber KG, Schunack W. Effect of probenecid on the distribution and elimination of ciprofloxacin in humans. Clin Pharmacol Ther. 1995;58:532-41.

204. Öström M, Eriksson A, Thorson J, Spigset O. Fatal overdose with citalopram. Lancet. 1996;348:339-40.

205. Overo KF. Preliminary studies of the kinetics of citalopram in man. Eur J Clin Pharmacol. 1978;14:69-73.

206. Overo KF. Kinetics of citalopram in man; plasma levels in patients. Prog Neuropsychopharmacol Biol Psychiatry. 1982;6:311-8.

207. Overo KF, Toft B, Christophersen L, Gylding-Sabroe JP. Kinetics of citalopram in elderly patients. Psychopharmacology (Berl). 1985;86:253-7.

208. Beutler E. Cladribine (2-chlorodeoxyadenosine). Lancet. 1992;340:952-6.

209. Johnson SA. Clinical pharmacokinetics of nucleoside analogues: focus on haematological malignancies. Clin Pharmacokinet. 2000;39:5-26.

210. Kath R, Knauf WU, Mitrou PS, Rummel M, Höffken K, Peters HD. Cladribin (2-CdA). Pharmakologisches Profil und klinische Anwendung [in German]. Onkologe. 1995;1:626.

211. Boruchoff SE, Sturgill MG, Grasing KW, Seibold JR, McCrea J, Winchell GA, et al. The steady-state disposition of indinavir is not altered by the concomitant administration of clarithromycin. Clin Pharmacol Ther. 2000;67:351-9.

212. Rodvold KA. Clinical pharmacokinetics of clarithromycin. Clin Pharmacokinet. 1999;37:385-98.

213. Schulz J. Clarithromycin - ein neues Makrolid-Antibiotikum [in German]. Pharm Ztg. 1992;137:1626-31.

214. Breccia A, Ferri E, Girotti S, Bignanmini AA, Budini RA. High performance liquid chromatography and capillary gas chromatography-mass spectrometry determination of clemastine in serum: plasma kinetics after dermatologic application. Curr Ther Res. 1991;49:622-6.

215. Knapp J, Boknik P, Gumbinger HG, Linck B, Luss H, Muller FU, et al. Quantitation of clobazam in human plasma using high-performance liquid chromatography. J Chromatogr Sci. 1999;37:145-9.

216. Klug E, Schneider V. Vergiftungen durch Clomethiazol [in German]. Z Rechtsmed. 1984;93:89-94.

217. Ulrich S, Danos P, Baumann B, Muller D, Lehmann D, Treuheit TO, et al. Serum concentration of chlormethiazole and therapeutic effect in acute alcohol withdrawal syndrome: an open clinical trial. Ther Drug Monit. 2002;24:446-54.

218. Balant-Gorgia AE, Gex-Fabry M, Balant LP. Clinical pharmacokinetics of clomipramine. Clin Pharmacokinet. 1991;20:447-62.

219. Dale O, Hole A. Biphasic time-course of serum concentrations of clomipramine and desmethylclomipramine after a near-fatal overdose. Vet Hum Toxicol. 1994;36:309-10.

220. Faravelli C, Ballerini A, Ambonetti A, Broadhurst AD, Das M. Plasma levels and clinical response during treatment with clomipramine. J Affect Disord. 1984;6:95-107.

221. Gex-Fabry M, Balant-Gorgia AE, Balant LP. Clomipramine concentration as a predictor of delayed response: a naturalistic study. Eur J Clin Pharmacol. 1999;54:895-902.

222. Kuss HJ, Jungkunz G. Nonlinear pharmacokinetics of chlorimipramine after infusion and oral administration in patients. Prog Neuropsychopharmacol Biol Psychiatry. 1986;10:739-48.

223. Waade RB, Molden E, Refsum H, Hermann M. Serum concentrations of antidepressants in the elderly. Ther Drug Monit. 2012;34:25-30.

224. Rey E, Treluyer JM, Pons G. Pharmacokinetic optimization of benzodiazepine therapy for acute seizures. Focus on delivery routes. Clin Pharmacokinet. 1999;36:409-24.

225. Erickson SJ, Duncan A. Clonidine poisoning--an emerging problem: epidemiology, clinical features, management and preventative strategies. J Paediatr Child Health. 1998;34:280-2.

226. Nichols MH, King WD, James LP. Clonidine poisoning in Jefferson County, Alabama. Ann Emerg Med. 1997;29:511-7.

227. Raber JH, Shinar C, Finkelstein S. Clonidine patch ingestion in an adult. Ann Pharmacother. 1993;27:719-22.

228. Couchman L, Morgan PE, Spencer EP, Flanagan RJ. Plasma clozapine, norclozapine, and the clozapine:norclozapine ratio in relation to prescribed dose and other factors: data from a therapeutic drug monitoring service, 1993-2007. Ther Drug Monit. 2010;32:438-47.

229. Dahl SG. Pharmacokinetics of antipsychotic drugs in man. Acta Psychiatr Scand. 1990;358(Suppl.):37-40.

230. Dettling M, Sachse C, Brockmoller J, Schley J, Muller-Oerlinghausen B, Pickersgill I, et al. Long-term therapeutic drug monitoring of clozapine and metabolites in psychiatric in- and outpatients. Psychopharmacology (Berl). 2000;152:80-6.

231. Fleischhaker C, Schulz E, Clement H-W, Krieg C, Remschmidt H. Therapeutisches Drug-Monitoring von Clozapin bei Kindern, Jugendlichen und Erwachsenen mit einer schizophrenen Psychose [in German]. Psychopharmakotherapie. 1999;6:102-5.

232. Guitton C, Kinowski JM, Abbar M, Chabrand P, Bressolle F. Clozapine and metabolite concentrations during treatment of patients with chronic schizophrenia. J Clin Pharmacol. 1999;39:721-8.

233. Ismail Z, Wessels AM, Uchida H, Ng W, Mamo DC, Rajji TK, et al. Age and sex impact clozapine plasma concentrations in inpatients and outpatients with schizophrenia. Am J Geriatr Psychiatry. 2012;20:53-60.

234. Mahoney MC, Connolly BF, Smith CM. A clozapine overdose with markedly elevated serum levels. J Clin Pharmacol. 1999;39:97-100.

235. Schulte P. What is an adequate trial with clozapine? Therapeutic drug monitoring and time to response in treatment-refractory schizophrenia. Clin Pharmacokinet. 2003;42:607-18.

236. Spina E, Avenoso A, Facciola G, Scordo MG, Ancione M, Madia AG, et al. Relationship between plasma concentrations of clozapine and norclozapine and therapeutic response in patients with schizophrenia resistant to conventional neuroleptics. Psychopharmacology (Berl). 2000;148:83-9.

237. Ulrich S, Wolf R, Staedt J. Serum level of clozapine and relapse. Ther Drug Monit. 2003;25:252-5.

238. Van der Zwaag C, McGee M, McEvoy JP, Freudenreich O, Wilson WH, Cooper TB. Response of patients with treatment-refractory schizophrenia to clozapine within three serum level ranges. Am J Psychiatry. 1996;153:1579-84.

239. Wohlfarth A, Toepfner N, Hermanns-Clausen M, Auwarter V. Sensitive quantification of clozapine and its main metabolites norclozapine and clozapine-N-oxide in serum and urine using LC-MS/MS after simple liquid-liquid extraction work-up. Anal Bioanal Chem. 2011;400:737-46.

240. Zaleon CR, Guthrie SK. Antipsychotic drug use in older adults. Am J Hosp Pharm. 1994;51:2917-43.

241. Kronstrand R, Roman M, Thelander G, Eriksson A. Unintentional fatal intoxications with mitragynine and O-desmethyltramadol from the herbal blend Krypton. J Anal Toxicol. 2011;35(4):242-7.

242. Perez-Reyes M, Jeffcoat AR. Ethanol/cocaine interaction: cocaine and cocaethylene plasma concentrations and their relationship to subjective and cardiovascular effects. Life Sci. 1992;51:553-63.

243. Skeith KJ, Brocks DR. Pharmacokinetic optimisation of the treatment of osteoarthritis. Clin Pharmacokinet. 1994;26:233-42.

244. Meyer MR, Maurer HH. Absorption, distribution, metabolism and excretion pharmacogenomics of drugs of abuse. Pharmacogenomics. 2011;12(2):215-33.

245. Baud FJ, Sabouraud A, Vicaut E, Taboulet P, Lang J, Bismuth C, et al. Brief report: treatment of severe colchicine overdose with colchicine-specific Fab fragments. N Engl J Med. 1995;332:642-5.

246. Brvar M, Ploj T, Kozelj G, Mozina M, Noc M, Bunc M. Case report: fatal poisoning with Colchicum autumnale. Crit Care. 2004;8:R56-9.

247. Peters FT, Beyer J, Ewald AH. Colchicine poisoning after mix-up of Ramsons (Allium ursinum L.) and meadow saffron (Colchicum autumnale L.). Toxichem Krimtech. 2004;71:156-60.

248. Rochdi M, Sabouraud A, Baud FJ, Bismuth C, Scherrmann JM. Toxicokinetics of colchicine in humans: analysis of tissue, plasma and urine data in ten cases. Hum Exp Toxicol. 1992;11:510-6.

249. Grobosch T, Angelow B, Lampe D. Akute Intoxikation mit Coumatetralyl. Simultane Bestimmung von 5 Superwarfarinen und 5 weiteren Vitamin K-Antagonisten in Humanserum mittels LC-ESI-MS [in German]. Toxichem Krimtech. 2005;72:46-55.

250. Baud FJ, Borron SW, Bavoux E, Astier A, Hoffman JR. Relation between plasma lactate and blood cyanide concentrations in acute cyanide poisoning. BMJ. 1996;312:26-7.

251. Gracia R, Shepherd G. Cyanide poisoning and its treatment. Pharmacotherapy. 2004;24:1358-65.

252. Hall AH, Rumack BH. Clinical toxicology of cyanide. Ann Emerg Med. 1986;15:1067-74.

253. Houeto P, Hoffman JR, Imbert M, Levillain P, Baud FJ. Relation of blood cyanide to plasma cyanocobalamin concentration after a fixed dose of hydroxocobalamin in cyanide poisoning. Lancet. 1995;346:605-8.

254. Rindone JP, Sloane EP. Cyanide toxicity from sodium nitroprusside: risks and management. Ann Pharmacother. 1992;26:515-9.

255. Salkowski AA, Penney DG. Cyanide poisoning in animals and humans: a review. Vet Hum Toxicol. 1994;36:455-66.

256. Strehl E. Cyanid-Intoxikationen. Hydroxycobalamin bereichert das Antidotarium [in German]. Krankenhauspharmazie. 2000;21:293-7.

257. Winchell GA, King JD, Chavez-Eng CM, Constanzer ML, Korn SH. Cyclobenzaprine pharmacokinetics, including the effects of age, gender, and hepatic insufficiency. J Clin Pharmacol. 2002;42:61-9.

258. Belldina EB, Huang MY, Schneider JA, Brundage RC, Tracy TS. Steady-state pharmacokinetics and pharmacodynamics of cysteamine bitartrate in paediatric nephropathic cystinosis patients. Br J Clin Pharmacol. 2003;56:520-5.

259. USP DI® Volume I. Drug Information for the Health Care Professional. 26th edition. Greenwood Village, CO, USA: Thomson Reuters (Healthcare) Inc.; 2006.

260. Kraemer T, Paul LD, Jochum C, Maurer HH. Acute poisoning with dapsone - a case report. Toxichem Krimtech. 2002;69:80-5.

261. Piscitelli SC, Occhipinti DJ, Danziger LH, Hill C, West DP, Fischer JH. Therapeutic monitoring and pharmacist intervention in a Hansen's disease clinic. Ann Pharmacother. 1993;27:1526-31.

262. Fabbiani M, Bracciale L, Ragazzoni E, Santangelo R, Cattani P, Di GS, et al. Relationship between antiretroviral plasma concentration and emergence of HIV-1 resistance mutations at treatment failure. Infection. 2011;39:563-9.

263. Goeringer KE, Raymon L, Logan BK. Postmortem forensic toxicology of trazodone. J Forensic Sci. 2000;45(4):850-6.

264. von Moltke LL, Greenblatt DJ, Shader RI. Clinical pharmacokinetics of antidepressants in the elderly. Therapeutic implications. Clin Pharmacokinet. 1993;24:141-60.

265. Richter O, Ern B, Reinhardt D, Becker B. Pharmacokinetics of dexamethasone in children. Pediatr Pharmacol (New York). 1983;3:329-37.

266. Gunn VL, Taha SH, Liebelt EL, Serwint JR. Toxicity of over-the-counter cough and cold medications. Pediatrics. 2001;108:E52.

267. Hanzlick R. National Association of Medical Examiners Pediatric Toxicology (PedTox) Registry Report 3. Case submission summary and data for acetaminophen, benzene, carboxyhemoglobin, dextromethorphan, ethanol,

phenobarbital, and pseudoephedrine. Am J Forensic Med Pathol. 1995;16:270-7.

268. Härtter S, Baier D, Dingemanse J, Ziegler G, Hiemke C. Steady state pharmacokinetics of dextromethorphan [Abstract]. Naunyn-Schmiedeberg's Arch Pharmacol. 1996;353(Suppl.):R154.

269. Marinetti L, Lehman L, Casto B, Harshbarger K, Kubiczek P, Davis J. Over-the-counter cold medications – postmortem findings in infants and the relationship to cause of death. J Anal Toxicol. 2005;29:738-43.

270. Repetto MR, Repetto M. Habitual, toxic, and lethal concentrations of 103 drugs of abuse in humans. J Toxicol Clin Toxicol. 1997;35(1):1-9.

271. Kintz P, Tracqui A, Mangin P, Lugnier AA, Chaumont AJ. Fatal intoxication by dextromoramide: a report on two cases. J Anal Toxicol. 1989;13:238-9.

272. Ufkes JG, de Vos JW, van Brussel GH. Determination and pharmacokinetics of dextromoramide in methadone maintenance therapy. Pharm World Sci. 1998;20:83-7.

273. Iwersen-Bergmann S, Toennes SW, Schmidt K, Köhler W, Zokai A, Kauert GF. Bewertung von Morphinspiegeln bei fraglich Herointoten - immer noch eine Herausforderung [Abstract] [in German]. Rechtsmedizin. 2007;17:259.

274. Teske J, Weller JP, Tröger HD, Koal T, Kaever V, Breyer R, et al. Blutspiegel von Heroinfolgeprodukten bei hochdosierter Heroinapplikation [Abstract] [in German]. Rechtsmedizin. 2004;14:315.

275. Halbsguth U, Rentsch KM, Eich-Hochli D, Diterich I, Fattinger K. Oral diacetylmorphine (heroin) yields greater morphine bioavailability than oral morphine: bioavailability related to dosage and prior opioid exposure. Br J Clin Pharmacol. 2008;66:781-91.

276. Perger L, Rentsch KM, Kullak-Ublick GA, Verotta D, Fattinger K. Oral heroin in opioid-dependent patients: pharmacokinetic comparison of immediate and extended release tablets. Eur J Pharm Sci. 2009;36:421-32.

277. Girardin F, Rentsch KM, Schwab MA, Maggiorini M, Pauli-Magnus C, Kullak-Ublick GA, et al. Pharmacokinetics of high doses of intramuscular and oral heroin in narcotic addicts. Clin Pharmacol Ther. 2003;74:341-52.

278. Kidd S, Brennan S, Stephen R, Minns R, Beattie T. Comparison of morphine concentration-time profiles following intravenous and intranasal diamorphine in children. Arch Dis Child. 2009;94:974-8.

279. Rentsch KM, Kullak-Ublick GA, Reichel C, Meier PJ, Fattinger K. Arterial and venous pharmacokinetics of intravenous heroin in subjects who are addicted to narcotics. Clin Pharmacol Ther. 2001;70:237-46.

280. Rook EJ, Huitema AD, van den Brink W, van Ree JM, Beijnen JH. Population pharmacokinetics of heroin and its major metabolites. Clin Pharmacokinet. 2006;45:401-17.

281. Rook EJ, van Ree JM, van den Brink W, Hillebrand MJ, Huitema AD, Hendriks VM, et al. Pharmacokinetics and pharmacodynamics of high doses of pharmaceutically prepared heroin, by intravenous or by inhalation route in opioid-dependent patients. Basic Clin Pharmacol Toxicol. 2006;98:86-96.

282. Bever CT, Jr., Leslie J, Camenga DL, Panitch HS, Johnson KP. Preliminary trial of 3,4-diaminopyridine in patients with multiple sclerosis. Ann Neurol. 1990;27:421-7.

283. Divoll M, Greenblatt DJ, Lacasse Y, Shader RI. Benzodiazepine overdosage: plasma concentrations and clinical outcome. Psychopharmacology (Berl). 1981;73:381-3.

284. Friedman H, Greenblatt DJ, Peters GR, Metzler CM, Charlton MD, Harmatz JS, et al. Pharmacokinetics and pharmacodynamics of oral diazepam: effect of dose, plasma concentration, and time. Clin Pharmacol Ther. 1992;52:139-50.

285. Klotz U, Avant GR, Hoyumpa A, Schenker S, Wilkinson GR. The effects of age and liver disease on the disposition and elimination of diazepam in adult man. J Clin Invest. 1975;55:347-59.

286. Traeger SM, Haug MT, III. Reduction of diazepam serum half life and reversal of coma by activated charcoal in a patient with severe liver disease. J Toxicol Clin Toxicol. 1986;24:329-37.

287. Davies NM, Anderson KE. Clinical pharmacokinetics of diclofenac. Therapeutic insights and pitfalls. Clin Pharmacokinet. 1997;33:184-213.

288. Fowler PD, Dawes PT, John VA, Shotton PA. Plasma and synovial fluid concentrations of diclofenac sodium and its hydroxylated metabolites during once-daily administration of a 100 mg slow-release formulation. Eur J Clin Pharmacol. 1986;31:469-72.

289. Fowler PD, Shadforth MF, Crook PR, John VA. Plasma and synovial fluid concentrations of diclofenac sodium and its major hydroxylated metabolites during long-term treatment of rheumatoid arthritis. Eur J Clin Pharmacol. 1983;25:389-94.

290. Burger DM, Meenhorst PL, Beijnen JH. Concise overview of the clinical pharmacokinetics of dideoxynucleoside antiretroviral agents. Pharm World Sci. 1995;17:25-30.

291. Burger DM, Meenhorst PL, ten Napel CHH, Mulder JW, Henrichs JH, Frissen PHJ, et al. Limited sampling models for the antiretroviral agent didanosine. J Pharm Sci. 1995;3:7-13.

292. Bolla S, Boinpally RR, Poondru S, Devaraj R, Jasti BR. Pharmacokinetics of diethylcarbamazine after single oral dose at two different times of day in human subjects. J Clin Pharmacol. 2002;42:327-31.

293. White S, Wong SH. Standards of laboratory practice: analgesic drug monitoring. National Academy of Clinical Biochemistry. Clin Chem. 1998;44:1110-23.

294. Dollery C. Therapeutic Drugs. Edinburgh: Churchill Livingstone; 1991.

295. Gschwantler M, Gulz W, Brownstone E, Feichtenschlager T, Pulgram T, Schrutka-Kolbl C, et al. Digitoxin-induzierte Thrombozytopenie [in German]. Wien Klin Wochenschr. 1993;105:500-2.

296. Daldrup T, Pier S. Tödliche Vergiftung durch Orphenadrin/Diphenhydramin [in German]. Toxichem Krimtech. 1994;61:9.

297. Isabelle C, Warner A. Long-term heavy use of diphenhydramine without anticholinergic delirium. Am J Health Syst Pharm. 1999;56:555-7.

298. Oikkonen M, Karkela J, Seppala T. CSF concentrations and clinical effects following intravenous dixyrazine premedication. Eur J Clin Pharmacol. 1995;47:445-7.

299. Barone JA. Domperidone: a peripherally acting dopamine2-receptor antagonist. Ann Pharmacother. 1999;33:429-40.

300. Heykants J, Hendriks R, Meuldermans W, Michiels M, Scheygrond H, Reyntjens H. On the pharmacokinetics of domperidone in animals and man. IV. The pharmacokinetics of intravenous domperidone and its bioavailability in man following intramuscular, oral and rectal administration. Eur J Drug Metab Pharmacokinet. 1981;6:61-70.

301. Huang YC, Colaizzi JL, Bierman RH, Woestenborghs R, Heykants JJ. Pharmacokinetics and dose proportionality of domperidone in healthy volunteers. J Clin Pharmacol. 1986;26:628-32.

302. Michiels M, Hendriks R, Heykants J. On the pharmacokinetics of domperidone in animals and man II. Tissue distribution, placental and milk transfer of domperidone in the Wistar rat. Eur J Drug Metab Pharmacokinet. 1981;6:37-48.

303. Imbimbo BP. Pharmacodynamic-tolerability relationships of cholinesterase inhibitors for Alzheimer's disease. CNS Drugs. 2001;15:375-90.

304. Rogers SL, Friedhoff LT. The efficacy and safety of donepezil in patients with Alzheimer's disease: results of a US multicentre, randomized, double-blind, placebo-controlled trial. The Donepezil Study Group. Dementia. 1996;7:293-303.

305. Keller T, Schneider A, Tutsch-Bauer E. Fatal intoxication due to dothiepin. Forensic Sci Int. 2000;109:159-66.

306. Schulz M, Schmoldt A. Successful physostigmine treatment of acute dothiepin intoxication. Pharmazie. 1994;49(8):614.

307. Barbe F, Hansen C, Badonnel Y, Legagneur H, Vert P, Boutroy MJ. Severe side effects and drug plasma concentrations in preterm infants treated with doxapram. Ther Drug Monit. 1999;21:547-52.

308. Apple FS. Postmortem tricyclic antidepressant concentrations: assessing cause of death using parent drug to metabolite ratio. J Anal Toxicol. 1989;13:197-8.

309. Ereshefsky L, Tran-Johnson T, Davis CM, LeRoy A. Pharmacokinetic factors affecting antidepressant drug clearance and clinical effect: evaluation of doxepin and imipramine – new data and review. Clin Chem. 1988;34:863-80.

310. Kretzschmar M. Intoxikationen mit schlaffördernden Mitteln [in German]. Z Arztl Fortbild Qual sich. 2001;95:45-9.

311. Bockholdt B, Klug E, Schneider V. Suicide through doxylamine poisoning. Forensic Sci Int. 2001;119:138-40.

312. Johansson E, Agurell S, Hollister LE, Halldin MM. Prolonged apparent half-life of delta 1-tetrahydrocannabinol in plasma of chronic marijuana users. J Pharm Pharmacol. 1988;40:374-5.

313. Ohlsson A, Lindgren JE, Wahlen A, Agurell S, Hollister LE, Gillespie HK. Plasma delta-9 tetrahydrocannabinol concentrations and clinical effects after oral and intravenous administration and smoking. Clin Pharmacol Ther. 1980;28:409-16.

314. Sawyer CA, Baker AB, Ramzan I, Regaglia F. Droperidol elimination after cardiopulmonary bypass surgery. J Clin Pharmacol. 1998;38:160-5.

315. Committee for Medicinal Products for Human Use (CHMP). Xigris (drotrecogin alfa) EPAR - Product information. [European Medicines Agency]. https://www.ema.europa.eu/en/medicines/human/EPAR/xigris. Accessed 25 Feb 2020.

316. Aquilonius SM, Hartvig P. Clinical pharmacokinetics of cholinesterase inhibitors. Clin Pharmacokinet. 1986;11:236-49.

317. Iseman MD. Treatment of multidrug-resistant tuberculosis. N Engl J Med. 1993;329:784-91.

318. Brent J, McMartin K, Phillips S, Burkhart KK, Donovan JW, Wells M, et al. Fomepizole for the treatment of ethylene glycol poisoning. Methylpyrazole for Toxic Alcohols Study Group. N Engl J Med. 1999;340:832-8.

319. Fraser AD. Clinical toxicologic implications of ethylene glycol and glycolic acid poisoning. Ther Drug Monit. 2002;24:232-8.

320. Divanon F, Leroyer R, Leprince MC, Riby JP, Collet C. A propos d'une intoxication par l'éthylène glycol. J Pharm Clin. 1997;16:177-82.

321. Hantson P, Vanbinst R, Mahieu P. Determination of ethylene glycol tissue content after fatal oral poisoning and pathologic findings. Am J Forensic Med Pathol. 2002;23:159-61.

322. Hoffmann U, Abel P, Neurath H. Acute ethylene glycol poisoning after intentional ingestion. Toxichem Krimtech. 2008;75:130-3.

323. Leikin JB, Toerne T, Burda A, McAllister K, Erickson T. Summertime cluster of intentional ethylene glycol ingestions. JAMA. 1997;278:1406.

324. Porter WH, Rutter PW, Bush BA, Pappas AA, Dunnington JE. Ethylene glycol toxicity: the role of serum glycolic acid in hemodialysis. J Toxicol Clin Toxicol. 2001;39:607-15.

325. Reddy NJ, Lewis LD, Gardner TB, Osterling W, Eskey CJ, Nierenberg DW. Two cases of rapid onset Parkinson's syndrome following toxic ingestion of ethylene glycol and methanol. Clin Pharmacol Ther. 2007;81:114-21.

326. Sivilotti ML, Burns MJ, McMartin KE, Brent J. Toxicokinetics of ethylene glycol during fomepizole therapy: implications for management. For the Methylpyrazole for Toxic Alcohols Study Group. Ann Emerg Med. 2000;36:114-25.

327. Wildsmith JA, Tucker GT, Cooper S, Scott DB, Covino BG. Plasma concentrations of local anaesthetics after interscalene brachial plexus block. Br J Anaesth. 1977;49:461-6.

328. Boni J, Korth-Bradley J, McGoldrick K, Appel A, Cooper S. Pharmacokinetic and pharmacodynamic action of etodolac in patients after oral surgery. J Clin Pharmacol. 1999;39:729-37.

329. Kirchner GI, Meier-Wiedenbach I, Manns MP. Clinical pharmacokinetics of everolimus. Clin Pharmacokinet. 2004;43:83-95.

330. Kovarik JM, Kaplan B, Tedesco SH, Kahan BD, Dantal J, Vitko S, et al. Exposure-response relationships for everolimus in de novo kidney transplantation: defining a therapeutic range. Transplantation. 2002;73:920-5.

331. Kovarik JM, Eisen H, Dorent R, Mancini D, Vigano M, Rouilly M, et al. Everolimus in de novo cardiac transplantation: pharmacokinetics, therapeutic range, and influence on cyclosporine exposure. J Heart Lung Transplant. 2003; 22:1117-25.

332. Starling RC, Hare JM, Hauptman P, McCurry KR, Mayer HW, Kovarik JM, et al. Therapeutic drug monitoring for everolimus in heart transplant recipients based on exposure-effect modeling. Am J Transplant. 2004;4:2126-31.

333. Ezzet F, Krishna G, Wexler DB, Statkevich P, Kosoglou T, Batra VK. A population pharmacokinetic model that describes multiple peaks due to enterohepatic recirculation of ezetimibe. Clin Ther. 2001;23:871-85.

334. Ezzet F, Wexler D, Statkevich P, Kosoglou T, Patrick J, Lipka L, et al. The plasma concentration and LDL-C relationship in patients receiving ezetimibe. J Clin Pharmacol. 2001;41:943-9.

335. AHFS. AHFS Drug Information. Bethesda: American Society of Health-System Pharmacists; 2002.

336. Yoshimoto K, Saima S, Echizen H, Nakamura Y, Kondo T, Yagishita Y, et al. Famotidine-associated central nervous system reactions and plasma and cerebrospinal drug concentrations in neurosurgical patients with renal failure. Clin Pharmacol Ther. 1994;55:693-700.

337. Graves NM. Felbamate. Ann Pharmacother. 1993;27(9):1073-81.

338. Troupin AS, Montouris G, Hussein G. Felbamate: Therapeutic range and other kinetic information. Epilepsy. 1997;10:26-31.

339. Wagner ML. Felbamate: a new antiepileptic drug. Am J Hosp Pharm. 1994;51:1657-66.

340. Bolten W, Salzmann G, Goldmann R, Miehlke K. Plasma- und Gewebekonzentrationen von Biphenylessigsäure nach einwöchiger oraler Fenbufenmedikation bzw. topischer Anwendung von Felbinac-Gel am Kniegelenk [in German]. Z Rheumatol. 1989;48:317-22.

341. Blychert E, Edgar B, Elmfeldt D, Hedner T. Plasma concentration–effect relationships for felodipine: a meta analysis. Clin Pharmacol Ther. 1992;52:80-9.

342. Lössner A, Banditt P, Troger U. Rapid and simple method for detection of fenofibric acid in human serum by high-performance liquid chromatography. Pharmazie. 2001;56:50-1.

343. Hercegova A, Polonsky J. Determination of non-steroidal anti-inflammatory drugs in biological fluids. Pharmazie. 1999;54:479-86.

344. Hug CC. Fentanyl and sufentanil anesthesia revisited: establish an effective plasma concentration and achieve it at the right time [Reply]. Anesthesiology. 1991;74(2):390.

345. Lehmann KA, Freier J, Daub D. Fentanyl-Pharmakokinetik und postoperative Atemdepression [in German]. Anaesthesist. 1982;31:111-8.

346. Philbin DM, Rosow CE, Schneider RC, Koski G, D'Ambra MN. Fentanyl and sufentanil anesthesia revisited: establish an effective plasma concentration and achieve it at the right time [Reply]. Anesthesiology. 1991;74(2):389-90.

347. Singleton MA, Rosen JI, Fisher DM. Plasma concentrations of fentanyl in infants, children and adults. Can J Anaesth. 1987;34:152-5.

348. Smialek JE, Levine B, Chin L, Wu SC, Jenkins AJ. A fentanyl epidemic in Maryland 1992. J Forensic Sci. 1994;39:159-64.

349. Stanley TH, Bailey PL. Fentanyl and sufentanil anesthesia revisited: establish an effective plasma concentration and achieve it at the right time [Letter]. Anesthesiology. 1991;74(2):388-89.

350. Yerasi AB, Butts JD, Butts JD. Disposal of used fentanyl patches. Am J Health Syst Pharm.1997;54:85-6.

351. Andresen H, Gullans A, Veselinovic M, Anders S, Schmoldt A, Iwersen-Bergmann S, et al. Fentanyl: toxic or therapeutic? Postmortem and antemortem blood concentrations after transdermal fentanyl application. J Anal Toxicol. 2012;36(3):182-94.

352. Simons FE, Bergman JN, Watson WT, Simons KJ. The clinical pharmacology of fexofenadine in children. J Allergy Clin Immunol. 1996;98:1062-4.

353. Steiner JF. Clinical pharmacokinetics and pharmacodynamics of finasteride. Clin Pharmacokinet. 1996;30(1):16-27.

354. Evers J, Eichelbaum M, Kroemer HK. Unpredictability of flecainide plasma concentrations in patients with renal failure: relationship to side effects and sudden death? Ther Drug Monit. 1994;16(4):349-51.

355. Debruyne D, Ryckelynck JP. Clinical pharmacokinetics of fluconazole. Clin Pharmacokinet. 1993;24(1):10-27.

356. Debruyne D. Clinical pharmacokinetics of fluconazole in superficial and systemic mycoses. Clin Pharmacokinet. 1997;33(1):52-77.

357. Reuman PD, Neiberger R, Kondor DA. Intraperitoneal and intravenous fluconazole pharmokinetics in a pediatric patient with end stage renal disease. Pediatr Infect Dis J. 1992;11:132-3.

358. Scholz J, Schulz M, Steinfath M, Hover S, Bause H. Fluconazole is removed by continuous venovenous hemofiltration in a liver transplant patient. J Mol Med. 1995;73(3):145-7.

359. Bond A, Seijas D, Dawling S, Lader M. Systemic absorption and abuse liability of snorted flunitrazepam. Addiction. 1994;89(7):821-30.

360. Pak CY, Sakhaee K, Rubin CD, Zerwekh JE. Sustained-release sodium fluoride in the management of established postmenopausal osteoporosis. Am J Med Sci. 1997;313:23-32.

361. Pitt P, Berry H. Fluoride treatment in osteoporosis. Postgrad Med J. 1991;67:323-6.

362. von Werder K, Schulz M. Prophylaxe und Therapie der Osteoporose [in German]. Stuttgart: WVG; 1991.

363. Henry JA. Toxicity of antidepressants: comparisons with fluoxetine. Int Clin Psychopharmacol. 1992;6(Suppl. 6):22-7.

364. Renshaw PF, Guimaraes AR, Fava M, Rosenbaum JF, Pearlman JD, Flood JG, et al. Accumulation of fluoxetine and norfluoxetine in human brain during therapeutic administration. Am J Psychiatry. 1992;149:1592-4.

365. Niebch G, Borbe HO, Hummel T, Kobal G. Dose-proportional plasma levels of the analgesic flupirtine maleate in man. Application of a new HPLC assay. Arzneimittelforschung. 1992;42:1343-5.

366. Forland SC, Wechter WJ, Witchwoot S, Clifford KH, Arnett RL, Cutler RE. Human plasma concentrations of R, S, and racemic flurbiprofen given as a toothpaste. J Clin Pharmacol. 1996;36:546-53.

367. Schulz M, Schmoldt A, Donn F, Becker H. The pharmacokinetics of flutamide and its major metabolites after a single oral dose and during chronic treatment. Eur J Clin Pharmacol. 1988;34(6):633-6.

368. Grimsley SR, Jann MW. Paroxetine, sertraline, and fluvoxamine: new selective serotonin reuptake inhibitors. Clin Pharm. 1992;11:930-57.

369. Wood DM, Rajalingam Y, Greene SL, Morgan PE, Gerrie D, Jones AL, et al. Status epilepticus following intentional overdose of fluvoxamine: a case report with serum fluvoxamine concentration. Clin Toxicol (Phila). 2007;45:791-3.

370. Andrews CO, Fischer JH. Gabapentin: a new agent for the management of epilepsy. Ann Pharmacother. 1994;28:1188-96.

371. Blum RA, Comstock TJ, Sica DA, Schultz RW, Keller E, Reetze P, et al. Pharmacokinetics of gabapentin in subjects with various degrees of renal function. Clin Pharmacol Ther. 1994;56:154-9.

372. Bockbrader HN. Clinical pharmacokinetics of gabapentin. Drugs Today. 1995;31:613-9.

373. Bockbrader HN, Wesche D, Miller R, Chapel S, Janiczek N, Burger P. A comparison of the pharmacokinetics and pharmacodynamics of pregabalin and gabapentin. Clin Pharmacokinet. 2010;49:661-9.

374. Btaiche IF, Woster PS. Gabapentin and lamotrigine: novel antiepileptic drugs. Am J Health Syst Pharm. 1995;52:61-9.

375. Knörle R, Feuerstein TJ, Schulze-Bonhage A. Determination of gabapentin-lactam in serum of patients under gabapentin therapy. Arzneimittelforschung. 2004;54:139-42.

376. Peruche B, Schulz M. Gabapentin, ein neues Antiepileptikum [in German]. Pharm Ztg. 1996;141(16):1396-404.

377. Radulovic LL, Taylor CP, Walker RM. The preclinical pharmacology, pharmacokinetics and toxicology of gabapentin. Drugs Today. 1995;31:597-611.

378. Sivenius J, Kalviainen R, Ylinen A, Riekkinen P. Double-blind study of Gabapentin in the treatment of partial seizures. Epilepsia. 1991;32:539-42.

379. Spiller HA, Dunaway MD, Cutino L. Massive gabapentin and presumptive quetiapine overdose. Vet Hum Toxicol. 2002;44:243-4.

380. Stewart BH, Kugler AR, Thompson PR, Bockbrader HN. A saturable transport mechanism in the intestinal absorption of gabapentin is the underlying cause of the lack of proportionality between increasing dose and drug levels in plasma. Pharm Res. 1993;10:276-81.

381. Tomson T, Johannessen SI. Therapeutic monitoring of the new antiepileptic drugs. Eur J Clin Pharmacol. 2000;55:697-705.

382. Wilson EA, Sills GJ, Forrest G, Brodie MJ. High dose gabapentin in refractory partial epilepsy: clinical observations in 50 patients. Epilepsy Res. 1998; 29:161-6.

383. Bickel U, Thomsen T, Weber W, Fischer JP, Bachus R, Nitz M, et al. Pharmacokinetics of galanthamine in humans and corresponding cholinesterase inhibition. Clin Pharmacol Ther. 1991;50:420-8.

384. Scott LJ, Goa KL. Galantamine: a review of its use in Alzheimer's disease. Drugs. 2000;60:1095-122.

385. Frese JH, Rohland L, Schulz M, Schmoldt A. Intoxikation mit Gallopamil. Verlauf und Therapie [Gallopamil poisoning. Its course and therapy] [in German]. Dtsch Med Wochenschr. 1988;113(19):770-2.

386. Andresen H, Sprys N, Schmoldt A, Mueller A, Iwersen-Bergmann S. Gamma-hydroxybutyrate in urine and serum: additional data supporting current cut-off recommendations. Forensic Sci Int. 2010;200:93-9.

387. Andresen H, Aydin BE, Mueller A, Iwersen-Bergmann S. An overview of gamma-hydroxybutyric acid: pharmacodynamics, pharmacokinetics, toxic effects, addiction, analytical methods, and interpretation of results. Drug Test Anal. 2011;3:560-8.

388. Jung D, Griffy K, Wong R, Colburn W, Hulse J. Steady-state relative bioavailability of three oral ganciclovir dosage regimens delivering 6,000 mg/day in patients with human immunodeficiency virus. J Clin Pharmacol. 1998;38:1021-4.

389. Brier ME, Zurada JM, Aronoff GR. Neural network predicted peak and trough gentamicin concentrations. Pharm Res. 1995;12:406-12.

390. Rybak M, Lomaestro B, Rotschafer JC, Moellering R Jr, Craig W, Billeter M, et al. Therapeutic monitoring of vancomycin in adult patients: a consensus review of the American Society of Health-System Pharmacists, the Infectious Diseases Society of America, and the Society of Infectious Diseases Pharmacists. Am J Health Syst Pharm. 2009;66(1):82-98.

391. Inciardi JF, Willits NH. Setting confidence intervals for drug concentrations from pharmacokinetic parameters. Ann Pharmacother. 1992;26:1070-4.

392. Kozyrskyj A, Masih M, Hahn J, Ho C, Wong M, Sirdevan M. New neonatal gentamicin dosing guidelines: results of an evaluation of serum concentrations. Can J Hosp Pharm. 1994;47:262-7.

393. Modi N, Maggs AF, Clarke C, Chapman C, Swann RA. Gentamicin concentration and toxicity. Lancet. 1998;352:70.

394. Watling SM, Kisor DF. Population pharmacokinetics: development of a medical intensive care unit-specific gentamicin dosing nomogram. Ann Pharmacother. 1993;27:151-4.

395. Lam YW, Jann MW, Chang WH, Yu HS, Lin SK, Chen H, et al. Intra- and interethnic variability in reduced haloperidol to haloperidol ratios. J Clin Pharmacol. 1995;35:128-36.

396. Potkin SG, Shen Y, Pardes H, Phelps BH, Zhou D, Shu L, et al. Haloperidol concentrations elevated in Chinese patients. Psychiatry Res. 1984;12:167-72.

397. Ulrich S, Wurthmann C, Brosz M, Meyer FP. The relationship between serum concentration and therapeutic effect of haloperidol in patients with acute schizophrenia. Clin Pharmacokinet. 1998;34:227-63.

398. Ulrich S, Meyer FP. Reduzierte Haloperidol und Haloperidolpyridinium-Metaboliten bei schizophrenen Patienten [in German]. Psychopharmakotherapie. 1999;6:100-2.

399. Challapalli R, Lefkovits J, Topol EJ. Clinical trials of recombinant hirudin in acute coronary syndromes. Coron Artery Dis. 1996;7:429-37.

400. Hagen N, Thirlwell MP, Dhaliwal HS, Babul N, Harsanyi Z, Darke AC. Steady-state pharmacokinetics of hydromorphone and hydromorphone-3-glucuronide in cancer patients after immediate and controlled-release hydromorphone. J Clin Pharmacol. 1995;35:37-44.

401. Mason PE, Kerns WP. Gamma hydroxybutyric acid (GHB) intoxication. Acad Emerg Med. 2002;9:730-9.

402. Steinecke H. Beitrag zur Bewertung von Gamma-Hydroxybuttersäure (GBH) - Konzentrationen im Blut lebender Personen sowie in postmortalem Blut [in German]. Toxichem Krimtech. 2007;74:150-4.

403. Kunze K, Kauert U, Schmoldt A. Drug induced myopathy by hydroxychloroquine. Vet Hum Toxicol. 1987;29:59-60.

404. Abraham TT, Barnes AJ, Lowe RH, Kolbrich Spargo EA, Milman G, Pirnay SO, et al. Urinary MDMA, MDA, HMMA, and HMA excretion following controlled MDMA administration to humans. J Anal Toxicol. 2009;33:439-46.

405. Barnes AJ, Scheidweiler KB, Kolbrich-Spargo EA, Gorelick DA, Goodwin RS, Huestis MA. MDMA and metabolite disposition in expectorated oral fluid after controlled oral MDMA administration. Ther Drug Monit. 2011;33:602-8.

406. Kolbrich EA, Goodwin RS, Gorelick DA, Hayes RJ, Stein EA, Huestis MA. Plasma pharmacokinetics of 3,4-methylenedioxymethamphetamine after controlled oral administration to young adults. Ther Drug Monit. 2008;30:320-32.

407. Forsyth DR, Jayasinghe KS, Roberts CJ. Do nizatidine and cimetidine interact with ibuprofen? Eur J Clin Pharmacol. 1988;35:85-8.

408. Holubek WJ, Wetter A, Howland MA, Hoffman RS, Nelson LS. Death from a massive ibuprofen overdose [Abstract]. Clin Toxicol. 2006;44:488.

409. Pisano P, Durand A, Autret E, Desnuelle C, Pinsard N, Serratrice G, et al. Plasma concentrations and pharmacokinetics of idebenone and its metabolites following single and repeated doses in young patients with mitochondrial encephalomyopathy. Eur J Clin Pharmacol. 1996;51:167-9.

410. Druker BJ, Talpaz M, Resta DJ, Peng B, Buchdunger E, Ford JM, et al. Efficacy and safety of a specific inhibitor of the BCR-ABL tyrosine kinase in chronic myeloid leukemia. N Engl J Med. 2001;344:1031-7.

411. Lyseng-Williamson K, Jarvis B. Imatinib. Drugs. 2001;61:1765-74.

412. Tegeder I, Bremer F, Oelkers R, Schüttler J, Brune K, Geisslinger G. Therapeutic drug monitoring of imipenem during continuous veno-venous hemofiltration [Abstract]. Naunyn-Schmiedeberg's Arch Pharmacol. 1996;355(Suppl.):R129.

413. Jorgensen OS, Lober M, Christiansen J, Gram LF. Plasma concentration and clinical effect in imipramine treatment of childhood enuresis. Clin Pharmacokinet. 1980;5:386-93.

414. Rayner CR, Galbraith KJ, Marriott JL, Duncan GJ. A critical evaluation of the therapeutic range of indinavir. Ann Pharmacother. 2002;36:1230-7.

415. Gilman JT, Gal P. Pharmacokinetic and pharmacodynamic data collection in children and neonates. A quiet frontier. Clin Pharmacokinet. 1992;23:1-9.

416. Mistry GC, Jensen BK, Rakhit A, Huselton CA, Patel IH. Systemic availability of retinoids following excessive topical application of isotrex to patients with acne vulgaris [Abstract]. Pharm Res. 1995;12:S-414.

417. Nulman I, Berkovitch M, Klein J, Pastuszak A, Lester RS, Shear N, et al. Steady-state pharmacokinetics of isotretinoin and its 4-oxo metabolite: implications for fetal safety. J Clin Pharmacol. 1998;38:926-30.

418. Chellingsworth MD, Willis JV, Jack DB, Kendall MJ. Pharmacokinetics and pharmacodynamics of isradipine (PN200-110) in young and elderly patients. Am J Med. 1988;72:72-9.

419. Romano MJ, Gaylor A, Sang CJ, Jr. Life-threatening isradipine poisoning in a child. Pharmacotherapy. 2002;22:766-70.

420. Tse FL, Jaffe JM. Pharmacokinetics of PN 200-110 (isradipine), a new calcium antagonist, after oral administration in man. Eur J Clin Pharmacol. 1987;32:361-5.

421. Barone JA, Moskovitz BL, Guarnieri J, Hassell AE, Colaizzi JL, Bierman RH, et al. Food interaction and steady-state pharmacokinetics of itraconazole oral solution in healthy volunteers. Pharmacotherapy. 1998;18:295-301.

422. Lipp H-P. Klinische Pharmakokinetik von Itraconazol [in German]. Krankenhauspharmazie. 1996;17:388-95.

423. Neuvonen PJ, Varhe A, Olkkola KT. The effect of ingestion time interval on the interaction between itraconazole and triazolam. Clin Pharmacol Ther. 1996;60:326-31.

424. Slain D, Rogers PD, Cleary JD, Chapman SW. Intravenous itraconazole. Ann Pharmacother. 2001;35:720-9.

425. Okonkwo PO, Ogbuokiri JE, Ofoegbu E, Klotz U. Protein binding and ivermectin estimations in patients with onchocerciasis. Clin Pharmacol Ther. 1993;53:426-30.

426. Bondesson U, Hartvig P, Danielsson B. Quantitative determination of the urinary excretion of ketobemidone and four of its metabolites after intravenous and oral administration in man. Drug Metab Dispos. 1981;9:376-80.

427. Hartvig P, Valtysson J, Lindner KJ, Kristensen J, Karlsten R, Gustafsson LL, et al. Central nervous system effects of subdissociative doses of (S)-ketamine are related to plasma and brain concentrations measured with positron emission tomography in healthy volunteers. Clin Pharmacol Ther. 1995;58:165-73.

428. Steentoft A, Worm K. Cases of fatal intoxication with Ketogan. J Forensic Sci Soc. 1994;34:181-5.

429. Ballerini R, Casini A, Chinol M, Mannucci C, Giaccai L, Salvi M. Study on the absorption of ketoprofen topically administered in man: comparison between tissue and plasma levels. Int J Clin Pharmacol Res. 1986;6:69-72.

430. Dionne RA, Gordon SM, Tahara M, Rowan J, Troullos E. Analgesic efficacy and pharmacokinetics of ketoprofen administered into a surgical site. J Clin Pharmacol. 1999;39:131-8.

431. Ishizaki T, Sasaki T, Suganuma T, Horai Y, Chiba K, Watanabe M, et al. Pharmacokinetics of ketoprofen following single oral, intramuscular and rectal doses and after repeated oral administration. Eur J Clin Pharmacol. 1980;18:407-14.

432. Lewellen ORW, Templeton RT. The pharmacokinetics of ketoprofen in man during and after repeated oral dosing (50 mg q.i.d.) with Orudis(R) [Abstract]. Scand J Rheumatol. 1976;14:53-62.

433. Netter P, Bannwarth B, Lapicque F, Harrewyn JM, Frydman A, Tamisier JN, et al. Total and free ketoprofen in serum and synovial fluid after intramuscular injection. Clin Pharmacol Ther. 1987;42:555-61.

434. Johnson MA, Moore KH, Yuen GJ, Bye A, Pakes GE. Clinical pharmacokinetics of lamivudine. Clin Pharmacokinet. 1999;36:41-66.

435. Rambeck B, Wolf P. Lamotrigine clinical pharmacokinetics. Clin Pharmacokinet. 1993;25:433-43.

436. Schapel GJ, Beran RG, Vajda FJ, Berkovic SF, Mashford ML, Dunagan FM, et al. Double-blind, placebo controlled, crossover study of lamotrigine in treatment resistant partial seizures. J Neurol Neurosurg Psychiatry. 1993;56:448-53.

437. French LK, McKeown NJ, Hendrickson RG. Complete heart block and death following lamotrigine overdose. Clin Toxicol (Phila). 2011;49:330-3.

438. Schaller K-H, Angerer H, Lehnert G. Bio-Monitoring in der Arbeits- und Umweltmedizin [in German]. Dt Ärztebl. 1993;90:C-1430-5.

439. Schmid I, Paulweber B, Pechböck W, Oberkofler H, Patsch W. Eine spät erkannte Bleiintoxikation [in German]. Toxichem Krimtech. 2000;67:96-7.

440. Beaman JM, Hackett LP, Luxton G, Illett KF. Effect of hemodialysis on leflunomide plasma concentrations. Ann Pharmacother. 2002;36:75-7.

441. Goldenberg MM. Leflunomide, a novel immunomodulator for the treatment of active rheumatoid arthritis. Clin Ther. 1999;21:1837-52.

442. Lucien J, Dias VC, LeGatt DF, Yatscoff RW. Blood distribution and single-dose pharmacokinetics of leflunomide. Ther Drug Monit. 1995;17:454-9.

443. Barrueto F, Jr., Williams K, Howland MA, Hoffman RS, Nelson LS. A case of levetiracetam (Keppra) poisoning with clinical and toxicokinetic data. J Toxicol Clin Toxicol. 2002;40:881-4.

444. Heykants J, Van PA, Van dV, V, Snoeck E, Meuldermans W, Woestenborghs R. The pharmacokinetic properties of topical levocabastine. A review. Clin Pharmacokinet. 1995;29:221-30.

445. Hoehn MM, Rutledge CO. Acute overdose with levodopa. Clinical and biochemical consequences. Neurology. 1975;25(8):792-4.

446. Harder S, Baas H, Rietbrock S. Concentration-effect relationship of levodopa in patients with Parkinson's disease. Clin Pharmacokinet. 1995;29:243-56.

447. Harder S, Baas H, Bergemann N, Demisch L, Rietbrock S. Concentration-effect relationship of levodopa in patients with Parkinson's disease after oral

administration of an immediate release and a controlled release formulation. Br J Clin Pharmacol. 1995;39:39-44.

448. Harder S, Baas H. Concentration-response relationship of levodopa in patients at different stages of Parkinson's disease. Clin Pharmacol Ther. 1998;64:183-91.

449. Sturner WQ, Garriott JC. L-dopa poisoning. J Forensic Sci. 1972;17:440-3.

450. Schall U, Katta T, Pries E, Klöppel A, Gastpar M. Dosierung von Levomethadon in der Substitutionsbehandlung i.v.-Opiatabhängiger [in German]. Dt Ärztebl. 1994;91:C-556-7.

451. Foy JL, Eastman RC, Nealon RC, Bowen PM, Pengelly ML, Drass JA, et al. Automated therapeutic drug monitoring in an ambulatory care endocrine clinic. Ann Pharmacother. 1992;26:675-8.

452. Dawling S, Flanagan RJ, Widdop B. Fatal lignocaine poisoning: report of two cases and review of the literature. Hum Toxicol. 1989;8:389-92.

453. den Hartigh J, Hilders CG, Schoemaker RC, Hulshof JH, Cohen AF, Vermeij P. Tinnitus suppression by intravenous lidocaine in relation to its plasma concentration. Clin Pharmacol Ther. 1993;54:415-20.

454. Wu FL, Razzaghi A, Souney PF. Seizure after lidocaine for bronchoscopy: case report and review of the use of lidocaine in airway anesthesia. Pharmacotherapy. 1993;13:72-8.

455. Alderman CP, Lindsay KS. Increased serum lithium concentration secondary to treatment with tiaprofenic acid and fosinopril. Ann Pharmacother. 1996;30:1411-3.

456. Goff DC, Baldessarini RJ. Drug interactions with antipsychotic agents. J Clin Psychopharmacol. 1993;13:57-67.

457. Grobosch T, Schönberg L, Lampe D. Toxikologisches Monitoring von Risperidon und Lithium bei einer akuten Intoxikation [in German]. Toxichem Krimtech. 2004;71:10-6.

458. Siwers B, Borg S, d'Elia G, Lundin G, Forshell GP, Raotma H, Román G. Comparative clinical evaluation of lofepramine and imipramine. Pharmacological aspects. Acta Psychiatr Scand. 1977;55(1):21-31.

459. Killinger JM, Weintraub HS, Fuller BL. Human pharmacokinetics and comparative bioavailability of loperamide hydrochloride. J Clin Pharmacol. 1979;19:211-8.

460. Radwanski E, Hilbert J, Symchowicz S, Zampaglione N. Loratadine: multiple-dose pharmacokinetics. J Clin Pharmacol. 1987;27:530-3.

461. Zhang YF, Chen XY, Zhong DF, Dong YM. Pharmacokinetics of loratadine and its active metabolite descarboethoxyloratadine in healthy Chinese subjects. Acta Pharmacol Sin. 2003;24(7):715-8.

462. Henry DW, Burwinkle JW, Klutman NE. Determination of sedative and amnestic doses of lorazepam in children. Clin Pharm. 1991;10:625-9.

463. Reiter PD, Stiles AD. Lorazepam toxicity in a premature infant. Ann Pharmacother. 1993;27:727-9.

464. Gillis AM, Kates RE. Clinical pharmacokinetics of the newer antiarrhythmic agents. Clin Pharmacokinet. 1984;9:375-403.

465. Mead RH, Keefe DL, Kates RE, Winkle RA. Chronic lorcainide therapy for symptomatic premature ventricular complexes: efficacy, pharmacokinetics and evidence for norlorcainide antiarrhythmic effect. Am J Cardiol. 1985;55:72-8.

466. Somani P, Simon V, Gupta RK, King P, Shapiro RS, Stockard H. Lorcainide kinetics and protein binding in patients with end-stage renal disease. Int J Clin Pharmacol Ther Toxicol. 1984;22:121-5.

467. Ludewig R, Regenthal R. Akute Vergiftungen und Arzneimittelüberdosierungen [in German]. 11 ed., Stuttgart: WVG; 2015.

468. Wilimzig C, Latz R, Vierling W, Mutschler E, Trnovec T, Nyulassy S. Increase in magnesium plasma level after orally administered trimagnesium dicitrate. Eur J Clin Pharmacol. 1996;49:317-23.

469. Burgmann H, Winkler S, Uhl F, Feucht M, Hellgren U, Bergqvist Y, et al. Mefloquin und Sulfadoxin/Pyrimethamin-Überdosierung bei Malaria tropica [in German]. Wien Klin Wochenschr. 1993;105:61-3.

470. Hellgren U, Jastrebova J, Jerling M, Krysen B, Bergqvist Y. Comparison between concentrations of racemic mefloquine, its separate enantiomers and the carboxylic acid metabolite in whole blood serum and plasma. Eur J Clin Pharmacol. 1996;51:171-3.

471. Noble S, Balfour JA. Meloxicam. Drugs. 1996;51:424-30.

472. Hui WK, Mitchell LB, Kavanagh KM, Gillis AM, Wyse DG, Manyari DE, et al. Melperone: electrophysiologic and antiarrhythmic activity in humans. J Cardiovasc Pharmacol. 1990;15:144-9.

473. Stein S, Schmoldt A, Schulz M. Fatal intoxication with melperone. Forensic Sci Int. 2000;113:409-13.

474. NN. Quecksilber-Referenzwerte [in German]. Bundesgesundhbl. 1998;41:270.

475. Singer AJ, Mofenson HC, Caraccio TR, Ilasi J. Mercuric chloride poisoning due to ingestion of a stool fixative. Clin Toxicol. 1994;32:577-82.

476. Bernhoft RA. Mercury toxicity and treatment: a review of the literature. J Environ Public Health. 2012;2012:460508.

477. Akyildiz BN, Kondolot M, Kurtoglu S, Konuskan B. Case series of mercury toxicity among children in a hot, closed environment. Pediatr Emerg Care. 2012;28:254-8.

478. Dargan PI, Giles LJ, Wallace CI, House IM, Thomson AH, Beale RJ, et al. Case report: severe mercuric sulphate poisoning treated with 2,3-dimercaptopropane-1-sulphonate and haemodiafiltration. Crit Care. 2003;7:R1-6.

479. Klotz U, Stracciari GL. Steady state disposition of 5-aminosalicyclic acid following oral dosing. Arzneim-Forsch/Drug Res. 1993;43:1357-9.

480. Desel H, Stedtler U, Behrens A, Neuratz H. Mischintoxikation mit Metformin [in German]. Toxichem Krimtech. 2000;67:4-8.

481. Reeker W, Schneider G, Felgenhauer N, Tempel G, Kochs E. Metformin-induzierte Laktazidose [in German]. Dtsch Med Wochenschr. 2000;125:249-51.

482. Chugh SS, Socoteanu C, Reinier K, Waltz J, Jui J, Gunson K. A community-based evaluation of sudden death associated with therapeutic levels of methadone. Am J Med. 2008;121:66-71.

483. Horns WH, Rado M, Goldstein A. Plasma levels and symptom complaints in patients maintained on daily dosage of methadone hydrochloride. Clin Pharmacol Ther. 1975;17:636-49.

484. Inturrisi CE, Colburn WA, Kaiko RF, Houde RW, Foley KM. Pharmacokinetics and pharmacodynamics of methadone in patients with chronic pain. Clin Pharmacol Ther. 1987;41:392-401.

485. Inturrisi CE, Verebely K. The levels of methadone in the plasma in methadone maintenance. Clin Pharmacol Ther. 1972;13:633-7.

486. Schmidt N, Sittl R, Brune K, Geisslinger G. Rapid determination of methadone in plasma, cerebrospinal fluid, and urine by gas chromatography and its application to routine drug monitoring. Pharm Res. 1993;10:441-4.

487. Ufkes JG, de Vos JW, Geerlings PJ, van Wilgenburg H. Determination of methadone and its primary metabolite in twenty opiate addicts [Abstract]. Pharm World Sci. 1994;16:D6.

488. Heinemann A, Iwersen-Bergmann S, Stein S, Schmoldt A, Püschel K. Methadone-related fatalities in Hamburg 1990-1999: implications for quality standards in maintenance treatment? Forensic Sci Int. 2000;113:449-55.

489. Karch SB. Is it time to reformulate racemic methadone? J Addict Med. 2011;5:229-31.

490. Epker JL, Bakker J. Accidental methanol ingestion: case report. BMC Emerg Med. 2010;10:3.

491. Stefan H. Epilepsietherapie. Teil 1: Konservative Behandlung [in German]. Dt Ärztebl. 1998;95:C-2204-10.

492. Schmoldt A, Iwersen S, Schluter W. Massive ingestion of the herbicide 2-methyl-4-chlorophenoxyacetic acid (MCPA). J Toxicol Clin Toxicol. 1997;35:405-8.

493. Kraemer T, Maurer HH. Toxicokinetics of amphetamines: metabolism and toxicokinetic data of designer drugs, amphetamine, methamphetamine, and their N-alkyl derivatives. Ther Drug Monit. 2002;24:277-89.

494. Moore KA, Mozayani A, Fierro MF, Poklis A. Distribution of 3,4-methylenedioxymethamphetamine (MDMA) and 3,4-methylenedioxyamphetamine (MDA) stereoisomers in a fatal poisoning. Forensic Sci Int. 1996;83:111-19.

495. Buechler J, Schwab M, Mikus G, Fischer B, Hermle L, Marx C, et al. Enantioselective quantitation of the ecstasy compound (R)- and (S)-N-ethyl-3,4-methylenedioxyamphetamine and its major metabolites in human plasma and urine. J Chromatogr B Analyt Technol Biomed Life Sci. 2003;793:207-22.

496. Freudenmann RW, Spitzer M. The Neuropsychopharmacology and Toxicology of 3,4-methylenedioxy-N-ethyl-amphetamine (MDEA). CNS Drug Rev. 2004;10:89-116.

497. Meyer MR, Peters FT, Maurer HH. The role of human hepatic cytochrome P450 isozymes in the metabolism of racemic 3,4-methylene-dioxyethylamphetamine and its single enantiomers. Drug Metab Dispos. 2009;37:1152-6.

498. Cami J, de la Torre R, Ortuno J, Farre M, Mas M, Roset PN, et al. Pharmacokinetics of ecstasy (MDMA) in healthy subjects [Abstract]. Eur J Clin Pharmacol. 1997;52:A168.

499. Fallon JK, Kicman AT, Henry JA, Milligan PJ, Cowan DA, Hutt AJ. Stereospecific analysis and enantiomeric disposition of 3, 4-methylenedioxymethamphetamine (Ecstasy) in humans. Clin Chem. 1999;45:1058-69.

500. de Boer D., Egberts T, Maes RA. Para-methylthioamphetamine, a new amphetamine designer drug of abuse. Pharm World Sci. 1999;21:47-8.

501. Elliott SP. Fatal poisoning with a new phenylethylamine: 4-methylthioamphetamine (4-MTA). J Anal Toxicol. 2000;24:85-9.

502. Poortman AJ, Lock E. Analytical profile of 4-methylthioamphetamine (4-MTA), a new street drug. Forensic Sci Int. 1999;100:221-33.

503. Tarbah FA, Zweipfennig P, Pier S, Temme O, Daldrup T. Tödliche Vergiftung mit dem Amphetaminderivat 4-MTA [in German]. Toxichem Krimtech. 2001;68:21.

504. Weise M. Das traurige Ende einer "Geburtstagsfeier" - Fatale Intoxikation mit 4-Methylthioamphetamin [in German]. Toxichem Krimtech. 2001;68:38-42.

505. Meibohm B, Wegener S. Mexiletin-Theophyllin-Interaktion. Pharmakokinetische Auswirkungen und klinische Relevanz [in German]. Krankenhauspharmazie. 1992;13:331-3.

506. Backman JT, Olkkola KT, Neuvonen PJ. Rifampin drastically reduces plasma concentrations and effects of oral midazolam. Clin Pharmacol Ther. 1996;59:7-13.

507. Blumer JL. Clinical pharmacology of midazolam in infants and children. Clin Pharmacokinet. 1998;35:37-47.

508. Hughes J, Gill AM, Mulhearn H, Powell E, Choonara I. Steady-state plasma concentrations of midazolam in critically ill infants and children. Ann Pharmacother. 1996;30:27-30.

509. Heikinheimo O. Clinical pharmacokinetics of mifepristone. Clin Pharmacokinet. 1997;33:7-17.

510. Stimmel GL, Dopheide JA, Stahl SM. Mirtazapine: an antidepressant with noradrenergic and specific serotonergic effects. Pharmacotherapy. 1997;17:10-21.

511. Tang OS, Schweer H, Seyberth HW, Lee SW, Ho PC. Pharmacokinetics of different routes of administration of misoprostol. Hum Reprod. 2002;17:332-6.

512. Tang OS, Schweer H, Lee SW, Ho PC. Pharmacokinetics of repeated doses of misoprostol. Hum Reprod. 2009;24:1862-9.

513. Committee for Medicinal Products for Human Use (CHMP). Lysodren (mitotane) EPAR - Product information. [European Medicines Agency]. https://www.ema.europa.eu/en/medicines/human/EPAR/lysodren. Accessed 18 Jan 2020.

514. Fulton B, Benfield P. Moclobemide. An update of its pharmacological properties and therapeutic use. Drugs. 1996;52:450-74.

515. Hackett LP, Joyce DA, Hall RW, Dusci LJ, Ilett KF. Disposition and clinical effects of moclobemide and three of its metabolites following overdose. Drug Invest. 1993;5:281-4.

516. Iwersen S, Schmoldt A. Three suicide attempts with moclobemide. J Toxicol Clin Toxicol. 1996;34(2):223-5.

517. Mayersohn M, Guentert TW. Clinical pharmacokinetics of the monoamine oxidase-A inhibitor moclobemide. Clin Pharmacokinet. 1995;29:292-332.

518. Myrenfors PG, Eriksson T, Sandsted CS, Sjoberg G. Moclobemide overdose. J Intern Med. 1993;233:113-5.

519. Neuvonen PJ, Pohjola-Sintonen S, Tacke U, Vuori E. Five fatal cases of serotonin syndrome after moclobemide-citalopram or moclobemide-clomipramine overdoses. Lancet. 1993;342:1419.

520. Moachon G, Kanmacher I, Clenet M, Matinier D. Pharmacokinetic profile of modafinil. Drugs Today. 1996;32:327-37.

521. Zhao JJ, Rogers JD, Holland SD, Larson P, Amin RD, Haesen R, et al. Pharmacokinetics and bioavailability of montelukast sodium (MK-0476) in healthy young and elderly volunteers. Biopharm Drug Dispos. 1997;18:769-77.

522. Aderjan R, Schmitt G, Hofmann S. Morphin und dessen Glucuronide im Serum von Heroinabhängigen [in German]. Toxichem Krimtech. 1994;61:24-9.

523. Glare PA, Walsh TD. Clinical pharmacokinetics of morphine. Ther Drug Monit. 1991;13:1-23.

524. June HL, Stitzer ML, Cone E. Acute physical dependence: time course and relation to human plasma morphine concentrations. Clin Pharmacol Ther. 1995;57:270-80.

525. Lugo RA, Kern SE. Clinical pharmacokinetics of morphine. J Pain Palliat Care Pharmacother. 2002;16:5-18.

526. Behrend M. Mycophenolate mofetil: suggested guidelines for use in kidney transplantation. BioDrugs. 2001;15:37-53.

527. Fulton B, Markham A. Mycophenolate mofetil. A review of its pharmacodynamic and pharmacokinetic properties and clinical efficacy in renal transplantation. Drugs. 1996;51:278-98.

528. Hübner GI, Eismann R, Sziegoleit W. Relationship between mycophenolate mofetil side effects and mycophenolic acid plasma trough levels in renal transplant patients. Arzneim-Forsch/Drug Res. 2000;50:936-40.

529. Sanquer S, Breil M, Baron C, Dahmane D, Astier A, Lang P. Trough blood concentrations in long-term treatment with mycophenolate mofetil. Lancet. 1998;351:1557.

530. Willkens RF. An overview of the long-term safety experience of nabumetone. Drugs. 1990;40:34-7.

531. Derungs A, Schietzel S, Meyer MR, Maurer HH, Krahenbuhl S, Liechti ME. Sympathomimetic toxicity in a case of analytically confirmed recreational use of naphyrone (naphthylpyrovalerone). Clin Toxicol (Phila). 2011;49:691-3.

532. Davies NM, Anderson KE. Clinical pharmacokinetics of naproxen. Clin Pharmacokinet. 1997;32:268-93.

533. Marzo A, Dal BL, Wool C, Cerutti R. Bioavailability, food effect and tolerability of S-naproxen betainate sodium salt monohydrate in steady state. Arzneimittelforschung. 1998;48:935-40.

534. Heinroth KM, Kuhn C, Walper R, Busch I, Winkler M, Prondzinsky R. Akute Intoxikation mit dem ß1-selektiven ß-Rezeptorenblocker Nebivolol in suizidaler Absicht [in German]. Dtsch Med Wochenschr. 1999;124:1230-4.

535. Himmelmann A, Hedner T, Snoeck E, Lundgren B, Hedner J. Haemodynamic effects and pharmacokinetics of oral d- and l-nebivolol in hypertensive patients. Eur J Clin Pharmacol. 1996;51:259-64.

536. McNeely W, Goa KL. Nebivolol in the management of essential hypertension: a review. Drugs. 1999;57:633-51.

537. Barbhaiya RH, Shukla UA, Chaikin P, Greene DS, Marathe PH. Nefazodone pharmacokinetics: assessment of nonlinearity, intra-subject variability and time to attain steady-state plasma concentrations after dose escalation and de-escalation. Eur J Clin Pharmacol. 1996;50:101-7.

538. Davis R, Whittington R, Bryson HM. Nefazodone. A review of its pharmacology and clinical efficacy in the management of major depression. Drugs. 1997;53:608-36.

539. Gaffney PN, Schuckman HA, Beeson MS. Nefazodone overdose. Ann Pharmacother. 1998;32:1249-50.

540. Greene DS, Barbhaiya RH. Clinical pharmacokinetics of nefazodone. Clin Pharmacokinet. 1997;33:260-75.

541. Kaul S, Shukla UA, Barbhaiya RH. Nonlinear pharmacokinetics of nefazodone after escalating single and multiple oral doses. J Clin Pharmacol. 1995;35:830-9.

542. Salazar DE, Marathe PH, Fulmor IE, Lee JS, Raymond RH, Uderman HD. Pharmacokinetic and pharmacodynamic evaluation during coadministration of nefazodone and propranolol in healthy men. J Clin Pharmacol. 1995;35:1109-18.

543. Calvey TN, Wareing M, Williams NE, Chan K. Pharmacokinetics and pharmacological effects of neostigmine in man. Br J Clin Pharmacol. 1979;7:149-55.

544. Corkery JM, Button J, Vento AE, Schifano F. Two UK suicides using nicotine extracted from tobacco employing instructions available on the Internet. Forensic Sci Int. 2010;199(1-3):e9-13.

545. Schneider S, Diederich N, Appenzeller B, Schartz A, Lorang C, Wennig R. Internet suicide guidelines: Report of a life threatening poisoning using tobacco extract. Toxichem Krimtech. 2008;75:134-6.

546. Bernareggi A. Clinical pharmacokinetics of nimesulide. Clin Pharmacokinet. 1998;35:247-74.

547. Bakdash A, Ganswindt M, Herre S, Nakulski T, Pragst F. Lethal poisoning with p-nitroaniline. Toxichem Krimtech. 2006;73:61-5.

548. Moller Jensen K, Berg Dahl J. Plasma concentrations of glyceryl trinitrate and its dinitrate metabolites after sublingual administration to volunteers. Simultaneous determination of glyceryl trinitrate and its dinitrate metabolites. Arzneim-Forsch/Drug Res. 1994;44:951-3.

549. Thiermann H, Mast U, Eyer P, Hilber A, Pfab R, Felgenhauer J, et al. Parathion poisoning: pharmacokinetics and laboratory findings during continuous infusion of obidoime and atropine [Abstract]. Naunyn-Schmiedeberg's Arch Pharmacol. 1996;353:R146.

550. Lamp KC, Bailey EM, Rybak MJ. Ofloxacin clinical pharmacokinetics. Clin Pharmacokinet. 1992;22:32-46.

551. Callaghan JT, Bergstrom RF, Ptak LR, Beasley CM. Olanzapine. Pharmacokinetic and pharmacodynamic profile. Clin Pharmacokinet. 1999;37:177-93.

552. Citrome L, Stauffer VL, Chen L, Kinon BJ, Kurtz DL, Jacobson JG, et al. Olanzapine plasma concentrations after treatment with 10, 20, and 40 mg/d in patients with schizophrenia: an analysis of correlations with efficacy, weight gain, and prolactin concentration. J Clin Psychopharmacol. 2009;29:278-83.

553. Elian AA. Fatal overdose of olanzepine [olanzapine]. Forensic Sci Int. 1998;91(3):231-5.

554. Perry PJ, Lund BC, Sanger T, Beasley C. Olanzapine plasma concentrations and clinical response: acute phase results of the North American Olanzapine Trial. J Clin Psychopharmacol. 2001;21:14-20.

555. Shrestha M, Hendrickson RG, Henretig FM. Striking extrapyramidal movements seen in large olanzapine overdose. Clin Toxicol. 2001;39:282.

556. Kees F, Jehkul A, Bucher M, Mair G, Kiermaier J, Grobecker H. Bioavailability of opipramol from a film-coated tablet, a sugar-coated tablet and an aqueous solution in healthy volunteers. Arzneimittelforschung. 2003;53:87-92.

557. Van Herreweghe I, Mertens K, Maes V, Ramet J. Orphenadrine poisoning in a child: clinical and analytical data. Intensive Care Med. 1999;25:1134-6.

558. Gonzalez-Esquivel DF, Ortega-Gavilan M, Alcantara-Lopez G, Jung-Cook H. Plasma level monitoring of oxcarbazepine in epileptic patients. Arch Med Res. 2000;31:202-5.

559. May TW, Korn-Merker E, Rambeck B. Clinical pharmacokinetics of oxcarbazepine. Clin Pharmacokinet. 2003;42:1023-42.

560. Theisohn M, Heimann G. Disposition of the antiepileptic oxcarbazepine and its metabolites in healthy volunteers. Eur J Clin Pharmacol. 1982;22:545-51.

561. Cremers S, Sparidans R, den HJ, Hamdy N, Vermeij P, Papapoulos S. A pharmacokinetic and pharmacodynamic model for intravenous bisphosphonate (pamidronate) in osteoporosis. Eur J Clin Pharmacol. 2002;57:883-90.

562. Vandenbrom RH, Wierda JM. Pancuronium bromide in the intensive care unit: a case of overdose. Anesthesiology. 1988;69:996-7.

563. Bond GR, Krenzelok EP, Normann SA, Tendler JD, Morris-Kukoski CL, McCoy DJ, et al. Acetaminophen ingestion in childhood--cost and relative risk of alternative referral strategies. J Toxicol Clin Toxicol. 1994;32:513-25.

564. Graudins A, Aaron CK, Linden CH. Overdose of extended-release acetaminophen. N Engl J Med. 1995;333:196.

565. Kamali F, Edwards C, Rawlins MD. The effect of pirenzepine on gastric emptying and salivary flow rate: constraints on the use of saliva paracetamol concentrations for the determination of paracetamol pharmacokinetics. Br J Clin Pharmacol. 1992;33:309-12.

566. van der Marel CD, van Lingen RA, Pluim MA, Scoones G, van DM, Vaandrager JM, et al. Analgesic efficacy of rectal versus oral acetaminophen in children after major craniofacial surgery. Clin Pharmacol Ther. 2001;70:82-90.

567. Nielsen JC, Bjerring P, Arendt-Nielsen L. A comparison of the hypoalgesic effect of paracetamol in slow-release and plain tablets on laser-induced pain. Br J Clin Pharmacol. 1991;31:267-70.

568. Rose SR. Subtleties of managing acetaminophen poisoning. Am J Hosp Pharm. 1994;51:3065-8.

569. Schiodt FV, Ott P, Christensen E, Bondesen S. The value of plasma acetaminophen half-life in antidote-treated acetaminophen overdosage. Clin Pharmacol Ther. 2002;71:221-5.

570. Smilkstein MJ, Douglas DR, Daya MR. Acetaminophen poisoning and liver function. N Engl J Med. 1994;331:1310-1.

571. Vale JA, Proudfoot AT. Paracetamol (acetaminophen) poisoning. Lancet. 1995;346:547-52.

572. Hart TB, Nevitt A, Whitehead A. A new statistical approach to the prognostic significance of plasma paraquat concentrations. Lancet. 1984;2:1222-3.

573. Bismuth C, Garnier R, Baud FJ, Muszynski J, Keyes C. Paraquat poisoning. An overview of the current status. Drug Saf. 1990;5:243-51.

574. Dinis-Oliveira RJ, Duarte JA, Sanchez-Navarro A, Remiao F, Bastos ML, Carvalho F. Paraquat poisonings: mechanisms of lung toxicity, clinical features, and treatment. Crit Rev Toxicol. 2008;38:13-71.

575. Fairshter RD, Dabir-Vaziri N, Smith WR, Glauser FL, Wilson AF. Paraquat poisoning: an analytical toxicologic study of three cases. Toxicology. 1979;12:259-66.

576. Gil HW, Kang MS, Yang JO, Lee EY, Hong SY. Association between plasma paraquat level and outcome of paraquat poisoning in 375 paraquat poisoning patients. Clin Toxicol (Phila). 2008;46:515-8.

577. Gawarammana IB, Buckley NA. Medical management of paraquat ingestion. Br J Clin Pharmacol. 2011;72:745-57.

578. Houze P, Baud FJ, Mouy R, Bismuth C, Bourdon R, Scherrmann JM. Toxicokinetics of paraquat in humans. Hum Exp Toxicol. 1990;9:5-12.

579. Kang MS, Gil HW, Yang JO, Lee EY, Hong SY. Comparison between kidney and hemoperfusion for paraquat elimination. J Korean Med Sci. 2009;24 Suppl: S156-60.

580. Lheureux P, Leduc D, Vanbinst R, Askenasi R. Survival in a case of massive paraquat ingestion. Chest. 1995;107:285-9.

581. Vermeulen T. Distribution of paroxetine in three postmortem cases. J Anal Toxicol. 1998;22(6):541-4.

582. Goeringer KE, Raymon L, Christian GD, Logan BK. Postmortem forensic toxicology of selective serotonin reuptake inhibitors: a review of pharmacology and report of 168 cases. J Forensic Sci. 2000;45(3):633-48.

583. Sindrup SH, Grodum E, Gram LF, Beck-Nielsen H. Concentration-response relationship in paroxetine treatment of diabetic neuropathy symptoms: a patient-blinded dose-escalation study. Ther Drug Monit. 1991;13:408-14.

584. Gosciniak H-T. Suizid mit Perazin [in German]. Psychopharmakotherapie. 1997;4:105.

585. Amoah AG, Gould BJ, Parke DV, Lockhart JD. Further studies on the pharmacokinetics of perhexiline maleate in humans. Xenobiotica. 1986;16:63-8.

586. Jones TE, Morris RG, Horowitz JD. Concentration-time profile for perhexiline and hydroxyperhexiline in patients at steady state. Br J Clin Pharmacol. 2004;57:263-9.

587. Linnet K, Wiborg O. Steady-state serum concentrations of the neuroleptic perphenazine in relation to CYP2D6 genetic polymorphism. Clin Pharmacol Ther. 1996;60:41-7.

588. Armstrong PJ, Bersten A. Normeperidine toxicity. Anesth Analg. 1986;65:536-8.

589. Baumann TJ, Smythe MA, Marikis B, Bivins BA. Meperidine serum concentrations and analgesic response in postsurgical patients. DICP. 1991;25(7-8):724-7.

590. Hagmeyer KO, Mauro LS, Mauro VF. Meperidine-related seizures associated with patient-controlled analgesia pumps. Ann Pharmacother. 1993;27(1):29-32.

591. Holmberg L, Odar-Cederlof I, Boreus LO, Heyner L, Ehrnebo M. Comparative disposition of pethidine and norpethidine in old and young patients. Eur J Clin Pharmacol. 1982;22:175-9.

592. Kaiko RF, Foley KM, Grabinski PY, Heidrich G, Rogers AG, Inturrisi CE, et al. Central nervous system excitatory effects of meperidine in cancer patients. Ann Neurol. 1983;13:180-5.

593. Anderson GD, Pak C, Doane KW, Griffy KG, Temkin NR, Wilensky AJ, et al. Revised Winter-Tozer equation for normalized phenytoin concentrations in trauma and elderly patients with hypoalbuminemia. Ann Pharmacother. 1997;31:279-84.

594. Brandolese R, Scordo MG, Spina E, Gusella M, Padrini R. Severe phenytoin intoxication in a subject homozygous for CYP2C9*3. Clin Pharmacol Ther. 2001;70:391-4.

595. Frey OR, von Brenndorff AI, Probst W. Comparison of phenytoin serum concentrations in premature neonates following intravenous and oral administration. Ann Pharmacother. 1998;32:300-3.

596. Hayes G, Kootsikas ME. Reassessing the lower end of the phenytoin therapeutic range: a review of the literature. Ann Pharmacother. 1993;27:1389-92.

597. Howard CE, Roberts RS, Ely DS, Moye RA. Use of multiple-dose activated charcoal in phenytoin toxicity. Ann Pharmacother. 1994;28:201-3.

598. Mlynarek ME, Peterson EL, Zarowitz BJ. Predicting unbound phenytoin concentrations in the critically ill neurosurgical patient. Ann Pharmacother. 1996;30:219-23.

599. Murphy JM, Motiwala R, Devinsky O. Phenytoin intoxication. South Med J. 1991;84:1199-204.

600. Privitera MD. Clinical rules for phenytoin dosing. Ann Pharmacother. 1993;27:1169-73.

601. Asthana S, Greig NH, Hegedus L, Holloway HH, Raffaele KC, Schapiro MB, et al. Clinical pharmacokinetics of physostigmine in patients with Alzheimer's disease. Clin Pharmacol Ther. 1995;58:299-309.

602. Kietzmann D, Hamm C, Bouillon T, Kettler D, Gundert-Remy U. Concentration-effect relationship of piritramid (Dipidolor®) in a postoperative pain model [Abstract]. Naunyn-Schmiedeberg's Arch Pharmacol. 1994;349(Suppl.):R139.

603. Heinz W, Trebesch I, Ulrich A, Klöser C. Ist ein therapeutisches Drug-Monitoring bei Azolen sinnvoll? [Abstract] [in German]. Med Klin. 2008;103 (Suppl.):63.

604. Hohmann C, Kang EM, Jancel T. Rifampin and posaconazole coadministration leads to decreased serum posaconazole concentrations. Clin Infect Dis. 2010;50:939-40.

605. Walsh TJ, Raad I, Patterson TF, Chandrasekar P, Donowitz GR, Graybill R, et al.. Treatment of invasive aspergillosis with posaconazole in patients who are refractory to or intolerant of conventional therapy: an externally controlled trial. Clin Infect Dis. 2007;44:2-12.

606. Shoji S, Suzuki M, Tomono Y, Bockbrader HN, Matsui S. Population pharmacokinetics of pregabalin in healthy subjects and patients with post-herpetic neuralgia or diabetic peripheral neuropathy. Br J Clin Pharmacol. 2011;72:63-76.

607. Wood DM, Berry DJ, Glover G, Eastwood J, Dargan PI. Significant pregabalin toxicity managed with supportive care alone. J Med Toxicol. 2010;6:435-7.

608. Goggin M, Crowley K, O'Malley K, Barry P, Kelly G, Blake J. Serum concentrations of prilocaine following retrobulbar block. Br J Anaesth. 1990;64:107-9.

609. Moffett BS, Cannon BC, Friedman RA, Kertesz NJ. Therapeutic levels of intravenous procainamide in neonates: a retrospective assessment. Pharmacotherapy. 2006;26:1687-93.

610. Edstein MD, Veenendaal JR, Scott HV, Rieckmann KH. Steady-state kinetics of proguanil and its active metabolite, cycloguanil, in man. Chemotherapy. 1988;34:385-92.

611. Gandia P, Saivin S, Le-Traon AP, Guell A, Houin G. Influence of simulated weightlessness on the intramuscular and oral pharmacokinetics of promethazine in 12 human volunteers. J Clin Pharmacol. 2006;46:1008-16.

612. Strenkoski-Nix LC, Ermer J, DeCleene S, Cevallos W, Mayer PR. Pharmacokinetics of promethazine hydrochloride after administration of rectal suppositories and oral syrup to healthy subjects. Am J Health Syst Pharm. 2000;57:1499-505.

613. Janousek J, Paul T, Reimer A, Kallfelz HC. Usefulness of propafenone for supraventricular arrhythmias in infants and children. Am J Cardiol. 1993;72:294-300.

614. Hartvig P, Roos BE, Ahs U, Ryde M. Pharmacokinetics of propiomazine following intravenous, intramuscular and oral administration with special reference to the elimination phase. Curr Ther Res.1981;29:351-62.

615. Arndt GA, Reiss WG, Bathke KA, Springman SR, Kenny G. The estimated plasma concentration (EPC) at which patients are induced and awake from general anesthesia with propofol. Clin Pharmacol Ther. 1993;53:224.

616. Iwersen-Bergmann S, Rosner P, Kuhnau HC, Junge M, Schmoldt A. Death after excessive propofol abuse. Int J Legal Med. 2001;114:248-51.

617. Brooks DE, Wallace KL. Acute propylene glycol ingestion. J Toxicol Clin Toxicol. 2002;40:513-6.

618. Centers for Disease Control and Prevention (CDC). Infant deaths associated with cough and cold medications – two states, 2005. MMWR Morb Mortal Wkly Rep. 2007;56:1-4.

619. Wingert WE, Mundy LA, Collins GL, Chmara ES. Possible role of pseudoephedrine and other over-the-counter cold medications in the deaths of very young children. J Forensic Sci. 2007;52:487-90.

620. Douglas JG, McLeod MJ. Pharmacokinetic factors in the modern drug treatment of tuberculosis. Clin Pharmacokinet. 1999;37:127-46.

621. White MC, De SP, Havard CW. Plasma pyridostigmine levels in myasthenia gravis. Neurology. 1981;31:145-50.

622. Williams NE, Calvey TN, Chan K. Plasma concentration of pyridostigmine during the antagonism of neuromuscular block. Br J Anaesth. 1983;55:27-31.

623. Arvanitis LA, Miller BG. Multiple fixed doses of "Seroquel" (quetiapine) in patients with acute exacerbation of schizophrenia: a comparison with haloperidol and placebo. The Seroquel Trial 13 Study Group. Biol Psychiatry. 1997;42:233-46.

624. DeVane CL, Nemeroff CB. Clinical pharmacokinetics of quetiapine: an atypical antipsychotic. Clin Pharmacokinet. 2001;40:509-22.

625. Harmon TJ, Benitez JG, Krenzelok EP, Cortes-Belen E. Loss of consciousness from acute quetiapine overdosage. J Toxicol Clin Toxicol. 1998;36:599-602.

626. Isbister GK, Friberg LE, Hackett LP, Duffull SB. Pharmacokinetics of quetiapine in overdose and the effect of activated charcoal. Clin Pharmacol Ther. 2007;81:821-7.

627. Nudelman E, Vinuela LM, Cohen CI. Safety in overdose of quetiapine: a case report. J Clin Psychiatry. 1998;59:433.

628. Pollak PT, Zbuk K. Quetiapine fumarate overdose: clinical and pharmacokinetic lessons from extreme conditions. Clin Pharmacol Ther. 2000;68:92-7.

629. von Düsterlho J, Homann J. Lebensbedrohliche Herzrhythmusstörungen unter Chinin-Medikation [in German]. Dtsch Med Wochenschr. 1995;120:542-3.

630. Paintaud G, Alvan G, Berninger E, Gustafsson LL, Idrizbegovic E, Karlsson KK, et al. The concentration-effect relationship of quinine-induced hearing impairment. Clin Pharmacol Ther. 1994;55:317-23.

631. Mignon M, Chau NP, Nguyen-Phuoc BK, Sauvage M, Leguy F, Bonfils S. Ranitidine upon meal-induced gastric secretion: oral pharmacokinetics and plasma concentration effect relationships. Br J Clin Pharmacol. 1982;14:187-93.

632. Fleishaker JC. Clinical pharmacokinetics of reboxetine, a selective norepinephrine reuptake inhibitor for the treatment of patients with depression. Clin Pharmacokinet. 2000;39:413-27.

633. Härtter S. Moderne Antidepressiva: Pharmakokinetik, Interaktionspotenzial und TDM [in German]. Pharm Unserer Zeit. 2004;33:296-303.

634. Anderson JL, Reddy CP, Myerburg RJ, Waxman HL, de Vane PJ. Antiarrhythmic and pharmacokinetic evaluation of intravenous recainam in patients with frequent ventricular premature complexes and unsustained ventricular tachycardia. Am J Cardiol. 1993;71:686-94.

635. Jostell KG, Lapierre YD. Plasma concentration of remoxipride in relation to antipsychotic effect and adverse symptoms. The Canadian Remoxipride Study Group. Acta Psychiatr Scand Suppl. 1990;358:48-50.

636. Lappenberg-Pelzer M, Baudisch H. Ein Todesfall nach Remoxiprideinnahme [in German]. Toxichem Krimtech. 1994;61:10.

637. Michaelsson K, Lithell H, Vessby B, Melhus H: Serum retinol levels and the risk of fracture. N Engl J Med. 2003;348:287-94.

638. Kopferschmitt J, Flesch F, Lugnier A, Sauder P, Jaeger A, Mantz JM. Acute voluntary intoxication by ricin. Hum Toxicol. 1983;2:239-42.

639. Plomp TA, Battista HJ, Unterdorfer H, van Ditmarsch WC, Maes RA. A case of fatal poisoning by rifampicin. Arch Toxicol. 1981;48:245-52.

640. Peruche B, Schulz M. Risperidon, ein neues atypisches Neuroleptikum [in German]. Pharm Ztg. 1996;141(32):2920-4.

641. Seto K, Dumontet J, Ensom MH. Risperidone in schizophrenia: is there a role for therapeutic drug monitoring? Ther Drug Monit. 2011;33:275-83.

642. Hsu A, Granneman GR, Bertz RJ. Ritonavir. Clinical pharmacokinetics and interactions with other anti-HIV agents. Clin Pharmacokinet. 1998;35:275-91.

643. Darreh-Shori T, Jelic V. Safety and tolerability of transdermal and oral rivastigmine in Alzheimer's disease and Parkinson's disease dementia. Expert Opin Drug Saf. 2010;9:167-76.

644. Dhillon S. Rivastigmine transdermal patch: a review of its use in the management of dementia of the Alzheimer's type. Drugs. 2011;71:1209-31.

645. Kaye CM, Nicholls B. Clinical pharmacokinetics of ropinirole. Clin Pharmacokinet. 2000;39:243-54.

646. Emanuelsson B-M, Norsten-Höög C, Sandberg R, Sjövall J. Ropivacaine and its 2H3-labelled analogue – bioanalysis and disposition in healthy volunteers. Eur J Pharm Sci. 1997;5:171-7.

647. Markham A, Faulds D. Ropivacaine. A review of its pharmacology and therapeutic use in regional anaesthesia. Drugs. 1996;52:429-49.

648. Scott DB, Lee A, Fagan D, Bowler GM, Bloomfield P, Lundh R. Acute toxicity of ropivacaine compared with that of bupivacaine. Anesth Analg. 1989;69:563-9.

649. Lewis LD, Essex E, Volans GN, Cochrane GM. A study of self poisoning with oral salbutamol--laboratory and clinical features. Hum Exp Toxicol. 1993;12:397-401.

650. Moser M, Buchberger W. Selenium status of elderly Austrians suffering from circulation disorders. Wien Klin Wochenschr. 1993;105:497-9.

651. Müller D, Desel H. Problematik, Klinik und Beispiele der Spurenelementvergiftung - Selen [in German]. Toxichem Krimtech. 2012;79:5-16.

652. Aldosary BM, Sutter ME, Schwartz M, Morgan BW. Case series of selenium toxicity from a nutritional supplement. Clin Toxicol (Phila). 2012;50:57-64.

653. Milner DA, Hall M, Davis GG, Brissie RM, Robinson CA. Fatal multiple drug intoxication following acute sertraline use. J Anal Toxicol. 1998;22:545-8.

654. Kahan BD, Napoli KL. Role of therapeutic drug monitoring of rapamycin. Transplant Proc. 1998;30:2189-91.

655. MacDonald A, Scarola J, Burke JT, Zimmerman JJ. Clinical pharmacokinetics and therapeutic drug monitoring of sirolimus. Clin Ther. 2000;22(Suppl. B):B101-21.

656. Mahalati K, Kahan BD. Clinical pharmacokinetics of sirolimus. Clin Pharmacokinet. 2001;40:573-85.

657. Zimmerman JJ, Kahan BD. Pharmacokinetics of sirolimus in stable renal transplant patients after multiple oral dose administration. J Clin Pharmacol. 1997;37:405-15.

658. Edvardsson N, Varnauskas E. Clinical course serum concentrations and elimination rate in a case of massive sotalol intoxication. Pharmacokinetics. 1989;6:558.

659. Steinecke H, Stein U, Lang J, Kluge S, Hentschel H, Muth G. Intoxikation mit Sotalol [in German]. Toxichem Krimtech. 1999;66:100-2.

660. Brook I. Pharmacodynamics and pharmacokinetics of spiramycin and their clinical significance. Clin Pharmacokinet. 1998;34:303-10.

661. Edmunds M, Sheehan TM, Van't Hoff W. Strychnine poisoning: clinical and toxicological observations on a non-fatal case. J Toxicol Clin Toxicol. 1986; 24:245-55.

662. Heiser JM, Daya MR, Magnussen AR, Norton RL, Spyker DA, Allen DW, et al. Massive strychnine intoxication: serial blood levels in a fatal case. J Toxicol Clin Toxicol. 1992;30:269-83.

663. Oberpaur B, Donoso A, Claveria C, Valverde C, Azocar M. Strychnine poisoning: an uncommon intoxication in children. Pediatr Emerg Care. 1999;15:264-5.

664. Palatnick W, Meatherall R, Sitar D, Tenenbein M. Toxicokinetics of acute strychnine poisoning. J Toxicol Clin Toxicol. 1997;35:617-20.

665. Rosano TG, Hubbard JD, Meola JM, Swift TA. Fatal strychnine poisoning: application of gas chromatography and tandem mass spectrometry. J Anal Toxicol. 2000;24:642-7.

666. Winek CL, Wahba WW, Esposito FM, Collom WD. Fatal strychnine ingestion. J Anal Toxicol. 1986;10:120-1.

667. Wood DM, Webster E, Martinez D, Dargan PI, Jones AL. Case report: Survival after deliberate strychnine self-poisoning, with toxicokinetic data. Critical Care. 2002;6:456-9.

668. Bailey JM, Schwieger IM, Hug CC, Jr. Evaluation of sufentanil anesthesia obtained by a computer-controlled infusion for cardiac surgery. Anesth Analg. 1993;76:247-52.

669. Borenstein M, Shupak RC, Barnette RE, Cooney GF, Tzeng T-B. Cardiovascular effects of different infusion rate of sufentanil (S) in patients undergoing coronary surgery (CS). Clin Pharmacol Ther. 1994;55:129.

670. Haynes G, Brahen NH, Hill HF. Plasma sufentanil concentration after intranasal administration to paediatric outpatients. Can J Anaesth. 1993;40:286.

671. Scholz J, Bause H, Schulz M, Klotz U, Krishna DR, Pohl S, et al. Pharmacokinetics and effects on intracranial pressure of sufentanil in head trauma patients. Br J Clin Pharmacol. 1994;38:369-72.

672. Paap CM, Nahata MC. Clinical use of trimethoprim/sulfamethoxazole during renal dysfunction. DICP. 1989;23:646-54.

673. Davies NM, Watson MS. Clinical pharmacokinetics of sulindac. A dynamic old drug. Clin Pharmacokinet. 1997;32:437-59.

674. Larsen AK. Suramin: an anticancer drug with unique biological effects. Cancer Chemother Pharmacol. 1993;32:96-8.

675. Hooks MA. Tacrolimus, a new immunosuppressant--a review of the literature. Ann Pharmacother. 1994;28:501-11.

676. Jusko WJ, Thomson AW, Fung J, McMaster P, Wong SH, Zylber-Katz E, et al. Consensus document: therapeutic monitoring of tacrolimus (FK-506). Ther Drug Monit. 1995;17:606-14.

677. Ahrend KF, Nagy L, Tiess D. [Morphology and analysis of sulthiame intoxication]. [in German]. Arch Toxikol. 1969;24(3):229-37.

678. Takahara S, Kokado Y, Kameoka H, Takano Y, Jiang H, Moutabarrik A, et al. Monitoring of FK 506 blood levels in kidney transplant recipients. Transplant Proc. 1994;26:2106-8.

679. Undre N, Moller A. Pharmacokinetic interpretation of FK 506 levels in blood and in plasma during a European randomised study in primary liver transplant patients. The FK 506 European Study Group. Transpl Int. 1994;7(Suppl. 1):S15-21.

680. Cantrell FL, Mallett P, Aldridge L, Verilhac K, McIntyre IM. A tapentadol related fatality: Case report with postmortem concentrations. Forensic Sci Int. 2016;266:e1-e3.

681. Venkataramanan R, Swaminathan A, Prasad T, Jain A, Zuckerman S, Warty V, et al. Clinical pharmacokinetics of tacrolimus. Clin Pharmacokinet. 1995;29:404-30.

682. Venkataramanan R, Shaw LM, Sarkozi L, Mullins R, Pirsch J, MacFarlane G, et al. Clinical utility of monitoring tacrolimus blood concentrations in liver transplant patients. J Clin Pharmacol. 2001;41:542-51.

683. Wallemacq PE, Verbeeck RK. Comparative clinical pharmacokinetics of tacrolimus in paediatric and adult patients. Clin Pharmacokinet. 2001;40:283-95.

684. Franco DM, Ali Z, Levine B, Middleberg RA, Fowler DR. Case report of a fatal intoxication by Nucynta. Am J Forensic Med Pathol. 2014;35(4):234-6.

685. Terhaag B, Grünert A, Richter K, Bahlmann G, Gloris A. Zur Effektivität der Hämoperfusion bei einer Talinolol-Intoxikation - ein Fallbericht [in German]. Z Klin Med. 1987;42:1463-4.

686. Krueger M, Achenbach H, Terhaag B, Haase H, Richter K, Oertel R, Preiss R. Pharmacokinetics of oral talinolol following a single dose and during steady state in patients with chronic renal failure and healthy volunteers. Int J Clin Pharmacol Ther. 2001;39(2):61-6.

687. Connors KP, Kuti JL, Nicolau DP. Optimizing antibiotic pharmacodynamics for clinical practice. Pharm Anal Acta. 2013;4(3):214.

688. Wilson AP. Clinical pharmacokinetics of teicoplanin. Clin Pharmacokinet. 2000;39:167-83.

689. Forrest AR, Marsh I, Bradshaw C, Braich SK. Fatal temazepam overdoses. Lancet. 1986;2(8500):226.

690. Heintz RC, Guentert TW, Enrico JF, Dubach UC, Brandt R, Jeunet FS. Pharmacokinetics of tenoxicam in healthy human volunteers. Eur J Rheumatol Inflamm. 1984;7(2):33-44.

691. Nilsen OG. Clinical pharmacokinetics of tenoxicam. Clin Pharmacokinet. 1994;26:16-43.

692. Woosley RL, Chen Y, Freiman JP, Gillis RA. Mechanism of the cardiotoxic actions of terfenadine. JAMA. 1993;269:1532-6.

693. Brodersen HP, Korsten S, Larbig D. Eliminationsverfahren bei potentiell letaler Thalliumintoxikation [in German]. Dtsch Med Wochenschr. 1995;120:1301.

694. Klemm M, Meißner D. Problematik, Klinik und Beispiele der Spurenelementvergiftung – Thallium [in German]. Toxichem Krimtech. 2012;79:17-22.

695. Dager WE, Albertson TE. Impact of therapeutic drug monitoring of intravenous theophylline regimens on serum theophylline concentrations in the medical intensive care unit. Ann Pharmacother. 1992;26:1287-91.

696. Dettloff RW, Touchette MA, Zarowitz BJ. Vasopressor-resistant hypotension following a massive ingestion of theophylline. Ann Pharmacother. 1993;27:781-4.

697. Elias-Jones AC, Larcher VF, Shaw PN. An investigation into the relationship between liver impairment and theophylline pharmacokinetics in children. Pharm Pharmacol Lett. 1992;2:115-8.

698. Hallas J, Davidsen O, Grodum E, Damsbo N, Gram LF. Drug-related illness as a cause of admission to a department of respiratory medicine. Respiration. 1992;59:30-4.

699. Holford N, Black P, Couch R, Kennedy J, Briant R. Theophylline target concentration in severe airways obstruction - 10 or 20 mg/L? A randomised concentration-controlled trial. Clin Pharmacokinet. 1993;25:495-505.

700. Kirk JK, Dupuis RE, Miles MV, Gaddy GD, Miranda-Massari JR, Williams DM. Salivary theophylline monitoring: reassessment and clinical considerations. Ther Drug Monit. 1994;16:58-66.

701. Phillips BA, Chrystyn H. The role of bayesian analysis to interpret serum theophylline concentrations in the community sector [Abstract]. Pharm J. 1991;247:R18.

702. Martin CD, Chan SC. Distribution of temazepam in body fluids and tissues in lethal overdose. J Anal Toxicol. 1986;10(2):77-8.

703. Shannon M. Predictors of major toxicity after theophylline overdose. Ann Intern Med. 1993;119:1161-7.

704. Sullivan P, Bekir S, Jaffar Z, Page C, Jeffery P, Costello J. Anti-inflammatory effects of low-dose oral theophylline in atopic asthma. Lancet. 1994;343:1006-8.

705. Wessel T, Unger W, Wilhelms E. Theophyllin: Drug monitoring beim internistischen Patienten sinnvoll? Versuch einer Bewertung für Arzt und Apotheker [in German]. Krankenhauspharmazie. 1996;17:335-9.

706. Ahearn DJ, Grim CE. Treatment of malignant hypertension with sodium nitroprusside. Arch Intern Med. 1974;133:187-91.

707. Koniaris LG, Zimmers TA, Lubarsky DA, Sheldon JP. Inadequate anaesthesia in lethal injection for execution. Lancet. 2005;365:1412-4.

708. Adkins JC, Noble S. Tiagabine. A review of its pharmacodynamic and pharmacokinetic properties and therapeutic potential in the management of epilepsy. Drugs. 1998;55:437-60.

709. Leach JP, Stolarek I, Brodie MJ. Deliberate overdose with the novel anticonvulsant tiagabine. Seizure. 1995;4:155-7.

710. Leach JP, Brodie MJ. Tiagabine. Lancet. 1998;351:203-7.

711. Luer MS, Rhoney DH. Tiagabine: a novel antiepileptic drug. Ann Pharmacother. 1998;32:1173-80.

712. Perucca E, Bialer M. The clinical pharmacokinetics of the newer antiepileptic drugs. Focus on topiramate, zonisamide and tiagabine. Clin Pharmacokinet. 1996:31:29-46.

713. Davies NM. Clinical pharmacokinetics of tiaprofenic acid and its enantiomers. Clin Pharmacokinet. 1996;31:331-47.

714. Pottier J, Cousty-Berlin D, Busigny M. Human pharmacokinetics of tiaprofenic acid. Rheumatology (Oxford). 1982;7:70-7.

715. Brennscheidt U, Brunnmüller U, Proppe D, Thomann P, Seiler KU. Pharmacokinetics of tilidine and naloxone in patients with severe hepatic impairment. Arzneimittelforschung. 2007;57(2):106-11.

716. Hajda JP, Jähnchen E, Oie S, Trenk D. Sequential first-pass metabolism of nortilidine: the active metabolite of the synthetic opioid drug tilidine. J Clin Pharmacol. 2002;42:1257-61.

717. Schwietert HR, Peeters PA, Dingemanse J, Thiercelin JF, Necciari J, de BH, et al. Multiple dose pharmacokinetics of tiludronate in healthy volunteers. Eur J Clin Pharmacol. 1996;51:175-81.

718. Gillet P, Gavriloff C, Hercelin B, Salles MF, Nicolas A, Netter P. Pharmacokinetics of tiopronin after repeated oral administration in rheumatoid arthritis. Fundam Clin Pharmacol. 1995;9:205-6.

719. Keam SJ, Keating GM. Tiotropium bromide. A review of its use as maintenance therapy in patients with COPD. Treat Respir Med. 2004;3:247-68.

720. Barnfield C, Kemmenoe AV. A sudden death due to tocainide overdose. Hum Toxicol. 1986;5:337-40.

721. Latini R, Maggioni AP, Cavalli A. Therapeutic drug monitoring of antiarrhythmic drugs. Rationale and current status. Clin Pharmacokinet. 1990;18:91-103.

722. Barthel W, Hüller G, Böhm C, Haustein K-O. Tolbutamid: Zwei Präparate im Vergleich [in German]. Pharm Ztg. 2007;141:4686.

723. Furman WL, Baker SD, Pratt CB, Rivera GK, Evans WE, Stewart CF. Escalating systemic exposure of continuous infusion topotecan in children with recurrent acute leukemia. J Clin Oncol. 1996;14:1504-11.

724. Herben VM, ten Bokkel Huinink WW, Beijnen JH. Clinical pharmacokinetics of topotecan. Clin Pharmacokinet. 1996;31:85-102.

725. Besson JM, Vickers MD: Tramadol analgesia. Synergy in research and therapy. Drugs. 1994;47(Suppl. 1):1-2.

726. Lehmann KA, Kratzenberg U, Schroeder-Bark B, Horrichs-Haermeyer G. Postoperative patient-controlled analgesia with tramadol: analgesic efficacy and minimum effective concentrations. Clin J Pain. 1990;6:212-20.

727. Iwersen S, Schmoldt A. One fatal and one nonfatal intoxication with tranylcypromine. Absence of amphetamines as metabolites. J Anal Toxicol. 1996;20:301-4.

728. Meyer FP. Trapidil (Rocornal) [in German]. Internist Prax. 1993;33:611-4.

729. Ohkubo T, Osanai T, Sugawara K, Ishida M, Otani K, Mihara K, et al. High-performance liquid chromatographic determination of trazodone and 1-m-chlorophenylpiperazine with ultraviolet and electrochemical detector. J Pharm Pharmacol. 1995;47:340-4.

730. Hukkinen SK, Varhe A, Olkkola KT, Neuvonen PJ. Plasma concentrations of triazolam are increased by concomitant ingestion of grapefruit juice. Clin Pharmacol Ther. 1995;58:127-31.

731. Olson KR, Yin L, Osterloh J, Tani A. Coma caused by trivial triazolam overdose. Am J Emerg Med. 1985;3(3):210-1.

732. Leader WG, Chandler MH, Castiglia M. Pharmacokinetic optimisation of vancomycin therapy. Clin Pharmacokinet. 1995;28:327-42.

733. MacGowan A, Lovering A, White L, Reeves D. Why monitor peak vancomycin concentrations? Lancet. 1995;345:645-7.

734. Fernandez de Gatta MD, Calvo MV, Hernandez JM, Caballero D, San Miguel JF, Dominguez-Gil A. Cost-effectiveness analysis of serum vancomycin concentration monitoring in patients with hematologic malignancies. Clin Pharmacol Ther. 1996;60:332-40.

735. Ye ZK, Li C, Zhai SD. Guidelines for therapeutic drug monitoring of vancomycin: a systematic review. PLoS One 2014;9(6): e99044.

736. Masich AM, Kalaria SN, Gonzales JP, Heil EL, Tata AL, Claeys KC, et al. Vancomycin pharmacokinetics in obese patients with sepsis or septic shock. Pharmacotherapy. 2020;40(3):211-20.

737. Klamerus KJ, Maloney K, Rudolph RL, Sisenwine SF, Jusko WJ, Chiang ST. Introduction of a composite parameter to the pharmacokinetics of venlafaxine and its active O-desmethyl metabolite. J Clin Pharmacol. 1992;32:716-24.

738. Lüscher TF, Noll G, Sturmer T, Huser B, Wenk M. Calcium gluconate in severe verapamil intoxication. N Engl J Med. 1994;330:718-20.

739. Schwab M, Oetzel C, Jägle C, Mörike K, Gleiter CH, Eichelbaum M. Using generic names is important to prevent iatrogenic drug overdose. A case report [Abstract]. Naunyn-Schmiedeberg's Arch Pharmacol. 2001;363(Suppl.):R132.

740. Malabanan A, Veronikis IE, Holick MF. Redefining vitamin D insufficiency. Lancet. 1998;351:805-6.

741. Heaney RP. Assessing vitamin D status. Curr Opin Clin Nutr Metab Care. 2011;14:440-4.

742. Mata-Granados JM, Luque de Castro MD, Quesada Gomez JM. Inappropriate serum levels of retinol, alpha-tocopherol, 25 hydroxyvitamin D3 and 24,25 dihydroxyvitamin D3 levels in healthy Spanish adults: simultaneous assessment by HPLC. Clin Biochem. 2008;41:676-80.

743. Pazaitou-Panayiotou K, Papapetrou PD, Chrisoulidou A, Konstantinidou S, Doumala E, Georgiou E, et al. Height, whole body surface area, gender, working outdoors, and sunbathing in previous summer are important determinants of serum 25-hydroxyvitamin D levels. Exp Clin Endocrinol Diabetes. 2012;120:14-22.

744. Potoski BA, Brown J. The safety of voriconazole. Clin Infect Dis. 2002;35:1273-5.

745. Tan K, Brayshaw N, Tomaszewski K, Troke P, Wood N. Investigation of the potential relationships between plasma voriconazole concentrations and visual adverse events or liver function test abnormalities. J Clin Pharmacol. 2006;46:235-43.

746. White RH, Zhou H, Romano P, Mungall D. Changes in plasma warfarin levels and variations in steady-state prothrombin times. Clin Pharmacol Ther. 1995;58:588-93.

747. Xu Z-X, Naadimuthu A, Lockwood G, Berger B, Maier G, Dukivic D. Pharmacokinetic/pharmacodynamic studies of zanoterone [Abstract]. Pharm Res. 1994;11:S-334.

748. Fletcher CV, Balfour HH, Jr. Variability in zidovudine serum concentrations. Pharmacotherapy. 1996;16:1154-8.

749. Fletcher CV, Acosta EP, Henry K, Page LM, Gross CR, Kawle SP, et al. Concentration-controlled zidovudine therapy. Clin Pharmacol Ther. 1998;64:331-8.

750. Hoetelmans RM, Burger DM, Meenhorst PL, Beijnen JH. Pharmacokinetic individualisation of zidovudine therapy. Current state of pharmacokinetic-pharmacodynamic relationships. Clin Pharmacokinet. 1996;30:314-27.

751. Chen T, Berenson J, Vescio R, Swift R, Gilchick A, Goodin S, et al. Pharmacokinetics and pharmacodynamics of zoledronic acid in cancer patients with bone metastases. J Clin Pharmacol. 2002;42:1228-36.

752. Skerjanec A, Berenson J, Hsu C, Major P, Miller WH, Jr., Ravera C, et al. The pharmacokinetics and pharmacodynamics of zoledronic acid in cancer patients with varying degrees of renal function. J Clin Pharmacol. 2003;43:154-62.

753. De Luca A., Lamura L, Gallo M, Daniele G, D'Alessio A, Giordano P, et al. Pharmacokinetic evaluation of zoledronic acid. Expert Opin Drug Metab Toxicol. 2011;7:911-8.

754. Debailleul G, Khalil FA, Lheureux P. HPLC quantification of zolpidem and prothipendyl in a voluntary intoxication. J Anal Toxicol. 1991;15(1):35-7.

755. Garnier R, Guerault E, Muzard D, Azoyan P, Chaumet-Riffaud AE, Efthymiou ML. Acute zolpidem poisoning--analysis of 344 cases. J Toxicol Clin Toxicol. 1994;32:391-404.

756. Winek CL, Wahba WW, Janssen JK, Rozin L, Rafizadeh V. Acute overdose of zolpidem. Forensic Sci Int. 1996;78:165-8.

757. Kochak GM, Page JG, Buchanan RA, Peters R, Padgett CS. Steady-state pharmacokinetics of zonisamide, an antiepileptic agent for treatment of refractory complex partial seizures. J Clin Pharmacol. 1998;38:166-71.

758. Mimaki T. Clinical pharmacology and therapeutic drug monitoring of zonisamide. Ther Drug Monit. 1998;20:593-7.

759. Schulz M, Schmoldt A. Therapeutic and toxic blood concentrations of more than 800 drugs and other xenobiotics. Pharmazie 2003;58(7):447-74.

760. Bircher J. Verbesserte Dosierung von Medikamenten durch Messung ihrer Plasmakonzentrationen [in German]. Ther Umsch. 1977;34:830-4.

761. Bircher J, Sommer W. Klinisch-pharmakologische Datensammlung [in German]. 2nd ed., Stuttgart: WVG; 1999.

762. Brosen K. Drug-metabolizing enzymes and therapeutic drug monitoring in psychiatry. Ther Drug Monit. 1996;18:393-6.

763. Caccia S, Garattini S. Formation of active metabolites of psychotropic drugs. An updated review of their significance. Clin Pharmacokinet. 1990;18:434-59.

764. Deom A. L'intoxication d'origine inconnue, l'apport du laboratoire pour le clinicien [in French]. Ther Umsch. 1986;43:259-68.

765. Deom A. Valeurs usuelles des taus sanguins, urinaires et autres lors de traitements ou lors d'intoxications chez l'homme [in French]. Ther Umsch. 1986;43:261-8.

766. Dinovo EC, Gottschalk LA, McGuire FL, Birch H, Heiser JF. Analysis of results of toxicological examinations performed by coroners' or medical examiners' laboratories in 2000 drug-involved deaths in nine major U.S. cities. Clin Chem. 1976;22:847-50.

767. Drayer DE. Pharmacologically active drug metabolites: therapeutic and toxic activities, plasma and urine data in man, accumulation in renal failure. Clin Pharmacokinet. 1976;1:426-43.

768. Drayer DE. Problems in therapeutic drug monitoring: the dilemma of enantiomeric drugs in man. Ther Drug Monit. 1988;10:1-7.

769. Gottschalk LA, Cravey RH. Toxicological and pathological studies on psychoactive drug-involved deaths. Davis: Biomedical Publishing; 1980.

770. Jack DB. Handbook of clinical pharmacokinetic data. Basingstoke: Macmillan; 1992.

771. Moffat AC, Jackson JV, Moss MS, Widdop B: Clarke's isolation and identification of drugs in pharmaceuticals, body fluids, and post-mortem material. 2nd ed. London: Pharmaceutical Press; 1986.

772. Ochs HR, Gugler R. Drug Monitoring in der Intensivmedizin [in German]. Internist (Berl). 1984;25:336-40.

773. Oellerich M, Sybrecht GW, Klein H. Therapieüberwachung durch Bestimmung von Plasmakonzentrationen im Serum [in German]. Internist (Berl). 1982;23:174-81.

774. Paterson SC. Drug levels found in cases of fatal self-poisoning. Forensic Sci Int. 1985;27:129-33.

775. Pentz B, Strubelt O, Gehlhoff C. Therapeutische, toxische und letale Arzneimittelkonzentrationen im menschlichen Plasma [in German]. Dt Ärztebl. 1979;43:2815-20.

776. Roots I. Wann ist die Bestimmung von Arzneimittelkonzentrationen im Plasma nützlich und notwendig? [in German]. Internist (Berl). 1986;27:40-52.

777. Rosenkranz B, Frölich JC. Plasmakonzentrationen von Arzneimitteln: Wann messen, wie interpretieren? [in German]. Dt Ärztebl. 1985;82:B-1769-78.

778. Spector R, Park GD, Johnson GF, Vesell ES. Therapeutic drug monitoring. Clin Pharmacol Ther. 1988;43:345-53.

779. Stead AH, Moffat AC. A collection of therapeutic, toxic and fatal blood drug concentrations in man. Hum Toxicol. 1983;2(3):437-64.

780. Sutherland JJ, Morrison RD, McNaughton CD, Daly TM, Milne SB, Daniels JS, et al. Assessment of patient medication adherence, medical record accuracy, and medication blood concentrations for prescription and over-the-counter medications (Supplementary Online Content eTable 2). JAMA Netw Open. 2018;1(7):e184196.

781. Krpo M, Luytkis HC, Haneborg AM, Høiseth G. A fatal blood concentration of 5-APB. Forensic Sci Int. 2018;291:e1-3.

782. Ambrosy AP, Butler J, Ahmed A, Vaduganathan M, van Veldhuisen DJ, Colucci WS, et al. The use of digoxin in patients with worsening heart failure: reconsidering an old drug to reduce hospital admissions. J Am Coll Cardiol. 2014;63(18):1823-32.

783. Bavendiek U, Aguirre Davila L, Koch A, Bauersachs J. Assumption versus evidence: the case of digoxin in atrial fibrillation and heart failure. Eur Heart J. 2017;38(27):2095-9.

784. Gheorghiade M, Patel K, Filippatos, G, Anker SD, Vn Veldhuisen DJ, Cleland JG, et al. Effect of oral digoxin in high-risk heart failure patients: a pre-specified subgroup analysis of the DIG trial. Eur J Heart Fail. 2013;15(5):551-9.

785. Adams KF Jr, Ghali JK, Herbert Patterson J, Stough WG, Butler J, Bauman JL, et al. A perspective on re-evaulating digoxin’s role in the current management of patients with chronic systolic heart failure: targeting serum concentration to reduce hospitalization and improve safety profile. Eur J Heart Fail. 2014;16(5):483-93.

786. Rathore SS, Curtis JP, Wang Y, Bristow MR, Krumholz HM. Association of serum digoxin concentration and outcomes in patients with heart failure. JAMA. 2003;289(7):871-8.

787. Morrison A, Stauffer ME, Kaufman AS. Defining medication adherence in individual patients. Patient Prefer Adherence. 2015;9:893-7.

788. Arzneimittelkommission der deutschen Ärzteschaft. Cyanid-Intoxikation nach oraler Amygdalin-Behandlung [in German]. Dtsch Ärztebl. 2014;111(50):A 2240-1.

789. Beamer WC, Shealy RM, Prough DS: Acute cyanide poisoning from laetrile ingestion. Ann Emerg Med. 1983;12:449-51.

790. Braico KT, Humbert JR, Terplan KL, Lehotay JM. Laetrile intoxication. Report of a fatal case. N Engl J Med. 1979;300:238-40.

791. O'Brien B, Quigg C, Leong T. Severe cyanide toxicity from 'vitamin supplements'. Eur J Emerg Med. 2005;12:257-8.

792. Sadoff L, Fuchs K, Hollander J. Rapid death associated with laetrile ingestion. JAMA. 1978;239:1532.

793. Smith FP, Butler TP, Cohan S, Schein PS. Laetrile toxicity: a report of two cases. JAMA. 1977;238:1361.

794. Bounameaux H, Camm AJ. Edoxaban: an update on the new oral direct factor Xa inhibitor. Drugs. 2014;74(11):1209-31.

795. Frost C, Nepal S, Wang J, Schuster A, Byon W, Boyd RA, et al. Safety, pharmacokinetics and pharmacodynamics of multiple oral doses of apixaban, a factor Xa inhibitor, in healthy subjects. Br J Clin Pharmacol. 2013;76(5):776-86.

796. Kitchen S, Gray E, Mackie I, Baglin T, Makris M; BCSH committee. Measurement of non-coumarin anticoagulants and their effects on tests of Haemostasis: Guidance from the British Committee for Standards in Haematology. Br J Haematol. 2014;166(6):830-41.

797. Steffel J, Verhamme P, Potpara TS, Albaladejo P, Antz M, Desteghe L, et al.; ESC Scientific Document Group. The 2018 European Heart Rhythm Association Practical Guide on the use of non-vitamin K antagonist oral anticoagulants in patients with atrial fibrillation. Eur Heart J. 2018;39(16):1330-93.

798. Skov L, Johansen SS, Linnet K. Postmortem femoral blood concentrations of aripiprazole, chlorprothixene, and quetiapine. J Anal Toxicol. 2015;39(1):41-4.

799. Meissner D. Arsen. In: Gressner AM, Arndt T (eds.): Lexikon der Medizinischen Laboratoriumsdiagnostik [in German]. 2. Auflage, Springer, 2013, 122-3.

800. Bässler K-H, Golly I, Loew D, Pietrik K (eds.). Vitamin-Lexikon [in German]. 2nd ed. 1997. Gustav Fischer/Govi, Stuttgart/Frankfurt.

801. Matta MK, Zusterzeel R, Pilli NR, Patel V, Volpe DA, Florian J, et al. Effect of sunscreen application under maximal use conditions on plasma concentration of sunscreen active ingredients: a randomized clinical trial. JAMA. 2019;321(21):2082-91.

802. Kuramoto K, Ichikawa S, Hirai A, Kanada S, Nakachi T, Ogihara T. Azelnidipine and amlodipine: a comparison of their pharmacokinetics and effects on ambulatory blood pressure. Hypertens Res. 2003;26(3):201-8.

803. Wellington K, Scott LJ. Azelnidipine. Drugs. 2013;63(23):2613-21.

804. Scholer A, Wernli C, Krebs D, Faffa G, Grienenberger D. Poisoning with unknown substance, “Munchhausen by proxy”? Toxichem Krimtech. 2013;80(1):38-42.

805. Schroeder C. Problematik, Klinik und Beispiele der Spurenelementvergiftung - Chrom [in German]. Toxichem Krimtech. 2016;83(2):79-81.

806. Hangartner S, Dussy F, Wyler D, Briellmann T. Einladung zum Suizid mit Chloroquin [in German]. Toxichem Krimtech. 2013;80(1):31-7.

807. Ebert K, Maurice E, Lukačin R, Fleischhaker C, Schulz E, Ebert D, et al. Serum and saliva concentrations of venlafaxine, O-desmethylvenlafaxine, quetiapine, and citalopram in psychiatric patients. Ther Drug Monit. 2018;40(3):351-5.

808. Haase D, König H. Serotonin-Syndrom einer Borderlinepatientin nach Suizidversuch mit Citalopram [in German]. Toxichem Krimtech. 2013;80(1):18-30.

809. Wohkittel C, Gerlach M, Taurines R, Wewetzer C, Unterecker S, Burger R, et al. Relationship between clozapine dose, serum concentration, and clinical outcome in children and adolescents in clinical practice. J Neural Transm. 2016;123(8):1021-31.

810. Arnestad M, Eldor KB, Stray-Pedersen A, Bachs L, Karinen R. Suicide due to cyclizine overdose. J Anal Toxicol. 2014;38(2):110-2.

811. Breimer DD, Winten MA. Pharmacokinetics and relative bioavailability of cyclobarbital calcium in man after oral administration. Eur J Clin Pharmacol. 1976;9:443-50.

812. Torimitsu S, Yajima D, Abe H, Kubo Y, Nagasawa S, Iwase H. Homicide–suicide by oral administration of cyclobarbital. Forensic Toxicol. 2014;32(1):180-5.

813. Fogarty MF, Papsun DM, Logan BK. Analysis of fentanyl and 18 novel fentanyl analogs and metabolites by LC-MS-MS, and report of fatalities associated with methoxyacetylfentanyl and cyclopropylfentanyl. J Anal Toxicol. 2018;42(9):592-604.

814. European Monitoring Centre for Drugs and Drug Addiction. Cyclopropylfentanyl. Report on the risk assessment of N-phenylN-[1-(2-phenylethyl)piperidin-4-yl] cyclopropanecarboxamide in the framework of the Council Decision on new psychoactive substances. https://www.ofdt.fr/BDD/publications/docs/RiskAssessmentCyclopropylfentanyl.pdf. Accessed 25 Feb 2020.

815. Maher S, Elliott SP, George S. The analytical challenges of cyclopropylfentanyl and crotonylfentanyl: an approach for toxicological analysis. Drug Test Anal. 2018;10:1483-7.

816. Müller D, Neurath H, Neukamm MA, Wilde M, Despicht C, Blaschke S, Grapp M. New synthetic opioid cyclopropylfentanyl together with other novel synthetic opioids in respiratory insufficient comatose patients detected by toxicological analysis. Clin Toxicol. 2019;57(9):806-12.

817. Blech S, Ebner T, Ludwig-Schwellinger E, Stangier J, Roth W. The metabolism and disposition of the oral direct thrombin inhibitor, dabigatran, in human. Drug Metab Dispos. 2008;36(2):386-99.

818. Legrand M, Mateo J, Aribaud A, Ginisty S, Eftekhari P, Huy PT, et al. The use of dabigatran in elderly patients. Arch Intern Med. 2011;171(14):1285-6.

819. Pernod G, Albaladejo P, Godier A, Samama CM, Susen S, Gruel Y, et al.; Working Group on Perioperative Haemostasis. Management of major bleeding complications and emergency surgery in patients on long-term treatment with direct oral anticoagulants, thrombin or factor-Xa inhibitors: proposals of the working group on perioperative haemostasis (GIHP) – March 2013. Arch Cardiovasc Dis. 2013;106(6-7):382-93.

820. Reilly PA, Lehr T, Haertter S, Connolly SJ, Yusuf S, Eikelboom JW, et al.; RE-LY Investigators. The effect of dabigatran plasma concentrations and patient characteristics on the frequency of ischemic stroke and major bleeding in atrial fibrillation patients: the RE-LY Trial (Randomized Evaluation of Long-Term Anticoagulation Therapy). J Am Coll Cardiol. 2014;63(4):321-8.

821. D'Avolio A, Pensi D, Baietto L, Pacini G, Di Perri G, De Rosa FG. Daptomycin pharmacokinetics and pharmacodynamics in septic and critically ill patients. Drugs. 2016;76(12):1161-74.

822. Bhavnani SM, Rubino CM, Ambrose PG, Drusano GL. Daptomycin exposure and the probability of elevations in the creatine phosphokinase level: data from a randomized trial of patients with bacteremia and endocarditis. Clin Infect Dis. 2010;50(12):1568–74.

823. Estes KS, Derendorf H. Comparison of the pharmacokinetic properties of vancomycin, linezolid, tigecyclin, and daptomycin. Eur J Med Res. 2010;15(12):533-43.

824. Galar A, Muñoz P, Valerio M, Cercenado E, García-González X, Burillo A, et al. Current use of daptomycin and systematic therapeutic drug monitoring: clinical experience in a tertiary care institution. Int J Antimicrob Agents. 2019;53(1):40-8.

825. Wong G, Sime FB, Lipman J, Roberts JA. How do we use therapeutic drug monitoring to improve outcomes from severe infections in critically ill patients? BMC Infect Dis. 2014;14:288.

826. Kim SW, Choe S, Kim DJ, Zang DY, Lee DH. Pharmacokinetics of doripenem in healthy Koreans and Monte Carlo simulations to explore optimal dosage regimens in patients with normal and enhanced renal function. Ther Drug Monit. 2018;40(4):425-34.

827. Eckes L, Tsokos, M, Herre S, Gapert R, Hartwig S. Post-mortem evidence of doxylamine in toxicological analyses. Sci Justice. 2014;54(1):61-5.

828. EMA. Dronedarone Summary of Product Characteristics. https://www.ema.europa.eu/documents/product-information/multaq-epar-product-information_en.pdf. Accessed 20 Feb 2020.

829. Isenmann E, Ambrosio G, Joseph JF, Mazzarino M, de la Torre X, Zimmer P, et al. Ecdysteroids as non-conventional anabolic agent: performance enhancement by ecdysterone supplementation in humans. Arch Toxicol. 2019;93(7):1807-16.

830. Aisenberg J, Chatterjee-Murphy P, Friedman Flack K, Weitz JI, Ruff CT, Nordio F, et al. Gastrointestinal bleeding with edoxaban versus warfarin: results from the ENGAGE AF-TIMI 48 trial (effective anticoagulation with factor Xa next generation in atrial fibrillation-thrombolysis in myocardial infarction). Circ Cardiovasc Qual Outcomes. 2018;11(5):e003998.

831. Gibbons J, de Vries M, Krauwinkel W, Ohtsu Y, Noukens J, van der Walt JS, et al. Pharmacokinetic drug interaction studies with enzalutamide. Clin Pharmacokinet. 2015;54(10):1067-9.

832. Gibbons J Ouatas T, Krauwinkel W, Ohtsu Y, van der Walt JS, Beddo V, et al. Clinical pharmacokinetic studies of enzalutamide. Clin Pharmacokinet. 2015;54(10):1043-55.

833. Huttner A, Harbarth S, Hope WW, Lipman J, Roberts JA. Therapeutic drug monitoring of the β-lactam antibiotics: what is the evidence and which patients should we be using it for? J Antimicrob Chemother. 2015;70(12):3178-83.

834. Wittau M, Paschke S, Kurlbaum M, Scheele J, Ly NS, Hemper E, et al. Population pharmacokinetics and target attainment of ertapenem in plasma and tissue assessed via microdialysis in morbidly obese patients after laparoscopic visceral surgery. Antimicrob Agents Chemother. 2016;61(1): e00952-16.

835. Høiseth G, Tuv SS, Karinen R. Blood concentrations of new designer benzodiazepines in forensic cases. Forensic Sci Int. 2016;268:35-38.

836. Huppertz LM, Moosmann B, Auwärter V. Flubromazolam - Basic pharmacokinetic evaluation of a highly potent designer benzodiazepine. Drug Test Anal. 2018;10(1):206-11.

837. Koch K, Auwärter V, Hermanns-Clausen, Wilde M, Neukamm MA. Mixed intoxication by the synthetic opioid U-47700 and the benzodiazepine flubromazepam with lethal outcome: pharmacokinetic data. Drug Test Anal. 2018. Doi: 10.1002/dta.2391.

838. Moosmann B, Huppertz LM, Hutter M, Buchwald A, Ferlaino S, Auwärter V. Detection and identification of the designer benzodiazepine flubromazepam and preliminary data on its metabolism and pharmacokinetics. J Mass Spectrom. 2013;48(11):1150-9.

839. Valli A, Lonati D, Locatelli CA, Buscaglia E, Tuccio MD, Papa P. Analytically diagnosed intoxication by 2-methoxphenidine and flubromazepam mimicking an ischemic cerebral disease. Clin Toxicol (Phila). 2017;55(6):611-2

840. Papa P, Valli A, Di Tuccio M, Frison G, Zancanaro F, Buscaglia E, Locatelli CA. Analytically confirmed intoxication by 4-fluoromethylphenidate, an analog of methylphenidate. J Anal Toxicol. 2019;43(5):e1-e7.

841. Cantrell FL, Mena O, Gary RD, McIntyre IM. An acute gabapentin fatality: a case report with postmortem concentrations. Int J Legal Med. 2015;129(4):771–5.

842. Evoy KE, Morrison MD, Saklad SR. Abuse and misuse of pregabalin and gabapentin. Drugs. 2017;77(4):403-26.

843. Andresen-Streichert H, Jensen P, Kietzerow J, Schrot M, Wilke N, Vettorazzi E, et al. Endogenous gamma-hydroxybutyric acid (GHB) concentrations in post-mortem specimens and further recommendation for interpretative cut-offs. Int J Legal Med. 2015;129(1):57-68.

844. Busardò FP, Jones AW. Interpreting γ-hydroxybutyrate concentrations for clinical and forensic purposes. Clin Toxicol (Phila). 2018;11:1-15.

845. Castro AL, Dias M, Reis F, Teixeira HM. Gamma-hydroxybutyric acid endogenous production and post-mortem behaviour - the importance of different biological matrices, cut-off reference values, sample collection and storage conditions. J Forensic Leg Med. 2014;27:17-24.

846. Castro AL, Tarelho S, Dias M, Reis F, Teixeira HM. Comparison of endogenous GHB concentrations in blood and hair in death cases with emphasis on the post mortem interval. Int J Legal Med. 2016;130(4):959-65.

847. Kegler R, Lehmann C, Rentsch D, Blömker M, Büttner A. Bestimmung von endogenen GHB-Konzentrationen in Haaren [in German]. Toxichem Krimtech. 2018;85(3):110-6.

848. Costedoat-Chalumeau N, Amoura Z, Hulot JS, Hammoud HA, Aymard G, Cacoub P, et al. Low blood concentration of hydroxychloroquine is a marker for and predictor of disease exacerbations in patients with systemic lupus erythematosus. Arthritis Rheum. 2006;54:3284-90.

849. Costedoat-Chalumeau N, Galicier L, Aumaître O, Francès C, Le Guern V, Lioté F, et al. Hydroxychloroquine in systemic lupus erythematosus: results of a French multicentre controlled trial (PLUS Study). Ann Rheum Dis. 2013;72:1786-92.

850. Durcan L, Clarke WA, Magder LS, Petri M. Hydroxychloroquine blood levels in systemic lupus erythematosus: clarifying dosing controversies and improving adherence. J Rheumatol. 2015;42:2092-7.

851. Yeon LJ, Lee J, Ki KS, Hyeon JJ, Su PK, Park SH. Factors related to blood hydroxychloroquine concentration in patients with systemic lupus erythematosus. Arthritis Care Res (Hoboken). 2017;69:536-42.

852. Costedoat-Chaloumeau N, Houssiau F, Izmirly P, Le Guern V, Navarra S, Jolly M, et al. A prospective international study on adherence to treatment in 305 patients with flaring SLE: Assessment by drug levels and self-administered questionnaires. Clin Pharmacol Ther. 2018;103(6):1074-82.

853. Kölle EU, Vollmer KO. Pharmacokinetics of isoxicam following intravenous, intramuscular, oral and rectal administration in healthy volunteers. Br J Clin Pharmacol. 1986;22(Suppl. 2):135S-41S.

854. Chen N, Lau H, Kong L, Kumar G, Zeldis JB, Knight R, et al. Pharmacokinetics of lenalidomide in subjects with various degrees of renal impairment and in subjects on hemodialysis. J Clin Pharmacol. 2007;47(12):1466-75.

855. Chen N, Zhou S, Palmisano M. Clinical pharmacokinetics and pharmacodynamics of lenalidomide. Clin Pharmacokinet. 2017;56(2):139-52.

856. Kobayashi T, Miura M, Niioka T, Abumiya M, Ito F, Kobayashi I, et al. Phase II clinical trial of lenalidomide and dexamethasone therapy in Japanese elderly patients with newly diagnosed multiple myeloma to determine optimal plasma concentration of lenalidomide. Ther Drug Monit. 2018;40(3):301-9.

857. Eggleston W, Nacca N, Marraffa JM. Loperamide toxicokinetics: serum concentrations in the overdose setting. Clin Toxicol. 2015;53(5):495-6.

858. Eggleston W, Clark KH, Marraffa JM. Loperamide abuse associated with cardiac dysrhythmia and death. Ann Emerg Med. 2017;69(1):83-6.

859. Schulz M, Braun R. Zur Pharmakologie und Toxikologie von Loperamid [Pharmacology and toxicology of loperamide] [in German]. Pharm Ztg. 1989;134(40):2426-7.

860. Spinner HL, Lonardo NW, Mulamalla R, Stehlik J. Ventricular tachycardia associated with high-dose chronic loperamide use. Pharmacotherapy. 2015;35(2):234-8.

861. Wang Z, Lee B, Pearce D, Qian S, Wang Y, Zhang Q, Chow MS. Meclizine metabolism and pharmacokinetics: formulation on its absorption. J Clin Pharmacol. 2012;52(9):1343-9.

862. Christensson BA, Nilsson-Ehle I, Hutchison M, Haworth SJ, Oqvist B, Norrby SR. Pharmacokinetics of meropenem in subjects with various degrees of renal impairment. Antimicrob Agents Chemother. 1992;36:1532-7.

863. Graham GG, Punt J, Arora M, Day RO, Doogue MP, Duong JK, et al. Clinical pharmacokinetics of metformin. Clin Pharmacokinet. 2011;50(2):81-98.

864. Scheen AJ. Clinical pharmacokinetics of metformin. Clin Pharmacokinet. 1996;30(5):359-71.

865. Stevens A, Hamel JF, Toure A, Hadjadj S, Boels D. Metformin overdose: A serious iatrogenic complication – Western France Poison Control Centre Data Analysis. Basic Clin Pharmacol Toxicol. 2019;125(5):466-73.

866. Klampfl K, Quattländer A, Burger R, Pfuhlmann B, Warnke A, Gerlach M. Case report: intoxication with high dose of long-acting methylphenidate (Concerta®) in a suicidal 14-year-old girl. Atten Defic Hyperact Disord. 2010;2(4):221-4.

867. Fatteh A, Dudley JB. Fatal poisoning involving methapyrilene. JAMA. 1972;219(6):756-7.

868. Sauer C, Hoffmann K, Schimmel U, Peters FT. Acute poisoning involving the pyrrolidinophenone-type designer drug 4'-methyl-alpha-pyrrolidinohexanophenone (MPHP). Forensic Sci Int. 2011;208(1-3):e20-5.

869. Abshagen U, Betzien G, Kaufmann B, Endele G. Pharmacokinetics of metipranolol in normal man. Eur J Clin Pharmacol. 1982;21(4):293-301.

870. Saedder EA, Thomsen AH, HasselstrØm JB, Jornil JR. Heart insufficiency after combination of verapamil and metoprolol: A fatal case report and literature review: Clin Case Rep. 2019;7(11):2042-8.

871. Holler JM, Vorce SP, McDonough-Bender PC, Magluilo J Jr, Solomon CJ, Levine B. A drug toxicity death involving propylhexedrine and mitragynine. J Anal Toxicol. 2011;35(1):54-9.

872. Karinen R, Fosen JT, Rogde S, Vindenes V. An accidental poisoning with mitragynine. Forensic Sci Int. 2014;245:e29-32.

873. Lu S, Tran BN, Nelsen JL, Aldous KM. Quantitative analysis of mitragynine in human urine by high performance liquid chromatography-tandem mass spectrometry. J Chromatogr B Analyt Technol Biomed Life Sci. 2009;877(24):2499-505.

874. McIntyre IM, Trochta A, Stolberg S, Campman SC. Mitragynine 'Kratom' related fatality: a case report with postmortem concentrations. J Anal Toxicol. 2015;39(2):152-5.

875. Neerman MF, Frost RE, Deking J. A drug fatality involving Kratom. J Forensic Sci. 2013;58 (Suppl. 1):S278-9.

876. Trakulsrichai S, Sathirakul K, Auparakkitanon S, Krongvorakul J, Sueajai J, Noumjad N, et al. Pharmacokinetics of mitragynine in man. Drug Des Devel Ther. 2015;9:2421-9.

877. Wright TH. Suspected driving under the influence case involving mitragynine. J Anal Toxicol. 2018;42(7):e65-8.

878. Nosseir NS, Michels G, Pfister R, Adam R, Wiesen MHJ, Müller, C. Therapeutisches Drug Monitoring (TDM) von Antiinfektiva in der Intensivmedizin [Therapeutic drug monitoring of antiinfectives in intensive care medicine] [in German]. Dtsch Med Wochenschr. 2014;139(38):1889-94.

879. Reis M, Aberg-Wistedt A, Agren H, Höglund P, Akerblad AC, Bengtsson F. Serum disposition of sertraline, N-desmethylsertraline and paroxetine: a pharmacokinetic evaluation of repeated drug concentration measurements during 6 months of treatment for major depression. Hum Psychopharmacol. 2004;19(5):283-91.

880. Allibe N, Richeval C, Phanithavong M, Faure A, Allorge D, Paysant F, et al. Fatality involving ocfentanil documented by identification of metabolites. Drug Test Anal. 2018;10(6):995-1000.

881. Casati S, Minoli M, Angeli I, Ravelli A, Crudele GDL, Orioli M. An ocfentanil-related death case: UHPLC-MS/MS analysis of the drug. Drug Test Anal. 2019;11(1):173-7.

882. Coopman V, Cordonnier J, De Leeuw M, Cirimele V. Ocfentanil overdose fatality in the recreational drug scene. Forensic Sci Int. 2016;266:469-73.

883. Dussy FE, Hangartner S, Hamberg C, Berchtold C, Scherer U, Schlotterbeck G, et al. An acute ocfentanil fatality: A case report with postmortem concentrations. J Anal Toxicol. 2016;40(9):761-6.

884. Cleland JG, Teerlink JR, Senior R, Nifontov EM, Mc Murray JJ, Lang CC, et al. The effects of the cardiac myosin activator, omecamtiv mecarbil, on cardiac function in systolic heart failure: a double-blind, placebo-controlled, crossover, dose-ranging phase 2 trial. Lancet. 2011;378(9792):676-83.

885. Palaparthy R, Banfield C, Alvarez P, Yan L, Smith B, Johnson J, et al. Relative bioavailability, food effect, and safety of the single-dose pharmacokinetics of omecamtiv mecarbil following administration of different modified-release formulations in healthy subjects. Int J Clin Pharmacol Ther. 2016;54(3):217-27.

886. Teerlink JR, Felker GM, McMurray JJV, Ponikowski P, Metra M, Filippatos GS, et al.; ATOMIC-AHF Investigators. Acute treatment with omecamtiv mecarbil to increase contractility in acute heart failure: The ATOMIC-AHF study. J Am Coll Cardiol. 2016;67(12):1444-55.

887. Teerlink JR, Felker GM, McMurray JJ, Solomon SD, Adams KF Jr, Cleland JG, et al.; COSMIC-HF Investigators. Chronic oral study of myosin activation to increase contractility in heart failure (COSMIC-HF): a phase 2, pharmacokinetic, randomised, placebo-controlled trial. Lancet. 2016;388(10062):2895-903.

888. Vu T, Ma P, Xiao JJ, Wang YM, Malik FI, Chow AT. Population pharmacokinetic-pharmacodynamic modeling of omecamtiv mecarbil, a cardiac

myosin activator, in healthy volunteers and patients with stable heart failure. J Clin Pharmacol. 2015;55(11):1236-47.

889. Bentur Y, Shoshani O, Tabak A, Bin-Nun A, Ramon Y, Ulman Y, et al. Prolonged elimination half-life of phenol after dermal exposure. J Toxicol Clin Toxicol. 1998;36(7):707-11.

890. Hengstmann JH, Goronzy J. Pharmacokinetics of 3H-phenylephrine in man. Eur J Clin Pharmacol. 1982;21(4):335-41.

891. Janin A, Monnet J. Bioavailability of paracetamol, phenylephrine hydrochloride and guaifenesin in a fixed-combination syrup versus an oral reference product. J Int Med Res. 2014;42(2):347-59. Corrigendum in: J Int Med Res. 2014;42(4):1060-1.

892. Ptácek P, Klíma J, Macek J. Development and validation of a liquid chromatography-tandem mass spectrometry method for the determination of phenylephrine in human plasma and its application to a pharmacokinetic study. J Chromatogr B Analyt Technol Biomed Life Sci. 2007;858(1-2):263-8.

893. Budde K, Neumayer HH, Fritsche L, Sulowicz W, Stompôr T, Eckland D. The pharmacokinetics of pioglitazone in patients with impaired renal function. Br J Clin Pharmacol. 2003;55(4):368-74.

894. Jang SH, Colangelo PM, Gobburu JV. Exposure-response of posaconazole used for prophylaxis against invasive fungal infections: evaluating the need to adjust doses based on drug concentrations in plasma. Clin Pharmacol Ther. 2010;88(1):115–9.

895. Yi WM, Schoeppler KE, Jaeger J, Mueller SW, MacLaren R, Fish DN, Kiser TH. Voriconazole and posaconazole therapeutic drug monitoring: a retrospective study. Ann Clin Microbiol Antimicrob. 2017;16(1):60.

896. Greenblatt DJ, Shader RI. Prazepam, a precursor of desmethyldiazepam. Lancet. 1978;1(8066):729.

897. Berry D, Millington C. Analysis of pregabalin at the therapeutic concentrations in human plasma/serum by reversed-phase HPLC. Ther Drug Monit. 2005;27(4):451–6.

898. Eastwood JA, Davison E. Pregabalin concentrations in post-mortem blood – a two year study. Forensic Sci Int. 2016;266:197–201.

899. Lottner-Nau S, Övgüer B, Paul LD, Graw M, Sach H, Roider G. Abuse of pregabalin – results of the postmortem toxicology from 2010 to 2012. Toxichem Krimtech. 2013;80(Special Issue):339-42.

900. Klausz G, Róna K, Kristóf I, Töro K. Evaluation of a fatal propofol intoxication due to self administration. J Forensic Leg Med. 2009;16(5):287-9.

901. Levy RJ. Clinical effects and lethal and forensic aspects of propofol. J Forensic Sci. 2011;56(Suppl. 1):S142-7.

902. Mannocchi G, Napoleoni F, Napoletano S, Pantano F, Santoni M, Tittarelli R, Arbarello P. Fatal self administration of tramadol and propofol: a case report. J Forensic Leg Med. 2013;20(6):715-9.

903. Shafer A, Doze VA, Shafer SL, White PF. Pharmacokinetics and pharmacodynamics of propofol infusions during general anesthesia. Anesthesiology. 1988;69:348-56.

904. Wesson DR. Propylhexedrine. Drug Alcohol Depend. 1986;17(2-3):273-8.

905. Albantakis L, Egberts K, Burger R, Kulpok C, Mehler-Wex C, Taurines R, et al. Relationship between daily dose, serum concentration, and clinical response to quetiapine in children and adolescents with psychotic and mood disorders. Pharmacopsychiatry. 2017;50(6):248-55.

906. Klampfl K, Taurines R, Preuss A, Burger R, Rothenhöfer S, Wewetzer Ch, et al. Serum concentrations, therapeutic response and side effects in children and adolescents with impulsive-aggressive symptoms during risperidone therapy. Pharmacopsychiatry. 2010;43(2):58-65.

907. Schoretsanitis G, de Leon J, Haen E, Stegmann B, Hiemke C, Gründer G, et al. Pharmacokinetics of risperidone in different application forms – comparing long-acting injectable and oral formulations. Eur Neuropsychopharmacol. 2018;28(1):130-7.

908. Mueck W, Borris LC, Dahl OE, Haas S, Huisman MV, Kakkar AK, et al. Population pharmacokinetics and pharmacodynamics of once- and twice-daily rivaroxaban for the prevention of venous thromboembolism in patients undergoing total hip replacement. Thromb Haemost. 2008;100(3):453-61.

909. Ayalasomayajula S, Langenickel T, Pal P, Boggarapu S, Sunkara G. Erratum to: Clinical pharmacokinetics of sacubitril/valsartan (LCZ696): a novel angiotensin receptor-neprilysin inhibitor. Clin Pharmacokinet. 2018;57(1):105-23.

910. Anderson R, Hayesa J, Stephens JW. Pharmacokinetic, pharmacodynamic and clinical evaluation of saxagliptin in type 2 diabetes. Expert Opin Drug Metab Toxicol 2016;12(4):467-73.

911. Scheen AJ. Pharmacokinetics of dipeptidylpeptidase-4 inhibitors. Diabetes Obes Metab. 2010;12(8):648-58.

912. Su H, Boulton DW, Barros A Jr, Wang L, Cao K, Bonacorsi SJ Jr, Iyer RA, et al. Characterization of the in vitro and in vivo metabolism and disposition and cytochrome P450 inhibition/induction profile of saxagliptin in human. Drug Metab Dispos. 2012;40(7):1345-56.

913. Chae H, Lee JJ, Cha K, Her SH, Kim HY, Han E, et al. Measurement of teicoplanin concentration with liquid chromatography-tandem mass spectrometry method demonstrates the usefulness of therapeutic drug monitoring in hematologic patient populations. Ther Drug Monit. 2018;40(3):330-6.

914. Dziadosz M, Klintschar M, Teske J. Postmortem concentration distribution in fatal cases involving the synthetic opioid U-47700. Int J Legal Med. 2017;131:1555-6.

915. Lehmann S, Teifel D, Rothschild MA, Andresen-Streichert H. Tödliche Intoxikation mit dem Designer-Opioid U-47700 [Fatal intoxication with designer-opioid U-47700] [in German]. Toxichem Krimtech. 2018;85(1):36.

916. Mohr AL, Friscia M, Papsun D, Kacinko SL, Buzby D, Logan BK. Analysis of novel synthetic opioids U-47700, U-50488 and furanyl fentanyl by LC-MS/MS in postmortem casework. J Anal Toxicol. 2016;40(9):709-17.

917. Rambaran KA, Fleming SW, An J, Burkhart S, Furmaga J, Kleinschmidt KC, et al. U-47700: a clinical review of the literature. J Emerg Med. 2017;53(4):509-19.

918. Richeval C, Gaulier JM, Romeuf L, Allorge D, Gaillard Y. Case report: relevance of metabolite identification to detect new synthetic opioid intoxications illustrated by U-47700. Int J Legal Med. 2019;133(1):133-42.

919. Vo KT, van Wijk XMR, Wu AH, Lynch KL, Ho RY. Synthetic agents of the darknet, a case of U-47700 and phenazepam abuse. Clin Toxicol (Phila). 2017;55:71-2.

920. World Health Organization. U-47700. Critical Review Report. Agenda Item 4.1. Expert Committee on Drug Dependence Thirty-eighth Meeting Geneva, 14-18 November 2016. http://www.who.int/medicines/access/controlled-substances/4.1_U-47700_CritReview.pdf. Accessed 25 Feb 2020.

921. Rosario M, Dirks NL, Milch C, Parikh A, Bargfrede M, Wyant T, et al. A review of the clinical pharmacokinetics, pharmacodynamics, and immunogenicity of vedolizumab. Clin Pharmacokinet. 2017;56(11):1287-301.

922. Sakurai H, Kei M, Matsubara K, Yokouchi K, Hattori K, Ichihashi R, et al. Cardiogenic shock triggered by verapamil and atenolol; a case report of therapeutic experience with intravenous calcium. Jpn Curc J. 2000;64(11):893-6.

923. He YL. Clinical pharmacokinetics and pharmacodynamics of vildagliptin. Clin Pharmacokinet. 2012;51(3):147-62.

924. Henness S, Keam SJ. Vildagliptin. Drugs. 2006;66(15):1989-2001.

925. German Nutrition Society. New reference values for vitamin D. Ann Nutr Metab. 2012;60(4):241-6.

926. Holubek W, Stolbach A, Nurok S, Lopez O, Wetter A, Nelson L. A report of two deaths from massive ibuprofen ingestion. J Med Toxicol. 2007;3(2):52-5.

927. Uchida S, Shimada K, Misaka S, Imai H, Katoh Y, Inui N, et al. Benzbromarone pharmacokinetics and pharmacodynamics in different

cytochrome P450 2C9 genotypes. Drug Metab Pharmacokinet. 2010;25(6):605-10.

928. Ferber H, Vergin H, Hitzenberger G. Pharmacokinetics and biotransformation of benzbromarone in man. Eur J Clin Pharmacol. 1981;19(6):431-5.

929. Hochhaus G, Möllmann H. Pharmacokinetic/pharmacodynamic characteristics of the beta-2-agonists terbutaline, salbutamol and fenoterol. Int J Clin Pharmacol Ther Toxicol. 1992;30(9):342-62.

930. Svedmyr N. Fenoterol: a beta2-adrenergic agonist for use in asthma. Pharmacology, pharmacokinetics, clinical efficacy and adverse effects. Pharmacotherapy. 1985;5(3):109-26.

931. Janssen U, Walker S, Maier K, von Gaisberg U, Klotz U. Flumazenil disposition and elimination in cirrhosis. Clin Pharmacol Ther. 1989;46(3):317-23.

932. Roncari G, Timm U, Zell M, Zumbrunnen R, Weber W. Flumazenil kinetics in the elderly. Eur J Clin Pharmacol. 1993;45(6):585-7.

933. Roncari G, Ziegler WH, Guentert TW. Pharmacokinetics of the new benzodiazepine antagonist Ro 15-1788 in man following intravenous and oral administration. Br J Clin Pharmacol. 1986;22(4):421-8.

934. Klotz U. Drug interactions and clinical pharmacokinetics of flumazenil. Eur J Anaesthesiol. 1988;2(Suppl.):103-8.

935. Gunja N. The clinical and forensic toxicology of Z-drugs. J Med Toxicol 2013;9(2):155-62.

936. Gessler T, Ghofrani HA, Held M, Klose H, Leuchte H, Olschewski H, et al. The safety and pharmacokinetics of rapid iloprost aerosol delivery via the BREELIB nebulizer in pulmonary arterial hypertension. Pulm Circ. 2017;7(2):505-513.

937. Grant SM, Goa KL. Iloprost. A review of its pharmacodynamic and pharmacokinetic properties, and therapeutic potential in peripheral vascular disease, myocardial ischaemia and extracorporeal circulation procedures. Drugs. 1992;43(6):889-924.

938. Hess C, Unger M, Madea B, Stratmann B, Tschoepe D. Range of therapeutic metformin concentrations in clinical blood samples and comparison to a forensic case with death due to lactic acidosis. Forensic Sci Int. 2018;286:106-12.

939. Walz L, Jönsson AK, Ahlner J, Östgren CJ, Druid H. Metformin – Postmortem fatal and non-fatal reference concentrations in femoral blood and risk factors associated with fatal intoxications. Forensic Sci Int. 2019;303:109935.

940. Lehmann S, Thomas A, Schiwy-Bochat KH, Geyer H, Thevis M, Glenewinkel F, et al. Death after misuse of anabolic substances (clenbuterol, stanozolol and metandienone). Forensic Sci Int. 2019;303:109925.

941. Spiller HA, James KJ, Scholzen S, Borys DJ. A descriptive study of adverse events from clenbuterol misuse and abuse for weight loss and bodybuilding. Subst Abus. 2013;34(3):306-12.

942. Hoffman RJ, Hoffman RS, Freyberg CL, Poppenga RH, Nelson LS. Clenbuterol ingestion causing prolonged tachycardia, hypokalemia, and hypophosphatemia with confirmation by quantitative levels. J Toxicol Clin Toxicol. 2001;39(4):339-44.

943. Fabresse N, Grassin-Delyle S, Etting I, Alvarez JC. Detection and quantification of 12 anabolic steroids and analogs in human whole blood and 20 in hair using LC-HRMS/MS: application to real cases. Int J Legal Med. 2017;131(4):989-99.

944. Thomas L (ed.). Labor und Diagnose [in German]. TH-Books, Frankfurt/Main, 2012.

945. McIntyre IM, Trochta A, Gary RD, Malamatos M, Lucas JR. An acute acetyl fentanyl fatality: a case report with postmortem concentrations. J Anal Toxicol. 2015;39(6):490-4.

946. Cunningham SM, Haikal NA, Kraner JC. Fatal intoxication with acetyl fentanyl. J Forensic Sci. 2016;61(Suppl. 1):S276-80.

947. Takase I, Koizumi T, Fujimoto I, Yanai A, Fujimiya T. An autopsy case of acetyl fentanyl intoxication caused by insufflation of 'designer drugs'. Leg Med (Tokyo). 2016;21:38-44.

948. Park HD, Kim HK, Kim JW, Kim DW, Lee JH, Huh W, et al. Evaluation of the transfusion safety of blood products and determination of plasma concentrations of acitretin and etretinate in patients receiving transfusions.Transfusion. 2008;48(11):2395-400.

949. Pilkington T, Brogden RN. Acitretin a review of its pharmacology and therapeutic used drugs 1992;43(4):597-627.

950. Guerrieri D, Rapp E, Roman M, Thelander G, Kronstrand R. Acrylfentanyl: another new psychoactive drug with fatal consequences. Forensic Sci Int. 2017;277:e21-9.

951. Helander A, Bäckberg M, Signell P, Beck O. Intoxications involving acrylfentanyl and other novel designer fentanyls - results from the Swedish STRIDA project. Clin Toxicol (Phila). 2017;55(6):589-99.

952. Ikeda N, Umetsu K, Suzuki T, Gonmori K, Takahashi K. An infant fatality involving ajmaline. J Forensic Sci. 1988;33(2):558-61.

953. Yildiz M, Kocabay G Unreported cardiac arrhythmias in aluminium worker. J Forensic Leg Med. 2013;20(6):760-2.

954. Jenkins A, Thomson AH, Brown NM, Semple Y, Sluman C, MacGowan A, et al. Amikacin use and therapeutic drug monitoring in adults: do dose regimes and

drug exposrues affect either outcome or adverse events? A systematic review. J Antimicrob Chemother. 2016;71(10):2754-9.

955. Elliott S, Evans J. A 3-year review of new psychoactive substances in casework. Forensic Sci Int. 2014;243:55-60.

956. Bäckberg M, Beck O, Hultén P, Rosengren-Holmberg J, Helander A. Intoxications of the new psychoactive substance 5-(2-aminopropyl)indole (5-IT): a case series from the Swedish STRIDA project. Clin Toxicol (Phila). 2014;52(6):618-24.

957. Kronstrand R, Roman M, Dahlgren M, Thelander G, Wikström M, Druid H. A cluster of deaths involving 5-(2-aminopropyl)indole (5-IT). J Anal Toxicol. 2013;37(8):542-6.

958. Tracqui A, Mutter-Schmidt C, Kintz P, Berton C, Mangin P. Amisulpride poisoning: a report on two cases. Hum Exp Toxicol. 1995;14(3):294-8.

959. Lauwers LF, Roelants A, Rosseel PM, Heyndrickx B, Baute L. Oral antimony intoxications in man. Crit Care Med. 1990;18(3):324-6.

960. Gous T, Couchman L, Patel JP, Paradzai C, Arya R, Flanagan RJ. Measurement of the direct oral anticoagulants apixaban, dabigatran, edoxaban, and rivaroxaban in human plasma using turbulent flow liquid chromatography with high-resolution mass spectrometry. Ther Drug Monit. 2014;36(5):597-605.

961. Simons FE, Simons KJ. Clinical pharmacology of new histamine H_1 receptor antagonists. Clin Pharmacokinet. 1999;36(5):329-52.

962. Garside D, Ropero-Miller JD, Riemer EC. Postmortem tissue distribution of atomoxetine following fatal and nonfatal doses – three case reports. J Forensic Sci. 2006;51(1):179-82.

963. Cohan JA, Manning TJ, Lukash L, Long C, Ziminski KR, Conradi SE. Two fatalities resulting from Tessalon (benzonatate). Vet Hum Toxicol. 1986;28(6):543-4.

964. Hashiyada M, Usui K, Hayashizaki Y, Hosoya T, Igari Y, Sakai J, et al. Unexpectedly high blood concentration of bisoprolol after an incorrect prescription: A case report. Leg Med (Tokyo). 2013;15(2):103-5.

965. Harron DW, Goa KL, Langtry HD. Bopindolol. A review of its pharmacodynamic and pharmacokinetic properties and therapeutic efficacy. Drugs. 1991;41(1):130-49.

966. Wiergowski M, Aszyk J, Kaliszan M, Wilczewska K, Anand JS, Kot-Wasik A, et al. Identification of novel psychoactive substances 25B-NBOMe and 4-CMC in biological material using HPLC-Q-TOF-MS and their quantification in blood using UPLC-MS/MS in case of severe intoxications. J Chromatogr B Analyt Technol Biomed Life Sci. 2017;1041-1042:1-10.

967. Iwersen-Bergmann S, Lehmann S, Heinemann A, Schröder C, Müller A, Jungen H, et al. Mass poisoning with NPS: 2C-E and Bromo-DragonFly. Int J Legal Med. 2019;133(1):123-9.

968. Andreasen MF, Telving, R, Birkler RID, Schumacher, B, Johannsen M. A fatal poisoning involving Bromo-Dragonfly. Forensic Sci Int. 2009;183:91-6.

969. Berling I, Buckley NA, Mostafa A, Downes MA, Grice J, Medley G, et al. 2-Methyl-4-chlorophenoxyacetic acid and bromoxynil herbicide death. Clin Toxicol (Phila). 2015;53(5):486-8.

970. Sakai K, Saito K, Takada A, Hikiji W, Kikuchi Y, Fukunaga T. Deaths associated with brotizolam poisoning from a single drug overdose: Four reported cases. Am J Forensic Med Pathol. 2018;39(1):82-4.

971. Yazzie J, Kelly SC, Zumwalt RE, Kerrigan S. Fatal bupivacaine intoxication following unusual erotic practices. J Forensic Sci. 2004;49(2):351-3.

972. Walmsley LM, Brodie RR, Chasseaud LF. Determination of carboxybupranolol, the major metabolite of bupranolol, in human plasma by high-performance liquid chromatography. J Chromatogr. 1984;311(1):227-33.

973. Overberg A, Morton S, Wagner E, Froberg B. Toxicity of bupropion overdose compared with selective serotonin reuptake inhibitors. Pediatrics. 2019;144(2):e20183295.

974. Poklis J, Poklis A, Wolf C, Hathaway C, Arbefeville E, Chrostowski L, et al. Two fatal intoxications involving butyryl fentanyl. J Anal Toxicol. 2016;40(8):703-8.

975. Eichhorn L, Michaelis D, Kemmerer M, Jüttner B, Tetzlaff K. Carbon monoxide poisoning from waterpipe smoking: a retrospective cohort study. Clin Toxicol (Phila). 2018;56(4):264-72.

976. Paulsen JF, Villads KV, Sonne ME. Acute carbon monoxide poisoning after water pipe tobacco smoking. Ugeskr Laeger. 2016;178(49):V06160417.

977. Veen M. Carbon monoxide poisoning caused by water pipe smoking: A case series. J Emerg Med. 2016;51(3):e41-4.

978. von Rappard J, Schönenberger M, Bärlocher L. Carbon monoxide poisoning following use of a water pipe/hookah. Dtsch Arztebl Int. 2014;111(40):674-9.

979. Shanks KG, Behonick GS. Detection of carfentanil by LC-MS-MS and reports of associated fatalities in the USA. J Anal Toxicol. 2017;41(6):466-72.

980. Swanson DM, Hair LS, Strauch Rivers SR, Smyth BC, Brogan SC, Ventoso AD, et al. Fatalities involving carfentanil and furanyl fentanyl: Two case reports. J Anal Toxicol. 2017;41(6):498-502.

981. Wood DM, Davies S, Cummins A, Button J, Holt DW, Ramsey J, et al. Energy-1 ('NRG-1'): don't believe what the newspapers say about it being legal. BMJ Case Rep. 2011;2011:bcr0720103184.

982. Høiseth G, Bramness JG, Christophersen AS, Mørland J. Carisoprodol intoxications: a retrospective study of forensic autopsy material from 1992-2003. Int J Legal Med. 2007;121(5):403-9.

983. Bouchard NC, Forde J, Hoffman RS. Carvedilol overdose with quantitative confirmation. Basic Clin Pharmacol Toxicol. 2008;103(1):102-3.

984. Toennes SW, Kauert GF. Driving under the influence of khat – alkaloid concentrations and observations in forensic cases. Forensic Sci Int. 2004;140(1):85-90.

985. Toennes SW, Harder S, Schramm M, Niess C, Kauert GF. Pharmacokinetics of cathinone, cathine and norephedrine after the chewing of khat leaves. Br J Clin Pharmacol. 2003;56(1):125-30.

986. Anderson WH, Stafford DT, Bell JS. Disopyramide (Norpace) distribution at autopsy of an overdose case. J Forensic Sci. 1980;25(1):33-9.

987. Heitland P, Blohm M, Breuer C, Brinkert F, Achilles EG, Pukite I, et al. Application of ICP-MS and HPLC-ICP-MS for diagnosis and therapy of a severe intoxication with hexavalent chromium and inorganic arsenic. J Trace Elem Med Biol. 2017;41:36-40.

988. Tang MH, Ching CK, Tsui MS, Chu FK, Mak TW. Two cases of severe intoxication associated with analytically confirmed use of the novel psychoactive substances 25B-NBOMe and 25C-NBOMe. Clin Toxicol (Phila). 2014;52(5):561-5.

989. Andreasen MF, Telving R, Rosendal I, Eg MB, Hasselstrøm JB, Andersen. A fatal poisoning involving 25C-NBOMe. Forensic Sci Int. 2015;251:e1-8.

990. Toennes SW, Skopp G. Cocaine and benzoylecgonine – pharmacokinetic basics and window of detection with respect to §24a (2) StVG. Blutalkohol. 2013;50:113-21.

991. DFG (ed.). List of MAK and BAT Values. Deutsche Forschungsgemeinschaft. Wiley-VCH Verlag, Weinheim, Germany, 2014, pp. 227-250.

992. Spiller HA, Cutino L. Fatal cyclobenzaprine overdose with postmortem values. J Forensic Sci. 2003;48(4):883-4.

993. European Monitoring Centre for Drugs and Drug Addiction (EMCDDA), MT- 45: Report on the risk assessment of MT-45 in the framework of the Council Decision on new psychoactive substances (2015). http://www.emcdda.europa.eu/publications/risk-assessments/mt-45_en. Accessed 25 Feb 2020.

994. Rohanová M, Pálenícek T, Balíková M. Disposition of 4-bromo-2,5-dimethoxyphenethylamine (2C-B) and its metabolite 4-bromo-2-hydroxy-5-methoxyphenethylamine in rats after subcutaneous administration. Toxicol Lett. 2008;178(1):29-36.

995. Caspar AT, Brandt SD, Stoever AE, Meyer MR, Maurer HH. Metabolic fate and detectability of the new psychoactive substances 2-(4-bromo-2,5-dimethoxyphenyl)-N-[(2-methoxyphenyl)methyl]ethanamine (25B-NBOMe) and 2-(4-chloro-2,5-dimethoxyphenyl)-N-[(2-methoxyphenyl)methyl]ethanamine (25C-NBOMe) in human and rat urine by GC-MS, LC-MS^n, and LC-HR-MS/MS approaches. J Pharm Biomed Anal. 2017;134:158-69.

996. Hasan M, Schumacher G, Seekamp A, Taedken T, Siegmund W, Oswald S. LC-MS/MS method for the determination of clodronate in human plasma. J Pharm Biomed Anal. 2014;100:341-7.

997. Villikka K, Perttunen K, Rosnell J, Ikävalko H, Vaho H, Pylkkänen L. T he absolute bioavailability of clodronate from two different oral doses. Bone. 2002;31(3):418-21.

998. Mitchell DY, Heise MA, Pallone KA, Clay ME, Nesbitt JD, Russell DA, Melson CW. The effect of dosing regimen on the pharmacokinetics of risedronate. Br J Clin Pharmacol. 1999;48(4):536-42.

999. Chae JW, Seo JW, Mahat B, Yun HY, Baek IH, Lee BY, Kim DH, Kwon KI. A simple pharmacokinetic model of alendronate developed using plasma concentration and urine excretion data from healthy men. Drug Dev Ind Pharm. 2014;40(10):1325-9.

1000. Coukell AJ, Faulds D. Epirubicin. An updated review of its pharmacodynamic and pharmacokinetic properties and therapeutic efficacy in the management of breast cancer. Drugs. 1997;53(3):453-82.

1001. Oldfield V, Keating GM, Plosker G. Enfuvirtide: a review of its use in the management of HIV infection. Drugs. 2005;65(8):1139-60.

1002. Hengstmann JH, Weyand U, Dengler HJ. The physiological disposition of etilefrine in man. Eur J Clin Pharmacol. 1975;9(2-3):179-87.

1003. Turnidge J. Fusidic acid pharmacology, pharmacokinetics and pharmacodynamics. Int J Antimicrob Agents. 1999;12(Suppl. 2):S23-34.

1004. Tarcomnicu I, Gheorghe MC, Silvestro L, Savu SR, Boaru I, Tudoroniu A. High-throughput HPLC-MS/MS method to determine ibandronate in human plasma for pharmacokinetic applications. J Chromatogr B Analyt Technol Biomed Life Sci. 2009;877(27):3159-68.

1005. Bergner R, Dill K, Boerner D, Uppenkamp M. Elimination of intravenously administered ibandronate in patients on haemodialysis: a monocentre open study. Nephrol Dial Transplant. 2002;17(7):1281-5.

1006. Edwards LD. Buprenorphine in Wisconsin drivers: concerns for impairment? J Anal Toxicol. 2019;43:644-50.

1007. Mayer B. How much nicotine kills a human? Tracing back the generally accepted lethal dose to dubious self-experiments in the nineteenth century Arch Toxicol. 2014;88(1):5-7.

1008. Seo AD, Kim DC, Yu HJ, Kang MJ. Accidental ingestion of E-cigarette liquid nicotine in a 15-month-old child: an infant mortality case of nicotine intoxication. Korean J Pediatr. 2016;59(12):490-3.

1009. Chen BC, Bright SB, Trivedi AR, Valento M. Death following intentional ingestion of e-liquid. Clin Toxicol (Phila). 2015;53(9):914-6.

1010. Hughes A, Hendrickson RG. An epidemiologic and clinical descrption of e-cigarette toxicity. Clin Toxicol (Phila). 2019;57(4):287-93.

1011. Concheiro M, Chesser R, Pardi J, Cooper G. Postmortem toxicology of new synthetic opioids. Front Pharmacol. 2018;9:1210.

1012. Jönsson AK, Söderberg C, Espnes KA, Ahlner J, Eriksson A, Reis M, Druid H. Sedative and hypnotic drugs – fatal and non-fatal reference blood concentrations. Forensic Sci Int. 2014;236:138-45.

1013. Lora-Tamayo C, Tena T, Rodríguez A, Sancho J, Molina E. Intoxication due to 1,4-butanediol. Forensic Sci Int. 2003;133(3):256-9.

1014. Warrick BJ, Wilson J, Hedge M, Freeman S, Leonard K, Aaron C. Lethal serotonin syndrome after methylone and butylone ingestion. J Med Toxicol. 2012;8(1):65-8.

1015. Kraemer M, Boehmer A, Madea B, Maas A. Death cases involving certain new psychoactive substances: A review of the literature. Forensic Sci Int. 2019;298:186-267.

1016. Moosmann B, Bisel P, Auwärter V. Characterization of the designer benzodiazepine diclazepam and preliminary data on its metabolism and pharmacokinetics. Drug Test Anal. 2014;6(7-8):757-63.

1017. Li A, Yuen VM, Goulay-Dufaÿ S, Sheng Y, Standing JF, Kwok PCL, Leung MKM, et al. Pharmacokinetic and pharmacodynamic study of intranasal and intravenous dexmedetomidine. Br J Anaesth. 2018;120(5):960-8.

1018. Usui K, Aramaki T, Hashiyada M, Hayashizaki Y, Funayama M. Quantitative analysis of 3,4-dimethylmethcathinone in blood and urine by liquid chromatography-tandem mass spectrometry in a fatal case. Leg Med (Tokyo). 2014;16(4):222-6.

1019. Hamel B, Audran M, Costa P, Bressolle F. Reversed-phase high-performance liquid chromatographic determination of enoxacin and 4-oxo-enoxacin in human plasma and prostatic tissue. Application to a pharmacokinetic study. J Chromatogr A. 1998;812(1-2):369-79.

1020. Iliuta IA, Lachance P, Ghannoum M, Bégin Y, Mac-Way F, Desmeules S, et al. Prediction and validation of the duration of hemodialysis sessions for the treatment of acute ethylene glycol poisoning. Kidney Int. 2017;92(2):453-60.

1021. Levine M, Curry SC, Ruha AM, Pizon AF, Boyer E, Burns J, et al. Ethylene glycol elimination kinetics and outcomes in patients managed without hemodialysis. Ann Emerg Med. 2012;59(6):527-31.

1022. Zaami S, Giorgetti R, Pichini S, Pantano F, Marinelli E, Busardò FP. Synthetic cathinones related fatalities: an update. Eur Rev Med Pharmacol Sci. 2018;22(1):268-74.

1023. Maskell PD, Smith PR, Cole R, Hikin L, Morley SR. Seven fatalities associated with ethylphenidate. Forensic Sci Int. 2016;265:70-4.

1024. Fracasso C, Confalonieri S, Garattini S, Caccia S. Single and multiple dose pharmacokinetics of etizolam in healthy subjects. Eur J Clin Pharmacol. 1991;40(2):181-5.

1025. Nakamae T, Shinozuka T, Sasaki C, Ogamo A, Murakami-Hashimoto C, Irie W, et al. Case report: Etizolam and its major metabolites in two unnatural death cases. Forensic Sci Int. 2008;182(1-3):e1-6.

1026. Kondo S, Fukasawa T, Yasui-Furukori N, Aoshima T, Suzuki A, Inoue Y, et al. Induction of the metabolism of etizolam by carbamazepine in humans. Eur J Clin Pharmacol. 2005;61(3):185-8.

1027. Benesch MGK, Iqbal SJ. Novel psychoactive substances: overdose of 3-fluorophenmetrazine (3-FPM) and etizolam in a 33-year-old man. BMJ Case Rep. 2018; pii: bcr-2018-224995.

1028. Tamama K, Lynch MJ. Newly emerging drugs of abuse. Handb Exp Pharmacol. 2019;Oct 9:doi: 10.1007/164_2019_260. [Epub ahead of print].

1029. Takasaki S, Yamaguchi H, Kawasaki Y, Kikuchi M, Tanaka M, Ito A, Mano N. Long-term relationship between everolimus blood concentration and clinical outcomes in Japanese patients with metastatic renal cell carcinoma: a prospective study. J Pharm Health Care Sci. 2019;5:6.

1030. Malhotra B, Guan Z, Wood N, Gandelman K. Pharmacokinetic profile of fesoterodine. Int J Clin Pharmacol Ther. 2008;46(11):556-63.

1031. Stuck AE, Kim DK, Frey FJ. Fleroxacin clinical pharmacokinetics. Clin Pharmacokinet. 1992;22(2):116-31.

1032. Trocóniz IF, Boland K, Staab A. Population pharmacokinetic/pharmacodynamic model for the sedative effects of flibanserin in healthy volunteers. Pharm Res. 2012;29(6):1518-29.

1033. Łukasik-Głębocka M, Sommerfeld K, Teżyk A, Zielińska-Psuja B, Panieński P, Żaba C. Flubromazolam – a new life-threatening designer benzodiazepine. Clin Toxicol (Phila). 2016;54(1):66-8.

1034. Kaiser C, Iwersen-Bergmann S, Kauert G. Kontroverse der tödlichen Intoxikation mit Flunitrazepam [in German]. Rechtsmedizin. 2009;19:85-9.

1035. Usui K, Fujita Y, Kamijo Y, Kokaji T, Funayama M. Identification of 5-fluoro ADB in human whole blood in four death cases. J Anal Toxicol. 2018;42(2):e21-5.

1036. Hasegawa K, Wurita A, Minakata K, Gonmori K, Nozawa H, Yamagishi I, et al. Postmortem distribution of MAB-CHMINACA in body fluids and solid tissues of a human cadaver. Forensic Toxicol. 2015;33(2):380-7.

1037. Shanks KG, Behonick GS. Death after use of the synthetic cannabinoid 5F-AMB. Forensic Sci Int. 2016;262:e21-4.

1038. Strehmel N, Vejmelka E, Kastner K, Roscher S, Tsokos M, Scholtis S. NPS-findings in forensic toxicology – three case reports. Toxichem Krimtech. 2017;84:199-204.

1039. Angerer V, Jacobi S, Franz F, Auwärter V, Pietsch J. Three fatalities associated with the synthetic cannabinoids 5F-ADB, 5F-PB-22, and AB-CHMINACA. Forensic Sci Int. 2017;281:e9-e15.

1040. Behonick G, Shanks KG, Firchau DJ, Mathur G, Lynch CF, Nashelsky M, et al. Four postmortem case reports with quantitative detection of the synthetic cannabinoid, 5F-PB-22. J Anal Toxicol. 2014;38(8):559-62.

1041. Karinen R, Tuv SS, Rogde S, Peres MD, Johansen U, Frost J, et al. Lethal poisonings with AH-7921 in combination with other substances. Forensic Sci Int. 2014;244:e21-4.

1042. Hoffmann O, Gommert LR, Egert M. Paradoxical cerebral cortical hyperexcitability following flupirtine overdose. J Toxicol Clin Toxicol. 2004;42(6):913-6.

1043. Lenz D, Rothschild M, Kroener L. Intoxications due to ingestion of gamma-butyrolactone: organ distribution of gamma-hydroxybutyric acid and gamma-butyrolactone. Ther Drug Monit. 2008;30(6):755-61.

1044. Andresen H, Bingel U, Streichert T, Schmoldt A, Zoerner AA, Tsikas D, et al. Severe glycerol intoxication after Menière's disease diagnostic – case report and overview of kinetic data. Clin Toxicol (Phila). 2009;47(4):312-6.

1045. Heitland P, Köster HD. Biomonitoring of 37 trace elements in blood samples from inhabitants of northern Germany by ICP-MS. J Trace Elem Med Biol. 2006;20(4):253-62.

1046. Dubois N, Demaret I, Ansseau M, Rozet E, Hubert P, Charlier C. Plasma level monitoring of the major metabolites of diacetylmorphine (heroin) by the "chasing the dragon" route in severe heroin addicts. Acta Clin Belg. 2013;68(5):359-67.

1047. Meatherall R, Lee C, Phillips S. Accidental death from hydromorphone ingestion. J Forensic Sci. 2011;56(Suppl. 1):271-4.

1048. Di Prospero NA, Sumner CJ, Penzak SR, Ravina B, Fischbeck KH, Taylor JP. Safety, tolerability, and pharmacokinetics of high-dose idebenone in patients with Friedreich ataxia. Arch Neurol. 2007;64(6):803-8.

1049. Pfeiffer S, Gunkelman S, Blackford M. Psychotropic exposures in pediatric patients: Symptomatic iloperidone and vilazodone ingestions. Clin Toxicol (Phila). 2015;53(3):188.

1050. Waldman W, Kała M, Lechowicz W, Gil D, Anand JS. Severe clinical toxicity caused by 25I-NBOMe confirmed analytically using LC-MS-MS method. Acta Biochim Pol. 2018;65(4):567-71.

1051. Lowe LM, Peterson BL, Couper FJ. A case review of the first analytically confirmed 25I-NBOMe-related death in Washington State. J Anal Toxicol. 2015;39(8):668-71.

1052. Walterscheid JP, Phillips GT, Lopez AE, Gonsoulin ML, Chen HH, Sanchez LA. Pathological findings in 2 cases of fatal 25I-NBOMe toxicity. Am J Forensic Med Pathol. 2014;35(1):20-5.

1053. Haustein KO, Hüller G. [Bioavailability of etilefrine from Thomasin and Thomasin sustained-release tablets] [Article in German]. Pharmazie. 1985;40(11):776-8.

1054. Rominger KL, Hitzenberger G. Pharmacokinetic comparison of etilefrine to its prodrug, the stearic acid ester of etilefrine. Int J Clin Pharmacol Ther. 1980;18(4):150-7.

1055. Battistella M. Fomepizole as an antidote for ethylene glycol poisoning. Ann Pharmacother. 2002;36(6):1085-9.

1056. Brent J. Fomepizole for ethylene glycol and methanol poisoning. N Engl J Med. 2009;360(21):2216-23.

1057. Druteika DP, Zed PJ, Ensom MH. Role of fomepizole in the management of ethylene glycol toxicity. Pharmacotherapy. 2002;22(3):365-72.

1058. Mycyk MB, Leikin JB. Antidote review: fomepizole for methanol poisoning. Am J Ther. 2003;10(1):68-70.

1059. Roberts DM, Buckley NA, Mohamed F, Eddleston M, Goldstein DA, Mehrsheikh A, et al. A prospective observational study of the clinical toxicology of glyphosate-containing herbicides in adults with acute self-poisoning. Clin Toxicol (Phila). 2010;48(2):129-36.

1060. Bradberry SM, Proudfoot AT, Vale JA. Glyphosate poisoning. Toxicol Rev. 2004;23(3):159-67.

1061. Schmidt-Redemann B, Brenneisen P, Schmidt-Redemann W, Gonda S. The determination of pharmacokinetic parameters of ketotifen in steady state in young children. Int J Clin Pharmacol Ther Toxicol. 1986;24(9):496-8.

1062. Chen Chen X, Zhong D, Liu D, Wang Y, Han Y, Gu J. Determination of ketotifen and its conjugated metabolite in human plasma by liquid chromatography/tandem mass spectrometry: application to a pharmacokinetic study. Rapid Commun Mass Spectrom. 2003;17(22):2459-63.

1063. Wagmann L, Hemmer S, Caspar AT, Meyer MR. Method development for quantitative determination of seven statins including four active metabolites by means of high-resolution tandem mass spectrometry applicable for adherence testing and therapeutic drug monitoring. Clin Chem Lab Med. 2020;58(5):664-72.

1064. Gundersen POM, Helland A, Spigset O, Hegstad S. Quantification of 21 antihypertensive drugs in serum using UHPLC-MS/MS. J Chromatograph B Analyt Technol Biomed Life Sci. 2018;1089:84-93.

1065. Drevin G, Palayer M, Compagnon P, Zabet D, Jousset N, Briet M, Abbara C. A fatal case report of acute yohimbine intoxication. Forensic Toxicol. 2020;38:287-91.

1066. Parr MK, Ambrosio G, Wuest B, Mazzarino M, de la Torre X, Sibilia F, Joseph JF, Diel P, Botrè F. Targeting the administration of ecdysterone in doping control samples. Forensic Toxicol. 2019;38:172-84.

1067. De Decker K, Cordonnier J, Jacobs W, Coucke V, Schepens P, Jorens PG. Fatal intoxication due to tramadol alone: Case report and review of the literature. Forensic Sci Int. 2008;175:79-82.

1068. Schiemsky T, Vundelinckx G, Croes K, Penders J, Desmet K, Pauwels S, Vermeersch P. An unconscious man with profound drug-induced hypoglycaemia. Biochem Med (Zagreb). 2020;30(1):010802.

1069. Stevens DC, Kleiman MB, Lietman PS, Schreiner RL. Exchange transfusion in acute chloramphenicol toxicity. J Pediatr. 1981;99(4):651-3.

1070. Fels H, Krueger J ,Sachs H, Musshoff F, Graw M, Rieder G, Stoever A. Two fatalities associated with synthetic opioids: AH-7921 and MT-45. Forensic Sci Int. 2017;277:e30-5.

1071. Salle S, Bodeau S, Dhersin A, Ferdonnet M, Goncalves R, Lenski M, et al. Novel synthetic opioids: A review of the literature. Toxicol Analyt Clin. 2019;31:298-316.

1072. Ojanperä I, Gergov M, Rasanen I, Lunetta P, Toivonen S, Tiainen E, Vuori E. Blood levels of 3-methylfentanyl in 3 fatal poisoning cases. Am J Forensic Med Pathol. 2006;27(4):328-31.

1073. Palmiere C, Bévalot F, Malicier D, Grouzmann E, Fracasso T, Fanton L. A case of suicide by self-injection of adrenaline. Forensic Sci Med Pathol. 2015:11(3): 421-6.

1074. Sommer S, Nau R, Wieland E, Prange HW. Pharmacokinetics of glycerol administered orally in healthy volunteers. Arzneimittelforschung – Drug Res. 1993;43(7):744-7.

1075. Cantrell FL, Sherrard J, Andrade M, Schaber B, McIntyre IM. A pediatric fatality due to accidental hydromorphone ingestion. Clin Toxicol (Phila). 2017;55(1):60-2.

1076. Le Blaye I, Donatini B, Hall M and Krupp P. Acute ketotifen overdosage. A review of present clinical experience. Drug Saf. 1992;7(5):387-92.

1077. Vande-Casteele N, Ferrante M, Van Assche G, Ballet V, Compernolle G, Van Steen K, et al.. Trough concentrations of infliximab guide dosing for patients with inflammatory bowel disease. Gastroenterology. 2015;148(7):1320-9.e3.

1078. Miyazaki T, Hanaoka K, Namiki A, Ogawa S, Kitajima T, Hosokawa T, et al. Efficacy, safety and pharmacokinetic study of a novel fentanyl-containing matrix transdermal patch system in Japanese patients with cancer pain. Clin Drug Investig. 2008;28(5):313-25.

1079. Fentanyl-ratiopharm Matrixpflaster (transdermal patch) and associated names. Summary of Product Characteristics [European Medicines Agency]. https://www.ema.europa.eu/en/documents/referral/fentanyl-ratiopharm-matrixpflaster-article-29-referral-annex-i-ii-iii_en.pdf. Accessed 6 Jan 2020.

1080. Solassol I, Bressolle F, Caumette L, Garcia F, Poujol S, Culine S, Pinguet F. Inter- and intraindividual variabilities in pharmacokinetics of fentanyl after repeated 72-hour transdermal applications in cancer pain patients. Ther Drug Monit. 2005;27(4):491-8.

1081. Mogler L, Halter S, Wilde M, Franz F, Auwärter V. Human phase I metabolism of the novel synthetic cannabinoid 5F-CUMYL-PEGACLONE. Forensic Toxicol. 2019;37(1):154-63.

1082. Giorgetti A, Mogler L, Halter S, Haschimi B, Alt A, Rentsch D, et al. Four cases of death involving the novel synthetic cannabinoid 5F-Cumyl-PEGACLONE. Forensic Toxicol. 2019; Epub December 24.

1083. Jones AW, Holmgren A. Concentrations of cocaine and benzoylecgonine in femoral blood from cocaine-related deaths compared with venous blood from impaired rivers. J Anal Toxicol. 2014;38(1):46-51.

1084. Reis M, Aamo T, Ahlner J, Druid H. Reference concentrations of antidepressants. A compilation of postmortem and therapeutic levels. J Anal Toxicol. 2007;31(5):254-64.

1085. Reis M, Aamo T, Spigset O, Ahlner J. Serum concentrations of antidepressant drugs in a naturalistic setting: compilation based on a large therapeutic drug monitoring database. Ther Drug Monit. 2009;31(1):42-56.

1086. Benoist GE, van der Meulen E, van Oort IM, Beumer JH, Somford DM, Schalken JA, et al. Development and validation of a bioanalytical method to

quantitate enzalutamide and its active metabolite N-desmethylenzalutamide in human plasma: application to clinical management of patients with metastatic castration-resistant prostate cancer. Ther Drug Monit. 2018;40(2):222-9.

1087. Da Ros L, Squassante L, Milleri S. Dose linearity of lacidipine pharmacokinetics after single and repeated doses in healthy volunteers. Clin Pharmacokinet. 2003:42:99-106.

1088. Gerloff J, Mignot A, Barth H, Heintze K. Pharmakokinetics and absolute bioavailability of lansoprazole. Eur J Clin Pharmacol. 1996;50:293-7.

1089. Dahut WL, Aragon-Ching JB, Woo S, Tohnya TM, Gulley JL, Arlen PM, Wright JJ, Ventiz J, Figg WD. Phase I study of oral lenalidomide in patients with refractory metastatic cancer. J Clin Pharmacol. 2009;49(6):650-60.

1090. Shida S, Takahashi N, Miura M, Niioka T, Matsumoto M, Hagihara M, et al. A limited sampling model to estimate exposure to lenalidomide in multiple myeloma patients. Ther Drug Monit. 2014;36(4):505-9.

1091. Luyckx M, Rousseau F, Cazin M, Brunet C, Cazin JC, Haguenoer JM, et al. Pharmacokinetics of levamisole in healthy subjects and cancer patients. Eur J Drug Metab Pharmacokinet. 1982;7(4):247-54.

1092. Reid JM, Kovach JS, O'Connell MJ, Bagniewski PG, Moertel CG. Clinical and pharmacokinetic studies of high-dose levamisole in combination with 5-fluorouracil in patients with advanced cancer. Cancer Chemother Pharmacol. 1998;41(6):477-84.

1093. Zazgornik J, Huang ML, Van Peer A, Woestenborghs R, Heykants J, Stephen A. Pharmacokinetics of orally administered levocabastine in patients with renal insufficiency. J Clin Pharmacol. 1993;33(12):1214-8.

1094. Dahl SG, Strandjord RE, Sigfusson S. Pharmacokinetics and relative bioavailability of levomepromazine after repeated administration of tablets and syrup. Eur J Clin Pharmacol. 1977;11:305-10.

1095. Iwersen-Bergmann S, Jungen H, Andresen-Streichert H, Müller A, Elakkary S, Püschel K, Heinemann A. Intravenous methadone application as a serious risk factor for an overdose death: methadone-related fatalities in Hamburg from 2007 to 2012. Int J Legal Med. 2014;128(5):751-64.

1096. Lucek R, Dixon R. Quantitation of levorphanol in plasma using high-performance liquid chromatography with electrochemical detection. J Chromatogr. 1985;341(1):239-43.

1097. Christofides ND, Sheehan CP. Multicenter evaluation of enhanced chemiluminescence labeled-antibody immunoassay (Amerlite-MAB) for free thyroxine. Clin Chem. 1995;41(1):24-31.

1098. Nukui Y, Hatakeyama S, Okamoto K, Yamamoto T, Hisaka A, Suzuki H, et al. High plasma linezolid concentration and impaired renal function affect

development of linezolid-induced thrombocytopenia. J Antimicrob Chemother. 2013;68(9):2128-33.

1099. Münst GJ, Karlaganis G, Bircher J. Plasma concentrations of mebendazole during treatment of echinococcosis: preliminary results. Eur J Clin Pharmacol. 1980;17(5):375-8.

1100. Krause W, Schwartzkopff W. Plasma levels of mepindolol in healthy volunteers after oral doses of mepindolol sulphate. Arzneimittelforschung. 1983;33(9):1306-7.

1101. Maskell PD, De Paoli G, Seetohul LN, Pounder DJ. Meptazinol and ethanol: a fatal intoxication. J Anal Toxicol. 2012;36(1):69-73.

1102. Kielbasa W, Helton DL. A new era for migraine: Pharmacokinetic and pharmacodynamic insights into monoclonal antibodies with a focus on galcanezumab, an anti-CGRP antibody. Cephalalgia. 2019;39(10):1284-97.

1103. Kielbasa W, Quinlan T. Population pharmacokinetics of galcanezumab, an anti-CGRP antibody, following subcutaneous dosing to healthy individuals and patients with migraine. J Clin Pharmacol. 2020;60(2):229-39.

1104. EMA. Ajovy® EPAR/Summary of Product Characteristics. https://www.ema.europa.eu/en/documents/product-information/ajovy-epar-product-information_en.pdf. Accessed 17 Jan 2020.

1105. EMA. Aimovig® EPAR/Summary of Product Characteristics. https://www.ema.europa.eu/en/documents/product-information/aimovig-epar-product-information_en.pdf. Accessed 17 Jan 2020.

1106. de Hoon J, Van Hecken A, Vandermeulen C, Yan L, Smith B, Chen JS, et al. Phase I, randomized, double-blind, placebo-controlled, single-dose, and multiple-dose studies of erenumab in healthy subjects and patients with migraine. Clin Pharmacol Ther. 2018;103(5):815-25.

1107. Lauven PM, Schwilden H, Stoeckel H. Threshold hypnotic concentration of methohexitone. Eur J Clin Pharmacol. 1987;33(3):261-5.

1108. Shaikh N, Sardar M, Raj R, Jariwala P. A rapidly fatal case of low-dose methotrexate toxicity. Case Rep Med. 2018;2018:9056086.

1109. Gwilt PR, Pankaskie MC, Thornburg JE, Zustiak R, Shoenthal DR. Pharmacokinetics of methyprylon following a single oral dose. J Pharm Sci. 1985;74(9):1001-3.

1110. Contos DA, Dixon KF, Guthrie RM, Gerber N, Mays DC. Nonlinear elimination of methyprylon (noludar) in an overdosed patient: correlation of clinical effects with plasma concentration. J Pharm Sci. 1991;80(8):768-71.

1111. Taylor DC, Cresswell PR, Pepper ES. The excretion and metabolism of metiamide in the rat, dog, and man. Drug Metab Dispos. 1979;7(6):393-8.

1112. Brotherton WP, Matteo RS. Pharmacokinetics and pharmacodynamics of metocurine in humans with and without renal failure. Anesthesiology. 1981;55(3):273-6.

1113. Jia Y, Zhang Y, Wang C, Wang Z, Liu Y, Wang J, Wen A. An improved LC-MS/MS method for quantitative determination of metolazone in human plasma and its application to a pharmacokinetic study. Biomed Chromatogr. 2011;25(10):1138-43.

1114. Kekkonen R, Heikinheimo O, Mandelin E, Lähteenmäki P. Pharmacokinetics of mifepristone after low oral doses. Contraception. 1996;54(4):229-34.

1115. Fanton L, Bévalot F, Grait H, Le Meur C, Gaillard Y, Malicier D. Fatal intoxication with milnacipran. J Forensic Leg Med. 2008;15(6):388-90.

1116. Poff SW, Rose SR. Minoxidil overdose with ECG changes: case report and review. J Emerg Med. 1992;10(1):53-7.

1117. Eltink C, Lee J, Schaddelee M, Zhang W, Kerbusch V, Meijer J, et al. Single dose pharmacokinetics and absolute bioavailability of mirabegron, a β_3-adrenoceptor agonist for treatment of overactive bladder. Int J Clin Pharmacol Ther. 2012;50(11):838-50.

1118. Frye LJ, Byrne ME, Winikoff B. A crossover pharmacokinetic study of misoprostol by the oral, sublingual and buccal routes. Eur J Contracept Reprod Health Care. 2016;21(4):265-8.

1119. Davi H, Bonnet JM, Berger Y. Disposition of minaprine in animals and in human extensive and limited debrisoquine hydroxylators. Xenobiotica. 1992;22(2):171-84.

1120. Roynard JL, Pourriat JL, Fournier JL, Hoang P, Sordelet D, Cupa M. [Fatal poisoning by minaprine]. Presse Med. 1989;18(19):986.

1121. Flores-Guerrero JL, Minovic I, Groothof D, Gruppen EG, Riphagen IJ, Kootstra-Ros J, et al. Association of plasma concentration of vitamin B12 with all-cause mortality in the general population in the Netherlands. JAMA Netw Open. 2020;3(1):e1919274.

1122. Matta MK, Florian J, Zusterzeel R, Nageswara RP, Patel V, Volpe DA, et al. Effect of sunscreen application on plasma concentration of sunscreen active ingredients. A randomized clinical trial. JAMA. 2020;323(3):256-67.

1123. Ahmadimanesh M, Shadnia S, Rouini MR, Sheikholeslami B, Ahsani Nasab S, Ghazi-Khansari M. Correlation between plasma concentrations of tramadol and its metabolites and the incidence of seizure in tramadol-intoxicated patients. Drug Metab Pers Ther. 2018;33(2):75-83.

1124. Gioia S, Lancia M, Bacci M, Suadoni F. Two Fatal Intoxications Due to Tramadol Alone: Autopsy Case Reports and Review of the Literature. Am J Forensic Med Pathol. 2017;38(4):345-8.

1125. Riedel F, von Stockhausen HB. Severe cerebral depression after intoxication with tramadol in a 6-month-old infant. Eur J Clin Pharmacol. 1984;26(5):631-2.

1126. Radünz L, Reuter H, Andresen-Streichert H. Modafinil in forensic and clinical toxicology - case reports, analytics and literature. J Anal Toxicol. 2018;42(5):353-9.

1127. Isbister GK, Hackett LP, Dawson AH, Whyte IM, Smith AJ. Moclobemide poisoning: toxicokinetics and occurrence of serotonin toxicity. Br J Clin Pharmacol. 2003;56(4):441-50.

1128. Ostrowski J, Resag K. Pharmacokinetics of molsidomine in humans. Am Heart J. 1985;109(3 Pt 2):641-3.

1129. Momcilović B. A case report of acute human molybdenum toxicity from a dietary molybdenum supplement - a new member of the "Lucor metallicum" family. Arh Hig Rada Toksikol. 1999;50(3):289-97.

1130. Cheng H, Leff JA, Amin R, Gertz BJ, De Smet M, Noonan N, et al. Pharmacokinetics, bioavailability, and safety of montelukast sodium (MK-0476) in healthy males and females. Pharm Res. 1996;13(3):445-8.

1131. Siddoway LA, Schwartz SL, Barbey JT, Woosley RL. Clinical pharmacokinetics of moricizine. Am J Cardiol. 1990;65(8):21D-25D; discussion 68D-71D.

1132. Woosley RL, Morganroth J, Fogoros RN, McMahon FG, Humphries JO, Mason DT, Williams RL. Pharmacokinetics of moricizine HCl. Am J Cardiol. 1987;60(11):35F-39F.

1133. Hooks MA, Wade CS, Millikan WJ Jr. Muromonab CD-3: a review of its pharmacology, pharmacokinetics, and clinical use in transplantation. Pharmacotherapy. 1991;11(1):26-37.

1134. Alloway R, Kotb M, Hathaway D, Ohman M, Strain S, Gaber AO. The pharmacokinetic profile of standard and low-dose OKT3 induction immunosuppression in renal transplant recipients. Transplantation. 1994;58(2):249-53.

1135. Bebarta VS, Heard K, Nadelson C. Lack of toxic effects following acute overdose of cellcept (mycophenolate mofetil). J Toxicol Clin Toxicol. 2004;42(6):917-9.

1136. Weaver ML, Orwig BA, Rodriguez LC, Graham ED, Chin JA, Shapiro MJ, et al. Pharmacokinetics and metabolism of nateglinide in humans. Drug Metab Dispos. 2001;29(4 Pt 1):415-21.

1137. Campoli-Richards DM1, Chaplin S, Sayce RH, Goa KL. Netilmicin. A review of its antibacterial activity, pharmacokinetic properties and therapeutic use. Drugs. 1989;38(5):703-56.

1138. Ikegaya H, Kobayashi M, Sakurada K, Takeichi H, Yoshida K, Iwase H. Detection of the calcium antagonist nicardipine and its metabolites by gas chromatography-mass spectrometry. Forensic Sci Int. 2002;130(1):25-8.

1139. Terakawa M, Tokuma Y, Shishido A, Noguchi H. Pharmacokinetics of nilvadipine in healthy volunteers. J Clin Pharmacol. 1987;27(2):111-7.

1140. Wijma RA, Huttner A, Koch BCP, Mouton JW, Muller AE. Review of the pharmacokinetic properties of nitrofurantoin and nitroxoline. J Antimicrob Chemother. 2018;73(11):2916-26.

1141. Hengstmann JH, Goronzy J. [Enteral resorption and bioavailability of norfenefrine]. [in German]. Arzneimittelforschung. 1980;30(12):2164-7.

1142. Thiermann H, Mast U, Klimmek R, Eyer P, Hibler A, Pfab R, et al. Cholinesterase status, pharmacokinetics and laboratory findings during obidoxime therapy in organophosphate poisoned patients. Hum Exp Toxicol. 1997;16(8):473-80.

1143. Kohler RB, Arkins N, Tack KJ. Accidental overdose of intravenous ofloxacin with benign outcome. Antimicrob Agents Chemother. 1991;35(6):1239-40.

1144. Skopp G, Miltner E, Aderjan R. Fatal poisoning with the antidepressive agent opipramol. Forensic Sci Int. 1996;77(1-2):45-51.

1145. Ochs HR, Greenblatt DJ, Verburg-Ochs B, Locniskar A. Comparative single dose kinetics of oxazolam, prazepam, and clorazepate: three precursors of desmethyldiazepam. J Clin Pharmacol**.** 1984;24(10):446-451.

1146. Adams MP, Ahdieh H. Pharmacokinetics and dose-proportionality of oxymorphone extended release and its metabolites: results of a randomized crossover study. Pharmacotherapy. 2004;24(4):468-76.

1147. Kim TY, Kim DW, Chung JY, Shin SG, Kim SC, Heo DS, et al. Phase I and pharmacokinetic study of Genexol-PM, a cremophor-free, polymeric micelle-formulated paclitaxel, in patients with advanced malignancies. Clin Cancer Res. 2004;10(11):3708-16.

1148. White SJ, Rumack BH. The acetaminophen toxicity equations: "solutions" for acetaminophen toxicity based on the Rumack-Matthew nomogram. Ann Emerg Med. 2005;45(5):563-4.

1149. Sivilotti ML, Good AM, Yarema MC, Juurlink DN, Johnson DW. A new predictor of toxicity following acetaminophen overdose based on pretreatment exposure. Clin Toxicol (Phila). 2005;43(4):229-34.

1150. Beermann B, Groschinsky-Grind M. Pharmacokinetics of hydrochlorothiazide in man. Eur J Clin Pharmacol. 1977;12(4):297-303.

1151. Moon SJ, Jeon JY, Yu KS, Kim MG. Pharmacokinetic interaction among telmisartan, amlodipine, and hydrochlorothiazide after a single oral administration in healthy male subjects. Clin Ther. 2019;41(11):2273-82.

1152. Borgström L, Johansson CG, Larsson H, Lenander R. Pharmacokinetics of bendroflumethiazide after low oral doses. J Pharmacokinet Biopharm. 1981;9(4):431-41.

1153. Borgström L, Johansson CG, Larsson H, Lenander R. Pharmacokinetics of propranolol. J Pharmacokinet Biopharm. 1981;9(4):419-29.

1154. Cai HL, Deng Y, Fang PF, Cao S, Hou ZY, Wu YQ, et al. A sensitive LC-MS/MS method for analysis of pericyazine in presence of 7-hydroxypericyazine and pericyazine sulphoxide in human plasma and its application to a comparative bioequivalence study in Chinese healthy volunteers. J Pharm Biomed Anal. 2017;135:67-74.

1155. Lelièvre B, Drouillard I, Thill C, Le Roux G, Bruneau C, Mahé J, et al. Severe poisoning with naproxen causing coagulopathy. Basic Clin Pharmacol Toxicol. 2019, Nov 19.

1156. Käferstein H, Sticht G, Pluisch F. [Poisoning by perazine – organ distribution and interpretation]. [in German]. Arch Kriminol. 2000;206(3-4):82-7.

1157. Maskell PD, De Paoli G, Nitin Seetohul L, Pounder DJ. Phenazepam: the drug that came in from the cold. J Forensic Leg Med. 2012;19(3):122-5.

1158. Rudolph GR, Miksic JR, Levitt MJ.GLC Determination of phendimetrazine in human plasma, serum, or urine. J Pharm Sci. 1983;72(5):519-21.

1159. Esnault P, Prunet B, Lacroix G, D'Aranda E, Gaillard Y, Boret H. Instantaneous rigor after fatal pholcodine intoxication. Br J Clin Pharmacol. 2014;77(3):578-9.

1160. Sallee FR, Pollock BG, Stiller RL, Stull S, Everett G, Perel JM. Pharmacokinetics of pimozide in adults and children with Tourette's syndrome. J Clin Pharmacol. 1987;27(10):776-81.

1161. Pacifici GM, Placidi GF, Fornaro P, Gomeni R. Pinazepam: a precursor of N-desmethyldiazepam. Eur J Clin Pharmacol. 1982;22(3):225-8.

1162. Jang SH, Colangelo PM, Gobburu JV. Exposure-response of posaconazole used for prophylaxis against invasive fungal infections: evaluating the need to adjust doses based on drug concentrations in plasma. Clin Pharmacol Ther. 2010;88(1):115-9.

1163. Jung H, Medina R, Castro N, Corona T, Sotelo J. Pharmacokinetic study of praziquantel administered alone and in combination with cimetidine in a single-day therapeutic regimen. Antimicrob Agents Chemother. 1997;41(6):1256-9.

1164. Wesson DR. Propylhexdrine. Drug Alcohol Depend. 1986;17(2-3):273-8.

1165. Volz M, Kellner HM. Kinetics and metabolism of pyrazolones (propyphenazone, aminopyrine and dipyrone). Br J Clin Pharmacol. 1980;10(Suppl. 2):299S-308S.

1166. Okonek S. Intoxication with pyrazolones. Br J Clin Pharmacol. 1980;10(Suppl. 2):385S-90S.

1167. Wu M, Schmitt G, Mattern R. [Suicide with prothipendyl]. [Article in German]. Arch Kriminol. 1994;193:159-62.

1168. Sticht G, Käferstein H. Detection of psilocin in body fluids. Forensic Sci Int. 2000;113(1-3):403-7.

1169. Imran M, Shafi H, Mahmood Z, Sarwar M, Usman HF, Tahir MA, Ashiq MZ. Fatal intoxications due to administration of isosorbide tablets contaminated with pyrimethamine. J Forensic Sci. 2016;61(5):1382-5.

1170. Conn HL Jr, Luchi RJ. Some cellular and metabolic considerations relating to the action of quinidine as a prototype antiarrhythmic agent. Am J Med. 1964;37:685-99.

1171. Skov L, Johansen SS, Linnet K. Postmortem quetiapine reference concentrations in brain and blood. J Anal Toxicol. 2015;39(7):557-61.

1172. Jerling M, Huan BL, Leung K, Chu N, Abdallah H, Hussein Z. Studies to investigate the pharmacokinetic interactions between ranolazine and ketoconazole, diltiazem, or simvastatin during combined administration in healthy subjects. J Clin Pharmacol. 2005;45(4):422-33.

1173. Cheng JW, Charland SL, Goldfarb S, Spinler SA. Effects of renal function on recainam pharmacokinetics and pharmacodynamics. Clin Pharmacol Ther. 1995;57(5):492-8.

1174. Egan TD, Kern SE, Muir KT, White J. Remifentanil by bolus injection: a safety, pharmacokinetic, pharmacodynamic, and age effect investigation in human volunteers. Br J Anaesth. 2004;92(3):335-43.

1175. Ruzilawati AB, Wahab MS, Imran A, Ismail Z, Gan SH. Method development and validation of repaglinide in human plasma by HPLC and its application in pharmacokinetic studies. J Pharm Biomed Anal. 2007;43(5):1831-5.

1176. Tripp SL, Williams E, Wagner WE Jr, Lukas G. A specific assay for subnanogram concentrations of reserpine in human plasma. Life Sci. 1975;16(7):1167-77.

1177. Fodstad O, Kvalheim G, Godal A, Lotsberg J, Aamdal S, Høst H, Pihl A. Phase I study of the plant protein ricin. Cancer Res. 1984;44(2):862-5.

1178. Lan NT, Thu NT, Barrail-Tran A, Duc NH, Lan NN, Laureillard D, et al. Randomised pharmacokinetic trial of rifabutin with lopinavir/ritonavir-antiretroviral therapy in patients with HIV-associated tuberculosis in Vietnam. PLoS One. 2014;9(1):e84866.

1179. Keung A, Eller MG, McKenzie KA, Weir SJ. Single and multiple dose pharmacokinetics of rifapentine in man: part II. Int J Tuberc Lung Dis. 1999;3(5):437-44.

1180. Mitchell DY, Eusebio RA, Sacco-Gibson NA, Pallone KA, Kelly SC, Nesbitt JD, et al. Dose-proportional pharmacokinetics of risedronate on single-dose oral administration to healthy volunteers. J Clin Pharmacol. 2000;40(3):258-65.

1181. Sennesael AL, Larock AS, Douxfils J, Elens L, Stillemans G, Wiesen M, et al. Rivaroxaban plasma levels in patients admitted for bleeding events: insights from a prospective study. Thromb J. 2018;16:28.

1182. McCoy EP, Mirakhur RK, Maddineni VR, Wierda JM, Proost JH. Pharmacokinetics of rocuronium after bolus and continuous infusion during halothane anaesthesia. Br J Anaesth. 1996;76(1):29-33.

1183. Bethke TD, Böhmer GM, Hermann R, Hauns B, Fux R, Mörike K, et al. Dose-proportional intraindividual single- and repeated-dose pharmacokinetics of roflumilast, an oral, once-daily phosphodiesterase 4 inhibitor. J Clin Pharmacol. 2007;47(1):26-36.

1184. Kirchheiner J, Thomas S, Bauer S, Tomalik-Scharte D, Hering U, Doroshyenko O, et al. Pharmacokinetics and pharmacodynamics of rosiglitazone in relation to CYP2C8 genotype. Clin Pharmacol Ther. 2006;80(6):657-67.

1185. Janson C, Boe J, Boman G, Mossberg B, Svedmyr N. Bronchodilator intake and plasma levels on admission for severe acute asthma. Eur Respir J. 1992;5(1):80-5.

1186. Breilh D, Honore PM, De Bels D, Roberts JA, Gordien JB, Fleureau C, et al. Pharmacokinetics and pharmacodynamics of anti-infective agents during continuous veno-venous hemofiltration in critically ill patients: lessons learned from an ancillary study of the IVOIRE trial. J Transl Int Med. 2019;7(4):155-69.

1187. Peridy E, Hamel JF, Rolland AL, Gohier B, Boels D. Quetiapine poisoning and factors influencing severity. J Clin Psychopharmacol. 2019;39(4):312-7.

1188. Samara E, Cao G, Locke C, Granneman GR, Dean R, Killian A. Population analysis of the pharmacokinetics and pharmacodynamics of seratrodast in patients with mild to moderate asthma. Clin Pharmacol Ther. 1997;62(4):426-35.

1189. Tracqui A, Miras A, Tabib A, Raul JS, Ludes B, Malicier D. Fatal overdosage with sildenafil citrate (Viagra): first report and review of the literature. Hum Exp Toxicol. 2002;21(11):623-9.

1190. Furukawa S, Kumagi T, Miyake T, Ueda T, Niiya T, Nishino K, et al. Suicide attempt by an overdose of sitagliptin, an oral hypoglycemic agent: a case report and a review of the literature. Endocr J. 2012;59(4):329-33.

1191. Firsov AA, Alekseeva ME, Lukomskiĭ GI, Umnova LV, Kashina LB. [Pharmacokinetic monitoring of aminoglycoside therapy: an optimal method of administration of individualized doses of gentamicin and sisomicin]. [in Russian]. Antibiot Khimioter. 1991;36(10):40-2.

1192. Kaplan SA, Weinfeld RE, Abruzzo CW, Lewis M. Pharmacokinetic profile of sulfisoxazole following intravenous, intramuscular, and oral administration to man. J Pharm Sci. 1972;61(5):773-8.

1193. Guentert TW, Heintz RC, Joly R. Overview on the pharmacokinetics of tenoxicam. Eur J Rheumatol Inflamm. 1987;9(2):15-25.

1194. Ketola RA, Ojanperä I. Summary statistics for drug concentrations in post-mortem femoral blood representing all causes of death. Drug Test Anal. 2019;11(9):1326-37.

1195. Nyberg L. Pharmacokinetic parameters of terbutaline in healthy man. An overview. Eur J Respir Dis Suppl. 1984;134:149-60.

1196. Holford NH, Clements P, Collier P, Orie NG, van Bork LE, Jonkman JH. Pharmacokinetics and pharmacodynamics of thiazinamium in asthmatic patients. Eur J Clin Pharmacol. 1987;33(3):237-42.

1197. Morris AA, Page RL 2nd, Baumgartner LJ, Mueller SW, MacLaren R, Fish DN, Kiser TH. Thiocyanate accumulation in critically ill patients receiving nitroprusside infusions. J Intensive Care Med. 2017;32(9):547-53.

1198. Schulz V. Clinical pharmacokinetics of nitroprusside, CN, thiosulfate and thiocyanate. Clin Pharmacokinet. 1984;9(3):239-51.

1199. Hung OR, Varvel JR, Shafer SL, Stanski DR. Thiopental pharmacodynamics. II. Quantitation of clinical and electroencephalographic depth of anesthesia. Anesthesiology. 1992;77(2):237-44.

1200. Proença P, Teixeira H, Pinheiro J, Monsanto PV, Vieira DN. Fatal intoxication with tianeptine (Stablon). Forensic Sci Int. 2007;170(2-3):200-3.

1201. Yuan B, Zhai N, Jiang X, Jin Y, Liu C, Li C, Xu H. Quantitative determination of tiopronin in human plasma by LC-MS/MS without derivatization. Biomed Chromatogr. 2012;26(7):839-43.

1202. Beer B, Libiseller K, Oberacher H, Pavlic M. A fatal intoxication case involving topiramate. Forensic Sci Int. 2010;202(1-3):e9-11.

1203. Clissold SP, Brogden RN. Piretanide. A preliminary review of its pharmacodynamic and pharmacokinetic properties, and therapeutic efficacy. Drugs. 1985;29(6):489-530.

1204. Marone C, Rivera B, Zwahlen H, Lahn W, Frey F. Efficacy and pharmacokinetics of piretanide in patients with congestive heart failure. Eur J Clin Invest. 1989;19(4):378-83.

1205. Ward A, Heel RC. Bumetanide. A review of its pharmacodynamic and pharmacokinetic properties and therapeutic use. Drugs. 1984;28(5):426-64.

1206. Friedel HA, Buckley MM. Torasemide. A review of its pharmacological properties and therapeutic potential. Drugs. 1991;41(1):81-103.

1207. Knauf H, Mutschler E. Clinical pharmacokinetics and pharmacodynamics of torasemide. Clin Pharmacokinet. 1998;34(1):1-24.

1208. Dunn CJ, Fitton A, Brogden RN. Torasemide. An update of its pharmacological properties and therapeutic efficacy. Drugs. 1995;49(1):121-42.

1209. Prichard BN, Brogden RN. Xipamide. A review of its pharmacodynamic and pharmacokinetic properties and therapeutic efficacy. Drugs. 1985;30(4):313-32.

1210. Furlong R, Brogden RN. Xamoterol. A preliminary review of its pharmacodynamic and pharmacokinetic properties, and therapeutic use. Drugs. 1988;36(4):455-74.

1211. Bastain W, Marlow HF. The effect of age and renal impairment on the pharmacokinetics of xamoterol. Br J Clin Pharmacol. 1989;28(Suppl. 1):65S-66S.

1212. Nicholls DP, Taggart AJ, McCann JP, Bastain W, Shanks RG. The pharmacokinetics of xamoterol in liver disease. Br J Clin Pharmacol. 1989;28(6):718-21.

1213. Bastain W, Boyce MJ, Stafford LE, Morton PB, Clarke DA, Marlow HF. Pharmacokinetics of xamoterol after intravenous and oral administration to volunteers. Eur J Clin Pharmacol. 1988;34(5):469-73.

1214. Koup JR, Tucker E, Thomas DJ, Kinkel AW, Sedman AJ, Dyer R, Sharoky M. A single and multiple dose pharmacokinetic and metabolism study of meclofenamate sodium. Biopharm Drug Dispos. 1990;11(1):1-15.

1215. Gall JA, Drummer OH, Landgren AJ. Death due to benzhexol toxicity. Forensic Sci Int. 1995;71(1):9-14.

1216. Matteo RS, Spector S, Horowitz PE. Relation of serum d-tubocurarine concentration to neuromuscular blockade in man. Anesthesiology. 1974;41(5):440-3.

1217. Bialer M, Haj-Yehia A, Barzaghi N, Pisani F, Perucca E. Pharmacokinetics of a valpromide isomer, valnoctamide, in healthy subjects. Eur J Clin Pharmacol. 1990;38(3):289-91.

1218. Stove CP, De Letter EA, Piette MH, Lambert WE. Fatality following a suicidal overdose with varenicline. Int J Legal Med. 2013;127(1):85-91.

1219. Cirimele V, Villain M, Pépin G, Ludes B, Kintz P. Screening procedure for eight quaternary nitrogen muscle relaxants in blood by high-performance liquid chromatography-electrospray ionization mass spectrometry. J Chromatogr B Analyt Technol Biomed Life Sci. 2003;789(1):107-13.

1220. Scott AK, Webster J, Petrie JC, Bastain W. The effect of age and cardiac failure on xamoterol pharmacokinetics. Br J Clin Pharmacol. 1988;25(2):165-8.

1221. Giampreti A, Lonati D, Locatelli C, Rocchi L, Campailla MT. Acute neurotoxicity after yohimbine ingestion by a body builder. Clin Toxicol (Phila). 2009;47(8):827-9.

1222. Lech T, Sadlik JK. Zinc in postmortem body tissues and fluids. Biol Trace Elem Res. 2011;142(1):11-7.

1223. Davies SJ, Westin AA, Castberg I, Lewis G, Lennard MS, Taylor S, Spigset O. Characterisation of zuclopenthixol metabolism by in vitro and therapeutic drug monitoring studies. Acta Psychiatr Scand. 2010;122(6):444-53.

1224. Gandhi AS, Wohlfarth A, Zhu M, Pang S, Castaneto M, Scheidweiler KB, Huestis MA. High-resolution mass spectrometric metabolite profiling of a novel synthetic designer drug, N-(adamantan-1-yl)-1-(5-fluoropentyl)-1H-indole-3-carboxamide (STS-135), using cryopreserved human hepatocytes and assessment of metabolic stability with human liver microsomes. Drug Test Anal. 2014;7(3):187-98.

1225. Castaneto MS, Wohlfarth A, Desrosiers NA, Hartman RL, Gorelick DA, Huestis MA. Synthetic cannabinoids pharmacokinetics and detection methods in biological matrices. Drug Metab Rev. 2015;47(2):124-74.

1226. Gautret P, Lagier JC, Parola P, Hoang VT, Meddeb L, Mailhe M, et al. Hydroxychloroquine and azithromycin as a treatment of COVID-19: results of an open-label non-randomized clinical trial. Int J Antimicrob Agents. 2020, March 17. DOI: 10.1016/j.ijantimicag.2020.105949.

1227. Foipan® tablets, Summary of Product Characteristics, 2019-11 [in Japanese]. https://www.pmda.go.jp/PmdaSearch/iyakuDetail/ResultDataSetPDF/180188_3999003F1297_1_09. Accessed 30 Mar 2020.

1228. Hiraku S, et al. Absorption and excretion of camostat orally administered to male rabbit and healthy subject [in Japanese]. Iyakuhin Kenkyu. 1982;13(3):756-65.

1229. Pharmaceuticals and Medical Devices Agency (PMDA). Report on Deliberation Results: Avigan Tablet 200 mg. Tokyo, Japan. 2014. https://www.pmda.go.jp/files/000210319.pdf. Accessed 31 Mar 2020.

1230. Trisenox (arsenic trioxide) Summary of Product Characteristics. https://www.ema.europa.eu/en/documents/product-information/trisenox-epar-product-information_en.pdf. Accessed 31 Mar 2020.

1231. Söderberg C, Tillmar A, Johansson A, Wernvik E, J¨onsson AK, Druid H. The importance of sample size with regard to the robustness of postmortem reference values. Forensic Sci Int. 2020, April 14. https://doi.org/10.1016/j.forsciint.2020.110292. Accessed 21 Apr 2020.

1232. Smit C, Peeters MYM, van den Anker JN, Knibbe CAJ. Chloroquine for SARS-CoV-2: implications of its unique pharmacokinetic and safety properties. Clin Pharmacokinet. 2020, April 18. https://doi.org/10.1007/s40262-020-00891-1. Accessed 21 Apr 2020.
